# Comparing the Biological Impact of Glatiramer Acetate with the Biological Impact of a Generic

**DOI:** 10.1371/journal.pone.0083757

**Published:** 2014-01-08

**Authors:** Fadi Towfic, Jason M. Funt, Kevin D. Fowler, Shlomo Bakshi, Eran Blaugrund, Maxim N. Artyomov, Michael R. Hayden, David Ladkani, Rivka Schwartz, Benjamin Zeskind

**Affiliations:** 1 Immuneering Corporation, Cambridge, Massachusetts, United States of America; 2 Teva Pharmaceutical Industries, Petach Tikva, Israel; Washington University, United States of America

## Abstract

For decades, policies regarding generic medicines have sought to provide patients with economical access to safe and effective drugs, while encouraging the development of new therapies. This balance is becoming more challenging for physicians and regulators as biologics and non-biological complex drugs (NBCDs) such as glatiramer acetate demonstrate remarkable efficacy, because generics for these medicines are more difficult to assess. We sought to develop computational methods that use transcriptional profiles to compare branded medicines to generics, robustly characterizing differences in biological impact. We combined multiple computational methods to determine whether differentially expressed genes result from random variation, or point to consistent differences in biological impact of the generic compared to the branded medicine. We applied these methods to analyze gene expression data from mouse splenocytes exposed to either branded glatiramer acetate or a generic. The computational methods identified extensive evidence that branded glatiramer acetate has a more consistent biological impact across batches than the generic, and has a distinct impact on regulatory T cells and myeloid lineage cells. In summary, we developed a computational pipeline that integrates multiple methods to compare two medicines in an innovative way. This pipeline, and the specific findings distinguishing branded glatiramer acetate from a generic, can help physicians and regulators take appropriate steps to ensure safety and efficacy.

## Introduction

The regulatory pathway for biosimilars has received significant attention, [1] with regulators in Europe and the US working to define appropriate ways to evaluate efficacy and safety. [2] As a result, the first application for a biosimilar monoclonal antibody is now under consideration. [3] A key consideration in all of these cases is the impact of the generic on the immune system, including its immunogenicity. [4] In addition to biosimilars, recent efforts have focused attention on the need for regulatory approaches to generic versions of non-biological complex drugs (NBCDs) [5] which include liposomal drugs, low-molecular weight heparins, iron-carbohydrate drugs, and glateramoids [6].

One such glatiramoid is glatiramer acetate (GA, Copaxone), which provides significant benefit for patients with multiple sclerosis (MS). MS is a complex neurological progressive disease that affects between 2 and 150 per 100,000 people worldwide. [7], [8] Its onset is typically in young adults, where the disease is manifested in demyelination of neural tissues such as the spinal cord, cerebellum or optic nerve which is believed to be driven at least in part by abnormalities in the immune system [7] including impairments in regulatory T cells (Treg) function [9].

GA’s putative mechanism of action involves the induction of tolerance via an interrelated set of immunomodulatory processes including binding to major histocompatibility complex (MHC) molecules, shifting from a Th1 cytokine profile to a Th2-biased anti-inflammatory profile, the activation of FoxP3+ regulatory T cells, and the inactivation of inflammatory monocytes [10]–[13].

GA is a copolymer of L-alanine, L-lysine, L-glutamic acid and L-tyrosine in a molar ratio of 4.2∶3.4∶1.4∶1.0 [11], [13] mimicking Myelin Basic Protein (MBP), which is proposed as one of the major autoantigens in MS. As GA is a glatiramoid class immunomodulator, [14] the heterogeneity of the various polypeptides in solution that make up GA cannot be fully characterized with current assay techniques. Furthermore, as response biomarkers and pharmacodynamic markers for the drug do not currently exist, [15] comparing GA to other glatiramers and characterizing differences in mechanism/efficacy has proven challenging. Previously we have compared GA to a generic GA (Glatimer®, Natco Pharma, Ltd., Hyderabad, India). [15] The previous analysis utilized high-throughput gene-expression assays to compare the transcriptional profiles of generic and GA in splenocytes of mice inoculated with GA and exposed *ex vivo* to generic or GA. The results focused on finding several specific genes (e.g., *FOXP3*) and functional pathways that were upregulated in GA relative to generic, concluding that the generic’s transcriptional profile differed significantly from GA. The lists of genes and pathways resulting from this traditional analysis were intriguing, but we recognized the need for new methods to integrate multiple lines of evidence and provide a clear and comprehensive picture of the differences in immunological impact that would contribute to the broader discussion of safety and efficacy.

Using GA and generic as examples, we sought to develop a thorough set of computational methods to go beyond lists of differentially expressed genes, using transcriptional profiles to robustly compare the immunological impact of two medicines. We focused on (1) comparing the variability of the two drugs across batches as measured by their transcriptional signatures; (2) characterizing the composition of the cell types modulated by each medicine; and (3) characterizing and explaining the immunomodulatory behaviors of the two drugs in the context of the genes they induce and suppress, as well as the immune cell types they modulate and the possible connections to clinical outcome.

## Methods

### Ethics Statement

All experimental procedures conformed to accepted ethical standards for use of animals in research and were in accordance with Committee for the Care and Use of Experimental Animals guidelines and approved by the Teva Institutional Animal Care and Use Committee.

### Experimental Design, Data Collection, and Pre-Processing

The experimental design, data collection, and pre-processing steps have been previously described. [15] In brief, 8 (Balb/c x SJL) F1 mice were injected with 100 µL of a 2.5 mg/mL solution GA reference standard in order to induce GA-reactive T cells. Our study design sought to model the situation in which a patient is initially treated with GA, and later switched to a generic. Mice were housed in individually ventilated cages for 3 days after immunization; mice were then sacrificed and their spleens were aseptically removed and placed on ice in RPMI 1640. The splenocytes were isolated, and these splenocytes were mixed with 125 µL per well of 80 µg/mL activator solutions for 24 hours. The activator solutions included 22 samples of GA reference standard, 34 samples from 30 different batches of GA (Copaxone® drug product, Teva Pharmaceutical Industries Ltd, Petach Tikva, Israel), 11 samples from 5 different batches of generic (Glatimer®, Natco Pharma, Ltd., Hyderabad, India), as well as a number of other glateramoids, deliberately modified or degraded GA, mannitol, and medium. Then the RNA was extracted and gene expression characterized by microarray using an Illumina WG-6_V2 chip. Samples were randomized on the chips to avoid batch effect (**Table S1**). Illumina’s BeadStudio software was utilized for image processing, quantification of signal intensity per bead, and background signal correction. The microarray data have been deposited in the Gene Expression Omnibus, under accession number GSE40566.

### Data Processing Steps

Starting with background-corrected bead-level signals, we quantile normalized the extracted data for all samples across all 46,547 probes via the “preprocessCore” package in R [16]. We then corrected for batch variation with ComBat [17] as implemented in the SVA package of R [18]. Each microarray’s chip designation was supplied a batch label; there were 18 batches in all. The labels for the treatments (i.e. drug product, reference standard…) were added as covariates. Principal Component Analysis (PCA) was utilized to check for outliers (**Figure S1A**).

### Variability Analysis Method

In order to identify probes with variability induced specifically by activation (as opposed to experimental noise), we sought to identify probes that were significantly more variable when activated with either GA or generic than medium. Using an F-test, we compared GA against Medium for each probe and compared generic against Medium. We then took the set of probes where either treatment comes up to be more variable than medium (union, passes in at least at least one). For those set of probes only, we compared the variability of GA across 34 samples representing 30 batches, to the variability of generic across 11 samples representing 5 batches, utilizing an F-test to measure significance of the differences between the probes.

### Tolerance Method for Assessing Process Variability

The goal of large scale industrial processing is to produce a large quantity of product which is of the same quality as that produced on a small scale. To assess the process consistency of GA and generic, we needed a standard of comparison. This standard of comparison was constructed by first identifying the top 1000 probes by absolute fold change of reference standard compared to the medium (**Table S2**). The list includes both upregulated and downregulated probes compared to medium. Probes were filtered such that ones upregulated by reference standard needed to have an average reference standard expression of 6.00 or higher and ones downregulated by reference standard needed to have an average medium expression of 6.00 or higher. This ensured that the list of 1000 probes were both significantly affected by reference standard and were sufficiently expressed to avoid noise associated with lowly expressed probes.

For each of the 1000 probes, the maximum and minimum expression observed by any reference standard sample was recorded. The range between the maximum and minimum expression observed for the reference standard served as the acceptable tolerance range for each probe. We then counted the number of samples from among the 34 GA samples representing 30 batches that fell within the acceptable tolerance range, and counted the number of samples from among the 11 generic samples representing 5 batches. We converted each result to a percentage of samples within range for all 1000 probes. These percentages were then sorted from smallest to largest separately for generic and GA and plotted against the integers, 1–1000. The net result was a plot that allowed for the determination for either drug of how many probes would fail to meet a given processing specification on the number of samples required to fall within the acceptable tolerance range for each probe. A visual representation of the method can be observed in **Figure S8**, which depicts scatter plots for Gpr83 vs. FoxP3 for both GA and generic. The red square in both plots is the acceptable tolerance range defined by the maximum and minimum reference standard expression for both probes. Five probes fall outside the acceptable tolerance range for GA, while twelve probes fall outside the range for generic.

### Variance Ratio Method

To measure relative differences in the variability among generic and GA samples, we utilized the sample variance as an unbiased estimate for the population variance for each set of treatments (GA and generic). Briefly, we divided the variance in the 11 generic samples representing 5 batches by the variance in the 34 GA samples representing 30 batches, obtaining a ratio for each probe in the Illumina microarray. The measure provided an intuitive comparison of the variance in each probe between treatments and this ratio is a basic statistic computed by the F-test [19]. We then sorted the probes from higher than 1 ratio (more variability in generic compared to GA) to lower than 1 (more variable in GA compared to generic).

### Coefficient of Variation Plots

To measure the relationship between probe intensity and probe variation, we calculated the log2 intensity of each probe after normalization/batch correction (averaging technical replicates to focus on biological variability rather than variability introduced by technical issues [20]). We also calculated the coefficient of variation, denoted CV, by dividing the standard deviation of each probe by its mean log2 intensity. We then plotted the relationship for all probes using the log2 intensity of the probe on the X-axis and the CV on the y-axis. The plots show a bias of probes with higher intensity to have lower CV and vice versa. Despite this bias, we still see that, as a class, generic-treated samples exhibit overall significantly higher variability (measure by F-test) compared to GA treated samples based on the number of probes that were highly variable between the two treatments and medium.

### Identification of Differentially Expressed Genes Using Multiple Parametric Tests

To find differentially expressed probes between generic and GA, we utilized various statistical tests at the probe level and merged the results across the different methods. First, we computed the statistical significance of differential expression between treatments using the Analysis of Variance (ANOVA) method for each probe [21], adjusting for multiple hypothesis testing using the Benjamini-Hochberg False Discovery Rate (FDR) correction [22]. Next, we utilized Linear Models for Microarray (LIMMA) data analysis [23], [24] R package, part of the Bioconductor framework [25], to compare generic and GA samples, fitting a linear model that adjusts for fixed effect from medium (Effect = (GA-generic) – (generic-Medium)). The coefficients for the linear model were tested for significance using a modified t-test (taking into account standard deviation) and the p-values for each probe were adjusted using FDR. [22] In parallel, we used Comparative Marker Selection as implemented in GenePattern [26] to directly compare probes between generic and GA. We applied two separate techniques within this framework; a traditional T-test and a Signal-to-Noise Ratio test (SNR). For each of these two tests, we adjusted the nominal p-values via FDR. For all four tests described, we used an adjusted threshold that was less than or equal to 0.05.

### Non-parametric/Wilcoxon

Because of natural variations of the distributions of probe expression in samples of generic and GA, we sought to identify differences via a non-parametric approach. For this, we used the Wilcoxon Rank Sum Test [27] as implemented in R (R version 2.15.1 (2012-06-22)) for each probe. Nominal p-values were FDR adjusted and only probes that were less than or equal to 0.05 were considered.

### GSEA with FoxP3 Target Gene Lists

In order to examine how genes downstream of the FoxP3 transcription factor were modulated by the two treatments, we utilized gene sets constructed by Zheng et al. [28] from FoxP3^+^ T-cells isolated from human thymus and periphery comprising genes that had FoxP3 binding sites by ChIP (Chromatin-Immuniprecipitation) and are differentially expressed relative to FoxP3^–^ T-cells. Orthology mapping from human to mouse was conducted using a map provided by the Mouse Genome Database. [29] We then utilized the Gene Set Enrichment Algorithm (GSEA) [30] to measure the enrichment of FoxP3 targets in genes upregulated in GA relative to Medium, and generic relative to Medium. Briefly, GSEA takes as input a gene set (in this case, the set of FoxP3 targets) and an expression matrix (the set of samples treated with either generic or GA or untreated/Medium), then it ranks genes based on their expression in the expression matrix for each class/treatment. GSEA then calculates an enrichment score for each geneset based on how overrepresented each geneset is at the extremes of expression (high or low expression) for each treatment.

### ANOVA-based Pattern Identification Method

We sought to identify probes matching specific patterns of expression across experimental conditions using a technique that referred to as the ANOVA-based Pattern Identification Method. For instance, one pattern of interest was where probes are only significantly affected by generic. In this pattern, the expression for a given probe should not be statistically different among cells treated by GA, reference standard, or Medium. In statistical terms, probes matching this pattern should have p-values for the comparisons between generic and GA (generic_A-generic_), generic and reference standard (p_generic-reference standard_), and generic and Medium (p_generic-Medium_) less than 0.05 and p-values for the comparisons between GA and Medium (generic_A-Medium_), GA and reference standard (generic_A-reference standard_), and reference standard and Medium (p_reference standard-Medium_) greater than 0.05.

To carry out the analysis in a general manner, we computed the 6 p-values required to do pairwise comparisons of all 4 conditions for all probes using the ANOVA1 function in MATLAB. We then identified sets of probes matching the desired pattern. In the example above, probes were identified as being only affected by generic if their 6 pairwise comparison p-values matched the following pattern (generic_A-generic_ <0.05, p_generic-reference standard_<0.05, p_generic-Medium_<0.05, generic_A-Medium_>0.05, generic_A-reference standard_>0.05, p_reference standard-Medium_>0.05).

### Cell Type Enrichment

To measure cell-type specificity in gene sets, we utilized Benita et al.’s enrichment tool [31] to calculate specificity scores relating each gene to each cell type in IMMGEN (Immunological Genome Project). [32] We then utilize a hyper-geometric test to calculate the significance of the summed specificity scores for the geneset across each cell type. Finally, we adjusted each p-value output by the hypergeometric enrichment using the Benjamini-Hochberg False Discovery Rate correction for multiple hypothesis testing [22].

### Functional Enrichment with MSigDB

To assess the functional significance of lists of genes (test set), we used version 3.1 of the database of MSigDB [30] as our reference set. We implemented a standard hypergeometic enrichment test with the additional criterion that at least three genes from our test set be in the reference set. We then applied the Benjamini-Hochberg correction procedure and used a significance threshold of 0.05.

## Results

### Variability Analysis: GA is Significantly More Consistent than Generic

**Figure 1:**
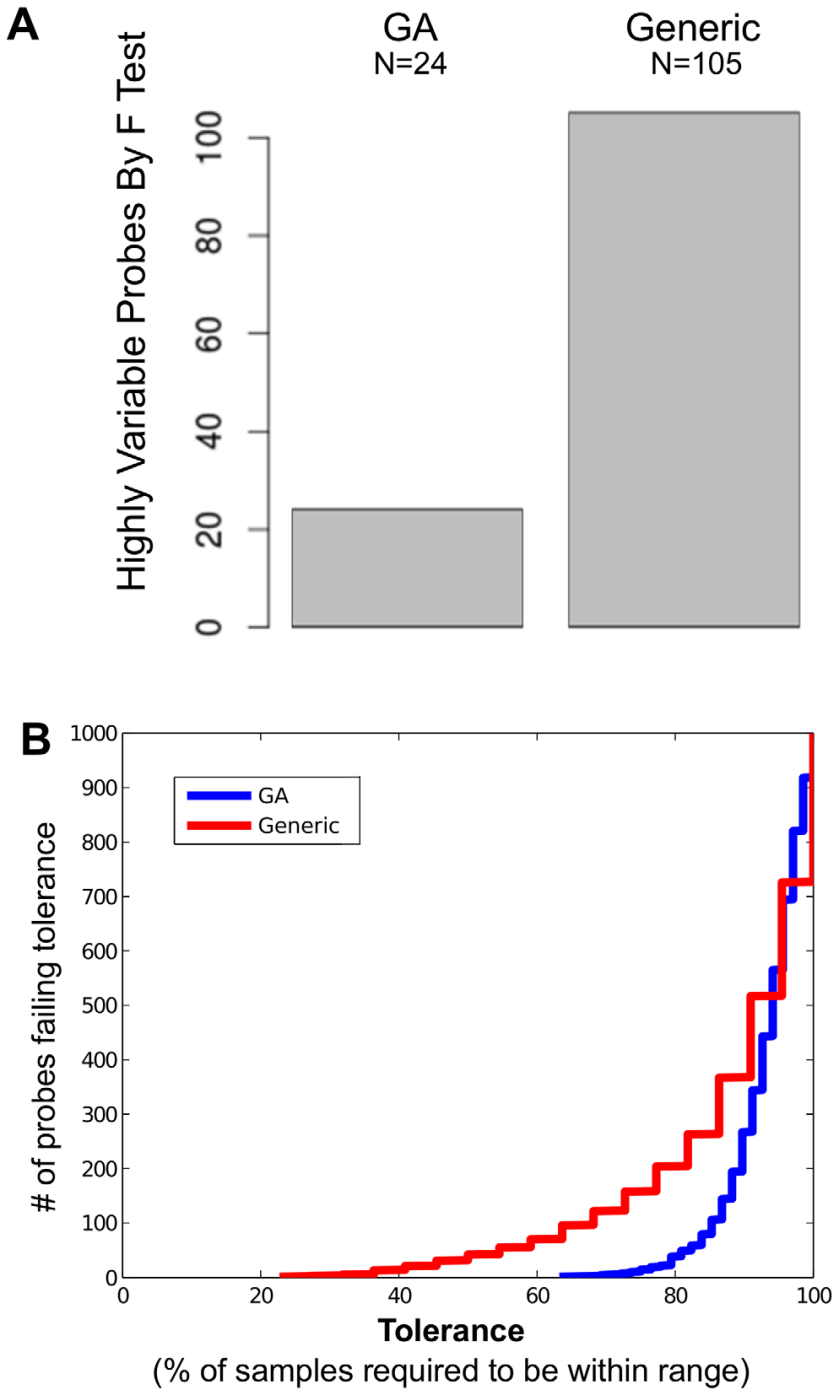
The biological impact of GA is significantly more consistent than that of generic. Among probes with variability induced by activation, 4-fold more probes had significant variation by F-test across 11 generic-activated samples from 5 batches, when compared to the number of probes with significant variation by F-test across 34 GA-activated samples from 30 batches (A). Defining tolerance as the percentage of samples with expression levels falling within the range between the maximum and minimum expression levels induced by reference standard for that probe, for any given tolerance threshold the number of probes failing to meet this this threshold is displayed for both generic and GA (B), showing that in almost all cases more probes fail to meet tolerance following induction by generic.

In comparing medicines produced by different manufacturing processes, it is important to assess if they are equally consistent in their biological impacts. We sought to examine differences in global variability across all relevant probes in order to address the question of whether the biological impact of generic was as consistent (across 5 batches) as GA (across 30 batches). Defining relevant probes as those with variability induced specifically by activation (as opposed to experimental noise such as the variability seen in samples exposed only to medium), we found that 4-fold more probes had significantly higher variability across the generic batches than across the GA batches (**Figure 1A** **and Table S3**). To ensure the robustness of the result, we randomly selected 11 samples from GA to match the 11 samples from generic, merging technical replicates to eliminate technical variability. We conducted a sensitivity analysis by repeating this process 10 times. Even by this strict method, the number of probes with greater variability in generic than GA was still significantly higher than the number of probes with greater variability in GA than generic (p<0.00089 by paired t-test, **Figure S1B**), consistent with the findings above.

As a second method of examining variability, we determined for each probe an acceptable range (i.e. between the minimum and maximum expression induced by GA reference standard). We determined the percentage of samples within this acceptable range across 34 samples including 30 batches of GA. We then determined the percentage of samples within this acceptable range across 11 samples including 5 batches of generic. We defined the maximum allowed percentage of samples with this range as the tolerance threshold, and found that for any given tolerance threshold generic almost always has more probes out of tolerance than GA (**Figure 1B**). For instance, 158 probes for generic fail to meet a tolerance of 75% of samples within the range defined by the reference standard, while only 10 probes for GA fail to meet the same tolerance. The very worst generic probe has 22.7% of samples within tolerance, while the very worst GA probe has 63% of samples within tolerance.

Finally, we examined the coefficient of variation (CV) across all probes in GA and in generic as a function of intensity, and found that there was a much narrower range of CV values present in GA than in generic at any given intensity (**Figure S1C**). We also examined the difference of CV(GA)-CV(generic) (**Figure S1D**), and observed more probes with highly negative values than highly positive values, consistent with the presence of greater variation in the generic.

It is important not only to identify differences in variability, but also to explore the potential biological impact of these differences. Thus, we calculated for each probe the ratio of the variance in generic to the variance in GA. The highest ranked probe by variability in generic relative to its variability in GA was for *FOXP3* (ILMN_2635132, ratio 4.17, **Table S4**), the key marker of tolerance-inducing regulatory T cells (Tregs). The probe with the second highest ratio of variance in generic to variance in GA was for *GPR83* (ILMN_2707941, ratio 4.14), which is also an established Treg marker [33].

### Comparing Impact on Key Immune System Genes: GA Induces Treg Markers FoxP3 and Gpr83 More Effectively than Generic

**Figure 2:**
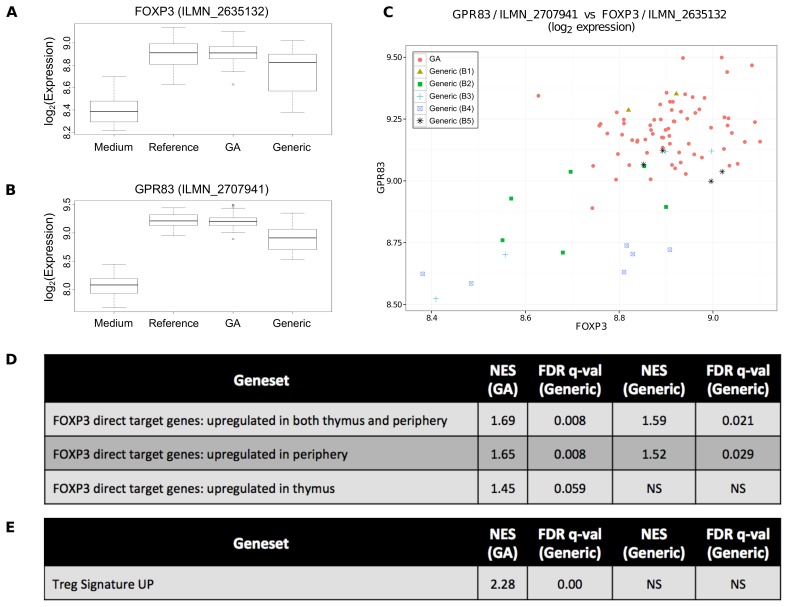
GA induces Tregs more effectively than generic. (A) GA induces significantly higher expression of FoxP3 than generic. FoxP3 is a key marker of Tregs, and (B) another key Treg marker Gpr83 shows a similar pattern of expression. (C) Both FoxP3 and Gpr83 are low in the same samples as indicated by scatter plot, further strengthening the case that generic fails to induce a strong Treg response in some patients. (D) As further evidence of the difference in FoxP3 induction, GSEA analysis found a significantly stronger upregulation of FoxP3 target genes in GA-activated samples than in generic-activated samples. (E) GSEA analysis also found a significant enrichment of Treg-specific genes among the genes with higher expression in GA than in generic. *NS = not significant.*

To systematically examine the differential expression of a particular gene in response to different medicines, we applied multiple methods including both parametric and non-parametric testing. Not only did GA induce *FOXP3* expression more consistently than generic, but GA also induced significantly higher expression as determined by 4 parametric methods: ANOVA (adjusted p<1.37×10^−3^), LIMMA with background subtraction (adjusted p<6.14×10^−4^), comparative marker selection using signal-to-noise (adjusted p<1.34×10^−2^ and t-test (adjusted p<2.12×10^−2^), and a non-parametric Wilcoxon rank-sum test (adjusted p<4.62×10^−2^ (**Figure 2A** **, Table S5**).

Applying the same methods to *GPR83*, we found that GA induced significantly higher levels of expression than generic: ANOVA (adjusted p<4.75×10^−8^), LIMMA with background subtraction (adjusted p<8.67×10^−10^), comparative marker selection using signal-to-noise (adjusted p<1.34×10^−2^) and t-test (adjusted p<1.49×10^−2^), and a non-parametric Wilcoxon rank-sum test (adjusted p<3.45×10^−4^, **Figure 2B**). *GPR83* is also in the top 20 probes by fold change from GA compared to generic (**Table S5**).

Because both *FOXP3* and *GPR83* are associated with Tregs [33] and both have more consistent and significantly higher expression induced by GA than by generic, we sought to determine on a per-sample basis whether the same subset of generic samples induced low levels of both genes, or whether *FOXP3* was low in some generic samples and *GPR83* was low in other generic samples. We confirmed that the same generic samples that were low in *FOXP3* were also low in *GPR83* (**Figure 2C**).

When the genes differentially expressed in response to different medicines are also transcription factors (e.g. *FOXP3*), we can further test the observation by examining the expression of genes known to be targets of that transcription factor. In this case, we sought to determine whether genes downstream of FoxP3 are upregulated following activation by GA as compared to generic. Through Gene Set Enrichment Analysis (GSEA), [30] we found that FoxP3 targets genes were enriched in GA samples compared to medium (FDR-adjusted q  = 0.008) to a more significant degree than in generic samples compared to medium (FDR-adjusted q  = 0.036, **Figure 2D** and **Figure S2A**).

For the list of genes with significantly higher expression induced by GA than generic by the non-parametric Wilcoxon rank-sum test, we utilized the Molecular Signature Database (MSigDB) [30] to determine that the same list of genes bound by FoxP3 was significantly enriched (adjusted p<1.59×10^−8^, **Table S6**).

### Comparing Potential Efficacy-related Impacts on Key Immune System Cell Types: GA Induces Tregs More Effectively than Generic

We wanted to systematically determine how two different medicines differentially impact immune system cells, using gene expression data. We first utilized an ANOVA-based pattern analysis method to identify a list of genes that are significantly downregulated only by generic compared to medium, and not by GA or GA reference standard compared to medium (**Table S7, Methods**). We then tested this list for immune system cell type enrichment via a novel approach (**Methods**). The genes downregulated only by generic are enriched in genes specific for a variety of T cells including Tregs (**Table S8**). From this same approach, genes significantly upregulated by GA and GA reference standard relative to medium, but not by generic relative to medium (**Table S7**) again yielded T cells, including Tregs, as the most enriched cell types (**Table S8**). Finally, genes that have significantly higher expression in samples activated by GA than generic as determined by the 4 parametric methods (**Table S5**) were, again, enriched for T cells, including Tregs (**Table S8**).

To further examine the impact of each medicine on specific immune system cells, we generated a list of genes with high cell-type specificity for Tregs, and utilized GSEA to determine the extent to which these Treg-specific genes were overrepresented at the top of the ranked list of genes differentially expressed between the two medicines. We confirmed that these Treg-specific genes were overrepresented at the top of the ranked list with higher expression in GA-activated samples than generic-activated samples (FDR-adjusted q  = 0.00, **Figure 2E** **and Figure S2B**).

Taken together, these findings emphasize that GA upregulates FoxP3+ Tregs more consistently and to a higher level than generic. This finding has implications for efficacy (**Discussion**).

### Comparing Potential Safety-related Impacts on Key Immune System Cell Types: Generic may Upregulate Myeloid Lineage Cells to a Greater Extent than GA

Using the ANOVA-based pattern analysis (**Methods**)**,** we identified a list of genes that are significantly upregulated only by generic compared to medium, and not by GA or GA reference standard compared to medium (**Table S7**). Cell type enrichment yielded a variety of stromal cells, macrophages, and monocytes (**Table S8**). Similarly, genes that were significantly downregulated by GA and GA reference standard relative to medium, but not by generic relative to medium (**Table S7**) were most enriched for macrophages, monocytes, and granulocytes (**Table S8**). Finally, genes that have significantly higher expression in samples activated by generic than in samples activated by GA by 4 different parametric methods (**Table S5**) were enriched primarily in macrophages and monocytes (**Table S8**).

**Figure 3:**
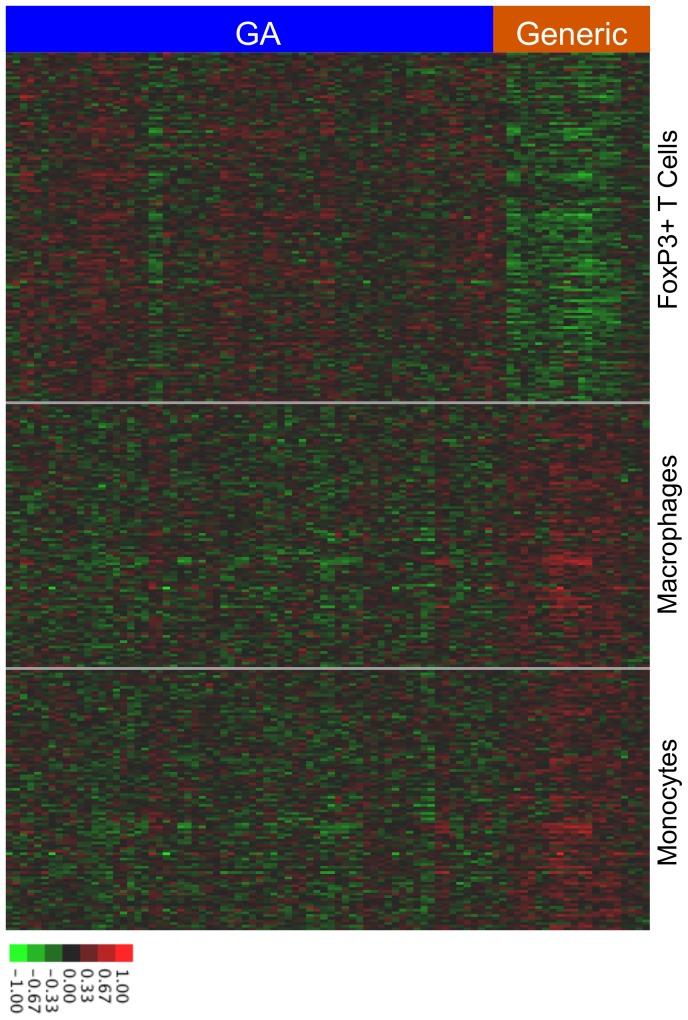
Cell-type specific differences in the biological impact of GA and generic. The heat map depicts relative expression of specific genes in GA-activated samples and generic activated samples. Each of the rows within the Treg section represents a gene with a high cell-type specificity scores for Tregs, while each of the rows in the macrophages and monocyte sections represents genes with high cell-type specificity scores for each of those cell types. The associated gene lists appear as supplementary information. Overall, GA induces higher expression of Treg-associated genes than generic, while generic induces higher expression of macrophage and monocyte-associated genes than GA.

To illustrate the cell-type specificity among the genes differentially expressed between GA and generic, we created a heat map of the differentially expressed genes showing the relative expression of Treg-specific genes, macrophage-specific genes, and monocyte-specific genes in samples activated by GA compared to samples activated by generic (**Figure 3** and **Table S9**). Consistent with our findings, generic appears to upregulate macrophage and monocyte-related genes while GA appears to upregulate T cell related genes including Tregs.

**Figure 4:**
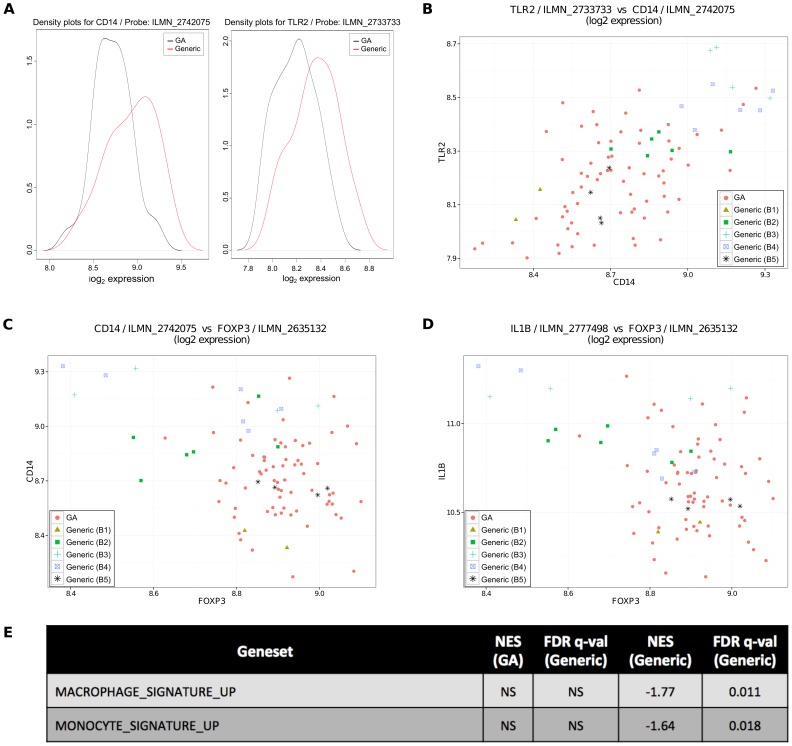
The generic’s impact on monocytes may differ from GA’s impact. (A) generic induces significantly higher expression of CD14 and TLR2, as determined by a Wilcoxon rank sum test and depicted as kernel density plots, which can be likened to a smoothed histogram. (B) CD14 and TLR2 expression are both unusually high in the same (mostly generic) samples. (C) FoxP3 expression is unusually low in the sample samples in which CD14 expression is unusually high, suggesting that the generic’s different impact on monocytes may be related to its different impact on Tregs and consistent with literature suggesting that monocytes play a role in GA-induced FoxP3 expression. (D) FoxP3 expression is unusually low in the sample samples in which IL1B expression is unusually high, suggesting that the generic’s different impact on monocytes may be related to the differences between LPS-activated monocytes and T-cell contact activated monocytes, which have been described in the literature as having opposite impacts on IL1B production. (E) GSEA analysis found a significant enrichment of monocyte and macrophage-specific genes among the genes with higher expression in generic than GA. *NS = not significant.*

To further investigate discrepant cell type activation between the GA and generic, we utilized the non-parametric Wilcoxon rank-sum test to determine which genes had significantly higher expression from generic than from GA, and performed an enrichment using MSigDB (**Methods**). The TLR signaling pathway was significantly enriched (adjusted p<1.27×10^−6^, **Table S6)**. Among the overlap genes significant by Wilcoxon and present in this pathway were *CD14* (adjusted p<4.77×10^−2^), a monocyte marker, and *TLR2* (adjusted p<3.65×10^−2^). Kernel density plots (**Figure 4A**), which can be likened to a smoothed histogram and effectively illustrate differences identified by non-parametric tests such as the Wilcoxon, show the differences in expression between generic and GA for these two genes. The boxplots for these two genes can be found in **Figure S3**.

Hypothesizing that both *CD14* and *TLR2* are associated with the same cell type (monocytes), we confirmed that the same generic samples had unusually high expression of both *CD14* and *TLR2* (**Figure 4B**).

### Investigating the Mechanisms Underlying Observed Differences: Why Does Generic Upregulate Tregs Less Effectively than GA?

Because monocytes may play a role in the mechanisms by which GA induces Tregs, [34] we sought to compare the expression of *FOXP3* and *CD14* in individual samples. Generic samples with low *FOXP3* also have high *CD14* (**Figure 4C**). This suggested that the differential impact on monocytes may be one mechanism by which GA and generic differentially impact Tregs.

Another mechanism by which GA and generic may differentially impact Tregs involves interferon gamma, which is known to induce *FOXP3* expression [35] and to be necessary for GA-induced *FOXP3* expression. [36] *IFNG* is upregulated dramatically by GA compared to generic: probes for *IFNG* are the #1 and #3 ranked probes by fold change for higher expression from GA (**Table S5** and **Figure S4**). Indeed, those generic samples with unusually low in *FOXP3* are also unusually low in *IFNG* (**Figure S4**).

### Investigating the Mechanisms Underlying Observed Differences: Why Does Generic Upregulate Monocytes?

GA is known to reduce *CD40* expression levels on monocytes, [34] which is consistent with our observation that *CD40* is among the list of genes with significantly lower expression (Wilcoxon, **Methods**) following activation by GA than following activation by generic (**Figure S5**). GA has a different impact on monocytes stimulated by T cell contact versus LPS: in the former case GA causes a decrease in monocyte *IL1B* production while in the latter case GA causes its increase. [37] This was notable because performing an MSigDB enrichment (**Methods**) on genes with higher expression from generic (Wilcoxon, **Methods**) also yielded significant enrichment in an LPS response pathway (adjusted p<4.96×10^−6^, **Table S6**). Moreover, we show that those generic samples with low levels of *FOXP3* also have high levels of *IL1B* (**Figure 4D**). GSEA analysis indicated that genes specific to monocytes and macrophages were significantly enriched among those genes with higher expression in generic than GA (**Figure 4E**). *IL1B* also appears to be associated with monocytes, as it is highly correlated with *CD14* (**Figure S6**). Finally, *IL1B* levels are significantly higher in generic than GA both by ANOVA (adjusted p<0.043) and LIMMA with background subtraction (adjusted p<0.037) **(Table S5**).

In order to determine if GA and generic were influencing different subtypes of monocytes, we performed a GSEA analysis using gene lists from literature examining human CD16^+^ and CD16^dim^ monocytes. [38] We found that among the genes upregulated by generic relative to medium, there was a significant enrichment in CD16^dim^ monocytes (FDR q  = 0.132, where the significance threshold is 0.25). Among the genes upregulated by GA relative to medium, there was a significant enrichment in CD16^+^ monocytes (FDR q  = 0.052, where the significance threshold is 0.25)(**Figure S7**).

## Discussion

We have developed a set of computational methods for comparing the immunological impact of a branded medicine with that of a generic.

The first set of methods involves comparing the variability of samples in expression of certain genes. We applied a broad range of computational methods including a variance ratio analysis that identifies specific genes for which one medicine is very consistent and the other is highly variable. We compared variability for individual genes directly using an F-test, plotted the coefficient of variation as a function of intensity to determine the relationship between variability and probe intensity, and investigated variability across batches. We also developed a new method using principles from chemical/process engineering to determine variability using acceptable ranges defined by a reference standard.

These methods produced multiple lines of evidence suggesting that generic has a significantly more variable biological impact than either GA reference standard or GA. For instance, 34 samples representing 30 different GA batches were found to be highly consistent and similar to GA reference standard. In contrast, more probes have higher variability in expression following stimulation with 11 samples representing 5 different generic batches. This variability itself is cause for concern among physicians and regulators, since the batch-to-batch variability of generic could manifest itself in ways that are harmful to patients. One possibility is that a patient could experience benefit from a particular batch of generic but not from a subsequent batch, preventing the patient from achieving the maximum benefit possible. Another, more disconcerting, possibility is that the variability could lead to a particular batch of generic causing adverse events. Due to the generic’s heterogeneity, such adverse events could be intermittent and therefore difficult to detect, monitor, and report.

The next set of methods involved identifying immunological impacts that differ between two medicines. We identified differentially expressed genes using a variety of methods (multiple parametric tests, non-parametric tests, and an ANOVA-based pattern matching method). We then explored the immunological relevance of these differentially expressed genes using a newly developed method for determining enrichment in genes specific to particular immune cell types. We further investigated the resulting hypotheses using established methods including hypergeometric enrichment with MSigDB [30] and GSEA [30] on lists of cell-type specific genes and transcription-factor target genes.

These methods identified specific genes and immune cell types that are upregulated significantly more by the GA than by generic. In this case, there is a preponderance of evidence suggesting that GA upregulates FoxP3+ Tregs more consistently and more effectively than generic. We have shown that the expression of FoxP3 itself, genes downstream of FoxP3, other known Treg markers, and Treg specific genes are all enriched from activation by GA relative to generic. This dramatic difference in biological impact on Tregs is certainly of note to physicians and regulators. It is well established that FoxP3+ Tregs induce beneficial tolerance in MS patients by suppressing harmful myelin reactive T cells, [39] so the more variable and reduced Treg induction raises questions about the potential efficacy of generic especially given recent findings demonstrating Copaxone’s impact on Tregs [36] and linking Tregs to clinical response in MS patients [40].

These methods also identified specific genes and immune cell types that are upregulated significantly more by generic than by GA. In this case, generic had a significantly higher impact on cells of the myeloid lineage such as monocytes and macrophages than GA did. Genes with significantly higher expression in generic than in GA include key monocyte markers such as CD14, enrich to macrophage and monocyte cell types, and are enriched in related pathways such as TLR signaling. The stronger upregulation of monocyte-specific genes warrants further investigation by physicians and regulators, especially given that monocytes are “prominent contributors” to neuroinflammation in MS [41] and given recent reports that one of GA’s mechanisms of action involves its impact on monocytes [37], [12].

The potential safety concerns stemming from the generic’s impact on monocytes were heightened by our GSEA analysis finding that the gene expression patterns following activation by generic more closely resemble the gene expression patterns of CD16^dim^ monocytes, while the expression patterns following GA activation more closely resemble the gene expression patterns associated with CD16^+^ monocytes. This is consistent with literature reports showing that Copaxone positively impacts CD16^+^ monocytes, [42] and is particularly concerning from a safety perspective because the CD16^dim^ monocytes favored by generic are known to play a different biological role.

The difference in impact on monocytes could also help explain the observed differences in Treg upregulation, since GA-treated monocytes are known to upregulate FoxP3 expression. [34] GA had an opposite impact on monocytes stimulated by LPS (resulting in increased IL1B production) as opposed to monocytes stimulated by T cell contact (resulting in decreased IL1B production). [37] The same generic samples that have unusually high levels of IL1B also have unusually low FoxP3. generic also shows upregulation in LPS response pathways. Together, these findings suggest that some component of generic, either deliberate or due to contamination, may trigger an LPS response pathway in monocytes leading to excessive IL1B production and unusually low induction of FoxP3+ Tregs. This possibility warrants further investigation with regard to safety.

One clear caveat to any gene expression study in mice lies in the inherent differences between healthy mouse models and human MS patients. Yet, there are clear differences in biological impact of GA and generic. One step to further address this lies in linking the differentially impact genes to markers and processes known to be linked to Copaxone response in humans with MS. [43], [44] Our study design sought to model the situation in which a patient is initially treated with GA, and later switched to a generic. A variety of other experimental designs could be explored in future studies, including priming *in vivo* with generic and reactivating with generic, then comparing the resulting transcription profiles to those that result from priming in vivo with GA and reactivating with GA. Such studies may demonstrate even more dramatic differences between GA and generic. Given the batch-to-batch variability that we identified in the generic, such studies should explore priming and reactivating with the same batch of generic, as well as priming and reactivating with different batches of generic. Further studies could also be conducted in human cell lines or PBMC, and could build upon the previously identified physiochemical differences between GA and generic [45] by assaying for mechanistically relevant immunological processes such as Treg activity, binding affinity to HLA class II molecules, Th1 to Th2 shift, and TCR modulation.

**Figure 5:**
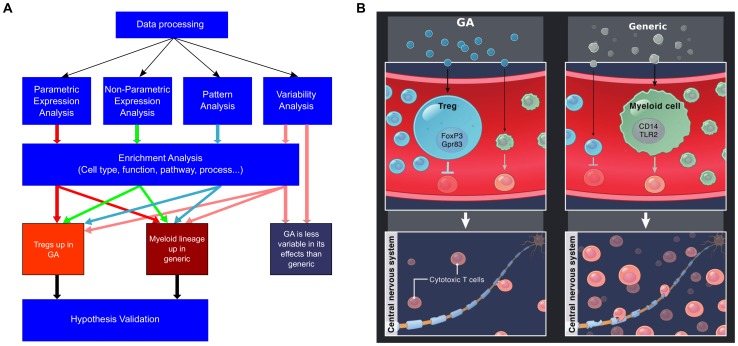
Flow chart of process for comparing a branded medicine to a generic, and model of key differences between GA and generic. (A) Overview of the methods for analyzing gene expression data to compare the immunological impact of GA to that of generic. After processing, direct differences are identified by multiple parametric methods, non-parametric methods, as well as ANOVA-based pattern analysis, and variability analysis. The genes identified by these methods are analyzed using a variety of enrichment-based methods, which result in hypotheses that are then verified through additional methods. (B) The key hypotheses emerging from our studies involve the greater heterogeneity in the generic’s biological impact compared to GA’s, and the fact that GA appears to more effectively upregulate FoxP3 expression and promote tolerance-inducing Tregs, while generic appears to upregulated myeloid lineage cells such as monocytes and macrophages which may impair tolerance. Given these findings, it is reasonable to hypothesize that GA may suppress harmful cytotoxic cells more effectively than generic, and this hypothesis warrants further investigation.

In these studies, we have sought to develop a broadly applicable set of computational methods for comparing branded medicines to generics (**Figure 5A**). We found higher variability in gene expression following activation by generic compared to GA, and the significant differences in impact on key biological processes including Tregs and monocytes (**Figure 5B**). These differences raise questions for physicians and regulators seeking safe and effective treatments for MS patients, and suggest that clinical studies are warranted, using appropriate safety and efficacy endpoints to compare generic to GA. More generally, the data analysis methods described here can be utilized in a variety of situations to compare the biological impacts of other branded and generic therapies, in order to ensure that patients receive the best possible medicines.

## Supporting Information

Figure S1 **Principal Component Analysis (PCA) was utilized to check for outliers, (A).** (B) Plot illustrating that the findings in Figure 1A still hold when the 11 generic samples are compared to 11 randomly selected GA samples. (C) Plot of the coefficient of variation (CV) as a function of intensity for each of the probes when activated by generic (black) and GA (red), showing the smaller range of CVs in GA and the wider range in generic at any given intensity. (D) Plot of the difference of CV(GA)-CV(generic) for each probe as a function of intensity, showing more probes with negative values indicating higher CV values in generic.
(TIFF)

Figure S2 **The GSEA enrichment plots for the FoxP3 and Treg GSEA analyses reported in** **Figure 2D–E** **.**
(TIFF)

Figure S3 **Box plots of CD14 and TLR2, depicting the lower expression levels in GA and Reference compared to generic.** This is an additional way of visualizing the differences depicted by kernel density plots in Figure 4A.
(TIFF)

Figure S4 **Scatter plots showing that the same generic samples with unusually low FoxP3 expression also had unusually low IFNG expression, by two different probes of IFNG.** Scatter plots illustrating that for two different probes of IFNG, GA and Reference standard upregulated IFNG to a greater extent than generic did.
(TIFF)

Figure S5 **Kernel density plot of CD40, illustrating the fact that this gene had higher expression in generic activated samples than in GA activated samples, consistent with the determination by the Wilcoxon rank-sum test and consistent with literature.**
(TIFF)

Figure S6 **Scatterplot illustrating the high degree of correlation between CD14 and IL1B, lending support to the hypothesis that the IL1B is expressed primarily by monocytes.**
(TIFF)

Figure S7 **GSEA analysis showing that genes with higher expression in generic than medium are enriched in genes specific to CD16dim monocytes, while genes with higher expression in GA than medium are enriched in genes specific to CD16+ monocytes.**
(TIFF)

Figure S8 **Illustration of the tolerance method for comparing variability.** The expression of genes following activation by GA and generic are assessed to determine the percentage of samples following within a tolerance defined by the maximum and minimum expression levels induced by the reference standard (top and bottom of the red box for Gpr83, left and right sides of the red box for FoxP3).
(TIFF)

Table S1 **Sample assignments on chip, illustrating randomization to avoid batch effects.**
(PDF)

Table S2 **Genes utilized for the tolerance method illustrated in** **Figure 1B** **.**
(PDF)

Table S3 **The highly variable probes that were significant by F-test in either GA or generic (see methods section) and are depicted in** **Figure 1A** **.**
(PDF)

Table S4 **Ranked list of probes by ratio of the variance in generic-activated samples to the variance in GA-activated samples.**
(PDF)

Table S5 **Comparison of expression in GA to expression in GA for each probe, including fold change, ANOVA, LIMMA with background subtraction, comparative marker selection by signal-to-noise ratio, comparative marker selection by t-test, and the Wilcoxon non-parametric method.**
(PDF)

Table S6 **MSigDB enrichment results for the list of genes with significantly different expression between GA and generic by the Wilcoxon rank sum test, including FoxP3 targets among the enriched signatures for genes higher in GA than generic, and TLR and LPS pathways among the enriched signatures for genes higher in generic than GA.**
(PDF)

Table S7 **Output of the ANOVA pattern matching method utilized to identify genes upregulated or downregulated only in generic or only in GA and reference standard.**
(PDF)

Table S8 **Outputs of cell-type enrichment analyses for various lists of genes (higher expression in GA than generic, higher expression in generic than GA, upregulated only by generic, downregulated only by generic, upregulated only by GA and reference standard, downregulated only by GA and reference standard).**
(PDF)

Table S9 **List of genes depicted in the heat map in** **Figure 3** **.**
(PDF)
