# Supplementary figures and images for: Comparing the Biological Impact of Glatiramer Acetate with the Biological Impact of a Generic
Source: PLoS One. 2014 Jan 8;9(1):e83757. doi: 10.1371/journal.pone.0083757 (PMC3885444; doi:10.1371/journal.pone.0083757)

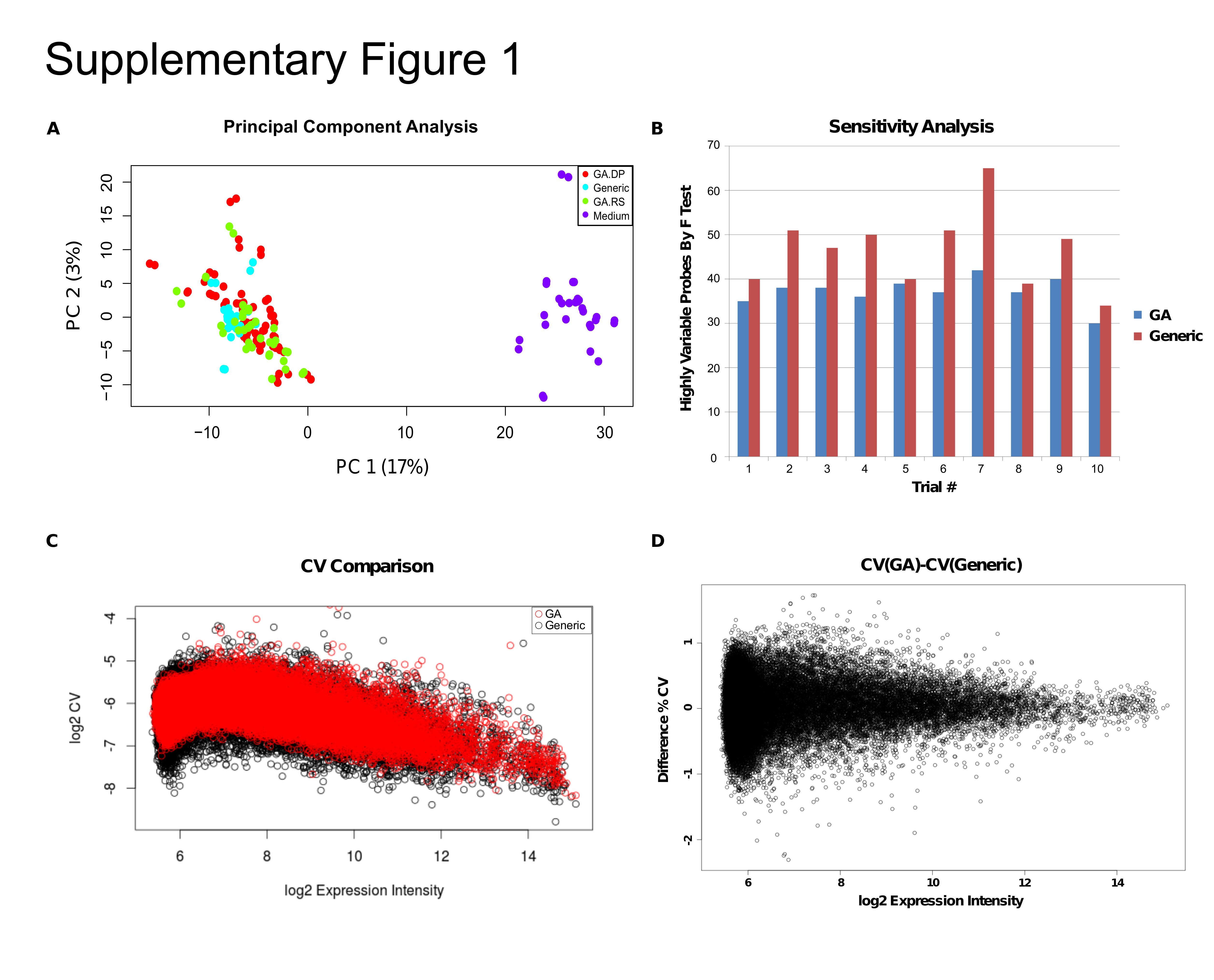

Supplement: Figure S1 — Principal Component Analysis (PCA) was utilized to check for outliers, (A). (B) Plot illustrating that the findings in Figure 1A still hold when the 11 generic samples are compared to 11 randomly selected GA samples. (C) Plot of the coefficient of variation (CV) as a function of intensity for each of the probes when activated by generic (black) and GA (red), showing the smaller range of CVs in GA and the wider range in generic at any given intensity. (D) Plot of the difference of CV(GA)-CV(generic) for each probe as a function of intensity, showing more probes with negative values indicating higher CV values in generic. (TIFF) [file pone.0083757.s001.tiff]

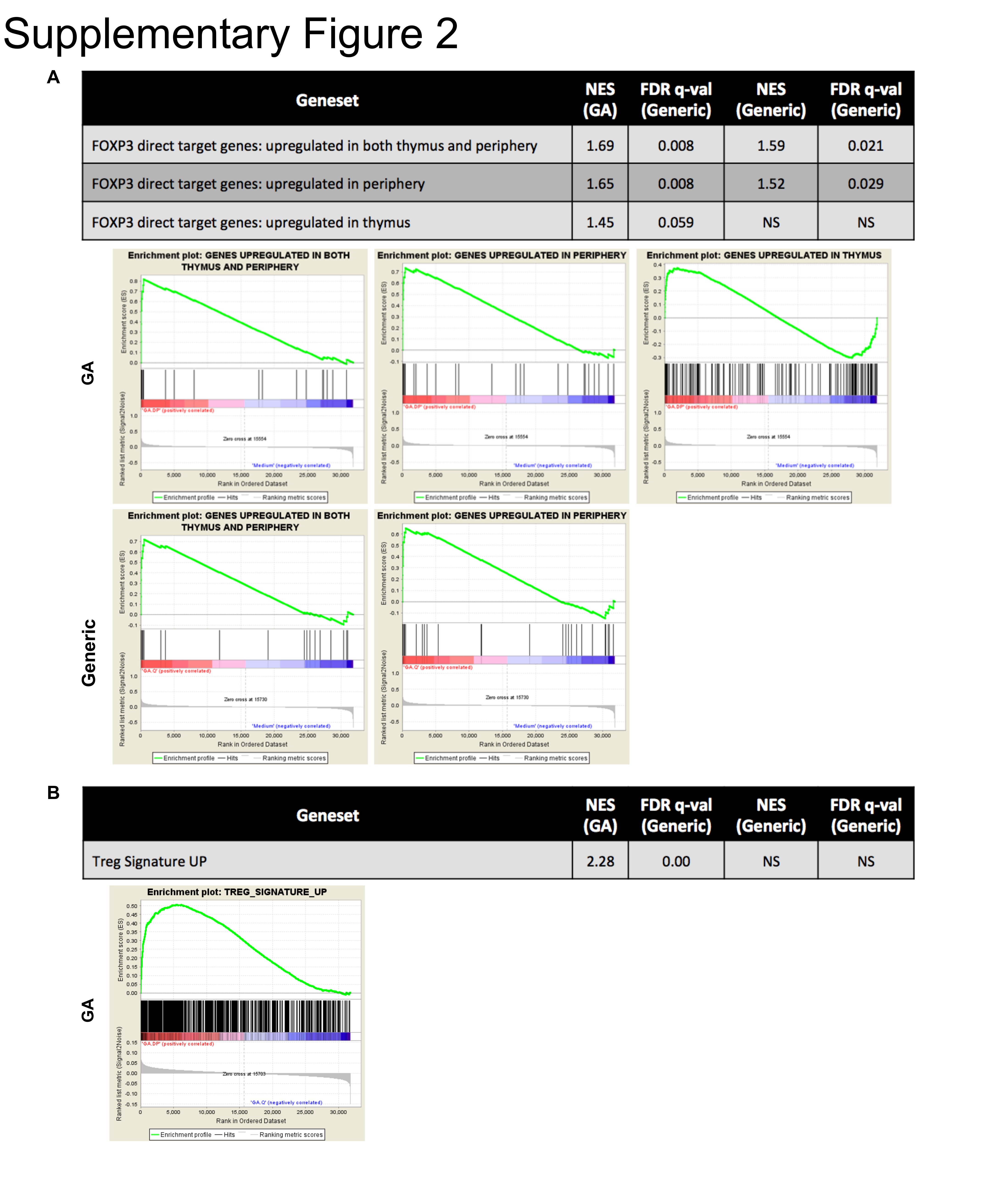

Supplement: Figure S2 — The GSEA enrichment plots for the FoxP3 and Treg GSEA analyses reported in Figure 2D–E . (TIFF) [file pone.0083757.s002.tiff]

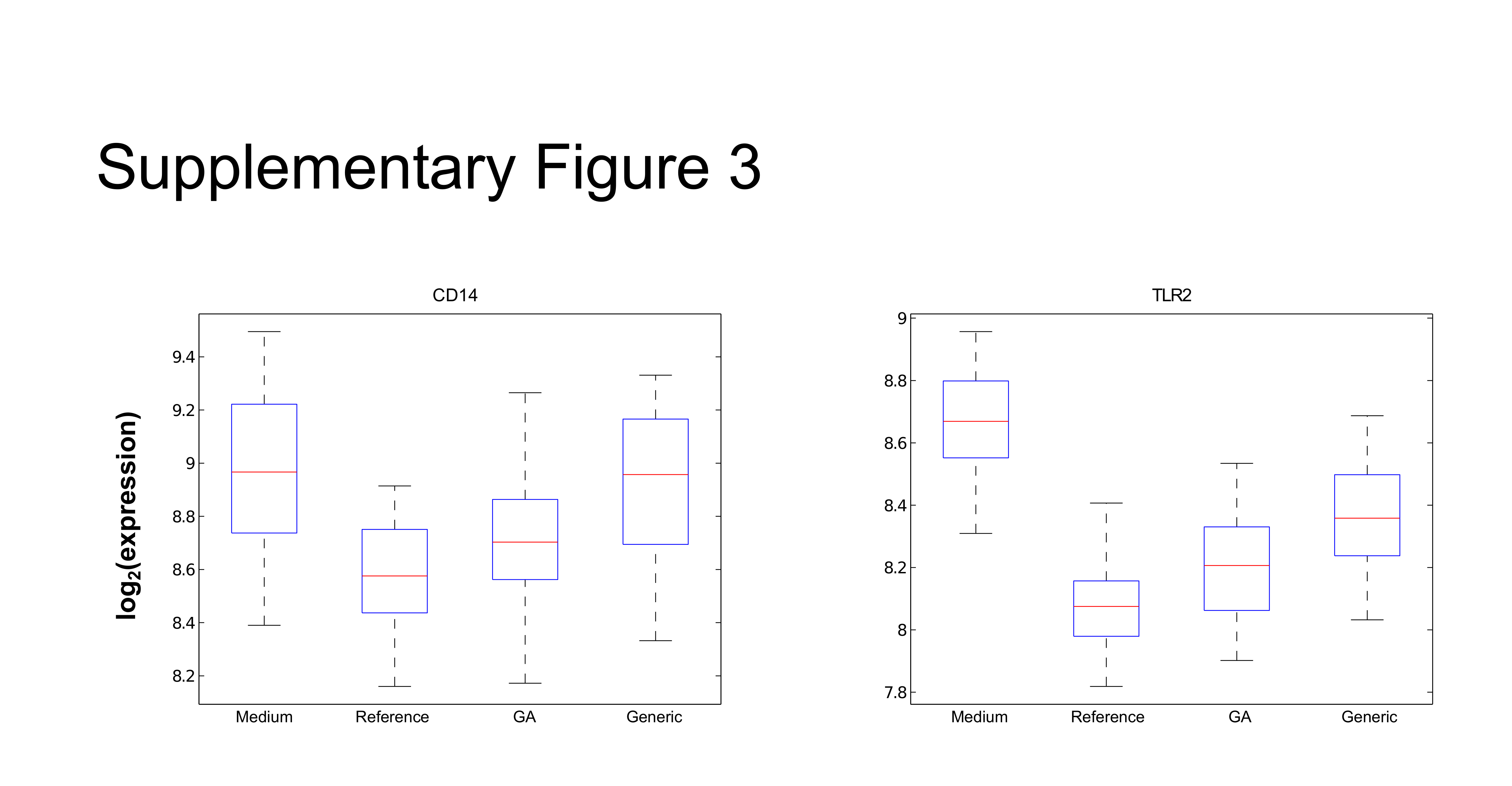

Supplement: Figure S3 — Box plots of CD14 and TLR2, depicting the lower expression levels in GA and Reference compared to generic. This is an additional way of visualizing the differences depicted by kernel density plots in Figure 4A. (TIFF) [file pone.0083757.s003.tiff]

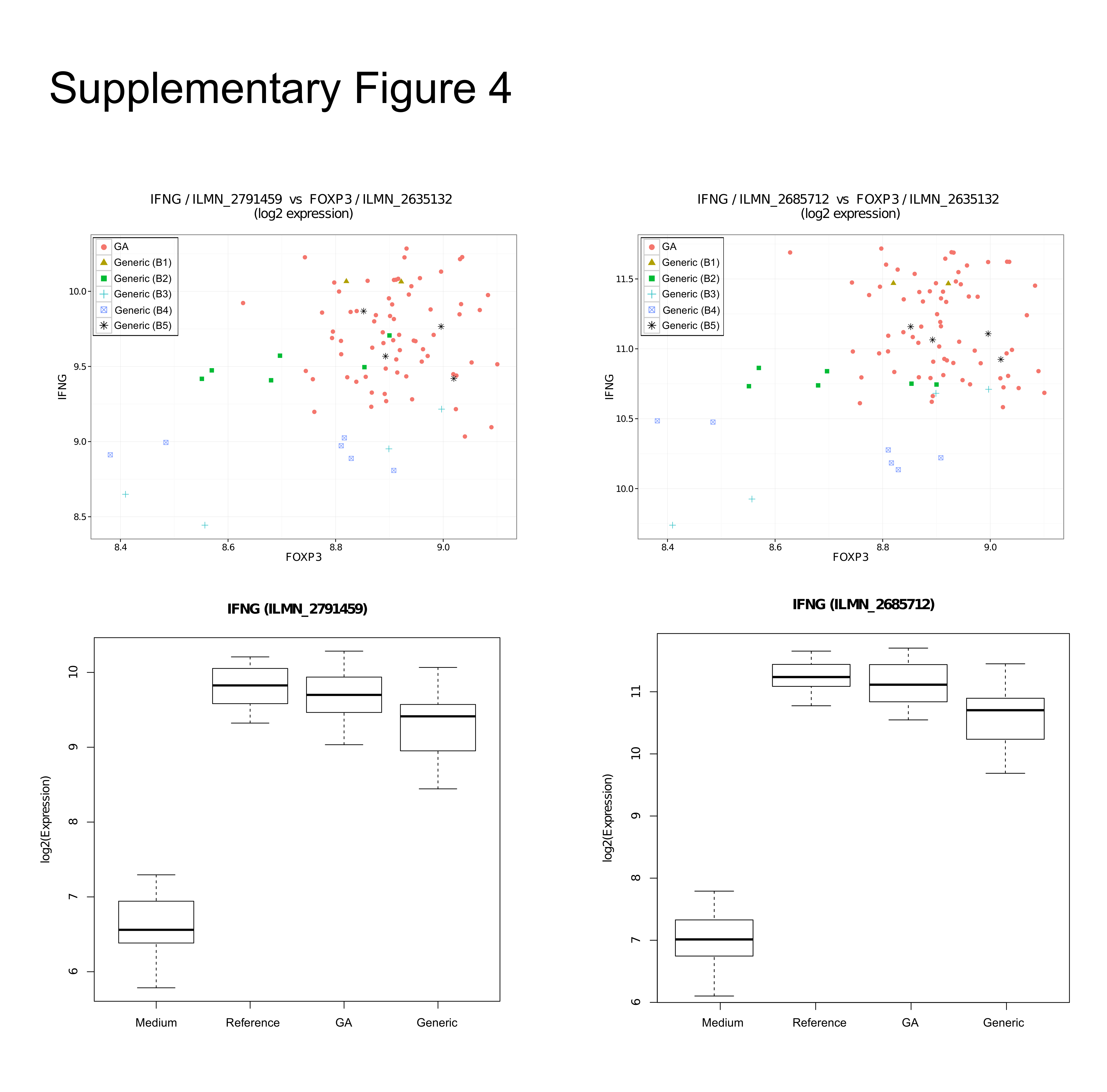

Supplement: Figure S4 — Scatter plots showing that the same generic samples with unusually low FoxP3 expression also had unusually low IFNG expression, by two different probes of IFNG. Scatter plots illustrating that for two different probes of IFNG, GA and Reference standard upregulated IFNG to a greater extent than generic did. (TIFF) [file pone.0083757.s004.tiff]

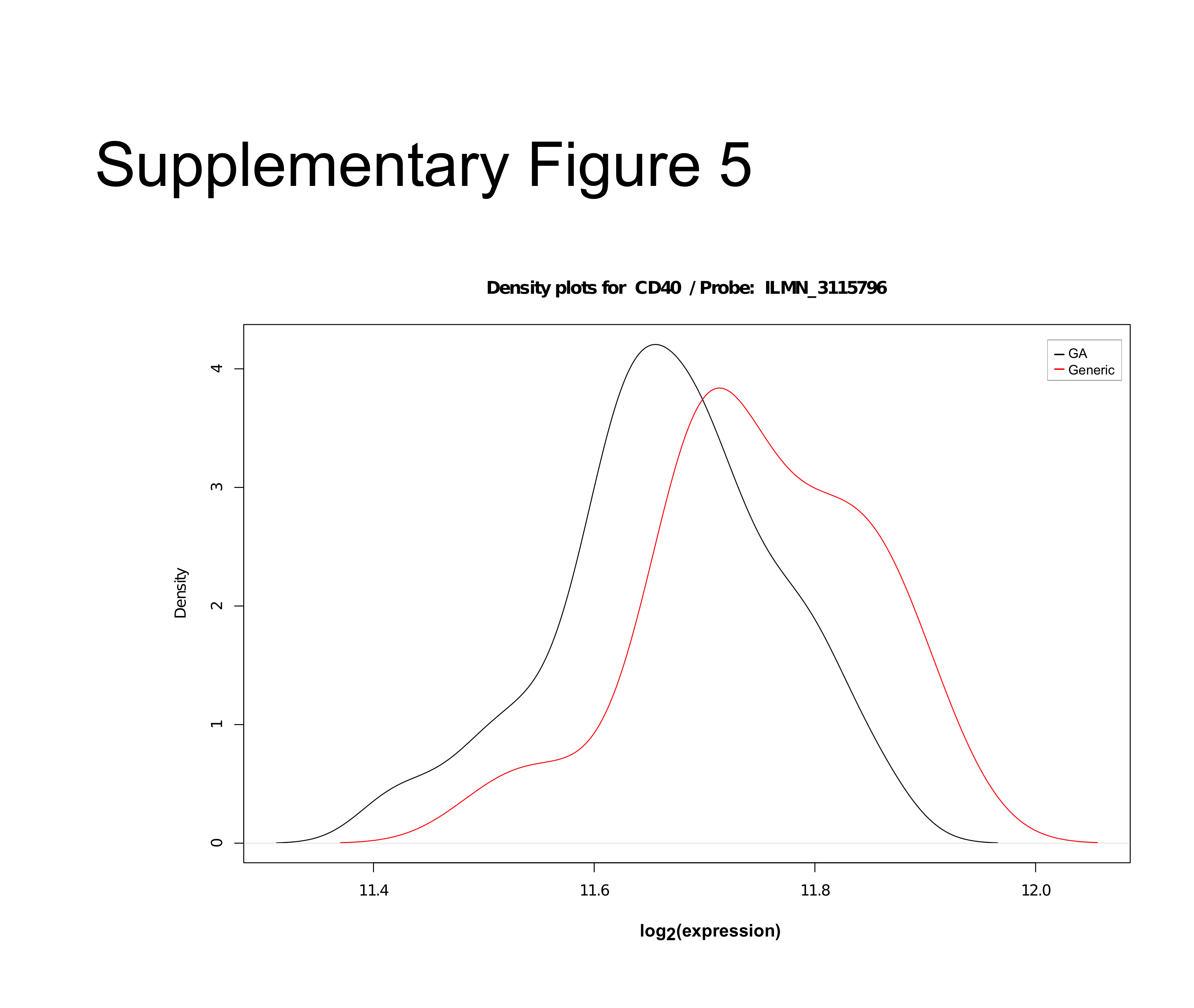

Supplement: Figure S5 — Kernel density plot of CD40, illustrating the fact that this gene had higher expression in generic activated samples than in GA activated samples, consistent with the determination by the Wilcoxon rank-sum test and consistent with literature. (TIFF) [file pone.0083757.s005.tiff]

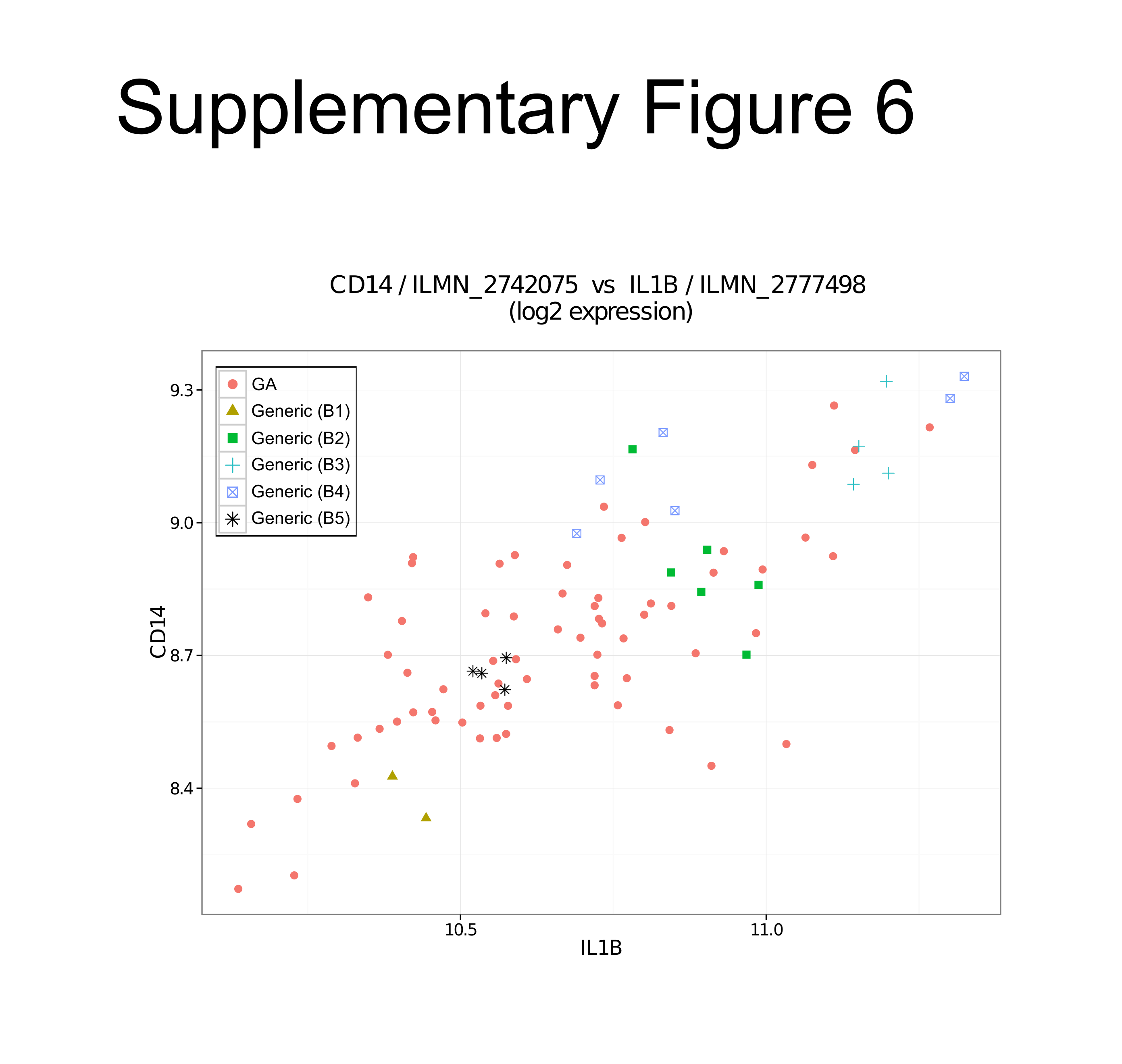

Supplement: Figure S6 — Scatterplot illustrating the high degree of correlation between CD14 and IL1B, lending support to the hypothesis that the IL1B is expressed primarily by monocytes. (TIFF) [file pone.0083757.s006.tiff]

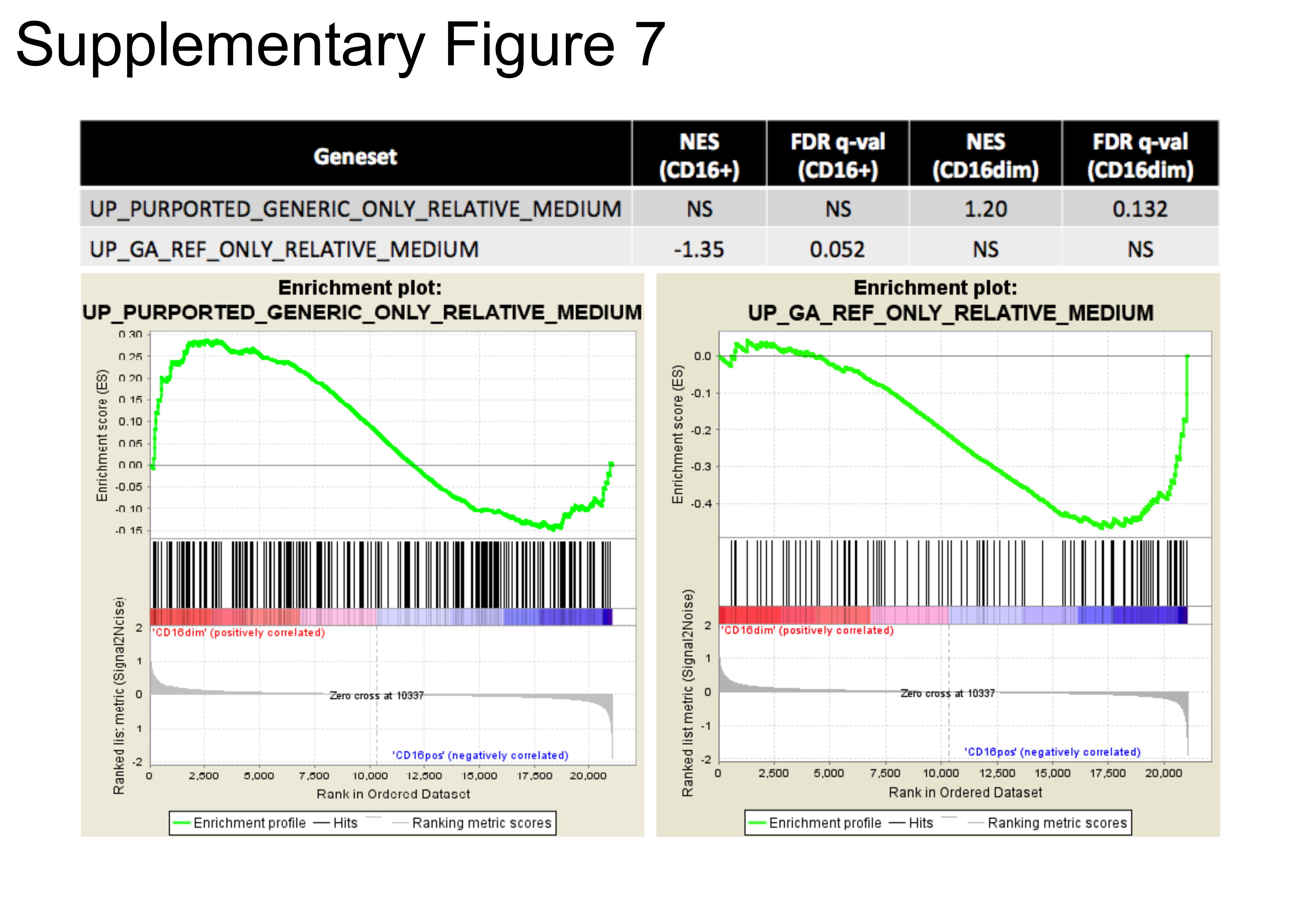

Supplement: Figure S7 — GSEA analysis showing that genes with higher expression in generic than medium are enriched in genes specific to CD16dim monocytes, while genes with higher expression in GA than medium are enriched in genes specific to CD16+ monocytes. (TIFF) [file pone.0083757.s007.tiff]

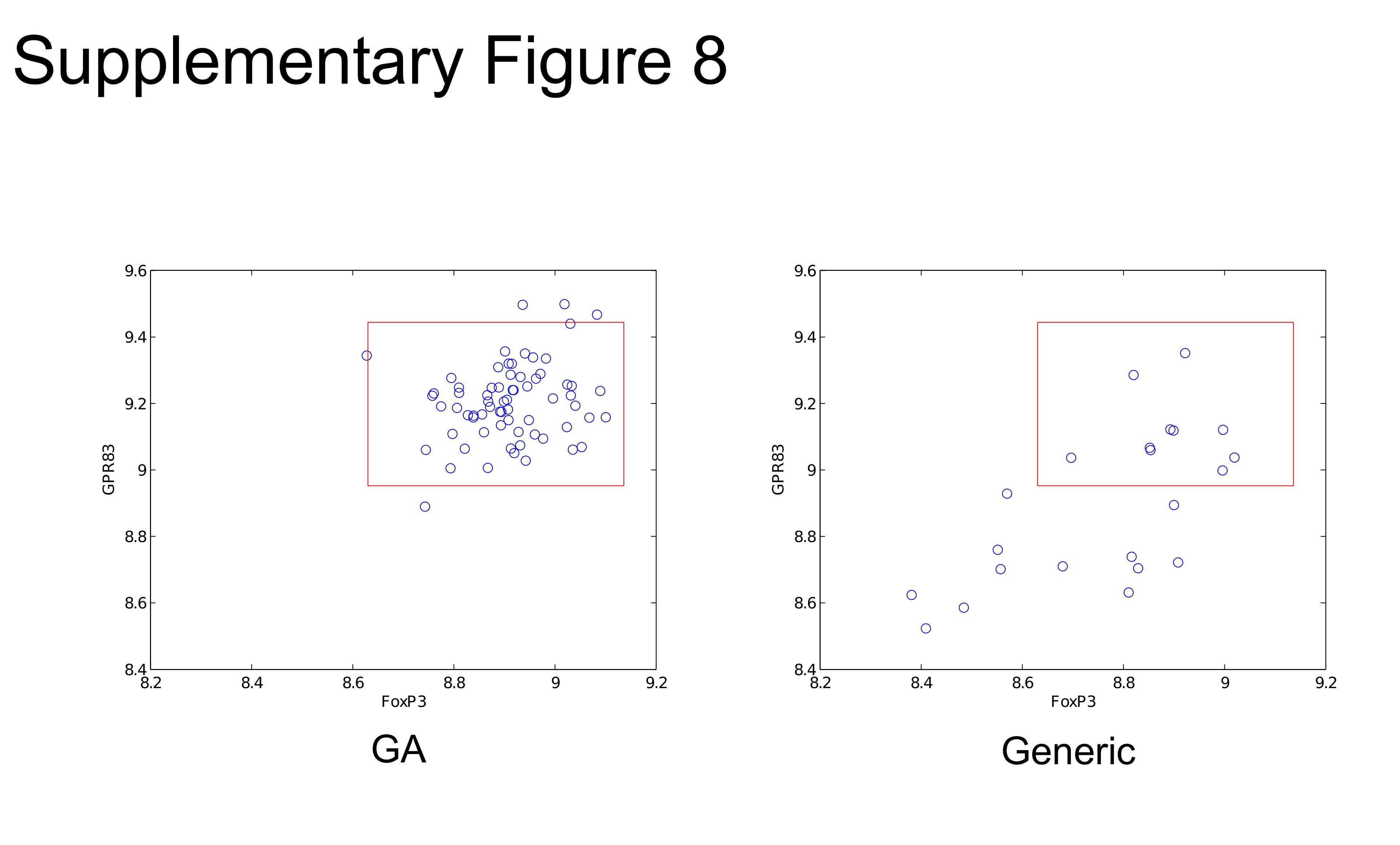

Supplement: Figure S8 — Illustration of the tolerance method for comparing variability. The expression of genes following activation by GA and generic are assessed to determine the percentage of samples following within a tolerance defined by the maximum and minimum expression levels induced by the reference standard (top and bottom of the red box for Gpr83, left and right sides of the red box for FoxP3). (TIFF) [file pone.0083757.s008.tiff]
