# Supplementary material for: Comparing the Biological Impact of Glatiramer Acetate with the Biological Impact of a Generic
Source: PLoS One. 2014 Jan 8;9(1):e83757. doi: 10.1371/journal.pone.0083757 (PMC3885444; doi:10.1371/journal.pone.0083757)
Supplement: Table S1 — Sample assignments on chip, illustrating randomization to avoid batch effects. (PDF) [file pone.0083757.s009.pdf]

TABLE S1			
№	Code	Chip #	Chip Location
1	RS-16	4634633002	A
2	DP-34	4634633002	B
3	DP-19	4634633002	C
4	DP-32	4634633002	D
5	DP-04	4634633002	E
6	MBP	4634633002	F
19	RS-17	4634633043	A
20	N1.1	4634633043	B
21	N5.1	4634633043	C
22	GA_Acid	4634633043	D
10	DM958	4634633043	E
23	Medium-01	4634633043	F
7	RS-01	4634633053	A
8	DP-31	4634633053	B
9	N3.1	4634633053	C
N/A	(failed QC)	4634633053	D
11	DM962.1	4634633053	E
12	Medium-15	4634633053	F
13	Manitol	4637105025	A
14	DP-20	4637105025	B
15	DP-16	4637105025	C
16	DP-15	4637105025	D
17	DP-30	4637105025	E
18	DP-11	4637105025	F
24	RS-02	4682416019	A
25	DP-13	4682416019	B
26	DP-21	4682416019	C
27	DP-18	4682416019	D
28	RS-03	4682416019	E
29	DP-17	4682416019	F
33	RS-18	4682416043	A
35	N3.2	4682416043	B
37	GA_Heat	4682416043	C
38	GA_Base	4682416043	D
39	RS-04	4682416043	E
40	DP-29	4682416043	F
41	DM961	4682416087	A
42	Medium-03	4682416087	B
43	RS-19	4682416087	C
45	TV-35	4682416087	D
46	TV-66	4682416087	E
47	TV109	4682416087	F
30	N4.1	4682416090	A
31	DP-14	4682416090	B
32	Medium-02	4682416090	C

34	DP-27	4682416090	D
36	DP-26	4682416090	E
44	DP-28	4682416090	F
72	RS-10	4763128059	A
73	N2.2	4763128059	B
74	DP-06	4763128059	C
75	DM962.3	4763128059	D
76	DP-07	4763128059	E
77	Medium-09	4763128059	F
78	RS-11	4763128060	A
79	N2.1	4763128060	B
80	DP-09	4763128060	C
81	DM962.2	4763128060	D
82	DP-05	4763128060	E
83	Medium-10	4763128060	F
48	RS-05	4763646004	A
49	RS_h-1	4763646004	B
50	DP-01	4763646004	C
51	Medium-04	4763646004	D
52	RS-06	4763646004	E
53	RS_h-2	4763646004	F
60	RS-08	4763646012	A
61	N4.2	4763646012	B
62	DP-33	4763646012	C
63	C1.1	4763646012	D
64	N5.2	4763646012	E
65	Medium-07	4763646012	F
66	RS-09	4763646026	A
67	N4.3	4763646026	B
68	DP-08	4763646026	C
69	C1.2	4763646026	D
70	N5.3	4763646026	E
71	Medium-08	4763646026	F
54	DP-02	4763646030	A
55	Medium-05	4763646030	B
56	RS-07	4763646030	C
57	RS_h-3	4763646030	D
58	DP-03	4763646030	E
59	Medium-06	4763646030	F
84	RS-12	5216898004	A
85	Appo1.2	5216898004	B
86	DP-22	5216898004	C
87	Appo2.1	5216898004	D
88	DP-12	5216898004	E
89	Medium-11	5216898004	F
90	RS-13	5216898008	A
91	Shira.1	5216898008	B
92	DP-24	5216898008	C

93	TV5010.1	5216898008	D
94	DP-10	5216898008	E
95	Medium-12	5216898008	F
96	RS-14	5216898012	A
97	Appo1.1	5216898012	B
98	DP-23	5216898012	C
99	Appo2.2	5216898012	D
100	Edan.1	5216898012	E
101	Medium-13	5216898012	F
102	RS-15	5216898024	A
103	Shira.2	5216898024	B
104	DP-25	5216898024	C
105	TV5010.2	5216898024	D
106	Edan.2	5216898024	E
107	Medium-14	5216898024	F
RS = GA reference standard			
DP = Copaxone® GA drug product			
N = Glatimer® generic			
