# Supplementary material for: Comparing the Biological Impact of Glatiramer Acetate with the Biological Impact of a Generic
Source: PLoS One. 2014 Jan 8;9(1):e83757. doi: 10.1371/journal.pone.0083757 (PMC3885444; doi:10.1371/journal.pone.0083757)
Supplement: Table S2 — Genes utilized for the tolerance method illustrated in Figure 1B . (PDF) [file pone.0083757.s010.pdf]

TABLE S2					
ID	Gene	AVG Medium	AVG Reference Standard	AVG GA	AVG generic
ILMN_2685712	IFNG	7.151943333	11.25528947	11.16330882	10.66788182
ILMN_1247309	IL3	5.823283333	9.406355263	9.384125	9.421563636
ILMN_2791459	IFNG	6.636043333	9.829173684	9.710919118	9.303940909
ILMN_1215862	CXCL9	7.341613333	9.816544737	9.756617647	9.524722727
ILMN_2595732	LOC100046	9.24871	11.50916579	11.54660147	11.76695
ILMN_2931334	IL4	6.171636667	8.367486842	8.425076471	8.496068182
ILMN_2718330	CISH	9.18378	11.37349211	11.34956176	11.42528636
ILMN_2445789	TNFRSF9	6.794626667	8.943107895	8.962886765	8.867340909
ILMN_1235499	PROS1	6.8909	8.894226316	8.934763235	8.691490909
ILMN_1236588	CAR1	7.761993333	9.729336842	9.793017647	9.715413636
ILMN_2588216	IL8RA	8.623573333	6.726942105	6.682098529	6.572263636
ILMN_2680827	IL7R	10.26270333	8.408492105	8.445019118	8.336627273
ILMN_3103746	TNFRSF9	6.92238	8.764752632	8.744404412	8.737481818
ILMN_2606162	PDLIM4	7.95422	9.731202632	9.727291176	9.501227273
ILMN_1213954	SGK1	11.41363	9.661723684	9.666986765	9.421709091
ILMN_1216042	APOE	9.313533333	7.564486842	7.5268	7.541936364
ILMN_2507761	7530404M1	5.58119	7.321434211	7.426433824	7.179863636
ILMN_3142803	CXCL10	6.9917	8.700381579	8.707408824	8.518177273
ILMN_1249030	MPO	9.78357	8.174247368	8.410351471	8.711063636
ILMN_1214841	IL24	6.20973	7.810955263	7.762489706	7.504672727
ILMN_2744890	GADD45G	8.972466667	10.57273421	10.65257941	10.53536818
ILMN_2742861	SERPINA3F	8.155613333	9.724289474	9.630060294	9.550477273
ILMN_1217389	PKIB	9.656156667	11.20944737	11.18970735	11.08776818
ILMN_2749412	CSF2	6.444243333	7.991860526	7.939511765	7.916009091
ILMN_2426853	UBD	6.649816667	8.167139474	8.137648529	7.952622727
ILMN_2725927	SERPINA3G	12.45885667	13.95378947	13.90402647	13.98564545
ILMN_3094853	PKIB	7.606836667	9.100334211	9.029398529	9.009122727
ILMN_1220788	GM590	6.52345	8.002165789	8.021569118	7.660568182
ILMN_2780247	LTA	7.175613333	8.633663158	8.581783824	8.382890909
ILMN_2600421	MPO	8.83189	7.385586842	7.594448529	7.965109091
ILMN_2947526	ECM1	8.577696667	10.02243947	10.05155441	9.810595455
ILMN_2772264	IGH-VJ558	11.98722333	10.55665789	10.57623824	10.44005909
ILMN_1229804	LTA	6.55449	7.963042105	8.009302941	7.831009091
ILMN_2506039	A130090K0	9.575673333	8.169478947	8.136902941	7.924245455
ILMN_2762983	TBX21	7.060853333	8.465081579	8.480467647	8.371186364
ILMN_2756046	FFAR2	7.936103333	9.323384211	9.336789706	9.122636364
ILMN_2903945	GADD45G	11.30813	12.65795526	12.67639853	12.61029091
ILMN_2878071	LYZ	11.69561333	10.38226579	10.50047647	10.79755909
ILMN_2460094	WNT10A	8.443006667	7.148705263	7.098610294	7.211363636
ILMN_1252076	LYZ2	9.709593333	8.418768421	8.645485294	8.893840909
ILMN_1224336	SPSB1	7.674476667	8.962918421	8.950477941	8.84225
ILMN_2446559	TNFRSF4	9.380393333	10.65664474	10.65343824	10.55523636
ILMN_2717176	RGL1	8.882566667	7.607873684	7.561447059	7.5231
ILMN_2741201	IL17F	7.970356667	9.233218421	9.281625	9.150545455

ILMN_3090207	CYP4F18	9.138546667	7.882936842	7.951694118	7.8979
ILMN_2546272	GNG12	8.461113333	9.715955263	9.731457353	9.698468182
ILMN_2548681	4930426D0	8.374753333	7.133186842	7.100458824	7.092509091
ILMN_2664224	EPHX1	11.05266667	9.821181579	9.83665	9.763663636
ILMN_1235926	A630072M	7.662946667	8.890823684	8.780570588	8.808022727
ILMN_1227900	IL12RB1	6.848926667	8.074565789	8.071257353	8.030231818
ILMN_1259075	NME7	9.56043	8.337597368	8.301139706	8.096959091
ILMN_2770066	LOC100044	10.47755333	9.276686842	9.229413235	9.231372727
ILMN_1227434	ITGB7	11.00959333	9.814152632	9.885063235	9.683159091
ILMN_1233474	IL2RA	6.869136667	8.061205263	8.134330882	7.977345455
ILMN_2906430	ICOS	8.19493	9.379094737	9.444848529	9.41605
ILMN_1221819	FCGRT	9.812453333	8.634481579	8.525132353	8.270463636
ILMN_2767615	ATP1B1	8.774563333	7.597357895	7.592442647	7.479663636
ILMN_2707941	GPR83	8.052503333	9.212121053	9.204789706	8.896509091
ILMN_1233987	1110014O2	8.7487	9.906276316	9.906113235	9.809395455
ILMN_2758720	SYPL	10.40974667	11.56390526	11.58090735	11.63484091
ILMN_2522750	TRIO	9.803086667	8.660626316	8.588198529	8.569590909
ILMN_2703182	LGALS7	5.84539	6.982352632	6.931479412	6.807209091
ILMN_2729252	5830431A1	8.978433333	7.842484211	7.881010294	7.62365
ILMN_1249366	LOC100046	8.23407	9.368084211	9.364801471	9.143586364
ILMN_2670778	KIF1B	9.548573333	8.416486842	8.422280882	8.337918182
ILMN_1254669	SYPL	9.745053333	10.87525263	10.84873088	10.82055
ILMN_2706514	LOC100046	7.5095	8.634492105	8.612260294	8.444136364
ILMN_2618148	C330008K1	8.92018	10.04062895	10.14341765	9.885018182
ILMN_2634796	SOCS1	6.6267	7.742844737	7.681489706	7.639990909
ILMN_2467190	BAMBI-PS1	8.600723333	7.486402632	7.445139706	7.239054545
ILMN_2917497	FCGRT	8.608183333	7.498360526	7.495925	7.383177273
ILMN_3130403	BC004728	8.17928	9.286213158	9.314702941	9.245672727
ILMN_2758264	CYP2S1	7.54357	6.441436842	6.418064706	6.3116
ILMN_2725259	IL2	6.447773333	7.549121053	7.490217647	7.949609091
ILMN_1247198	BC004728	9.205513333	10.29546842	10.36407941	10.27191818
ILMN_2612125	ARHGEF18	11.20628	10.12344737	10.09455882	9.868472727
ILMN_1218240	CD69	10.80300333	11.88487105	11.83365882	11.85055909
ILMN_2510694	NME7	8.52766	7.449481579	7.434319118	7.279186364
ILMN_2706232	SYNGR2	9.92304	10.98201579	11.08668088	11.19834545
ILMN_2754364	LTF	9.291486667	8.233002632	8.257197059	8.373727273
ILMN_2844996	ACTN1	10.95206667	9.899202632	9.844863235	9.639622727
ILMN_2596596	RGS1	7.544413333	8.596763158	8.683054412	8.546304545
ILMN_1234706	IGHA_J004	8.30976	7.263773684	7.221332353	7.160227273
ILMN_1244874	SYNGR2	9.75433	10.79814737	10.86721029	10.9914
ILMN_2724465	CCR8	5.868983333	6.910739474	6.962161765	6.952495455
ILMN_2890515	NSG2	7.526546667	6.487660526	6.466486765	6.366909091
ILMN_2690241	PKIB	6.76308	7.799152632	7.745076471	7.7524
ILMN_2731237	D8ERTD82E	7.88429	8.912992105	8.974445588	9.155922727
ILMN_1218037	TMIE	7.753076667	8.776018421	8.751725	8.420295455
ILMN_2740407	PDE2A	7.706153333	6.685718421	6.704382353	6.679877273
ILMN_2799452	IL12RB1	6.439253333	7.457005263	7.404963235	7.448872727
ILMN_2678724	DUSP10	7.155803333	8.172573684	8.260263235	8.096786364

ILMN_2669714	CTSA	8.099546667	9.113136842	9.205033824	9.180018182
ILMN_2636536	PLA2G12A	8.84106	9.8543	9.883701471	9.88105
ILMN_1224116	SMPDL3A	8.557293333	7.546207895	7.587558824	7.453181818
ILMN_2625377	RGS1	8.738546667	9.746213158	9.769272059	9.687659091
ILMN_2635272	IGH-VJ558	14.29845	13.29094737	13.22015294	13.18095
ILMN_2534151	IRGB10	8.701546667	9.704373684	9.691323529	9.5531
ILMN_1239797	6030400N1	7.333296667	6.331097368	6.341064706	6.274213636
ILMN_1249021	BCL2	7.82453	8.822494737	8.747633824	8.542981818
ILMN_2987709	SLC15A3	9.175186667	10.16891579	10.19635147	10.39172273
ILMN_2717975	TCF7	9.211256667	8.222889474	8.215898529	8.083790909
ILMN_2706231	SYNGR2	10.31389	11.30210263	11.42013235	11.58176364
ILMN_2769772	PIK3IP1	8.618043333	7.649376316	7.746248529	7.440986364
ILMN_2944824	HP	8.98318	8.016268421	8.174917647	8.30705
ILMN_2947234	CBX7	8.6477	7.682768421	7.728530882	7.684054545
ILMN_1242308	TCF7	9.232466667	8.26825	8.260585294	8.138781818
ILMN_3053593	GLIPR2	10.08712	11.04892632	11.06006324	10.93565455
ILMN_2658501	IFITM3	10.38816667	11.34819474	11.42799853	11.42086818
ILMN_2668333	PRG4	6.984513333	6.026434211	5.988755882	5.921604545
ILMN_1247893	CGEF2-PEN	9.050576667	8.096818421	8.110351471	7.883945455
ILMN_1248843	GATA3	7.34306	8.294652632	8.224369118	8.104072727
ILMN_2773900	GLIPR2	9.729866667	10.68112895	10.63013088	10.46443182
ILMN_2873750	GLDC	5.92132	6.870686842	6.770875	6.741440909
ILMN_2699898	ITGAE	8.418296667	7.469634211	7.501901471	7.236572727
ILMN_1217629	ITGAE	8.464196667	7.527947368	7.552825	7.319981818
ILMN_2897891	RGS1	7.728746667	8.660318421	8.707230882	8.614995455
ILMN_2806549	PRM1	5.990036667	6.916326316	6.894220588	6.788813636
ILMN_1229827	ST7	6.727336667	7.649644737	7.737477941	7.809095455
ILMN_1254634	ACPL2	8.50204	7.581063158	7.572779412	7.580222727
ILMN_2925094	MPO	7.105436667	6.185805263	6.332797059	6.577845455
ILMN_2687652	PRM1	5.935833333	6.854623684	6.849060294	6.777136364
ILMN_3047389	GBP2	7.03435	7.947555263	7.887723529	7.679313636
ILMN_2659739	IL7R	7.364306667	6.458986842	6.442688235	6.261427273
ILMN_1246543	SIAT7C	9.223836667	8.320423684	8.378747059	8.210077273
ILMN_2818964	DUSP10	7.31253	8.215365789	8.287358824	8.062272727
ILMN_2600928	OVGP1	7.453986667	6.557389474	6.540479412	6.507018182
ILMN_2631752	CTLA4	7.81105	8.706171053	8.619783824	8.483668182
ILMN_2663249	SLAMF9	9.11594	8.222615789	8.243369118	8.1212
ILMN_2614889	B3GNT8	9.81282	8.920905263	8.953698529	8.841236364
ILMN_2939681	LYZS	7.795363333	6.9062	6.970098529	7.154781818
ILMN_2696492	PHF11	8.6652	9.553802632	9.454427941	9.271490909
ILMN_1230045	4933437K1	8.913463333	9.798655263	9.824382353	9.735468182
ILMN_2996904	OBFC2A	6.96731	7.850726316	7.798394118	7.615513636
ILMN_2513451	1110046J1	11.05034	10.16700789	10.15531618	10.12564091
ILMN_1217180	IFITM1	10.89041667	11.77217632	11.84941765	11.83110455
ILMN_1259174	SCIN	8.775283333	9.655794737	9.640454412	9.632631818
ILMN_2481117	1700052O2	10.66834667	9.788118421	9.815542647	9.722204545
ILMN_1234539	IRGM1	10.58084333	11.46092105	11.438425	11.37995455
ILMN_2611755	SERPINB6B	7.277453333	8.156736842	8.188377941	8.19805

ILMN_1233293	GBP1	10.84405667	11.71913684	11.66407353	11.49095455
ILMN_2646625	JUN	7.34434	8.218031579	8.291385294	8.082372727
ILMN_2761720	LOC100041	8.500646667	7.629236842	7.612494118	7.366713636
ILMN_1237871	AMPD1	7.304966667	6.433939474	6.446288235	6.283995455
ILMN_2789650	CSRP3	6.64884	7.517015789	7.601960294	7.724718182
ILMN_2682162	BCL2	7.844683333	8.709405263	8.674883824	8.569540909
ILMN_2932359	TPI1	10.31272	11.17369737	11.19473235	10.92615455
ILMN_1214071	IFITM1	10.60328	11.4639	11.54568971	11.61521364
ILMN_1242622	CREM	7.411453333	8.271910526	8.247942647	8.217618182
ILMN_1237338	DDX25	7.421036667	6.560686842	6.532476471	6.45865
ILMN_1218525	IL18R1	8.817636667	7.958268421	7.945545588	7.754745455
ILMN_2636403	AXUD1	9.995656667	10.85401842	10.84707206	10.69045909
ILMN_2578341	B930094H0	7.454906667	6.597310526	6.497302941	6.428463636
ILMN_2658878	TG	6.5678	7.422852632	7.479155882	7.382472727
ILMN_2726471	EN2	5.743626667	6.598660526	6.582991176	6.530154545
ILMN_1214294	SNX29	6.848423333	7.702268421	7.713407353	7.705336364
ILMN_2646322	SAMSN1	10.94873	11.79982368	11.86731176	11.88902273
ILMN_2777019	SPO11	7.814436667	6.967128947	6.908411765	6.728213636
ILMN_2678726	RASGRP2	7.477166667	6.636023684	6.733875	6.734577273
ILMN_2700689	IBRDC3	8.3528	9.193568421	9.290398529	9.354804545
ILMN_1244866	GBP5	8.2183	9.058268421	9.012258824	8.881472727
ILMN_2765759	ASB2	8.020873333	8.860181579	8.794510294	8.691077273
ILMN_1239878	PRSS34	7.13797	6.298813158	6.278086765	6.330131818
ILMN_2689401	MCOLN2	8.4654	7.62645	7.5921	7.59695
ILMN_2447712	IBRDC3	6.955663333	7.792826316	7.789882353	7.901122727
ILMN_1223600	INDO	6.495596667	7.332621053	7.296913235	7.166577273
ILMN_2751603	PHXR4	9.941726667	9.109602632	8.973426471	8.817872727
ILMN_1221516	LOC100048	6.434853333	7.2654	7.302933824	7.260995455
ILMN_2419494	TNFRSF18	9.906223333	10.73479474	10.79889559	10.57273636
ILMN_1214783	A530050E0	8.485986667	7.659539474	7.615842647	7.62025
ILMN_1239729	KIF1B	8.392053333	7.567934211	7.516839706	7.370368182
ILMN_1233501	C530027B1	9.48782	8.663984211	8.683922059	8.504722727
ILMN_1253182	HS3ST1	8.033893333	8.855842105	8.805114706	8.860563636
ILMN_2638491	SLAMF7	7.82434	8.644642105	8.627872059	8.595940909
ILMN_2592953	SCG5	7.77508	6.956702632	6.960557353	6.79455
ILMN_1218036	SCO1	8.34345	9.161613158	9.071594118	9.090790909
ILMN_1229840	FCRLA	10.64000667	9.823918421	9.907154412	9.789463636
ILMN_1260046	SFT2D2	9.51672	10.33144474	10.2783	10.18119545
ILMN_2662097	RAB35	10.63486	11.44911053	11.42574559	11.5381
ILMN_2696491	PHF11	8.4808	9.294965789	9.237983824	9.094609091
ILMN_1249824	IGF1R	7.569356667	6.755618421	6.779692647	6.7302
ILMN_2789900	CD177	7.385106667	6.571739474	6.62825	6.835731818
ILMN_2665545	RIN3	9.836286667	9.024592105	9.014795588	8.852218182
ILMN_3122961	GBP2	12.90305333	13.71437895	13.71902353	13.60109545
ILMN_2602899	CREM	6.903246667	7.7135	7.713844118	7.7371
ILMN_1258965	TMEM66	11.24646333	10.43824474	10.41262794	10.26992273
ILMN_1238725	C130086J1	6.425446667	7.233239474	7.187627941	7.029145455
ILMN_2855315	HIST1H1C	9.337553333	8.530276316	8.505617647	8.621104545

ILMN_2658961	DGKA	11.82494	11.01769737	11.06722353	10.9061
ILMN_1217723	STAU2	7.913883333	8.719473684	8.736936765	8.7426
ILMN_2431237	XBP1	11.49460333	12.30005789	12.26956618	12.30432273
ILMN_2619200	ERAF	7.865656667	8.669657895	8.714435294	8.671418182
ILMN_1234698	TSPAN2	6.670486667	7.473368421	7.425954412	7.439659091
ILMN_2795178	RFTN2	8.005233333	7.209273684	7.217733824	7.237681818
ILMN_1258283	LTB	11.83477333	11.04274211	11.03906324	10.91391364
ILMN_2643513	ASNS	9.04453	9.835744737	9.741755882	9.861822727
ILMN_1258357	DOK3	12.26605	11.47897632	11.44765	11.44477727
ILMN_2491741	TRIO	7.336273333	6.549247368	6.611138235	6.557227273
ILMN_2492284	AA536717	7.253643333	6.468510526	6.484120588	6.349872727
ILMN_3128992	CD27	11.71615	10.93310263	10.95738971	10.65876364
ILMN_2742068	CSRP3	6.410026667	7.191834211	7.259801471	7.395309091
ILMN_2419490	TNFRSF18	10.22464333	11.00578158	11.07498088	10.83858636
ILMN_2650953	SLC12A7	7.512276667	6.731831579	6.744029412	6.646413636
ILMN_2749063	DOCK10	7.141206667	7.920371053	7.865589706	7.847540909
ILMN_2807084	ACOT7	8.594826667	9.372	9.352392647	9.372054545
ILMN_2473692	1110059G0	7.65595	6.880018421	6.850135294	6.816413636
ILMN_1237695	PFKP	9.860906667	10.63633421	10.65613824	10.56410455
ILMN_2592486	genericLYRI	8.485243333	7.710365789	7.785183824	7.734190909
ILMN_2630993	PPAP2B	8.248056667	7.473742105	7.506554412	7.530063636
ILMN_3006767	MLKL	7.866353333	8.638142105	8.575160294	8.697018182
ILMN_1244343	B230369L0	10.64261667	9.871126316	9.899548529	9.819563636
ILMN_2888191	CCR5	7.154613333	7.925273684	7.880173529	7.807190909
ILMN_1248413	FASL	6.024673333	6.795171053	6.780129412	6.697140909
ILMN_2803674	S100A9	11.68038333	10.91212105	11.05074559	11.24515455
ILMN_2691014	KLHL14	7.10078	6.333918421	6.30465	6.381831818
ILMN_2725448	SYTL3	6.27369	7.040426316	7.029935294	6.914209091
ILMN_2666272	HEMK1	8.380666667	9.14595	9.173805882	9.25
ILMN_2540103	LOC666559	9.92083	10.68557895	10.7199	10.70793636
ILMN_2488997	2010005O1	9.116286667	9.880084211	9.797192647	9.668695455
ILMN_3137287	LIF	5.682976667	6.44575	6.403722059	6.416013636
ILMN_2668510	HP	8.153523333	7.391392105	7.507407353	7.527177273
ILMN_2646891	CENTD1	10.04395333	10.80583684	10.76900441	10.81540909
ILMN_2614161	LSS	8.567596667	9.328973684	9.397113235	9.497386364
ILMN_2976440	BLK	10.83170333	10.07106842	10.05113971	10.12329545
ILMN_2654074	SESN1	11.24036667	10.47988947	10.44926176	10.32896818
ILMN_2593143	DOCK10	10.52829667	11.28851579	11.30300882	11.33595
ILMN_1239397	LOC100047	9.405073333	10.16416842	10.16762353	10.09550909
ILMN_2668509	HP	7.943053333	7.184218421	7.287372059	7.381036364
ILMN_1232621	ANK	7.856653333	7.099386842	7.122891176	7.169036364
ILMN_1221700	ELA2	7.074613333	6.318086842	6.367277941	6.59045
ILMN_2734391	RAMP1	7.914883333	7.158755263	7.212301471	6.993427273
ILMN_1230137	TPI1	11.74445667	12.50032895	12.51968382	12.25161818
ILMN_1234746	AI790298	7.578593333	6.822742105	6.873454412	6.792159091
ILMN_2648454	SIGLECG	10.3029	9.547089474	9.551536765	9.551795455
ILMN_2716622	MAPK11	9.600273333	8.844921053	8.852513235	8.744913636
ILMN_2685023	HMHA1	12.16146	11.40637895	11.42380441	11.32649091

ILMN_1231814	CCL5	9.356793333	8.602223684	8.662139706	8.673418182
ILMN_2818246	CPNE4	7.812076667	7.057652632	7.052097059	6.961063636
ILMN_1254685	EG240327	6.397796667	7.151681579	7.153916176	7.099763636
ILMN_2940446	DGKA	11.23734667	10.48404737	10.49755588	10.33128182
ILMN_2862379	RHOD	7.05575	6.304131579	6.303676471	6.307731818
ILMN_2733778	IL4I1	12.624	13.37522368	13.390125	13.45152273
ILMN_2545445	HIPK2	6.55741	7.308373684	7.332480882	7.280054545
ILMN_2700168	CCND2	10.03954667	10.79047632	10.82301765	10.75205455
ILMN_2618918	SLC2A6	9.982256667	10.73254211	10.75579118	10.77306364
ILMN_2881296	TMEM66	10.33295333	9.5849	9.557625	9.396040909
ILMN_2700166	CCND2	10.59667667	11.34464211	11.37600588	11.32170909
ILMN_2696609	MSCP	8.963526667	8.215607895	8.264191176	8.327386364
ILMN_1234973	A630004K0	6.210026667	6.954189474	6.972236765	6.985181818
ILMN_2492264	WISP1	5.968076667	6.711328947	6.711036765	6.729045455
ILMN_2606180	ACOT7	8.450883333	9.193897368	9.205269118	9.229513636
ILMN_1233589	CD27	8.96667	8.224807895	8.296473529	8.010390909
ILMN_2459899	ADAMTSL4	7.314856667	8.055031579	8.038655882	7.792595455
ILMN_2617468	CHAC1	7.318223333	8.0581	8.051151471	8.204913636
ILMN_1225730	FDPS	11.93995	12.67975	12.64576029	12.72600455
ILMN_1239632	NG23	9.102366667	8.364705263	8.462726471	8.421986364
ILMN_2833243	C330023M(	9.257843333	9.993915789	9.987714706	9.956754545
ILMN_3131478	BCAT1	8.48867	9.224363158	9.234775	9.319163636
ILMN_2734212	CD1D1	8.991273333	8.255613158	8.262241176	8.221804545
ILMN_2615096	DPP4	10.40495	9.669584211	9.690317647	9.540363636
ILMN_1258864	BC106179	8.446476667	7.711865789	7.749288235	7.574309091
ILMN_1229197	OTTMUSG(	8.368543333	7.633965789	7.618216176	7.357127273
ILMN_1255743	IL6RA	8.160456667	7.426255263	7.478202941	7.268536364
ILMN_2560567	A630006E0	10.59341	9.863042105	9.936775	9.685986364
ILMN_2619107	LGALS1	9.918706667	10.6487	10.70566324	10.63135
ILMN_2619316	PRNP	9.040923333	9.770686842	9.719486765	9.604395455
ILMN_2960108	CYP27A1	6.88932	6.160155263	6.166485294	6.144959091
ILMN_3153982	NACC2	7.91061	7.181697368	7.109570588	7.017036364
ILMN_2695047	PNPLA7	8.31495	7.586323684	7.637077941	7.563486364
ILMN_2976441	BLK	11.54462667	10.81708684	10.82114853	10.89478636
ILMN_3136744	SESN1	10.62493	9.897607895	9.837792647	9.802745455
ILMN_2710905	S100A8	13.23183	12.50617368	12.62048529	12.83921818
ILMN_1256644	SLC6A12	7.60547	6.879828947	6.942016176	7.050290909
ILMN_2639036	HSPD1	8.82105	9.546586842	9.439136765	9.368622727
ILMN_2490076	PKIB	6.435753333	7.161171053	7.095933824	7.160954545
ILMN_1232668	MAD	7.291183333	8.015381579	7.986783824	7.835204545
ILMN_2828768	USP28	8.408456667	7.685742105	7.665252941	7.660695455
ILMN_2860649	GBP6	7.584156667	8.306197368	8.263667647	8.019059091
ILMN_2517611	7530408C1	7.806793333	7.085144737	7.035369118	6.93015
ILMN_2865016	CD83	11.44668667	12.16742368	12.24165294	12.34662273
ILMN_1220101	EBI2	10.51159667	11.23125263	11.28764265	11.27350909
ILMN_2715042	SDC3	8.413436667	9.132221053	9.146326471	9.056245455
ILMN_2860645	GBP6	8.79986	9.517697368	9.558605882	9.364745455
ILMN_1248028	EMP3	10.66351333	9.946255263	10.05402647	10.04704545

ILMN_1220236	CTSG	8.164396667	7.447392105	7.593658824	7.725227273
ILMN_2774537	HIST1H1C	8.914876667	8.198747368	8.165147059	8.258654545
ILMN_2678127	RNF144A	8.33433	7.618615789	7.589805882	7.484554545
ILMN_3162239	PRR7	7.936	7.221010526	7.209419118	7.151090909
ILMN_1255860	KLRD1	9.2035	8.489178947	8.505567647	8.348959091
ILMN_1212653	RFTN2	7.837833333	7.125721053	7.108895588	7.111963636
ILMN_2457437	A830080H0	10.58319667	9.872428947	9.842497059	9.785954545
ILMN_2852672	TSPAN32	10.27871	9.570586842	9.543492647	9.500654545
ILMN_1254314	4931429I11	7.49642	6.788428947	6.757355882	6.756540909
ILMN_2696610	SLC25A37	8.959986667	8.252265789	8.278280882	8.350490909
ILMN_2712986	CHI3L3	8.252926667	7.545768421	7.58855	7.786881818
ILMN_1227277	D9ERTD392	10.15505	9.448323684	9.413408824	9.290313636
ILMN_3005441	PPA1	11.77690333	12.47936316	12.44846176	12.45698636
ILMN_2553041	A130093I21	8.614756667	7.912957895	7.931957353	7.739868182
ILMN_2457571	MAML2	9.231806667	8.530407895	8.504491176	8.382118182
ILMN_3103896	TIMP1	6.028073333	6.728947368	6.733719118	6.625322727
ILMN_2758029	PRTN3	7.127993333	6.427576316	6.471620588	6.740686364
ILMN_1221048	PSAT1	9.916976667	10.61730789	10.49174706	10.59564545
ILMN_2757569	ENO3	9.369076667	8.668905263	8.710376471	8.733259091
ILMN_2804559	TMEM108	8.12613	7.426189474	7.472713235	7.367513636
ILMN_2690460	JAKMIP1	10.34174667	9.644271053	9.597117647	9.541822727
ILMN_2883326	PIRA3	8.234013333	7.537413158	7.603591176	7.675386364
ILMN_2675922	2310047A0	8.327533333	7.632757895	7.605654412	7.557727273
ILMN_1228804	A430106G1	7.31336	6.618973684	6.595263235	6.54365
ILMN_1239055	XDH	6.36512	7.058510526	7.064069118	6.989463636
ILMN_2650739	ARHGAP9	9.78214	9.089992105	9.140601471	9.032004545
ILMN_2500370	9430080K1	10.87341	10.18259737	10.17046471	10.11225
ILMN_2611532	SLC25A19	8.19492	8.885142105	9.008430882	9.032790909
ILMN_2735615	ISG20	7.518246667	8.208171053	8.139072059	8.239459091
ILMN_3156343	WARS	8.263773333	8.951560526	8.995901471	8.8753
ILMN_3157081	SYTL3	6.38456	7.070873684	7.083748529	7.023881818
ILMN_2632264	ACOT7	10.69470667	11.38071053	11.36450147	11.44196818
ILMN_2631610	TMEM71	7.29944	6.615542105	6.536094118	6.333768182
ILMN_2754158	TGTP	7.284873333	7.967889474	7.893519118	7.793495455
ILMN_3018445	CYP4F18	7.031773333	6.349197368	6.342822059	6.333136364
ILMN_1258364	5430434G1	9.318306667	8.636010526	8.610383824	8.485986364
ILMN_2660182	ACOT7	7.83013	8.510831579	8.549666176	8.507154545
ILMN_2971142	AMPD1	6.766323333	6.086144737	6.075851471	6.011377273
ILMN_1244516	RASGRP2	7.476703333	6.7969	6.799802941	6.836954545
ILMN_2703829	FCRLA	12.25565	11.57618421	11.55282353	11.51362273
ILMN_2629191	CPM	9.91393	9.234957895	9.169702941	9.147559091
ILMN_2979248	TMED8	8.15378	8.831739474	8.811263235	8.855245455
ILMN_1221530	ADCY7	9.032993333	8.355923684	8.271894118	8.166209091
ILMN_1236111	FCRLA	12.55218	11.87590263	11.88261912	11.83079091
ILMN_1213609	TXNIP	12.58893667	11.91322368	11.88612353	11.75423182
ILMN_2816180	LBH	11.77342667	11.09807368	11.09418676	11.01050455
ILMN_2606072	AGFG1	8.354076667	9.028615789	9.006667647	8.942363636
ILMN_2751111	4931429I11	7.654283333	6.980063158	6.943754412	6.948795455

ILMN_1234054	TMED8	7.90326	8.575631579	8.518032353	8.587863636
ILMN_2766875	OCIAD2	6.029033333	6.699218421	6.716430882	6.700781818
ILMN_2524763	4930597A2	8.344646667	7.674497368	7.673958824	7.653013636
ILMN_3006123	ASNS	7.223503333	7.891473684	7.884744118	7.997090909
ILMN_2628174	ACSS1	10.95242667	10.28503158	10.25702794	10.11349091
ILMN_1233402	LOC100045	10.00150667	9.334863158	9.334583824	9.159545455
ILMN_1237074	SLC25A19	11.00412333	11.66939737	11.67991765	11.73784545
ILMN_2852034	HIF1A	7.287836667	7.951565789	7.906245588	7.818386364
ILMN_1250410	EIF2AK2	9.431796667	10.09508684	10.06052647	9.927563636
ILMN_2837493	EG634650	5.993646667	6.656815789	6.735947059	6.467818182
ILMN_2754698	CD84	10.28207333	9.619726316	9.660651471	9.608509091
ILMN_1256356	PML	10.07259	10.73383421	10.68198088	10.62860455
ILMN_1239472	CEACAM2	7.422096667	6.760892105	6.785801471	6.778659091
ILMN_2421220	BRWD1	8.272236667	7.614028947	7.590058824	7.579027273
ILMN_1233999	PPP3CC	8.017933333	8.674994737	8.723741176	8.655763636
ILMN_1245731	HHEX	10.25222333	9.595684211	9.542633824	9.5176
ILMN_2656854	MYO6	7.86478	8.521181579	8.467532353	8.266945455
ILMN_2731760	MYO1F	9.27305	8.617213158	8.592685294	8.631081818
ILMN_2642462	GTF2I	12.18328	11.52754211	11.51643971	11.44292727
ILMN_1218533	A630054M1	8.19438	8.849878947	8.827914706	8.862331818
ILMN_3115917	HSD11B1	9.131606667	8.476536842	8.515530882	8.443059091
ILMN_2945940	RAgenericE	7.861843333	7.20755	7.202657353	7.017481818
ILMN_2433990	LOC100048	8.55353	9.207039474	9.203336765	9.144786364
ILMN_2752436	2210010N0	7.966906667	7.314218421	7.364426471	7.283672727
ILMN_1258197	D230004N0	7.73881	8.391144737	8.361938235	8.371745455
ILMN_1218181	IFITM6	6.918566667	6.268265789	6.280010294	6.432531818
ILMN_2742042	NUPR1	6.801553333	7.451723684	7.512701471	7.494413636
ILMN_3112873	TXNIP	11.50670667	10.85741053	10.80823824	10.65764545
ILMN_1254405	LMNA	8.1473	8.796184211	8.835444118	8.852681818
ILMN_2705878	LIMD2	8.693046667	8.044581579	8.037157353	7.890031818
ILMN_2594525	NSDHL	7.7064	8.354552632	8.365227941	8.322163636
ILMN_2606825	CMAH	8.312323333	7.6647	7.634252941	7.396363636
ILMN_2522460	3010031K0	8.543816667	9.191336842	9.234385294	9.170268182
ILMN_3132898	YPEL3	13.39026333	12.74307368	12.76953824	12.67990455
ILMN_1243621	A130026C1	9.7118	9.064681579	9.029929412	8.839186364
ILMN_2751037	SPATA13	11.19429	10.54886053	10.48956912	10.47108636
ILMN_1232235	THADA	9.334693333	8.690102632	8.651617647	8.673077273
ILMN_1254157	WARS	9.735603333	10.37886316	10.35691912	10.27182273
ILMN_2777769	H2-DMA	11.86790667	12.51002368	12.53100441	12.56078636
ILMN_1228832	NGP	8.59455	7.952828947	8.124844118	8.267295455
ILMN_1219896	LOC623121	9.744836667	9.106268421	9.038876471	8.866836364
ILMN_1255510	LMNA	8.915643333	9.554178947	9.570432353	9.583027273
ILMN_1230546	CLIC4	10.53869333	11.17695526	11.15126618	11.20304091
ILMN_2724570	MAPK12	7.503686667	6.865684211	6.859533824	6.8399
ILMN_2977791	MAN2A2	7.365183333	6.727613158	6.778547059	6.671459091
ILMN_2722784	CD3G	11.37494	10.73794474	10.74789559	10.50351818
ILMN_3137291	LIF	5.86542	6.499805263	6.54505	6.506213636
ILMN_2983525	D12ERTD55	9.794036667	9.160018421	9.136989706	9.133836364

ILMN_1243249	2810410A0	10.7807	10.14750789	10.12662647	10.07005
ILMN_3141048	SEPP1	7.432253333	6.799544737	6.843607353	6.697863636
ILMN_2769918	TIMP1	5.969726667	6.602010526	6.577436765	6.519463636
ILMN_1237567	IL7R	6.91826	6.286176316	6.243760294	6.260904545
ILMN_1257803	A130095K0	6.768486667	6.137434211	6.146425	6.027690909
ILMN_1241171	ANXA3	8.77009	8.140389474	8.239335294	8.327272727
ILMN_2993314	CLEC4N	8.155996667	7.526681579	7.760479412	8.076522727
ILMN_1231204	LOC270152	7.684566667	8.313434211	8.328936765	8.274381818
ILMN_2758878	TMEM66	8.604496667	7.976713158	8.059894118	7.877477273
ILMN_2661185	SCML4	9.284993333	8.657247368	8.584035294	8.341754545
ILMN_1239346	KLRE1	6.16771	6.795257895	6.804683824	6.700913636
ILMN_2520264	2010016I18	8.114473333	7.487081579	7.539891176	7.383822727
ILMN_3014674	FAM169B	11.03242333	10.40547895	10.41317206	10.31021364
ILMN_2743902	MATK	7.576503333	6.950942105	6.954210294	6.817622727
ILMN_2757807	BC031353	9.67076	9.045855263	9.010575	9.019727273
ILMN_1236245	RAB11FIP4	7.012513333	6.387965789	6.419583824	6.470972727
ILMN_2591342	BC021614	8.086576667	7.463065789	7.519083824	7.435113636
ILMN_2697415	CD3D	12.02597333	11.40273947	11.44940588	11.19695909
ILMN_2581681	D530007E1	9.03998	8.417405263	8.385516176	8.304936364
ILMN_2601453	A130092J0	11.47359667	10.85129211	10.81008529	10.56360455
ILMN_2642913	EMP1	7.33474	7.956981579	7.96035	7.996531818
ILMN_2524865	CHI3L4	8.01563	7.393626316	7.45365	7.612545455
ILMN_1256219	LOC100047	11.70327667	11.08238158	11.13535147	11.07144545
ILMN_2903169	GM484	6.439876667	7.060573684	7.045426471	7.021390909
ILMN_3004864	UNC84B	11.49079	10.87096579	10.87125294	10.75787727
ILMN_2648669	GPNMB	7.951466667	7.331673684	7.401155882	7.527768182
ILMN_1216313	STIM1	8.977736667	8.359431579	8.348152941	8.196377273
ILMN_3139253	BTBD11	7.22281	6.604578947	6.573738235	6.500940909
ILMN_2728270	B4GALNT1	10.79857333	10.18042632	10.25875294	10.24815
ILMN_1236507	5830496L1	10.00914667	9.393421053	9.308585294	9.088395455
ILMN_1221820	FAIM3	14.32124333	13.70555526	13.69330147	13.7856
ILMN_2766604	CAMP	8.722323333	8.106676316	8.175870588	8.264754545
ILMN_2444984	MACF1	8.425146667	7.809781579	7.706641176	7.701818182
ILMN_3131679	USP18	9.363286667	9.978313158	9.931382353	9.806536364
ILMN_2692615	TGM2	7.39533	8.009855263	8.055310294	8.010068182
ILMN_2449150	MAP4K2	9.819663333	9.205721053	9.206395588	9.199613636
ILMN_1249975	IGHG	9.61923	9.005610526	8.89495	8.797990909
ILMN_2571414	NDR3	6.860516667	6.247115789	6.288842647	6.190795455
ILMN_2923865	CASP6	8.833286667	9.446118421	9.522104412	9.610272727
ILMN_3121891	LMNA	7.756683333	8.369328947	8.354533824	8.464245455
ILMN_2619408	ATF3	6.142813333	6.755302632	6.790607353	6.803554545
ILMN_2964185	H2-M2	7.242236667	6.630168421	6.649022059	6.640009091
ILMN_2685393	CCR5	6.59509	7.20655	7.201767647	7.132563636
ILMN_2856926	GPR114	9.017556667	9.628647368	9.590291176	9.239136364
ILMN_1221311	TTYH3	8.409613333	7.799102632	7.858344118	7.778395455
ILMN_3034877	KIF1B	7.821043333	7.211271053	7.208664706	7.2898
ILMN_1258587	LOC100044	9.9625	9.352865789	9.335433824	9.182454545
ILMN_1218717	LOC385615	8.83002	8.220628947	8.213408824	7.985740909

ILMN_2594521	NSDHL	7.481536667	8.090739474	8.077933824	8.035827273
ILMN_2919411	OSM	9.0134	9.622018421	9.631194118	9.557322727
ILMN_2573826	GAD1	6.285003333	6.893271053	6.891225	6.819222727
ILMN_1253354	DAPP1	7.88621	8.494418421	8.474091176	8.498481818
ILMN_2476452	CPM	7.303943333	6.69575	6.678776471	6.617622727
ILMN_2834573	BRWD1	8.29335	7.685668421	7.683051471	7.642636364
ILMN_2591754	QPRT	7.878863333	7.271292105	7.245197059	7.181613636
ILMN_2738433	IL4RA	6.239963333	6.846697368	6.896517647	6.838059091
ILMN_2695158	KIFC2	7.343773333	6.737642105	6.732835294	6.713495455
ILMN_2685392	CCR5	6.51683	7.122668421	7.051832353	7.004754545
ILMN_1229746	ECM1	6.132986667	6.738778947	6.806132353	6.615786364
ILMN_2984744	EMP3	12.08539	11.47984474	11.51957794	11.52749091
ILMN_2652500	LRG1	6.716696667	6.111805263	6.120491176	6.114954545
ILMN_2518483	PLA2G12A	6.280293333	6.883357895	6.955323529	6.928695455
ILMN_2845272	KLHDC2	11.45535	12.05764211	12.05591029	12.03670455
ILMN_3105936	CETN4	6.688636667	7.2909	7.333645588	7.254654545
ILMN_3117876	CHI3L3	8.021693333	7.419489474	7.418464706	7.543572727
ILMN_2971171	FCRLA	11.14227667	10.54146579	10.48822647	10.40451818
ILMN_1225132	4930519L0	6.88778	7.488065789	7.525252941	7.638254545
ILMN_2774410	STFA1	8.846636667	8.247494737	8.3801	8.518636364
ILMN_3002095	IL27RA	11.1748	10.57675	10.51693824	10.35572273
ILMN_2543417	AUH	7.760993333	8.358818421	8.450914706	8.496777273
ILMN_1257771	LOC638301	10.25393667	9.656502632	9.600141176	9.438890909
ILMN_2692960	ERO1LB	9.701216667	10.29802105	10.31935588	10.34075909
ILMN_2673233	PFKP	7.466366667	8.062705263	8.096848529	7.994904545
ILMN_1244891	CST7	7.014076667	7.610355263	7.708827941	7.532645455
ILMN_2651886	genericK1	10.80276	11.39885	11.35615735	11.28045455
ILMN_1228917	C330023M0	9.177703333	9.773615789	9.762547059	9.792254545
ILMN_3135781	ANXA3	8.453543333	7.858018421	7.964625	8.134063636
ILMN_2454823	6720418B0	8.454683333	7.859492105	7.843507353	7.601404545
ILMN_1244853	LOC100044	10.73101	11.32439474	11.35923971	11.16399545
ILMN_1254577	AI607873	6.892946667	7.486244737	7.533180882	7.504613636
ILMN_1222543	UGT1A10	6.93978	6.34705	6.38325	6.614104545
ILMN_2458765	AHNAK	9.03018	8.437463158	8.463539706	8.512445455
ILMN_2777696	GCAT	8.453923333	9.046523684	8.955386765	8.900027273
ILMN_2591156	IL27RA	9.22076	8.628213158	8.582977941	8.52225
ILMN_2676543	GRIFIN	6.82902	6.237215789	6.233313235	6.192986364
ILMN_2589401	PLTP	6.6056	7.196813158	7.226439706	7.17085
ILMN_2641360	AK2	9.80652	10.39720263	10.39346912	10.44981818
ILMN_2678431	MNS1	7.623163333	8.211194737	8.219722059	8.216736364
ILMN_1252496	WDR9	8.256553333	7.668823684	7.624570588	7.593372727
ILMN_2919259	SCD2	6.989606667	7.577144737	7.493097059	7.432013636
ILMN_2960114	CYP27A1	7.016623333	6.429744737	6.441141176	6.38235
ILMN_2628178	SOCS2	9.017703333	9.604081579	9.548186765	9.538022727
ILMN_2716511	SLC12A6	10.52652333	9.940578947	9.952173529	9.941263636
ILMN_2609813	CHI3L1	9.720116667	9.134286842	9.338616176	9.407072727
ILMN_1226514	GAB3	8.101403333	7.515847368	7.562842647	7.41685
ILMN_2699531	RGS10	10.51234667	9.926992105	9.937233824	9.797122727

ILMN_1217899	ITK	6.49019	7.075144737	7.055417647	7.021372727
ILMN_2964986	CREM	6.63287	7.216378947	7.199739706	7.17265
ILMN_1245300	F730045P10	7.17087	7.754107895	7.741594118	7.777690909
ILMN_3007862	ABHD14B	8.3503	7.767239474	7.744358824	7.750127273
ILMN_1222036	PAQR7	7.079483333	6.496497368	6.448283824	6.485727273
ILMN_1214998	HSD11B1	8.95546	8.372778947	8.421463235	8.356695455
ILMN_2645275	MVD	8.810396667	9.392594737	9.422547059	9.527990909
ILMN_1217102	TPCN1	9.111683333	8.529544737	8.543391176	8.474540909
ILMN_2692554	9330186A1	7.414916667	6.833423684	6.748882353	6.802618182
ILMN_2605858	AI646023	6.66345	6.082005263	6.04915	6.055072727
ILMN_2652857	IFI47	11.22192333	11.80331316	11.72213676	11.57583636
ILMN_1257444	SLC37A1	7.32784	7.907944737	7.907548529	7.987254545
ILMN_2450767	DUSP4	8.0951	8.675071053	8.696419118	8.827554545
ILMN_2611450	IFT172	9.435683333	8.855721053	8.856341176	8.830854545
ILMN_1242769	AKAP8L	10.58592	10.00616053	9.970663235	9.833063636
ILMN_2756438	D7BWG061	7.023296667	6.444452632	6.424191176	6.389677273
ILMN_1219017	5031436O0	8.368066667	7.789578947	7.810842647	7.816740909
ILMN_2493030	2310043N1	9.993046667	9.414568421	9.383005882	9.301872727
ILMN_1217913	D230007K0	9.815053333	9.236857895	9.225373529	8.991240909
ILMN_1256430	MCOLN3	7.33355	6.755771053	6.748322059	6.62405
ILMN_2650255	MAN2A2	7.403953333	6.826876316	6.818861765	6.736995455
ILMN_1248714	CD55	8.813533333	8.236618421	8.2139	8.190377273
ILMN_2740628	NDRG3	7.83159	7.255418421	7.260383824	7.269290909
ILMN_2761109	CLIC4	11.06524333	11.64044474	11.625275	11.63201364
ILMN_1230605	GM336	7.11325	7.687934211	7.6587	7.673177273
ILMN_1219860	LTK	8.006776667	7.432202632	7.398382353	7.413186364
ILMN_2810882	PPIC	9.265503333	8.691218421	8.651352941	8.542872727
ILMN_2807335	3110001A1	9.544203333	8.970439474	8.958279412	8.976627273
ILMN_2504447	EG330070	7.050706667	6.477302632	6.498975	6.470545455
ILMN_2864309	OTTMUSG0	8.49457	7.921613158	7.999452941	8.111331818
ILMN_1234020	SCL000200	9.443223333	8.870315789	8.903952941	8.853154545
ILMN_1228213	IFI30	11.57212333	12.14438947	12.18978676	12.1859
ILMN_1247199	LOC100046	8.874063333	8.301918421	8.328780882	8.228481818
ILMN_3077377	WARS	7.11178	7.6835	7.700954412	7.649859091
ILMN_1252295	LOC100038	6.395856667	6.966326316	6.988614706	6.970359091
ILMN_2644350	THY1	10.65563333	11.22597105	11.23514412	11.07841364
ILMN_1253008	ACAT2	8.73794	9.308271053	9.341198529	9.285022727
ILMN_2629112	ASAH3L	6.420043333	6.989957895	6.995489706	6.845595455
ILMN_2693679	CCDC125	7.619536667	7.050602632	7.065383824	7.088504545
ILMN_3149776	B3GNT8	7.61935	7.050542105	7.034622059	7.055154545
ILMN_2823778	SC4MOL	9.339616667	9.908247368	9.800502941	9.942090909
ILMN_2733185	CDC42EP3	8.85545	8.286834211	8.258167647	8.201140909
ILMN_2725402	NSDHL	7.48332	8.050342105	8.035323529	8.0115
ILMN_2833248	C330023M0	8.10134	8.667568421	8.672432353	8.655227273
ILMN_2774160	HSD11B1	8.706833333	8.140992105	8.175951471	8.124913636
ILMN_2711948	LOC100047	10.43146667	10.99716842	10.94898088	11.03114545
ILMN_2763739	BC032203	10.80728	10.24174474	10.17495441	10.13608636
ILMN_2593554	IGTP	12.18893	12.75399474	12.77557059	12.59678636

ILMN_2733733	TLR2	8.649903333	8.085921053	8.190616176	8.354554545
ILMN_2605453	RANBP10	10.22959667	9.666002632	9.65415	9.647813636
ILMN_2919263	SLC25A1	9.166143333	9.728544737	9.787044118	9.847804545
ILMN_1220397	TTC28	7.667103333	7.1052	7.127416176	7.132168182
ILMN_1249740	TSSC6	7.490173333	6.929126316	6.912072059	6.813518182
ILMN_1245754	CD84	7.52019	6.959386842	6.929688235	6.729927273
ILMN_2930552	9130211I03	6.768166667	7.327544737	7.359879412	7.235159091
ILMN_2698519	HIST1H3H	7.513023333	8.071597368	8.014075	8.023959091
ILMN_2721571	SLAMF1	8.52409	9.082347368	9.069436765	8.967859091
ILMN_2900462	ATP2A3	10.93327	10.37553947	10.34407206	10.38361364
ILMN_2883267	LRRK2	9.364593333	9.921984211	9.942058824	9.879545455
ILMN_1248651	C330023M0	8.314133333	8.871386842	8.864563235	8.889795455
ILMN_2531737	LOC240672	7.56991	8.125805263	8.094517647	8.219213636
ILMN_1215796	LOC100046	6.529113333	7.084997368	7.035101471	6.976813636
ILMN_2703563	STAC2	7.38922	6.834084211	6.875988235	6.836763636
ILMN_2599719	SLC44A2	11.92352	11.36847105	11.35597647	11.30884091
ILMN_1227570	LOC386545	8.743896667	8.189294737	8.230036765	7.990959091
ILMN_1222821	ROGDI	8.786446667	8.232284211	8.291411765	8.395709091
ILMN_2493826	UGT1A10	7.023866667	6.470215789	6.490170588	6.716004545
ILMN_1230143	BATF2	5.85682	6.409157895	6.445595588	6.382068182
ILMN_1229193	MSN	10.28875	9.737642105	9.735795588	9.645190909
ILMN_2483811	2210408F1	11.01110667	10.46008421	10.42315588	10.34929091
ILMN_2660837	ST6GALNAC	7.92546	8.476447368	8.480754412	8.539186364
ILMN_2767918	IFI30	10.28789667	10.83849737	10.96806324	11.00708182
ILMN_2725414	CD9	9.422196667	8.872436842	8.904213235	8.970577273
ILMN_1231447	TPI1	7.384893333	7.934492105	7.937911765	7.839004545
ILMN_2700354	DENND5B	8.391203333	7.842021053	7.911129412	7.785286364
ILMN_1224945	E130113K2	8.219303333	7.670147368	7.685198529	7.493963636
ILMN_1226755	E030007N0	9.077833333	8.528928947	8.625920588	8.525272727
ILMN_1225214	CDKN2D	8.793143333	8.244407895	8.240169118	8.105931818
ILMN_1212982	ZFP318	10.27203	9.723307895	9.703748529	9.6047
ILMN_1254218	NISCH	10.79873	10.25001842	10.28934265	10.102
ILMN_2474052	5830411I20	9.479836667	10.02834474	10.04819559	9.973677273
ILMN_1226901	MAN2A2	7.381073333	6.832571053	6.870908824	6.774418182
ILMN_2684600	NALP6	5.683113333	6.230942105	6.205075	6.286981818
ILMN_1217408	PRKACB	12.42386	11.87606842	11.88194706	11.79191818
ILMN_1241610	ADRB2	9.541823333	8.994415789	8.973683824	8.949086364
ILMN_2928599	SIGLECH	7.47144	6.924655263	6.936369118	6.803009091
ILMN_2712867	TIMP2	7.230993333	6.684518421	6.748505882	6.6003
ILMN_2593787	KCNK13	5.92443	6.470689474	6.479725	6.553636364
ILMN_1243150	A130010J15	9.42285	8.876610526	8.875494118	8.746018182
ILMN_2957862	NOC4L	9.10952	9.6555	9.677673529	9.723059091
ILMN_2846865	ACTB	10.15901333	9.613102632	9.645202941	9.835927273
ILMN_1218799	EMB	10.10022333	9.554318421	9.501319118	9.343713636
ILMN_3054914	USP18	7.713743333	8.259573684	8.224657353	8.138522727
ILMN_1238547	AREG	6.01426	6.559955263	6.514355882	6.508963636
ILMN_2711172	IRGM1	6.705733333	7.251423684	7.156748529	7.172445455
ILMN_2845080	PSAP	12.3915	11.84617895	11.88253382	11.91787727

ILMN_1239411	6430510M0	9.146923333	8.603228947	8.535841176	8.503222727
ILMN_3079919	AHNAK	7.987826667	7.444255263	7.509125	7.546490909
ILMN_1251984	C730026J16	8.910436667	9.453307895	9.458835294	9.488
ILMN_1242794	D630014A1	8.913583333	8.370760526	8.378547059	8.273154545
ILMN_3163572	ADCY7	8.611366667	8.068673684	8.002292647	7.965472727
ILMN_2537948	LOC624610	8.479553333	9.02185	8.960245588	8.972936364
ILMN_1246895	C78339	10.46701333	9.924855263	9.913516176	9.792804545
ILMN_1249014	TBX6	7.509416667	6.967384211	6.934380882	6.88865
ILMN_2988931	STFA1	8.375453333	7.83345	7.962795588	8.1119
ILMN_2863390	FCER1A	6.565336667	7.107023684	7.149798529	7.251063636
ILMN_1257107	LOC100043	11.29150333	10.74987368	10.77962353	10.63337727
ILMN_1228937	CYHR1	8.615836667	8.074321053	8.140413235	8.088863636
ILMN_2729958	HIST1H3D	9.276753333	9.817342105	9.843720588	9.779963636
ILMN_2664726	3110013H0	7.524233333	8.064392105	8.045011765	8.076427273
ILMN_2662387	LMNA	7.710816667	8.250452632	8.270158824	8.388381818
ILMN_2944601	4933439C2	10.40494	9.865452632	9.853114706	9.903972727
ILMN_1224473	LOC380797	8.568663333	8.029181579	8.096419118	7.870022727
ILMN_1225085	PRKCB	11.67589	11.13770789	11.13858382	11.04082273
ILMN_2604029	KLF2	11.83327	11.29523421	11.33738088	11.20542727
ILMN_2443164	SCL0002069	7.571263333	8.109255263	8.113289706	8.152909091
ILMN_1227907	GMFG	11.43036667	10.89250526	10.94621176	10.78452727
ILMN_1225764	MFSD2	7.970076667	8.507844737	8.490097059	8.5117
ILMN_2967266	FXYD5	12.00867333	11.47132632	11.60679559	11.49645
ILMN_1236354	E130302P1	7.808183333	7.271594737	7.243542647	7.254131818
ILMN_3115796	CD40	11.16950667	11.70592105	11.66586912	11.74807727
ILMN_2597710	LMNA	6.945293333	7.481457895	7.527360294	7.643618182
ILMN_1236256	ARHGEF1	10.30117333	9.765702632	9.777082353	9.565368182
ILMN_1231146	AGFG1	10.03638	10.57182895	10.54505441	10.58565
ILMN_2689731	DUSP4	7.44453	7.979928947	7.960576471	8.077036364
ILMN_2476329	WHRN	8.198216667	7.662876316	7.624944118	7.688081818
ILMN_3149680	FBXL12	9.55774	9.022781579	8.979083824	8.911004545
ILMN_2891245	RHBDL2	11.17166667	10.63687632	10.66231765	10.44623636
ILMN_3109491	6430527G1	10.69194667	10.15745	10.05446765	9.996531818
ILMN_1251713	CAR12	6.303143333	6.837231579	6.808888235	6.762877273
ILMN_2516221	TCRB-V8.2	8.713246667	8.179173684	8.227629412	7.963136364
ILMN_1234988	GAD1	6.138176667	6.672023684	6.655942647	6.64945
ILMN_2737713	EDN1	6.211046667	6.744673684	6.792708824	6.801963636
ILMN_1220739	NSG2	6.515793333	5.982639474	5.980491176	5.877881818
ILMN_2615739	GM459	10.43873667	9.905744737	9.871022059	10.01163636
ILMN_1228330	PLCL2	9.758736667	9.225823684	9.202452941	9.242722727
ILMN_3160842	BC087945	11.48216667	12.01505526	11.98382206	11.96960909
ILMN_1249637	PEG13	7.152553333	6.619871053	6.594275	6.661804545
ILMN_2620326	CYP27A1	6.618803333	6.086228947	6.130180882	6.038081818
ILMN_1247823	LRP8	6.931496667	7.464068421	7.458729412	7.48775
ILMN_2722996	SIRPA	8.616523333	8.084239474	8.143004412	8.417745455
ILMN_2914010	DMWD	6.59818	7.130428947	7.099555882	7.121695455
ILMN_1231858	FCRL1	9.11691	8.584847368	8.649417647	8.543518182
ILMN_2684515	SRPK3	11.26210333	10.73063158	10.67311765	10.63067727

ILMN_3160218	AMICA1	7.86067	8.392005263	8.426604412	8.322131818
ILMN_2469253	VPREB3	8.61418	8.083365789	8.268382353	8.165345455
ILMN_2616989	KCTD17	7.071666667	7.602447368	7.611333824	7.592340909
ILMN_2725595	THRA	6.775176667	6.244463158	6.252422059	6.26795
ILMN_1217406	1110013L0	8.25656	7.726518421	7.709145588	7.483686364
ILMN_1233545	LBH	9.173483333	8.643457895	8.585191176	8.667568182
ILMN_2502346	SAMSN1	6.83519	7.365021053	7.424139706	7.413554545
ILMN_1245924	MAP3K8	8.41872	8.948184211	8.970817647	8.893895455
ILMN_2760765	BC021381	8.589116667	8.059802632	8.060226471	8.142354545
ILMN_2664686	CHAF1B	8.82029	9.349602632	9.297583824	9.275677273
ILMN_2424721	PDGFA	6.399903333	6.928989474	6.853522059	6.927486364
ILMN_2502136	CD40	10.06332667	10.59213684	10.70311912	10.74522273
ILMN_3116935	BTLA	10.49572	9.967563158	9.945167647	9.868113636
ILMN_2925711	DUSP6	9.465566667	8.938113158	8.877455882	9.070204545
ILMN_2770119	ZBTB32	10.86225333	11.38918947	11.30878824	11.42603182
ILMN_1256702	S100A10	9.221003333	8.694873684	8.738216176	8.63095
ILMN_2833441	TRAFD1	10.66275667	11.18868947	11.17483971	11.16915455
ILMN_2522571	SETD7	8.49144	7.965744737	7.900425	7.745868182
ILMN_2448404	SCL000297	8.533736667	8.008318421	8.116742647	7.919968182
ILMN_2561749	A630072I1	6.90215	6.376881579	6.363104412	6.256609091
ILMN_2992653	MSH5	7.365693333	6.840486842	6.806542647	6.832295455
ILMN_3068231	MAX	7.004783333	7.529568421	7.488708824	7.388690909
ILMN_2775202	RRAGD	7.19449	7.719044737	7.690863235	7.569304545
ILMN_1253601	AACS	9.945826667	10.47036053	10.46929412	10.52398636
ILMN_2694170	CD97	9.80694	9.283168421	9.352319118	9.337640909
ILMN_1222471	GMFG	11.24841667	10.72540789	10.77340735	10.59015
ILMN_2923864	CASP6	8.035723333	8.557984211	8.620341176	8.727013636
ILMN_2497190	BMF	6.99474	6.472515789	6.411789706	6.365568182
ILMN_2750062	ARHGAP24	7.191523333	6.669510526	6.721417647	6.661459091
ILMN_2776952	TMEM55B	8.84177	9.363155263	9.356698529	9.371772727
ILMN_2880536	UCK2	10.53304667	11.05398684	11.00229706	11.02895909
ILMN_2694175	CD97	9.902043333	9.381402632	9.428180882	9.414463636
ILMN_1253851	HIST2H2BE	8.37224	8.892865789	8.900198529	8.836145455
ILMN_2756439	GRAMD1A	7.12322	6.602613158	6.640825	6.581772727
ILMN_1214703	NME7	6.773053333	6.252663158	6.253341176	6.174459091
ILMN_3151503	C130032J1	8.786306667	9.305681579	9.304608824	9.324440909
ILMN_2621901	BC004022	9.374146667	9.893202632	9.914832353	9.842027273
ILMN_2657685	AASS	6.827346667	7.346360526	7.374670588	7.304227273
ILMN_1221568	CDCA7	10.56978333	11.08840526	11.05373088	11.14640455
ILMN_1246270	GINS2	7.822786667	8.340892105	8.295725	8.257509091
ILMN_2688236	ATP2A3	12.58813	12.07048684	12.02009118	11.99795455
ILMN_2792601	P2RY5	10.28267667	9.765228947	9.736329412	9.777036364
ILMN_2760979	TGFBR2	12.03852	11.52211316	11.50930147	11.44098182
ILMN_1213483	FLCN	10.64120333	10.12503947	10.09291765	10.15793636
ILMN_2705628	CLEC4D	8.82084	8.305036842	8.482082353	8.779268182
ILMN_1214318	RASGRP1	6.667076667	7.182584211	7.145963235	6.966668182
ILMN_2771349	0610007P1	9.340376667	9.855694737	9.816160294	9.903672727
ILMN_2681601	SLC44A2	8.348826667	7.834094737	7.856692647	7.706281818

ILMN_1246609	RASGRP1	11.33855	11.85307105	11.85594265	11.60770455
ILMN_2508626	PEX11C	6.693453333	6.179115789	6.188005882	6.216986364
ILMN_2617820	PPP3CC	10.23106667	10.74528684	10.718925	10.57838182
ILMN_2433964	GIGYF1	9.174583333	8.660778947	8.685676471	8.544772727
ILMN_1229458	PRKCB	9.352536667	8.83875	8.763423529	8.807190909
ILMN_2945694	HIST1H3A	8.371906667	8.885407895	8.898982353	8.834895455
ILMN_1231309	LY9	10.57010333	10.05668158	10.06185147	10.05565
ILMN_2510383	TNFRSF25	8.859573333	8.346544737	8.361252941	8.340177273
ILMN_2711075	MMP9	7.191016667	6.678302632	6.702389706	6.812577273
ILMN_1249378	BHLHB2	9.07604	9.588647368	9.570416176	9.578259091
ILMN_2506012	TRP53INP1	10.08522	9.572681579	9.5451	9.469318182
ILMN_2509340	FOXO1	8.654366667	8.141878947	8.104170588	8.069745455
ILMN_1221703	ABCA7	10.62202667	10.11	10.13834265	10.13358636
ILMN_2665087	KLF1	6.577006667	7.0889	7.095722059	7.126468182
ILMN_2689307	SPNB2	8.52137	8.010315789	8.029977941	7.859663636
ILMN_1226261	FAM158A	7.96853	7.457605263	7.488210294	7.446190909
ILMN_1245354	TLR7	9.301983333	9.812560526	9.875805882	9.876613636
ILMN_2615672	PRELID2	6.108273333	6.618676316	6.652044118	6.542881818
ILMN_2418957	5930418K1	9.004963333	8.495173684	8.470373529	8.374686364
ILMN_2639012	CCR6	10.53305333	10.02367368	10.09640441	10.09141818
ILMN_1233917	LOC100041	6.175063333	6.684076316	6.631039706	6.519009091
ILMN_2645208	ARHGEF3	12.13058667	11.62189474	11.58621324	11.50325909
ILMN_1252488	CLEC2I	7.829716667	7.321136842	7.3058	7.242727273
ILMN_2925008	SFT2D2	6.48672	6.995207895	6.920232353	6.906186364
ILMN_2421246	TCRB-V8.3	8.367016667	7.858584211	7.941542647	7.684159091
ILMN_2742928	FXYD5	10.26460667	9.756181579	9.868969118	9.726668182
ILMN_2604556	PDE7A	7.412516667	6.904297368	6.839942647	6.878745455
ILMN_1260323	AKR1C18	5.69947	6.207665789	6.194161765	6.198459091
ILMN_2883268	LRRK2	9.31324	9.821171053	9.822963235	9.761345455
ILMN_2480682	TMEM23	8.860773333	8.353092105	8.289407353	8.190659091
ILMN_2748164	HIST1H3F	8.912953333	9.420621053	9.416923529	9.431840909
ILMN_1224034	PDE1B	9.389363333	8.881973684	8.900580882	8.945822727
ILMN_2712075	LCN2	11.52582333	11.01917105	11.18763088	11.47061818
ILMN_3125814	ACSL6	5.60918	6.115702632	6.077122059	6.127881818
ILMN_2790357	SEMA7A	8.332696667	8.839102632	8.841805882	8.810909091
ILMN_2416460	TRBV8_AE0	8.769396667	8.263507895	8.351523529	8.056295455
ILMN_2568028	IL2RG	9.496336667	8.990715789	9.037976471	8.851422727
ILMN_1247377	MPEG1	8.820523333	9.325578947	9.300376471	9.23945
ILMN_3153753	CACNB3	7.98161	7.476615789	7.583061765	7.606531818
ILMN_2588055	ACTB	9.934683333	9.429810526	9.439613235	9.614481818
ILMN_2880346	RRP1B	8.254853333	8.759510526	8.699454412	8.764309091
ILMN_2540344	LOC381889	7.8252	8.329739474	8.275372059	8.279772727
ILMN_1237625	FAM113B	8.187733333	7.683394737	7.662777941	7.734872727
ILMN_1214800	RNF167	9.872936667	9.368607895	9.405135294	9.303831818
ILMN_2519313	TMOD4	8.734043333	8.229944737	8.188375	8.138731818
ILMN_2724545	SBK	11.35983333	10.85581316	10.80280588	10.82701818
ILMN_2874816	CDC14B	7.573256667	7.069355263	7.069520588	7.048240909
ILMN_1224770	GPRASP1	9.77645	9.2726	9.234861765	9.250504545

ILMN_2681516	SLC39A6	8.814913333	9.318736842	9.304892647	9.30945
ILMN_1237990	GALE	6.785393333	7.288428947	7.329561765	7.342213636
ILMN_1228783	LOC100043	6.91082	6.407823684	6.473876471	6.429927273
ILMN_1237114	TMEM154	8.138643333	7.636152632	7.585944118	7.646022727
ILMN_2828916	FRMD6	8.007456667	8.509536842	8.570277941	8.408954545
ILMN_2824002	FRAT2	7.71096	7.208939474	7.178638235	7.178854545
ILMN_2687744	RNF167	9.199576667	8.697613158	8.739005882	8.606045455
ILMN_2920849	PIRA4	7.654663333	7.1527	7.232370588	7.233395455
ILMN_2718453	ARID5A	7.283676667	7.785302632	7.793682353	7.815790909
ILMN_2970532	MCM10	9.424043333	9.925539474	9.829147059	9.9184
ILMN_1235795	LOC675594	7.448003333	7.948989474	7.910132353	7.82655
ILMN_2524667	4833427B1	7.739353333	8.240297368	8.256905882	8.22985
ILMN_2673889	MCM7	8.074	8.574697368	8.487373529	8.401995455
ILMN_1259566	ST6GALNA(	7.177983333	7.678428947	7.679841176	7.755468182
ILMN_1246473	CCND3	7.324866667	7.825297368	7.841207353	7.725622727
ILMN_2830898	CDC25B	7.01245	6.512081579	6.527039706	6.583763636
ILMN_3071764	1200015F2	7.43188	7.931826316	7.960994118	7.896445455
ILMN_2628900	ITGAE	6.657026667	6.157218421	6.219135294	6.113486364
ILMN_1224472	CCL4	8.713336667	9.212842105	9.305651471	9.529104545
ILMN_2552490	6720463L1	10.58386333	10.08456579	10.07292353	9.899459091
ILMN_2635132	FOXP3	8.401456667	8.900668421	8.910723529	8.765727273
ILMN_1260585	STFA2	7.616773333	7.117684211	7.219723529	7.385345455
ILMN_2625035	SFT2D2	6.582473333	7.08145	7.109589706	7.160159091
ILMN_1237580	KLRA3	6.114223333	6.612936842	6.616825	6.557209091
ILMN_1244211	LOC238943	7.880973333	8.379628947	8.359485294	8.234081818
ILMN_2811263	ZXDA	10.18303333	9.684407895	9.719638235	9.468354545
ILMN_2661971	GM2A	11.28908333	10.79073421	10.82771912	10.73904545
ILMN_2730926	LSM12	8.963933333	9.462136842	9.368045588	9.434645455
ILMN_1247942	MGEA5	7.989676667	7.492289474	7.497663235	7.336986364
ILMN_1242024	SETD4	7.181716667	6.684497368	6.698276471	6.780113636
ILMN_2929896	PBK	8.14347	8.640523684	8.593113235	8.659018182
ILMN_2476733	1200016E2	8.391553333	7.895302632	7.857470588	7.77645
ILMN_2543929	2610036L1	9.511626667	10.00778421	10.03502353	10.05321364
ILMN_2456216	5330403D1	8.630133333	8.134192105	8.131176471	7.9148
ILMN_2727546	D930048N1	8.084543333	7.5889	7.561372059	7.503077273
ILMN_3059476	SESN1	7.46764	6.972268421	7.001925	6.872913636
ILMN_2834379	TGFBI	7.914363333	7.419384211	7.540954412	7.770859091
ILMN_1247704	HMGN3	11.16247667	11.65745263	11.61625	11.68214091
ILMN_1249888	ADCY6	7.810496667	7.315673684	7.367497059	7.301754545
ILMN_1229534	RNF167	9.146563333	8.651771053	8.652933824	8.584977273
ILMN_1244123	SLC38A2	10.97275	10.47811316	10.44468824	10.36232273
ILMN_1229957	IL11RA1	8.82848	8.334539474	8.307539706	8.341713636
ILMN_2757966	CXCL4	7.484453333	6.99065	7.080525	7.048609091
ILMN_2915951	D13ERTD6(	7.322256667	6.828815789	6.840588235	6.650377273
ILMN_2986605	PDSS1	7.05284	7.54625	7.506525	7.626577273
ILMN_1257631	APOBEC1	8.986086667	8.492778947	8.517795588	8.435559091
ILMN_1256025	LOC100041	6.884853333	6.391676316	6.44335	6.474904545
ILMN_2750725	PIGZ	5.767723333	6.260307895	6.21195	6.156859091

ILMN_2848828	CEP97	7.650426667	7.157897368	7.149170588	7.033681818
ILMN_2730293	PDE1B	9.44438	8.951897368	8.937267647	9.015109091
ILMN_2918002	GBP3	10.33665333	10.82889474	10.80859559	10.65228636
ILMN_2428252	RASA2	7.455803333	7.947894737	7.939733824	8.004595455
ILMN_1248837	TBXA2R	7.84156	7.349494737	7.354123529	7.182513636
ILMN_2866856	H2-DMA	12.52671	13.01846053	13.07551324	13.17113636
ILMN_1226555	5430417L2	9.897846667	9.406210526	9.400447059	9.308795455
ILMN_1212938	AIF1	6.576943333	7.068328947	7.123913235	7.142572727
ILMN_2484707	TYMS	9.133346667	9.624002632	9.627598529	9.622622727
ILMN_3148489	FURIN	10.46270667	10.95335	10.90541324	10.89725909
ILMN_2808485	GBP10	6.781303333	7.271921053	7.213405882	7.041513636
ILMN_1226143	RABGEF1	10.34694667	9.856457895	9.845116176	9.860609091
ILMN_2684563	CLDND1	8.172773333	8.663239474	8.625180882	8.674740909
ILMN_2670398	EIF4EBP1	9.293236667	9.783623684	9.711872059	9.773036364
ILMN_2977535	D930015E0	9.695646667	9.205305263	9.291123529	9.344186364
ILMN_1256257	LOC100038	10.10456	10.59481842	10.55595588	10.57585
ILMN_2613832	MGST2	9.044613333	8.554489474	8.546391176	8.333404545
ILMN_2646834	D11LGP2E	8.081966667	8.571726316	8.579542647	8.546236364
ILMN_1242399	HIST1H2BC	6.873676667	6.384471053	6.383308824	6.379063636
ILMN_2428798	5031439G0	8.838863333	8.350186842	8.323948529	8.293472727
ILMN_2644140	PANK4	10.61254	10.12393684	10.18636176	10.05719545
ILMN_3163044	OLA1	8.91342	9.401918421	9.352347059	9.322536364
ILMN_1227814	SRR	11.62509	11.13730526	11.21078088	11.00605909
ILMN_2644719	HMGN3	10.94508667	11.43285263	11.41632206	11.48374545
ILMN_2600348	SQLE	8.251303333	8.738868421	8.748764706	8.667427273
ILMN_2769971	LINCR	7.524023333	7.036918421	7.068355882	6.961972727
ILMN_2630328	PSMA7	11.52430333	12.01132105	12.01410294	12.02497727
ILMN_3006990	EG622339	12.98081	13.46777632	13.46261471	13.36065455
ILMN_2915060	DGKZ	9.53046	9.043510526	9.038536765	9.060131818
ILMN_2606693	INSIG1	7.262293333	7.748865789	7.678260294	7.641254545
ILMN_2728118	RRP12	9.71417	10.20048684	10.15763088	10.29602273
ILMN_2683811	GALE	6.75883	7.245031579	7.267663235	7.272968182
ILMN_2733524	NRM	11.53344	11.04740263	11.00323971	10.99424091
ILMN_2660551	LAT	9.573943333	9.088315789	9.004679412	8.757095455
ILMN_1241225	DCTD	7.73365	8.219268421	8.183508824	8.209872727
ILMN_1259564	IIGP2	10.87997667	11.36546316	11.33019118	11.20709545
ILMN_1214498	CYP2D22	8.11384	7.6284	7.634107353	7.595440909
ILMN_3148550	GOLM1	8.432583333	7.947163158	7.913177941	7.917890909
ILMN_1260512	MIF	11.02496667	11.51028421	11.51826471	11.4299
ILMN_1248389	INPP5K	12.04935667	11.56430789	11.54765294	11.48464091
ILMN_2547942	GINS2	7.60201	8.087055263	8.042544118	8.018609091
ILMN_1223257	CCL4	11.14475	11.62975263	11.75993824	11.86287727
ILMN_2622780	5530601I19	7.999706667	7.515147368	7.602066176	7.569886364
ILMN_2974064	OSBPL3	7.03954	7.523976316	7.509626471	7.605709091
ILMN_1228333	PRF1	6.592786667	7.077028947	7.063494118	6.897177273
ILMN_2915059	DGKZ	10.26964333	9.785439474	9.7872	9.816468182
ILMN_2721360	GALK1	9.165806667	9.649981579	9.677827941	9.563363636
ILMN_2608151	FCRL1	9.303546667	8.819431579	8.865113235	8.792713636

ILMN_2417174	UBE2H	7.774013333	7.291031579	7.304219118	7.268663636
ILMN_2901283	ADD3	10.85499	10.37201579	10.37048824	10.28376818
ILMN_2508001	CLTC	7.302493333	7.785318421	7.737447059	7.817286364
ILMN_2697615	MAPK12	7.052833333	6.570236842	6.618429412	6.654509091
ILMN_1230696	IGL-V1	12.40741667	11.92506842	12.00315735	12.03508182
ILMN_1237978	9930005O1	7.786423333	7.304186842	7.242588235	7.192336364
ILMN_1233141	KLRA22	5.737783333	6.220013158	6.217479412	6.088368182
ILMN_2597255	CDC6	7.808103333	8.289960526	8.186636765	8.23955
ILMN_1214578	CRTC3	8.313713333	7.832310526	7.893926471	7.840259091
ILMN_2878548	MTHFD2	8.144953333	8.626257895	8.628497059	8.6541
ILMN_2671738	IL16	10.34373	9.862731579	9.813888235	9.759777273
ILMN_2755021	CHKB	9.912793333	9.432428947	9.525319118	9.472736364
ILMN_1249217	LOC380763	8.500983333	8.020868421	8.019075	7.913895455
ILMN_2623536	GOLM1	8.876253333	8.396268421	8.368392647	8.371622727
ILMN_1257097	CNP	8.4583	8.937923684	8.996055882	8.950890909
ILMN_3142573	MAPK1IP1	8.969603333	8.490186842	8.481775	8.445359091
ILMN_1220284	PSMD7	9.257483333	9.736878947	9.752014706	9.74925
ILMN_3067831	ZFP187	9.802603333	9.323465789	9.313083824	9.315436364
ILMN_2665131	IHPK1	10.7729	10.29377105	10.30605147	10.30897273
ILMN_2600744	RGS16	6.451433333	6.930547368	6.940605882	7.104027273
ILMN_1220595	IL17A	6.454306667	6.933336842	6.982905882	6.86355
ILMN_2641793	DTX1	9.060943333	8.582018421	8.668863235	8.689840909
ILMN_2880529	UCK2	7.70306	8.181926316	8.224991176	8.167672727
ILMN_2819380	BC030476	11.90582667	11.42737895	11.43873382	11.16817727
ILMN_1232093	LOC386330	10.13935	9.660942105	9.652973529	9.685572727
ILMN_1242013	UCK2	9.413536667	9.891402632	9.895961765	9.882868182
ILMN_2899599	DDX3Y	9.722653333	9.245560526	9.202622059	9.122063636
ILMN_1242466	PSMB9	8.003013333	8.479313158	8.365239706	8.325395455
ILMN_2901284	ADD3	11.49975333	11.02383684	10.98140147	10.84233636
ILMN_2589477	DARS	8.916623333	9.392265789	9.302201471	9.310081818
ILMN_1255416	LY6A	14.19523333	14.67078421	14.64110735	14.66443636
ILMN_1236517	IL18	6.902506667	6.427465789	6.373588235	6.419559091
ILMN_2470564	IGHV1S120	9.692856667	9.217965789	9.226329412	9.119081818
ILMN_1240702	ATP1B3	12.44345333	11.96903947	11.89987059	11.84145455
ILMN_1242046	AQR	8.068703333	7.594457895	7.619404412	7.522959091
ILMN_1220972	IL16	7.42725	6.953173684	6.940511765	6.881495455
ILMN_3154810	SMARCA2	11.02438	10.55043684	10.50354265	10.53565909
ILMN_1255664	LOC100046	7.896743333	7.422971053	7.463869118	7.464240909
ILMN_2951691	HIST1H3E	9.41995	9.893715789	9.930129412	9.921827273
ILMN_1247832	CD74	10.73217667	11.20577895	11.29666471	11.35442273
ILMN_2918114	APEX1	8.05855	8.53215	8.552566176	8.5933
ILMN_1216386	CD86	8.408796667	8.882105263	8.877667647	8.874636364
ILMN_2663604	FIGNL1	8.27945	8.752171053	8.647223529	8.705381818
ILMN_2778122	PDCD4	11.32195333	10.84939211	10.88932794	10.78596818
ILMN_2565835	B930008G0	11.46631667	10.99378421	10.96833235	10.91645
ILMN_3020240	HBP1	10.99606333	10.52378947	10.556525	10.49137273
ILMN_1246339	6330403E0	9.15174	8.679492105	8.702944118	8.593131818
ILMN_2467429	D6MIT97	11.45000333	10.97782632	10.98452206	11.02852727

ILMN_1258394	AARS	10.25052333	10.72222632	10.64604118	10.8079
ILMN_2804166	IGSF9	7.72168	7.250102632	7.209426471	7.318354545
ILMN_1255750	IL7R	6.3964	5.925234211	5.865013235	5.894859091
ILMN_2737710	TIAM1	8.273783333	8.744307895	8.661008824	8.648690909
ILMN_2595814	A630082K2	10.71696667	10.24649474	10.22477353	10.16600909
ILMN_1214486	TK2	10.35887333	9.888563158	9.842289706	9.781713636
ILMN_2432255	LOC630337	9.698646667	9.228815789	9.138663235	9.038581818
ILMN_1248439	ST6GALNAC	8.2337	8.703436842	8.618572059	8.675536364
ILMN_2623056	CLSPN	8.064613333	8.534094737	8.475572059	8.56605
ILMN_2979639	H2-DMB2	11.28671667	11.75598684	11.78886176	11.93475455
ILMN_2869312	FBXO4	10.21257	10.68178421	10.61175	10.58898182
ILMN_2513781	A130038J17	7.070466667	6.601365789	6.61995	6.578772727
ILMN_1247762	RASAL1	7.21572	6.746647368	6.816261765	6.937468182
ILMN_1232667	IFITM2	10.14822	10.6172	10.71305735	10.73515909
ILMN_2918479	HSPA8	12.24956	12.71828947	12.60738235	12.47838636
ILMN_1251178	HS3ST3B1	7.42913	6.960407895	6.955142647	6.921104545
ILMN_3000080	1110038D1	10.41259667	9.943955263	9.934307353	9.923922727
ILMN_1230599	ADAM23	6.62476	6.156160526	6.138545588	6.24995
ILMN_2644092	AASS	7.530816667	7.999257895	7.906854412	8.041690909
ILMN_2668319	HSP90AA1	8.8777	9.345960526	9.299935294	9.217740909
ILMN_2723860	PKM2	8.854423333	9.322594737	9.358717647	9.247309091
ILMN_1214634	AQP9	6.903756667	7.371810526	7.386388235	7.409309091
ILMN_2866185	BTG1	13.71557333	13.24755263	13.26201324	13.26215
ILMN_2660466	EG433229	7.618856667	7.150876316	7.158276471	7.124095455
ILMN_2431398	2810001G2	7.20617	6.738457895	6.759751471	6.705422727
ILMN_2859847	PYGL	7.790936667	7.3233	7.422495588	7.578627273
ILMN_2883952	1810015A1	8.104256667	8.571594737	8.509154412	8.564577273
ILMN_1232989	EHD3	8.013176667	7.546005263	7.554476471	7.601840909
ILMN_2499056	IGKV2-137_	11.41894333	10.95204211	10.96605441	10.97854091
ILMN_1233116	MAP4K4	7.267023333	6.800147368	6.785232353	6.852222727
ILMN_2477221	CITED2	9.57618	10.04302895	10.01740882	10.04663636
ILMN_2933431	INPP5K	9.374156667	8.907313158	8.987426471	8.93495
ILMN_1224942	SHMT2	8.329433333	8.795918421	8.750304412	8.834072727
ILMN_2464474	9430029L2(	9.4229	8.95645	8.990469118	8.856822727
ILMN_3130350	GLIPR2	6.38496	6.850863158	6.813057353	6.812659091
ILMN_1225733	LOC677643	10.39900333	9.9334	9.931026471	9.857759091
ILMN_1252202	TNFAIP3	8.73625	9.200576316	9.205004412	9.258704545
ILMN_1252481	FOSL2	7.555466667	8.019776316	8.056545588	8.1423
ILMN_1228653	ZBP1	6.948423333	7.412642105	7.444173529	7.299622727
ILMN_1231168	H3F3B	9.362493333	8.898360526	8.812038235	8.781254545
ILMN_2858359	CLSPN	8.452636667	8.916521053	8.857144118	8.940954545
ILMN_2574982	SPN	7.25949	6.7957	6.822661765	6.698304545
ILMN_2600022	HEG1	8.128633333	7.664915789	7.681570588	7.608509091
ILMN_2593774	1190002H2	8.001166667	7.537692105	7.583389706	7.431922727
ILMN_2892856	RNF167	9.68891	9.225528947	9.181063235	9.116222727
ILMN_2899863	TNF	8.296036667	8.759357895	8.846445588	8.912131818
ILMN_2619846	SLC25A1	7.50881	7.971526316	8.031520588	8.062972727
ILMN_2804103	BC038822	7.910843333	7.448302632	7.379916176	7.437740909

ILMN_2469743	LOC100046	12.45598333	11.99356316	11.99824265	12.05897727
ILMN_2660596	RUNDC3B	6.71609	7.178071053	7.1822	7.121272727
ILMN_2431619	UBE2L6	8.088856667	8.550692105	8.561057353	8.579322727
ILMN_2688075	CYP51	9.241013333	9.702565789	9.707444118	9.776145455
ILMN_1255256	SGCB	6.903226667	7.364521053	7.292351471	7.317954545
ILMN_3133748	GAB3	7.71051	7.249381579	7.207739706	7.064881818
ILMN_2769567	F2RL1	6.789253333	6.328434211	6.311585294	6.318131818
ILMN_2927131	IL13	6.278206667	6.738778947	6.699602941	6.664881818
ILMN_2889832	SERPINA3H	7.487796667	7.948363158	7.94995	8.069
ILMN_2793946	CSNK1G3	10.25665333	9.796518421	9.729845588	9.704345455
ILMN_2770917	BLVRB	8.54669	9.006694737	9.003377941	8.950313636
ILMN_2631259	IGK-V5	11.67123	11.21130789	11.21311029	11.28002727
ILMN_2784272	IFNGR2	10.17693333	9.717152632	9.806033824	9.636736364
ILMN_2467365	PEX11C	6.63978	6.1805	6.161683824	6.187445455
ILMN_3093089	TARDBP	8.83852	8.379476316	8.366308824	8.351659091
ILMN_2599018	CLIC4	6.676103333	7.134968421	7.206091176	7.233940909
ILMN_2772920	FBXO4	9.511573333	9.970065789	9.933588235	9.823972727
ILMN_2814847	SCN4A	6.732753333	7.191044737	7.299491176	7.273713636
ILMN_1240153	UCHL5	8.30777	8.765928947	8.739652941	8.759190909
ILMN_1250135	A930005H1	10.44449	9.986555263	10.03557794	9.894318182
ILMN_2587761	KIF1B	6.898756667	6.440939474	6.393510294	6.337227273
ILMN_3026397	CHKA	12.51251667	12.05482368	12.07835147	11.87520909
ILMN_2888448	CCR6	11.8719	11.41442368	11.42346618	11.433
ILMN_1258600	LOC100043	9.537613333	9.99505	9.958407353	10.02151818
ILMN_2917180	FOXP3	7.212343333	7.669513158	7.709433824	7.612072727
ILMN_1248891	CRTC3	8.721226667	8.2641	8.262083824	8.305422727
ILMN_2612448	NFAT5	10.1261	9.669371053	9.617407353	9.650840909
ILMN_3149143	ENTPD5	7.866756667	7.41035	7.433564706	7.419763636
ILMN_1248824	C230082I21	9.24562	8.789247368	8.743510294	8.73275
ILMN_1229263	LOC100046	10.91076	10.45446316	10.498725	10.60142727
ILMN_2677876	ARHGAP4	11.83005333	11.37396842	11.37664706	11.29995909
ILMN_3118584	BEX4	6.290316667	6.745934211	6.717019118	6.743859091
ILMN_2469190	B230345P0	9.48775	9.032318421	8.997217647	8.814136364
ILMN_2753149	AVIL	5.968846667	6.424028947	6.406513235	6.338922727
ILMN_2514723	1110067B1	8.664656667	8.209513158	8.227560294	8.173459091
ILMN_1239601	LOC637711	7.591996667	8.047021053	8.053213235	8.032568182
ILMN_2686132	ADA	7.039233333	7.494160526	7.524789706	7.525754545
ILMN_1235493	FXYD5	10.50235333	10.04759737	10.05027206	9.985063636
ILMN_1244857	RABAC1	11.18026333	10.72565526	10.79111324	10.75775455
ILMN_1257965	LDHA	12.56264333	13.01716842	12.98936765	12.88280455
ILMN_2597030	SMOX	7.755666667	8.210173684	8.189514706	8.309086364
ILMN_1241923	MSH5	7.286276667	6.83185	6.818532353	6.777359091
ILMN_1239607	DGKZ	8.896886667	8.442460526	8.459808824	8.481786364
ILMN_2522495	C130078N1	9.92795	10.38224737	10.34155441	10.21982727
ILMN_1232144	A130087I02	6.94611	6.491957895	6.482135294	6.369822727
ILMN_2695217	2810485I05	10.85201	10.39787632	10.37109853	10.27317273
ILMN_1249864	A630077B1	8.645473333	9.099492105	9.099207353	8.909022727
ILMN_1221943	SDF2L1	9.424183333	9.878152632	9.842982353	10.00285909

ILMN_1218934	RDM1	9.691513333	9.237818421	9.296355882	9.263672727
ILMN_1225932	CCND2	7.983533333	8.437152632	8.424633824	8.339681818
ILMN_2538029	LOC386005	11.26105	10.80766316	10.76714265	10.68383636
ILMN_2571616	C430002D1	8.354466667	7.901107895	7.951077941	7.801709091
ILMN_2891506	TBCD	9.07411	9.527063158	9.515958824	9.499972727
ILMN_2516348	VAMP4	8.802203333	8.349344737	8.311704412	8.185272727
ILMN_2624938	PEA15	8.426816667	7.973960526	8.031760294	7.905472727
ILMN_2484838	UBAC2	9.095233333	8.642694737	8.613807353	8.500886364
ILMN_2673369	IRF8	10.46650667	10.91894737	10.92003235	10.8755
ILMN_2663230	SLCO3A1	7.528683333	7.980968421	8.006858824	7.821268182
ILMN_1213645	AI467606	10.8157	10.36356316	10.381575	10.31484545
ILMN_1224390	1700129I04	6.254066667	6.706015789	6.752007353	6.905109091
ILMN_2672091	HTT	7.075923333	7.527834211	7.447980882	7.573559091
ILMN_2705097	DEADC1	8.784596667	9.236284211	9.246120588	9.286304545
ILMN_2417863	TNIP1	10.67977667	10.22862895	10.33654118	10.3292
ILMN_2630605	FSCN1	9.264746667	8.813610526	8.879035294	9.026695455
ILMN_2710229	CCNG1	8.237643333	8.688307895	8.719710294	8.740531818
ILMN_2602597	SH3RF1	6.97383	7.424310526	7.302129412	7.306981818
ILMN_1227018	IL1A	7.508863333	7.959210526	8.102714706	8.221045455
ILMN_2727235	ANKRD11	10.3901	9.939768421	9.97785	9.793654545
ILMN_2759484	C3	8.713386667	8.263144737	8.351541176	8.556345455
ILMN_2814484	IFITM1	6.603923333	7.054015789	7.066145588	7.122472727
ILMN_2526739	BATF3	6.226883333	6.676947368	6.730197059	6.660731818
ILMN_1215825	LOC100047	9.10279	8.652736842	8.764692647	8.732336364
ILMN_1219978	APPL2	8.213966667	7.764534211	7.741592647	7.672322727
ILMN_2751948	HIST2H2AA	7.52977	7.080352632	7.127307353	7.225190909
ILMN_1225769	CLASP1	8.361373333	7.912028947	7.920219118	7.866618182
ILMN_1218547	DLM1-PEND	6.341386667	6.79065	6.869138235	6.909118182
ILMN_2974737	BC065085	7.958643333	7.509571053	7.581791176	7.586068182
ILMN_2655571	B3GNT7	6.033913333	6.482823684	6.563373529	6.687959091
ILMN_2585533	LY78	8.423283333	7.974515789	7.950780882	7.901995455
ILMN_3138439	DYRK2	7.370266667	6.921794737	6.905360294	6.828077273
ILMN_2524861	CHD3	8.387586667	7.939173684	7.977804412	7.910968182
ILMN_2995688	EG433016	9.116706667	8.66845	8.852829412	9.032322727
ILMN_3004142	STK4	11.51383	11.06564474	11.06916765	11.10165
ILMN_1217855	NKG7	9.221053333	9.669168421	9.689545588	9.423554545
ILMN_2543834	RFC3	6.905386667	7.353431579	7.309782353	7.283140909
ILMN_1236304	HAMP	5.92571	6.373726316	6.455057353	6.366813636
ILMN_1223697	CD44	9.625953333	10.07376842	10.11732941	10.10371364
ILMN_2700848	ARRB2	9.449236667	9.001426316	9.048977941	9.081027273
ILMN_2949605	UBAC2	10.55435	10.10654737	10.07797647	9.933981818
ILMN_2743503	E2F3	7.260286667	7.707805263	7.726333824	7.720931818
ILMN_2546073	WDR68	7.13092	7.577952632	7.545673529	7.717172727
ILMN_2595973	GRN	11.08555667	10.63854737	10.66243382	10.77950909
