# Supplementary material for: Comparing the Biological Impact of Glatiramer Acetate with the Biological Impact of a Generic
Source: PLoS One. 2014 Jan 8;9(1):e83757. doi: 10.1371/journal.pone.0083757 (PMC3885444; doi:10.1371/journal.pone.0083757)
Supplement: Table S3 — The highly variable probes that were significant by F-test in either GA or generic (see methods section) and are depicted in Figure 1A . (PDF) [file pone.0083757.s011.pdf]

| TABLE S3 | | |
|---|---|---|
| Type | Probe | Gene |
| More variable in generic | ILMN_2927369 | KRT82 |
| More variable in generic | ILMN_1248780 | SPRR2B |
| More variable in generic | ILMN_1255612 | 9430016C22RIK |
| More variable in generic | ILMN_2465182 | XLR3B |
| More variable in generic | ILMN_1220648 | A630014I05RIK |
| More variable in generic | ILMN_2441895 | TYMS |
| More variable in generic | ILMN_1215680 | SAMD3 |
| More variable in generic | ILMN_2662283 | 2010007H12RIK |
| More variable in generic | ILMN_2730829 | TMUB1 |
| More variable in generic | ILMN_2534501 | LOC380738 |
| More variable in generic | ILMN_1229286 | 2410042D21RIK |
| More variable in generic | ILMN_2511527 | 9330133O14RIK |
| More variable in generic | ILMN_1228867 | A130082M07RIK |
| More variable in generic | ILMN_3009928 | 4930524E20RIK |
| More variable in generic | ILMN_2870788 | UPB1 |
| More variable in generic | ILMN_2941062 | SHROOM2 |
| More variable in generic | ILMN_2444847 | 4930521G14RIK |
| More variable in generic | ILMN_2805419 | OLFR885 |
| More variable in generic | ILMN_2825562 | CYBRD1 |
| More variable in generic | ILMN_2456193 | 6446580_669 |
| More variable in generic | ILMN_1225456 | CHRNA7 |
| More variable in generic | ILMN_2621217 | 5730508B09RIK |
| More variable in generic | ILMN_2495555 | MAPK8IP2 |
| More variable in generic | ILMN_2653725 | LOC100046802 |
| More variable in generic | ILMN_3161601 | SNCA |
| More variable in generic | ILMN_2436890 | DHRSX |
| More variable in generic | ILMN_1225663 | LOC384162 |
| More variable in generic | ILMN_1222683 | OLFR1441 |
| More variable in generic | ILMN_2691752 | OTX2 |
| More variable in generic | ILMN_2460292 | 8430427H17RIK |
| More variable in generic | ILMN_2706906 | LOC100047937 |
| More variable in generic | ILMN_2820893 | SELPLG |
| More variable in generic | ILMN_1233075 | ERCC2 |
| More variable in generic | ILMN_2733594 | D630004A14RIK |
| More variable in generic | ILMN_2768252 | MYL7 |
| More variable in generic | ILMN_1233191 | 5730422E09RIK |
| More variable in generic | ILMN_2935462 | SCN2B |
| More variable in generic | ILMN_2541335 | LOC384887 |
| More variable in generic | ILMN_2626648 | 41522 |
| More variable in generic | ILMN_2494532 | NRG1 |
| More variable in generic | ILMN_2682263 | KLHL28 |
| More variable in generic | ILMN_1378821 | control_ILMN_1378821 |
| More variable in generic | ILMN_1245775 | FUT9 |
| More variable in generic | ILMN_2752524 | PAQR9 |
| More variable in generic | ILMN_1234848 | TRIM2 |
| More variable in generic | ILMN_2564352 | SDHA |

| | | |
|---|---|---|
| More variable in generic | ILMN_1260478 | HECW1 |
| More variable in generic | ILMN_2620284 | PHLDB1 |
| More variable in generic | ILMN_2741169 | CD8B1 |
| More variable in generic | ILMN_2728928 | FABP1 |
| More variable in generic | ILMN_2690135 | ACSL4 |
| More variable in generic | ILMN_2530757 | LOC210143 |
| More variable in generic | ILMN_1223894 | E530018B05RIK |
| More variable in generic | ILMN_2654541 | MED19 |
| More variable in generic | ILMN_2769762 | CTNNA2 |
| More variable in generic | ILMN_1378704 | control_ILMN_1378704 |
| More variable in generic | ILMN_1259595 | AI595406 |
| More variable in generic | ILMN_2635132 | FOXP3 |
| More variable in generic | ILMN_1250340 | A630055A13RIK |
| More variable in generic | ILMN_2524263 | ZFP251 |
| More variable in generic | ILMN_2690574 | FOXO3 |
| More variable in generic | ILMN_1239070 | MYO18B |
| More variable in generic | ILMN_2580818 | D930042N17RIK |
| More variable in generic | ILMN_2697415 | CD3D |
| More variable in generic | ILMN_1228620 | ZFP583 |
| More variable in generic | ILMN_2502290 | TRIM71 |
| More variable in generic | ILMN_2642321 | CEACAM13 |
| More variable in generic | ILMN_2661820 | AGXT2L1 |
| More variable in generic | ILMN_1244840 | 4833435K08RIK |
| More variable in generic | ILMN_1233941 | UNCX |
| More variable in generic | ILMN_1233843 | PTPN21 |
| More variable in generic | ILMN_1224922 | LRRN2 |
| More variable in generic | ILMN_2737523 | F2RL3 |
| More variable in generic | ILMN_2637897 | NLRX1 |
| More variable in generic | ILMN_1254631 | AI481316 |
| More variable in generic | ILMN_2498330 | LOC632997 |
| More variable in generic | ILMN_2769330 | CD6 |
| More variable in generic | ILMN_2666330 | OIT3 |
| More variable in generic | ILMN_2527328 | LOC381360 |
| More variable in generic | ILMN_2883666 | PIGN |
| More variable in generic | ILMN_1257579 | NUP210 |
| More variable in generic | ILMN_1235441 | STK22S1 |
| More variable in generic | ILMN_2769325 | CD6 |
| More variable in generic | ILMN_3006990 | EG622339 |
| More variable in generic | ILMN_1216552 | CORO2B |
| More variable in generic | ILMN_2701383 | ANKS3 |
| More variable in generic | ILMN_1247936 | OLFR571 |
| More variable in generic | ILMN_2998230 | GALNS |
| More variable in generic | ILMN_2610965 | DAB2IP |
| More variable in generic | ILMN_1223081 | LOC241962 |
| More variable in generic | ILMN_2598271 | ASB17 |
| More variable in generic | ILMN_2979729 | OLFR1287 |
| More variable in generic | ILMN_1245710 | CCL2 |
| More variable in generic | ILMN_2705689 | GGA2 |

| | | |
|---|---|---|
| More variable in generic | ILMN_1242912 | POF1B |
| More variable in generic | ILMN_2445249 | KRT36 |
| More variable in generic | ILMN_1235485 | LOC218501 |
| More variable in generic | ILMN_2964420 | LIPK |
| More variable in generic | ILMN_3104704 | KLRA4 |
| More variable in generic | ILMN_1246768 | A830026B15RIK |
| More variable in generic | ILMN_2923717 | KCNT1 |
| More variable in generic | ILMN_2798167 | BB287469 |
| More variable in generic | ILMN_1246408 | LOC100042427 |
| More variable in generic | ILMN_2749437 | RHOC |
| More variable in generic | ILMN_1243092 | EG546980 |
| More variable in GA | ILMN_1222230 | MEPE |
| More variable in GA | ILMN_1237927 | 8430407G10RIK |
| More variable in GA | ILMN_1380391 | control_ILMN_1380391 |
| More variable in GA | ILMN_1222029 | RTTN |
| More variable in GA | ILMN_1256073 | PXDN |
| More variable in GA | ILMN_1221957 | LOC637776 |
| More variable in GA | ILMN_1257904 | 2810403A07RIK |
| More variable in GA | ILMN_1244123 | SLC38A2 |
| More variable in GA | ILMN_2529810 | GPR113 |
| More variable in GA | ILMN_1231281 | LOC382900 |
| More variable in GA | ILMN_2756771 | GMFB |
| More variable in GA | ILMN_1260480 | 5033411B22RIK |
| More variable in GA | ILMN_1258114 | A930014P08RIK |
| More variable in GA | ILMN_2524553 | 4930555L11RIK |
| More variable in GA | ILMN_2701797 | MEN1 |
| More variable in GA | ILMN_2499058 | UBE2I |
| More variable in GA | ILMN_2759380 | 2310010M20RIK |
| More variable in GA | ILMN_1244585 | RAP1GAP |
| More variable in GA | ILMN_2666205 | DPP10 |
| More variable in GA | ILMN_1240784 | LOC384938 |
| More variable in GA | 1770446 | 1770446 |
| More variable in GA | ILMN_1252393 | LOC635340 |
| More variable in GA | ILMN_2700224 | KTN1 |
| More variable in GA | ILMN_1217225 | CCDC91 |
