# Supplementary material for: Comparing the Biological Impact of Glatiramer Acetate with the Biological Impact of a Generic
Source: PLoS One. 2014 Jan 8;9(1):e83757. doi: 10.1371/journal.pone.0083757 (PMC3885444; doi:10.1371/journal.pone.0083757)
Supplement: Table S4 — Ranked list of probes by ratio of the variance in generic-activated samples to the variance in GA-activated samples. (PDF) [file pone.0083757.s012.pdf]

TABLE S4		
Probe	Gene	Var(generic)/Var(GA)
ILMN_2635132	FOXP3	4.169784637
ILMN_2707941	GPR83	4.141645173
ILMN_2752702	KLHL12	3.56612757
ILMN_2741169	CD8B1	3.455712087
ILMN_2783997	TRIM10	3.370881625
ILMN_3161601	SNCA	3.340589295
ILMN_2601946	5033414K04RIK	3.297834654
ILMN_1228867	A130082M07RIK	3.157262632
ILMN_3136638	SNCA	3.14591837
ILMN_2895284	2210023G05RIK	3.126076675
ILMN_2886128	PCDHA10	3.085624289
ILMN_1257368	GM566	3.073208924
ILMN_2635718	BZRPL1	3.059590672
ILMN_1239724	PSMB1	3.031738703
ILMN_1256142	MARCKS	3.022926531
ILMN_2690574	FOXO3	2.972307993
ILMN_1258520	4933421E18RIK	2.903836286
ILMN_2703061	2810408P10RIK	2.867474892
ILMN_2824625	GM1604	2.825719679
ILMN_1233843	PTPN21	2.825301578
ILMN_2620284	PHLDB1	2.814372655
ILMN_2745073	LOC100047353	2.809357296
ILMN_2749437	RHOC	2.773049302
ILMN_1248780	SPRR2B	2.770363507
ILMN_2703079	PUNC	2.754789803
ILMN_1255719	A930004L03RIK	2.735701046
ILMN_2733594	D630004A14RIK	2.712208469
ILMN_1254631	AI481316	2.710449678
ILMN_2939277	SNCG	2.703626953
ILMN_1225059	SLC16A12	2.697244381
ILMN_1217855	NKG7	2.690910757
ILMN_2753697	CD2	2.674585226
ILMN_1255513	CDR2	2.671217726
ILMN_2965737	KLHL34	2.670969821
ILMN_2524992	EAR2	2.664017605
ILMN_2498330	LOC632997	2.64101161
ILMN_1217629	ITGAE	2.640617288
ILMN_2626648	41522	2.611392387
ILMN_1215949	1700040F17RIK	2.595459871
ILMN_3114124	HSD3B7	2.594406596
ILMN_1216823	UBR5	2.584859171
ILMN_1222683	OLFR1441	2.577789931
ILMN_2606162	PDLIM4	2.57565855
ILMN_1223081	LOC241962	2.54822461
ILMN_2435477	5830406C15RIK	2.537539864
ILMN_2686825	5430427O19RIK	2.536086924

ILMN_1222512	MFSD7B	2.525669655
ILMN_1248854	ICK	2.525643685
ILMN_2705689	GGA2	2.522100556
ILMN_2826881	MYBL2	2.522004768
ILMN_1220648	A630014I05RIK	2.518845526
ILMN_1378704	control_ILMN_13	2.501520902
ILMN_2715400	TEF	2.495018969
ILMN_1257579	NUP210	2.490045841
ILMN_2598271	ASB17	2.490015142
ILMN_1225557	E030019B06RIK	2.484827893
ILMN_2644350	THY1	2.476638172
ILMN_1216972	CLEC4E	2.47211937
ILMN_2679094	MAP3K7IP2	2.471543957
ILMN_2820893	SELPLG	2.462368133
ILMN_1223480	D530031C13RIK	2.461106681
ILMN_2495703	CLIP2	2.449801678
ILMN_2679591	ERAP1	2.449203035
ILMN_2465182	XLR3B	2.447786159
ILMN_1219011	3110040M04RIK	2.445802442
ILMN_2870696	HFE	2.434961339
ILMN_3005873	SORT1	2.431402316
ILMN_2515285	WASF3	2.431071739
ILMN_2495555	MAPK8IP2	2.429611427
ILMN_2539917	LOC384538	2.421951597
ILMN_2757838	IPPK	2.416554447
ILMN_3162301	OTTMUSG000000	2.414336977
ILMN_2923717	KCNT1	2.414048507
ILMN_2725054	A930006J02RIK	2.409634421
ILMN_1230224	DNAJC6	2.408553983
ILMN_1225663	LOC384162	2.408249488
ILMN_1230818	9630015E22RIK	2.406694567
ILMN_2539295	LOC621968	2.406675656
ILMN_2950828	C030018G13RIK	2.405823034
ILMN_2606792	GBP4	2.404718824
ILMN_1226665	ISLR	2.404321761
ILMN_2769325	CD6	2.398106015
ILMN_1228696	ADA	2.39687535
ILMN_2670368	CAR5B	2.393795851
ILMN_2658878	TG	2.392922859
ILMN_1242548	HIF1A	2.389939819
ILMN_1254380	DBR1	2.386871534
ILMN_2446592	RAD18	2.378012721
ILMN_2425143	MJ-500-31_190	2.377329303
ILMN_2769330	CD6	2.373941676
ILMN_3155363	PPL	2.37383497
ILMN_2454209	TRBV6_AE000663	2.372090892
ILMN_1235584	PLEKHA5	2.371229664
ILMN_1218694	C630017J20RIK	2.370593123

ILMN_2859348	TAF9B	2.367681107
ILMN_1231458	IQGAP1	2.366074862
ILMN_2426480	3830612M24	2.365830273
ILMN_2664439	9230109A22RIK	2.3624709
ILMN_2498851	2310075M15RIK	2.359962418
