# Supplementary material for: Comparing the Biological Impact of Glatiramer Acetate with the Biological Impact of a Generic
Source: PLoS One. 2014 Jan 8;9(1):e83757. doi: 10.1371/journal.pone.0083757 (PMC3885444; doi:10.1371/journal.pone.0083757)
Supplement: Table S5 — Comparison of expression in GA to expression in GA for each probe, including fold change, ANOVA, LIMMA with background subtraction, comparative marker selection by signal-to-noise ratio, comparative marker selection by t-test, and the Wilcoxon non-parametric method. (PDF) [file pone.0083757.s013.pdf]

TABLE S5													
			Comparative Marker Selection								Significant in Parametric Tests		
			T-test		SNR		LIMMA		ANOVA			Wilcoxon Rank Sum	
Probe	Gene	Fold Change	Nom. P.	Adj. P.	Nom. P.	Adj. P.	Nom. P.	Adj. P.	Nom. P.	Adj. P.	1-yes,0-no	Nom. P.	Adj. P.
ILMN_2685712	IFNG	1.41	0.0002995	0.01489206	0.0002995	0.01335371	2.36E-07	0.0000718	4.01E-07	0.000139	1	2.06483E-05	0.00430994
ILMN_2595857	ANKRD37	1.36	0.0002995	0.01489206	0.0002995	0.01335371	5.82E-12	1.69E-08	1.23E-08	0.0000119	1	6.03547E-07	0.00057333
ILMN_2791459	IFNG	1.33	0.0002995	0.01489206	0.0002995	0.01335371	0.00000199	0.00037564	0.00000642	0.00108	1	0.000197175	0.01547711
ILMN_2675232	KLK8	1.284884601	0.0002995	0.01489206	0.0002995	0.01335371	7.43E-19	3.46E-14	5.6E-14	2.61E-09	1	7.95833E-09	0.00012222
ILMN_1220788	GM590	1.284311292	0.0002995	0.01489206	0.0002995	0.01335371	3.92E-10	0.00000057	4.06E-09	0.00000573	1	4.9657E-07	0.00052531
ILMN_1253828	CD247	1.280944226	0.0002995	0.01489206	0.0002995	0.01335371	7.18E-09	0.00000514	1.89E-07	0.0000836	1	1.53585E-05	0.00359242
ILMN_2856926	GPR114	1.275591039	0.0002995	0.01489206	0.0002995	0.01335371	3.75E-15	5.82E-11	5.66E-11	0.000000293	1	5.89499E-08	0.00024945
ILMN_1218037	TMIE	1.25825101	0.0002995	0.01489206	0.0002995	0.01335371	1.6E-10	2.67E-07	8.95E-09	0.0000105	1	1.80477E-06	0.001091
ILMN_2664929	CD8B1	1.257683544	0.0002995	0.01489206	0.0002995	0.01335371	6.15E-13	2.6E-09	8E-11	0.000000367	1	1.49655E-07	0.00034541
ILMN_1240428	SATB1	1.253362369	0.0002995	0.01489206	0.0002995	0.01335371	3.36E-10	5.04E-07	3.35E-08	0.0000244	1	2.88347E-07	0.00041943
ILMN_2741169	CD8B1	1.24933849	0.0002995	0.01489206	0.0002995	0.01335371	2.32E-15	5.4E-11	3.06E-12	4.75E-08	1	1.57497E-07	0.00034541
ILMN_1250001	4930583H14RIK	1.24042799	0.00094262	0.02607154	0.0006399	0.01941092	0.00000259	0.00046493	0.000033	0.003242217	1	0.000130304	0.01220373
ILMN_2611022	BCL11B	1.239019043	0.0002995	0.01489206	0.0002995	0.01335371	7.52E-10	9.15E-07	0.00000014	0.0000707	1	1.5356E-05	0.00359242
ILMN_2707941	GPR83	1.238234714	0.0002995	0.01489206	0.0002995	0.01335371	9.31E-14	8.67E-10	2.22E-12	4.75E-08	1	1.65715E-07	0.00034541
ILMN_2769772	PIK3IP1	1.235639618	0.0002995	0.01489206	0.0002995	0.01335371	8.08E-12	2.21E-08	2.26E-09	0.00000363	1	4.9657E-07	0.00052531
ILMN_2775055	LOC669166	1.234427524	0.0002995	0.01489206	0.0002995	0.01335371	3.92E-12	1.4E-08	2.69E-09	0.00000405	1	4.7283E-07	0.00052531
ILMN_3128992	CD27	1.229968408	0.0002995	0.01489206	0.0002995	0.01335371	5.34E-13	2.48E-09	8.67E-11	0.000000367	1	1.10038E-07	0.00029962
ILMN_2416460	TRBV8_AE000663_	1.227075821	0.0002995	0.01489206	0.0002995	0.01335371	5.05E-12	1.57E-08	2.14E-09	0.00000357	1	2.49646E-06	0.00133566
ILMN_2670150	OAS2	1.223676484	0.0002995	0.01489206	0.0002995	0.01335371	1.69E-08	0.0000104	2.75E-07	0.000109783	1	1.80477E-06	0.001091
ILMN_2454209	TRBV6_AE000663_	1.223323464	0.0002995	0.01489206	0.0002995	0.01335371	4.23E-09	0.00000323	0.00000012	0.0000626	1	0.000103509	0.01061242
ILMN_2960700	PRF1	1.220037267	0.0002995	0.01489206	0.0002995	0.01335371	0.00000023	0.0000712	0.00000418	0.000771264	1	3.99889E-05	0.00618393
ILMN_1229318	PDK1	1.219922378	0.0002995	0.01489206	0.0002995	0.01335371	1.26E-09	0.00000128	5.7E-08	0.0000353	1	1.98091E-06	0.00115257
ILMN_1233589	CD27	1.219317837	0.0002995	0.01489206	0.0002995	0.01335371	3.67E-13	1.9E-09	1.77E-11	0.000000121	1	2.95621E-08	0.00022934
ILMN_2678521	GPR146	1.215770831	0.0002995	0.01489206	0.0002995	0.01335371	1.33E-13	8.87E-10	3.83E-11	0.000000223	1	7.26855E-08	0.00026025
ILMN_1256701	2900016B01RIK	1.215770789	0.0002995	0.01489206	0.0002995	0.01335371	2.75E-08	0.0000147	4.48E-07	0.000148861	1	2.54447E-05	0.00475651
ILMN_1215167	DDX5	1.214758445	0.0006399	0.0211535	0.0006399	0.01941092	0.00010165	0.00670197	0.0000543	0.004539402	1	7.0054E-05	0.00846962
ILMN_3145331	TMSB10	1.209579271	0.0002995	0.01489206	0.0002995	0.01335371	4.98E-12	1.57E-08	1.59E-10	0.000000461	1	2.13433E-07	0.00036795
ILMN_1252200	A830055I09RIK	1.206497974	0.0002995	0.01489206	0.0002995	0.01335371	0.00000761	0.0010354	0.0000334	0.003276709	1	5.74504E-05	0.00761864
ILMN_2819380	BC030476	1.206275831	0.0002995	0.01489206	0.0002995	0.01335371	0.000036	0.00314503	0.00012566	0.008308344	1	0.000197175	0.01547711
ILMN_2932359	TPI1	1.204626968	0.0002995	0.01489206	0.0002995	0.01335371	5.08E-08	0.0000227	0.0000011	0.000288061	1	1.53585E-05	0.00359242
ILMN_2837493	EG634650	1.204245484	0.0002995	0.01489206	0.0002995	0.01335371	5.69E-08	0.0000248	5.02E-08	0.0000324	1	4.9657E-07	0.00052531
ILMN_1230137	TPI1	1.204175926	0.0002995	0.01489206	0.0002995	0.01335371	2.06E-08	0.0000124	0.00000121	0.000298774	1	2.88127E-05	0.00509941
ILMN_2944666	IFIT3	1.202746675	0.0002995	0.01489206	0.0002995	0.01335371	5.52E-08	0.0000242	2.84E-08	0.0000217	1	2.74304E-07	0.00041187
ILMN_1217855	NKG7	1.202473931	0.0006399	0.0211535	0.0006399	0.01941092	2.84E-08	0.0000149	5.31E-07	0.000169129	1	0.000264806	0.01861919
ILMN_2699898	ITGAE	1.201910561	0.0002995	0.01489206	0.0002995	0.01335371	1.42E-11	3.32E-08	1.38E-10	0.000000429	1	1.04521E-07	0.00029962
ILMN_2516221	TCRB-V8.2	1.20121668	0.0002995	0.01489206	0.0002995	0.01335371	1.11E-08	0.00000752	4.35E-07	0.000147963	1	1.13766E-05	0.00307877
ILMN_2652867	TRAT1	1.199470243	0.0002995	0.01489206	0.0002995	0.01335371	6.33E-10	8.19E-07	3.26E-08	0.0000241	1	4.12145E-06	0.00171287
ILMN_1229197	OTTMUSG000000	1.198389483	0.0002995	0.01489206	0.0002995	0.01335371	4.13E-08	0.00002	1.88E-08	0.0000159	1	9.77116E-07	0.00079793
ILMN_2618148	C330008K14RIK	1.196159524	0.0002995	0.01489206	0.0002995	0.01335371	2.81E-11	5.95E-08	1.46E-08	0.000013	1	1.18196E-06	0.00088737
ILMN_1214841	IL24	1.195664917	0.00094262	0.02607154	0.00094262	0.02430167	0.0000373	0.00322909	0.00014082	0.009031912	1	0.002173782	0.0607706

ILMN_1257496	LOC665446	1.195420235	0.0002995	0.01489206	0.0002995	0.01335371	8.57E-08	0.0000344	0.00000185	0.000412402	1	1.47177E-05	0.00353126
ILMN_2421246	TCRB-V8.3	1.195307771	0.0002995	0.01489206	0.0002995	0.01335371	1.42E-09	0.00000136	4.63E-08	0.0000308	1	5.89742E-06	0.0021116
ILMN_2729252	5830431A10RIK	1.195292385	0.0002995	0.01489206	0.0002995	0.01335371	1.38E-09	0.00000136	2.06E-08	0.0000171	1	1.2393E-06	0.00090134
ILMN_2484932	C030002B11RIK	1.193361778	0.0002995	0.01489206	0.0002995	0.01335371	6.33E-10	8.19E-07	5.47E-08	0.0000344	1	8.06586E-07	0.00070845
ILMN_1221819	FCGRT	1.193070246	0.0002995	0.01489206	0.0002995	0.01335371	7.12E-09	0.00000514	2.33E-08	0.0000187	1	9.56517E-06	0.00274856
ILMN_2981167	IFIT2	1.192834198	0.0002995	0.01489206	0.0002995	0.01335371	2.31E-08	0.000013	1.07E-08	0.0000115	1	3.88359E-07	0.00048857
ILMN_2585233	SELPL	1.192768508	0.0002995	0.01489206	0.0002995	0.01335371	8.22E-10	9.34E-07	8.63E-08	0.0000485	1	1.41024E-05	0.00347315
ILMN_2697415	CD3D	1.191227881	0.0002995	0.01489206	0.0002995	0.01335371	5.84E-10	7.99E-07	3.71E-08	0.0000262	1	5.64085E-06	0.00208385
ILMN_1230345	B830007D08RIK	1.191119956	0.0002995	0.01489206	0.0002995	0.01335371	3.22E-09	0.00000253	1.09E-08	0.0000115	1	1.57497E-07	0.00034541
ILMN_2811263	ZXDA	1.190267135	0.0002995	0.01489206	0.0006399	0.01941092	0.0000175	0.00184283	0.00015367	0.009469751	1	0.000176289	0.01444673
ILMN_2560567	A630006E02RIK	1.189860092	0.0002995	0.01489206	0.0002995	0.01335371	0.0000009	0.00020543	0.0000211	0.002464033	1	9.21443E-05	0.00995203
ILMN_2820893	SELPLG	1.188849449	0.0002995	0.01489206	0.0002995	0.01335371	1.27E-13	8.87E-10	8.13E-12	7.57E-08	1	5.59305E-08	0.00024945
ILMN_1246609	RASGRP1	1.18775349	0.0002995	0.01489206	0.0002995	0.01335371	3.02E-11	6.12E-08	2.46E-09	0.00000382	1	3.34766E-07	0.00044521
ILMN_2660551	LAT	1.187218377	0.0002995	0.01489206	0.0002995	0.01335371	9.43E-07	0.00021216	0.00000642	0.001075031	1	0.000130304	0.01220373
ILMN_2507761	7530404M11RIK	1.186381811	0.0002995	0.01489206	0.0002995	0.01335371	0.0000307	0.00278714	0.0000826	0.006091257	1	9.21504E-05	0.00995203
ILMN_2753697	CD2	1.186334291	0.0002995	0.01489206	0.0002995	0.01335371	2.97E-13	1.73E-09	1.04E-10	0.000000395	1	1.92936E-07	0.00034541
ILMN_2601453	A130092J06RIK	1.186313516	0.0002995	0.01489206	0.0002995	0.01335371	3.23E-11	6.26E-08	9.08E-10	0.00000192	1	9.77016E-07	0.00079793
ILMN_1234565	P4HA1	1.18626686	0.0002995	0.01489206	0.0002995	0.01335371	1.07E-07	0.0000412	7.49E-07	0.000219513	1	8.39227E-06	0.00252023
ILMN_2459899	ADAMTSL4	1.185956519	0.0002995	0.01489206	0.0002995	0.01335371	2.09E-09	0.00000174	2.06E-07	0.0000903	1	3.93982E-06	0.00166715
ILMN_2761720	LOC100041103	1.185737904	0.0002995	0.01489206	0.0002995	0.01335371	1.19E-09	0.00000123	3.43E-08	0.0000245	1	9.77016E-07	0.00079793
ILMN_1213954	SGK1	1.185317215	0.0002995	0.01489206	0.0002995	0.01335371	1.06E-07	0.000041	3.55E-07	0.000132883	1	1.49642E-06	0.00102442
ILMN_2632971	B3GALT4	1.185249067	0.0002995	0.01489206	0.0002995	0.01335371	1.78E-09	0.00000151	2.34E-10	0.000000605	1	6.36427E-09	0.00012222
ILMN_2860649	GBP6	1.184770963	0.0002995	0.01489206	0.0002995	0.01335371	0.00000014	0.0000492	1.01E-07	0.0000545	1	1.72245E-06	0.00108344
ILMN_2722784	CD3G	1.184582149	0.0002995	0.01489206	0.0002995	0.01335371	4.91E-08	0.0000224	0.00000016	0.0000754	1	2.06467E-05	0.00430994
ILMN_2667829	PRKCQ	1.183849443	0.0002995	0.01489206	0.0002995	0.01335371	2.77E-11	5.95E-08	1.89E-10	0.000000518	1	8.49731E-08	0.00028252
ILMN_1235499	PROS1	1.183674675	0.0002995	0.01489206	0.0002995	0.01335371	4.45E-07	0.00012175	0.00000119	0.00029799	1	2.24533E-05	0.00440984
ILMN_2694955	IGFBP4	1.183278575	0.0002995	0.01489206	0.0002995	0.01335371	0.00000158	0.00031636	0.00000706	0.001150107	1	9.95836E-05	0.01046347
ILMN_2491445	LOC639001	1.183039504	0.0006399	0.0211535	0.0002995	0.01335371	2.23E-08	0.0000126	4.29E-07	0.000146987	1	4.70154E-05	0.00688184
ILMN_2661185	SCML4	1.18284704	0.0002995	0.01489206	0.0002995	0.01335371	0.00000016	0.0000542	0.0000016	0.000369569	1	1.81978E-05	0.00399552
ILMN_2454823	6720418B01RIK	1.182721445	0.0002995	0.01489206	0.0002995	0.01335371	0.0000166	0.00178634	0.00013436	0.00874694	1	0.00057968	0.02968357
ILMN_2947526	ECM1	1.181777956	0.0002995	0.01489206	0.0002995	0.01335371	0.00000016	0.0000542	7.57E-07	0.000219513	1	7.35697E-06	0.00232956
ILMN_2705407	PABPC1	1.181328619	0.00094262	0.02607154	0.0006399	0.01941092	0.00011396	0.00731485	0.00024603	0.012809759	1	0.000329201	0.02128238
ILMN_1227570	LOC386545	1.180241912	0.0006399	0.0211535	0.0002995	0.01335371	1.36E-07	0.0000491	0.00000102	0.000272691	1	5.30373E-05	0.00748099
ILMN_2475156	XIST	1.179666178	0.0006399	0.0211535	0.0006399	0.01941092	0.00036367	0.01638681	0.00063489	0.023714186	1	0.001104095	0.04229821
ILMN_1214650	SCL0002368.1_75	1.179443236	0.0002995	0.01489206	0.0002995	0.01335371	0.0000372	0.00322909	0.0000222	0.00254822	1	3.68613E-05	0.00599924
ILMN_2606825	CMAH	1.17926401	0.0002995	0.01489206	0.0002995	0.01335371	1.13E-07	0.0000431	3.18E-07	0.000121491	1	7.35697E-06	0.00232956
ILMN_1224032	SKAP1	1.178850818	0.0002995	0.01489206	0.0002995	0.01335371	1.24E-08	0.00000822	6.12E-08	0.0000375	1	3.76554E-06	0.00162291
ILMN_2436183	BCL11B	1.178588337	0.0002995	0.01489206	0.0002995	0.01335371	8.65E-08	0.0000344	0.00000254	0.000523539	1	4.7012E-05	0.00688184
ILMN_2419490	TNFRSF18	1.178043424	0.0002995	0.01489206	0.0002995	0.01335371	7.56E-11	1.35E-07	1.53E-08	0.0000132	1	1.56849E-06	0.00104298
ILMN_2432110	LOC100046855	1.176564584	0.0002995	0.01489206	0.0002995	0.01335371	1.32E-07	0.0000482	9.52E-08	0.0000521	1	1.12718E-06	0.00087445
ILMN_2443624	BCL11B	1.17644894	0.0002995	0.01489206	0.0002995	0.01335371	2.53E-07	0.0000755	0.00000244	0.000512485	1	2.24533E-05	0.00440984
ILMN_1217913	D230007K08RIK	1.176197032	0.0002995	0.01489206	0.0002995	0.01335371	2.34E-08	0.000013	8.68E-08	0.0000485	1	1.02476E-06	0.0008224
ILMN_1215649	C230075M21RIK	1.176099557	0.0002995	0.01489206	0.0002995	0.01335371	0.0000672	0.00493359	0.00041344	0.017763501	1	0.000393771	0.02371136
ILMN_1217629	ITGAE	1.175144353	0.0002995	0.01489206	0.0002995	0.01335371	2.19E-08	0.0000126	1.49E-08	0.0000131	1	4.51028E-06	0.00180983
ILMN_1215862	CXCL9	1.174363745	0.00176049	0.0385938	0.00122569	0.02881591	0.01957651	0.17739607	0.00069086	0.02516235	0	0.001301571	0.04617913
ILMN_2721399	CTSW	1.172718248	0.0002995	0.01489206	0.0002995	0.01335371	2.77E-10	4.45E-07	4.69E-09	0.00000642	1	1.8046E-06	0.001091
ILMN_1248340	LSG1	1.172636468	0.0002995	0.01489206	0.0002995	0.01335371	0.00000275	0.00048633	0.0000158	0.00199766	1	2.65234E-05	0.00480383

ILMN_2618176	SOCS3	1.171028633	0.0002995	0.01489206	0.0002995	0.01335371	5.43E-10	7.65E-07	2.48E-08	0.0000192	1	7.35697E-06	0.00232956
ILMN_1218717	LOC385615	1.170940971	0.0002995	0.01489206	0.0002995	0.01335371	0.00000442	0.00070872	0.0000267	0.002856495	1	7.57938E-05	0.00879794
ILMN_1247893	CGEF2-PENDING	1.169922593	0.0002995	0.01489206	0.0002995	0.01335371	0.00000467	0.00072982	0.0000243	0.002709534	1	0.000120719	0.01149104
ILMN_1224473	LOC380797	1.16992167	0.00122569	0.03054155	0.00094262	0.02430167	0.00015376	0.00895396	0.00147216	0.0392018	1	0.00305523	0.07341857
ILMN_2419494	TNFRSF18	1.169716279	0.0002995	0.01489206	0.0002995	0.01335371	7.66E-10	9.15E-07	4.99E-08	0.0000324	1	5.39498E-06	0.00202516
ILMN_2612125	ARHGEF18	1.169661163	0.0002995	0.01489206	0.0002995	0.01335371	4.41E-08	0.0000212	0.00000152	0.000360463	1	9.15833E-06	0.00268109
ILMN_2606162	PDLIM4	1.169633574	0.0006399	0.0211535	0.0002995	0.01335371	4.78E-07	0.00012706	0.00000832	0.001279253	1	0.000600122	0.03046224
ILMN_1217406	1110013L07RIK	1.169153834	0.0002995	0.01489206	0.0002995	0.01335371	4.77E-08	0.0000223	2.86E-07	0.000110081	1	1.13766E-05	0.00307877
ILMN_2568571	C130079K02RIK	1.168909165	0.0002995	0.01489206	0.0002995	0.01335371	5.37E-07	0.00013971	0.0000201	0.002389566	1	6.22097E-05	0.00789013
ILMN_2818964	DUSP10	1.168854568	0.0002995	0.01489206	0.0002995	0.01335371	0.0000386	0.00328743	0.000041	0.003797436	1	3.39668E-05	0.00570777
ILMN_2431390	TCRB-V8.2	1.167218786	0.0006399	0.0211535	0.0002995	0.01335371	1.7E-09	0.00000149	7.75E-08	0.0000451	1	1.23973E-05	0.00322459
ILMN_2456911	TRBV31_X03277_T	1.166841194	0.0006399	0.0211535	0.0002995	0.01335371	0.00000163	0.00032459	0.0000123	0.001649893	1	0.000540727	0.02850419
ILMN_2981169	IFIT2	1.165766787	0.0002995	0.01489206	0.0002995	0.01335371	0.0000117	0.00139985	0.00000211	0.00045645	1	3.43889E-06	0.00156931
ILMN_1249366	LOC100046608	1.165718568	0.0002995	0.01489206	0.0002995	0.01335371	1.59E-09	0.00000145	1.55E-07	0.0000735	1	5.64035E-06	0.00208385
ILMN_1246194	LOC667370	1.165517165	0.0002995	0.01489206	0.0002995	0.01335371	0.0008987	0.02917128	0.00023641	0.012496999	1	0.00022037	0.01646479
ILMN_1236507	5830496L11RIK	1.164879502	0.0002995	0.01489206	0.0002995	0.01335371	0.0000665	0.00488934	0.00043189	0.018308965	1	0.000486846	0.02669165
ILMN_1254692	MS4A6B	1.164318447	0.0002995	0.01489206	0.0002995	0.01335371	0.00000049	0.00012954	8.08E-07	0.000227616	1	3.59866E-06	0.00158025
ILMN_3156208	GPR146	1.164011442	0.0002995	0.01489206	0.0002995	0.01335371	2.38E-08	0.000013	3.86E-07	0.00013797	1	6.44447E-06	0.00218957
ILMN_2734391	RAMP1	1.16383138	0.0002995	0.01489206	0.0002995	0.01335371	5.98E-09	0.00000442	2.14E-09	0.00000357	1	3.18544E-07	0.00044521
ILMN_1224091	XIST	1.16230787	0.0006399	0.0211535	0.00094262	0.02430167	0.00084048	0.02804442	0.00095208	0.030395331	1	0.001531279	0.05016159
ILMN_2456216	5330403D14RIK	1.161809103	0.00094262	0.02607154	0.00094262	0.02430167	0.0000367	0.00319787	0.00031494	0.015019787	1	0.000903743	0.03800048
ILMN_2627441	PELI1	1.161752176	0.0002995	0.01489206	0.0002995	0.01335371	0.00000119	0.00025415	0.00000113	0.000290416	1	4.31186E-06	0.00177614
ILMN_2891245	RHBDL2	1.161573842	0.0020184	0.04200694	0.0020184	0.03953445	0.00050184	0.02040365	0.00120654	0.035013094	1	0.001977573	0.05767797
ILMN_1232804	LOC276837	1.161420855	0.0002995	0.01489206	0.0002995	0.01335371	3.19E-07	0.0000928	0.000001	0.000269028	1	6.47271E-05	0.00803428
ILMN_1232537	ARL4C	1.160980448	0.0002995	0.01489206	0.0002995	0.01335371	4.13E-07	0.000115	0.00000371	0.000722503	1	7.28705E-05	0.00865281
ILMN_2444432	TRBV1_AE000663_	1.160799824	0.0002995	0.01489206	0.0002995	0.01335371	0.00000104	0.00022691	0.00000112	0.000290018	1	7.57938E-05	0.00879794
ILMN_2756046	FFAR2	1.160027707	0.0002995	0.01489206	0.0002995	0.01335371	0.0000389	0.00330056	1.33E-07	0.000068	1	2.99936E-06	0.00146959
ILMN_1214163	B230380D07RIK	1.159421403	0.0002995	0.01489206	0.0002995	0.01335371	6.66E-08	0.0000279	4.61E-07	0.000152045	1	4.16456E-05	0.00639762
ILMN_2613832	MGST2	1.159082249	0.0002995	0.01489206	0.0002995	0.01335371	0.00000018	0.0000594	1.27E-08	0.0000119	1	1.49656E-06	0.00102442
ILMN_2506039	A130090K04RIK	1.158817015	0.0002995	0.01489206	0.0002995	0.01335371	6.61E-07	0.0001602	4.04E-07	0.00013924	1	7.03988E-06	0.00230764
ILMN_1219179	A830081L15RIK	1.158794735	0.0002995	0.01489206	0.0002995	0.01335371	0.00000165	0.00032655	0.00000379	0.000732759	1	8.39227E-06	0.00252023
ILMN_1248898	A830092P18RIK	1.158658732	0.0002995	0.01489206	0.0002995	0.01335371	0.00000523	0.00078323	0.0000278	0.002931426	1	9.5799E-05	0.01018072
ILMN_2651575	IFNGR1	1.158581155	0.0002995	0.01489206	0.0002995	0.01335371	5.63E-07	0.00014332	0.00000707	0.001150107	1	1.1878E-05	0.00314139
ILMN_2708580	INADL	1.158423303	0.0002995	0.01489206	0.0002995	0.01335371	1.33E-07	0.0000482	2.41E-07	0.000101903	1	3.76554E-06	0.00162291
ILMN_1236256	ARHGEF1	1.158058706	0.0002995	0.01489206	0.0006399	0.01941092	0.00012116	0.00760514	0.00023881	0.012588785	1	0.00022037	0.01646479
ILMN_2525855	FUS	1.157139415	0.0002995	0.01489206	0.0002995	0.01335371	0.0000124	0.00144807	0.0000814	0.006023296	1	9.5799E-05	0.01018072
ILMN_2526938	LOC623121	1.157084959	0.0002995	0.01489206	0.0002995	0.01335371	0.0000881	0.00606307	0.0001654	0.009948464	1	0.000393771	0.02371136
ILMN_1213664	LOC665425	1.156699853	0.00094262	0.02607154	0.0006399	0.01941092	0.00000457	0.0007204	0.0000219	0.002525607	1	0.000157497	0.01350097
ILMN_1255743	IL6RA	1.1564233	0.0002995	0.01489206	0.0002995	0.01335371	0.0000121	0.00143247	0.0000346	0.003367292	1	0.00011618	0.0112429
ILMN_2646625	JUN	1.155896052	0.0002995	0.01489206	0.0002995	0.01335371	4.75E-08	0.0000223	4.3E-08	0.0000294	1	8.0667E-07	0.00070845
ILMN_2843019	KLHDC1	1.155520822	0.0002995	0.01489206	0.0002995	0.01335371	5.72E-07	0.00014393	0.00000446	0.000810182	1	3.00244E-05	0.00527375
ILMN_3047389	GBP2	1.155409559	0.00397039	0.064249	0.00467484	0.06877491	0.00439071	0.07547063	0.00354418	0.067235737	0	0.003774648	0.08295494
ILMN_1257639	0610009J05RIK	1.155036694	0.0002995	0.01489206	0.0002995	0.01335371	0.0000254	0.00245781	0.00014036	0.009023792	1	0.000157487	0.01350097
ILMN_1239770	PELI1	1.154754524	0.0002995	0.01489206	0.0002995	0.01335371	2.15E-08	0.0000125	6.23E-08	0.0000377	1	1.56849E-06	0.00104298
ILMN_2669841	6330581N18RIK	1.153993411	0.0002995	0.01489206	0.0002995	0.01335371	0.00000028	0.0000823	1.21E-07	0.0000627	1	1.29931E-06	0.00093044
ILMN_2797061	ACTN2	1.153924476	0.0002995	0.01489206	0.0002995	0.01335371	3.85E-08	0.0000189	1.24E-08	0.0000119	1	1.18196E-06	0.00088737
ILMN_2784580	CD3E	1.153886402	0.00094262	0.02607154	0.0002995	0.01335371	0.00000294	0.00051612	0.0000297	0.003067006	1	0.000763113	0.03482416

ILMN_3006219	FYB	1.153762655	0.0002995	0.01489206	0.0002995	0.01335371	0.0000042	0.0006766	0.0000232	0.002627969	1	0.000157497	0.01350097
ILMN_2467190	BAMBI-PS1	1.15354344	0.0002995	0.01489206	0.0002995	0.01335371	0.00000128	0.00026647	0.0000012	0.00029799	1	2.76434E-05	0.00493033
ILMN_2536349	LOC386513	1.153082558	0.0002995	0.01489206	0.0002995	0.01335371	6.12E-08	0.0000262	4.94E-07	0.00016183	1	4.16456E-05	0.00639762
ILMN_2432550	TRIB2	1.15299348	0.0002995	0.01489206	0.0002995	0.01335371	2.08E-07	0.0000667	3.94E-07	0.00013797	1	6.16514E-06	0.0021257
ILMN_2844996	ACTN1	1.152882536	0.0002995	0.01489206	0.0002995	0.01335371	0.00000474	0.0007333	0.0000112	0.001548243	1	0.000140602	0.01273272
ILMN_1247281	5830468F06RIK	1.15264746	0.0002995	0.01489206	0.0002995	0.01335371	3.03E-07	0.0000888	7.59E-07	0.000219513	1	2.73684E-06	0.00139991
ILMN_1227814	SRR	1.1524679	0.00444101	0.0691859	0.00373333	0.05925673	0.00032551	0.01515171	0.00191731	0.04584872	0	0.004782836	0.09409752
ILMN_1249021	BCL2	1.152415672	0.0002995	0.01489206	0.0002995	0.01335371	2.39E-08	0.000013	2.71E-07	0.000109783	1	7.35697E-06	0.00232956
ILMN_2427592	LOC546630	1.152263699	0.0002995	0.01489206	0.0002995	0.01335371	3.87E-08	0.0000189	7.4E-08	0.0000436	1	8.87963E-07	0.00076541
ILMN_1259075	NME7	1.152030836	0.0002995	0.01489206	0.0002995	0.01335371	6.14E-08	0.0000262	2.53E-07	0.000106116	1	3.43889E-06	0.00156931
ILMN_1343049	control_ILMN_134	1.151797691	0.0006399	0.0211535	0.0006399	0.01941092	0.0000486	0.00386324	0.0000962	0.006777584	1	0.000111795	0.01097902
ILMN_3026397	CHKA	1.1512093	0.00373333	0.06184411	0.00397039	0.06196763	0.0007963	0.02730155	0.00350197	0.066778429	0	0.005373402	0.10008988
ILMN_2631610	TMEM71	1.15055696	0.0002995	0.01489206	0.0002995	0.01335371	0.00000196	0.00037186	7.31E-07	0.000218104	1	2.99936E-06	0.00146959
ILMN_1227434	ITGB7	1.150210635	0.0006399	0.0211535	0.0002995	0.01335371	0.00000383	0.00063236	0.00000744	0.001193798	1	7.57886E-05	0.00879794
ILMN_1232600	RNF125	1.150131069	0.0002995	0.01489206	0.0002995	0.01335371	7.88E-08	0.0000325	5.26E-07	0.000168884	1	8.0326E-06	0.00247612
ILMN_2656854	MYO6	1.149171076	0.0002995	0.01489206	0.0002995	0.01335371	0.0000106	0.00129112	0.000015	0.001927918	1	0.000763113	0.03482416
ILMN_1258652	RBBP2	1.148685264	0.0002995	0.01489206	0.0002995	0.01335371	0.0000026	0.00046493	0.00000949	0.00136995	1	2.24533E-05	0.00440984
ILMN_1245754	CD84	1.148512188	0.0002995	0.01489206	0.0002995	0.01335371	0.0000661	0.0048741	0.00015814	0.009685436	1	0.00042277	0.02466001
ILMN_2780247	LTA	1.147812483	0.00149689	0.03478153	0.00094262	0.02430167	0.0000477	0.00381669	0.000069	0.005349701	1	0.000665544	0.03206944
ILMN_2663930	SLFN1	1.147768768	0.0002995	0.01489206	0.0002995	0.01335371	0.00000138	0.00028053	0.00000835	0.001279253	1	0.000111802	0.01097902
ILMN_3023230	JMJD3	1.147753644	0.0002995	0.01489206	0.0002995	0.01335371	0.00000543	0.00080725	0.0000199	0.002370654	1	0.00011618	0.0112429
ILMN_2764727	ACTN2	1.146764113	0.0002995	0.01489206	0.0002995	0.01335371	1.37E-08	0.00000883	0.00000044	0.000148085	1	2.65234E-05	0.00480383
ILMN_2539295	LOC621968	1.14667536	0.00149689	0.03478153	0.0006399	0.01941092	0.00000448	0.00070872	0.000044	0.003965776	1	0.001797898	0.05487657
ILMN_2448404	SCL0002975.1_34(	1.146129707	0.00176049	0.0385938	0.0020184	0.03953445	0.00133016	0.03704155	0.00169107	0.042433629	1	0.002787111	0.07008733
ILMN_1241915	NOTCH1	1.145832115	0.0002995	0.01489206	0.0002995	0.01335371	0.00000308	0.00053349	0.00000333	0.000657593	1	2.24515E-05	0.00440984
ILMN_2605819	EGLN3	1.145595193	0.0006399	0.0211535	0.0006399	0.01941092	0.00000579	0.00084519	0.0000358	0.003445285	1	0.000353739	0.02219068
ILMN_1253699	2900045G02RIK	1.145548347	0.0002995	0.01489206	0.0002995	0.01335371	0.00000247	0.00045603	0.0000131	0.001740513	1	3.12891E-05	0.00543437
ILMN_1244853	LOC100044948	1.14491469	0.0002995	0.01489206	0.0002995	0.01335371	0.00000528	0.0007879	0.000019	0.002312072	1	0.00022037	0.01646479
ILMN_2589871	CD28	1.144678691	0.0006399	0.0211535	0.0002995	0.01335371	0.00000485	0.00074167	0.00000392	0.000746457	1	2.34111E-05	0.00450296
ILMN_2997406	ARL4C	1.144292866	0.00094262	0.02607154	0.0006399	0.01941092	0.0000259	0.00247667	0.0000788	0.005900268	1	0.001686537	0.05325863
ILMN_2860645	GBP6	1.143811908	0.0006399	0.0211535	0.0006399	0.01941092	0.00023405	0.01207808	0.00011452	0.007736431	1	0.000169803	0.01418997
ILMN_1249547	ZFP292	1.143548873	0.0002995	0.01489206	0.0002995	0.01335371	0.0000159	0.00174469	0.0000524	0.004443054	1	0.000163541	0.01374071
ILMN_1236539	PFKL	1.142647393	0.0002995	0.01489206	0.0002995	0.01335371	0.00000177	0.00034525	0.0000425	0.00387062	1	0.000329201	0.02128238
ILMN_2425990	4933421G18RIK	1.142576402	0.0002995	0.01489206	0.0002995	0.01335371	0.0000176	0.00184358	0.0000196	0.002353419	1	5.89742E-06	0.0021116
ILMN_2553041	A130093I21RIK	1.142422921	0.00176049	0.0385938	0.00227139	0.0429166	0.00011916	0.00754608	0.00091734	0.029755562	1	0.000873828	0.03714525
ILMN_1224945	E130113K22RIK	1.141736556	0.0002995	0.01489206	0.0002995	0.01335371	0.00000824	0.0010961	0.0000241	0.002707128	1	1.89821E-05	0.00409055
ILMN_1218525	IL18R1	1.141400055	0.0002995	0.01489206	0.0002995	0.01335371	0.0000071	0.00098878	0.00000253	0.000522839	1	5.52021E-05	0.00760204
ILMN_1243621	A130026C10RIK	1.141355268	0.0002995	0.01489206	0.0002995	0.01335371	0.00014647	0.00871594	0.00067402	0.024761981	1	0.00057968	0.02968357
ILMN_1228608	LOC637353	1.141241268	0.00373333	0.06184411	0.00349473	0.0566205	0.00162544	0.04219715	0.00320971	0.063172249	0	0.005070533	0.09692735
ILMN_1229746	ECM1	1.141034556	0.0002995	0.01489206	0.0002995	0.01335371	0.00000063	0.00015526	0.00000018	0.0000829	1	1.89087E-06	0.00111411
ILMN_2915951	D13ERTD608E	1.140936106	0.0002995	0.01489206	0.0002995	0.01335371	0.0000242	0.0023517	0.0000549	0.004568149	1	5.97852E-05	0.00777325
ILMN_1249864	A630077B13RIK	1.140911297	0.00094262	0.02607154	0.0002995	0.01335371	0.0000339	0.00303141	0.0000306	0.003131644	1	0.000111802	0.01097902
ILMN_2995934	LOC547323	1.140861547	0.0002995	0.01489206	0.0002995	0.01335371	0.00000251	0.00046209	0.00000828	0.001279253	1	3.99889E-05	0.00618393
ILMN_2456391	TCRB-J	1.140797339	0.00176049	0.0385938	0.0002995	0.01335371	0.0000215	0.00216998	0.00010468	0.007215102	1	0.001068077	0.04167292
ILMN_1239469	LEF1	1.140750182	0.0002995	0.01489206	0.0002995	0.01335371	0.00000385	0.00063236	0.0000028	0.000567027	1	9.56597E-06	0.00274856
ILMN_1259463	ANKZF1	1.140723241	0.0002995	0.01489206	0.0002995	0.01335371	1.15E-07	0.0000431	8.51E-07	0.00023576	1	2.27642E-06	0.00127663
ILMN_3142803	CXCL10	1.140155785	0.0032539	0.05678214	0.00301234	0.05155233	0.00516161	0.08343803	0.00230778	0.05128928	0	0.003346676	0.07734743

ILMN_2594768	THA1	1.139893861	0.00149689	0.03478153	0.00094262	0.02430167	0.0000101	0.00124325	0.00018697	0.010823311	1	0.000470028	0.02607676
ILMN_2677207	LOC100047788	1.139882555	0.0056004	0.08017457	0.00490758	0.07073729	0.00128112	0.03625071	0.00280343	0.058355577	0	0.00675613	0.11179438
ILMN_1226048	AI504432	1.13986418	0.0002995	0.01489206	0.0002995	0.01335371	4.96E-07	0.00013044	5.84E-07	0.000181161	1	6.16514E-06	0.0021257
ILMN_1241598	LY116	1.139617809	0.0002995	0.01489206	0.0002995	0.01335371	0.0000304	0.00278339	0.000043	0.003909	1	0.000135352	0.01242731
ILMN_1258272	LOC382646	1.139404236	0.00149689	0.03478153	0.00122569	0.02881591	0.00019446	0.01053711	0.00033198	0.01550964	1	0.001033149	0.04099743
ILMN_1238847	CD3E	1.139135835	0.0006399	0.0211535	0.0002995	0.01335371	0.00000182	0.00035008	0.00000947	0.00136995	1	0.000353739	0.02219068
ILMN_1254218	NISCH	1.138664045	0.0002995	0.01489206	0.0002995	0.01335371	0.0000288	0.00267634	0.00013436	0.00874694	1	7.28705E-05	0.00865281
ILMN_2879858	PRKX	1.138650161	0.0002995	0.01489206	0.0002995	0.01335371	9.55E-12	2.47E-08	4.3E-12	0.00000005	1	1.05033E-08	0.00012222
ILMN_1231490	2410006H16RIK	1.138597457	0.0002995	0.01489206	0.0002995	0.01335371	0.0000613	0.00458827	0.000093	0.006607866	1	9.5799E-05	0.01018072
ILMN_1232707	SAMHD1	1.138552379	0.0002995	0.01489206	0.0002995	0.01335371	0.00000184	0.00035317	2.73E-07	0.000109783	1	8.0326E-06	0.00247612
ILMN_1240592	ALS2CL	1.138470539	0.01404445	0.13554668	0.01142074	0.11750706	0.00241847	0.05412137	0.00804871	0.105414587	0	0.019643191	0.19233384
ILMN_3142602	B3GALT4	1.138320625	0.0002995	0.01489206	0.0002995	0.01335371	1.28E-07	0.0000472	2.85E-07	0.000110081	1	1.72245E-06	0.00108344
ILMN_3031781	ARID5B	1.138169635	0.0006399	0.0211535	0.00094262	0.02430167	0.00017446	0.00971369	0.00037839	0.0167608	1	0.000665544	0.03206944
ILMN_2568028	IL2RG	1.138046812	0.0006399	0.0211535	0.0002995	0.01335371	0.0000163	0.00177372	0.00010898	0.007416137	1	0.000151664	0.01324485
ILMN_1239776	LOC100040243	1.1379032	0.00094262	0.02607154	0.0006399	0.01941092	0.0000766	0.00547181	0.00037845	0.0167608	1	0.000341264	0.02181978
ILMN_1253691	B430201A12RIK	1.137684368	0.0002995	0.01489206	0.0002995	0.01335371	3.76E-07	0.00010665	3.57E-07	0.000132883	1	4.50187E-07	0.00052531
ILMN_1239238	PRKCH	1.137598283	0.0002995	0.01489206	0.0002995	0.01335371	0.00000445	0.00070872	9.27E-07	0.000252346	1	5.30373E-05	0.00748099
ILMN_2663230	SLCO3A1	1.137294887	0.00094262	0.02607154	0.0006399	0.01941092	2.28E-07	0.0000712	0.00000754	0.001205762	1	0.00010758	0.01083883
ILMN_1255869	CATNB	1.137232235	0.0002995	0.01489206	0.0002995	0.01335371	0.0000558	0.0042775	0.0000935	0.006622679	1	0.000135361	0.01242731
ILMN_2847773	OTTMUSG0000000	1.137208625	0.0002995	0.01489206	0.0002995	0.01335371	0.00000407	0.0006631	0.00000411	0.000768531	1	3.39668E-05	0.00570777
ILMN_2945940	RAPGEF4	1.136953118	0.0002995	0.01489206	0.0002995	0.01335371	0.0000964	0.00649408	0.0000445	0.003979315	1	0.000220357	0.01646479
ILMN_2426853	UBD	1.13683555	0.00149689	0.03478153	0.00149689	0.03284241	0.00151895	0.04027676	0.00035471	0.016129651	1	0.001389344	0.04772897
ILMN_2639972	PPP1R3B	1.136776701	0.0002995	0.01489206	0.0002995	0.01335371	0.0000018	0.00034718	0.00000665	0.001105293	1	0.000146035	0.01292295
ILMN_2693461	SSBP2	1.136455877	0.0002995	0.01489206	0.0002995	0.01335371	0.00000811	0.00108443	0.0000104	0.001467743	1	9.95836E-05	0.01046347
ILMN_1258988	IGFBP4	1.136364638	0.00605844	0.08369461	0.00397039	0.06196763	0.00044687	0.01884113	0.00175534	0.043416704	0	0.012677869	0.1539569
ILMN_2509988	LBR	1.136214053	0.0002995	0.01489206	0.0002995	0.01335371	0.00037342	0.01669711	0.00049349	0.020167155	1	0.000317538	0.02078825
ILMN_1230458	IFIT3	1.136194338	0.0020184	0.04200694	0.00149689	0.03284241	0.00399845	0.07166564	0.00197876	0.046801373	0	0.001301631	0.04617913
ILMN_2727235	ANKRD11	1.136184371	0.0020184	0.04200694	0.00122569	0.02881591	0.00057505	0.02210324	0.00110173	0.033170811	1	0.004006777	0.0855913
ILMN_2637714	RASA3	1.135912258	0.0002995	0.01489206	0.0002995	0.01335371	1.38E-07	0.0000492	7.38E-08	0.0000436	1	1.92936E-07	0.00034541
ILMN_2595918	GIMAP7	1.135856716	0.0006399	0.0211535	0.0006399	0.01941092	0.00000021	0.0000667	0.00000695	0.001138958	1	0.000183009	0.01471244
ILMN_1231573	SERPINB1A	1.135730392	0.0002995	0.01489206	0.0002995	0.01335371	9.05E-07	0.00020553	0.00000222	0.000472255	1	3.83949E-05	0.00609954
ILMN_2459155	ZFP238	1.135647435	0.0002995	0.01489206	0.0002995	0.01335371	0.00017162	0.00962449	0.00031671	0.015073371	1	0.000470028	0.02607676
ILMN_1243910	ZFP292	1.13546193	0.0006399	0.0211535	0.0002995	0.01335371	0.00016234	0.00923784	0.00035402	0.016129651	1	0.000712779	0.03334445
ILMN_1222471	GMFG	1.13543581	0.0002995	0.01489206	0.0002995	0.01335371	9.07E-09	0.0000064	9.8E-09	0.0000111	1	5.74828E-07	0.00056929
ILMN_2469190	B230345P09RIK	1.135305336	0.0006399	0.0211535	0.00094262	0.02430167	0.00017922	0.00989578	0.00053924	0.021235038	1	0.000212355	0.01604723
ILMN_2696492	PHF11	1.135183534	0.00094262	0.02607154	0.0006399	0.01941092	0.0000941	0.00638343	0.00015178	0.009420008	1	0.000504223	0.02732257
ILMN_2996904	OBFC2A	1.135148949	0.0002995	0.01489206	0.0002995	0.01335371	0.00000351	0.00059253	0.00000887	0.001330104	1	2.88127E-05	0.00509941
ILMN_1233449	LOC100044430	1.13513543	0.0002995	0.01489206	0.0002995	0.01335371	0.00000334	0.00057008	0.00000669	0.00110836	1	0.000212368	0.01604723
ILMN_1251193	A630084D02RIK	1.134829034	0.0002995	0.01489206	0.0002995	0.01335371	0.00032183	0.01505533	0.0002505	0.012926945	1	0.000379979	0.02324165
ILMN_2758878	TMEM66	1.13478407	0.00276802	0.05133777	0.00176049	0.03635757	0.00043749	0.01861404	0.00091474	0.029733662	0	0.003149577	0.07483876
ILMN_3160137	ALDOC	1.134554634	0.0076447	0.09672601	0.00583002	0.07876136	0.00082471	0.02769675	0.0021101	0.048647455	0	0.012022966	0.14955451
ILMN_1218051	IQGAP2	1.134418217	0.00149689	0.03478153	0.00122569	0.02881591	0.0000255	0.00246014	0.0000551	0.00457056	1	0.000643	0.03157309
ILMN_1230726	5430406J06RIK	1.13436247	0.00122569	0.03054155	0.00094262	0.02430167	0.00022223	0.01167751	0.0008189	0.027904422	1	0.001581552	0.05126497
ILMN_2879614	ZBP1	1.13418419	0.0006399	0.0211535	0.0006399	0.01941092	0.0000132	0.00150968	0.0000283	0.002963331	1	0.000255296	0.01817016
ILMN_2534207	LOC380706	1.133996759	0.0002995	0.01489206	0.0002995	0.01335371	3.59E-08	0.000018	8.2E-09	0.0000103	1	7.68761E-07	0.00070164
ILMN_2659739	IL7R	1.133870404	0.0002995	0.01489206	0.0002995	0.01335371	0.0000625	0.00467003	0.0000102	0.001439966	1	7.88278E-05	0.00901523
ILMN_2958159	ENO1	1.13384821	0.0002995	0.01489206	0.0002995	0.01335371	0.00000265	0.00047116	0.0000399	0.003729707	1	0.000329201	0.02128238

ILMN_1257704	6030458P06RIK	1.133768716	0.0006399	0.0211535	0.0006399	0.01941092	0.0000499	0.00393179	0.00019668	0.011191632	1	0.000197175	0.01547711
ILMN_2477213	LOC100045240	1.133690768	0.0002995	0.01489206	0.0002995	0.01335371	2.65E-07	0.0000787	7.57E-07	0.000219513	1	4.12145E-06	0.00171287
ILMN_2513870	ZAP70	1.133508982	0.0002995	0.01489206	0.0002995	0.01335371	2.43E-07	0.0000731	0.00000252	0.000522839	1	0.000163531	0.01374071
ILMN_1343061	control_ILMN_134	1.133413667	0.05896005	0.29652912	0.05313904	0.27805718	0.0444304	0.27002241	0.05091233	0.282020251	0	0.090139534	0.39515789
ILMN_1258340	TCF12	1.133249325	0.0002995	0.01489206	0.0002995	0.01335371	0.0000229	0.00226216	0.000017	0.002131671	1	2.06483E-05	0.00430994
ILMN_2777019	SPO11	1.133035275	0.0006399	0.0211535	0.0006399	0.01941092	0.00017061	0.00961402	0.000037	0.003539707	1	0.000157497	0.01350097
ILMN_1255907	ARP2	1.132980051	0.0002995	0.01489206	0.0002995	0.01335371	0.0000147	0.00164902	0.0000292	0.003023837	1	4.51564E-05	0.00675851
ILMN_2449620	5830427D02RIK	1.132961187	0.0002995	0.01489206	0.0002995	0.01335371	0.0000477	0.00381669	0.00019019	0.010942585	1	7.57938E-05	0.00879794
ILMN_2477946	SCL0001464.1_61	1.132866527	0.0002995	0.01489206	0.0002995	0.01335371	0.00000865	0.00113789	0.000027	0.002873981	1	0.00040803	0.02413289
ILMN_2627179	ELL3	1.132790682	0.0002995	0.01489206	0.0002995	0.01335371	9.29E-09	0.00000645	1.03E-08	0.0000115	1	1.74307E-07	0.00034541
ILMN_1214318	RASGRP1	1.132335612	0.0006399	0.0211535	0.0002995	0.01335371	0.0000771	0.0054869	0.0000845	0.006173424	1	0.000873828	0.03714525
ILMN_1218206	KLF13	1.132335512	0.0020184	0.04200694	0.00149689	0.03284241	0.00013977	0.00848224	0.00056901	0.022034784	1	0.002540648	0.06640064
ILMN_1233501	C530027B15RIK	1.132246044	0.00122569	0.03054155	0.00122569	0.02881591	0.00014451	0.00864564	0.00060955	0.023104722	1	0.000737547	0.0342621
ILMN_1241522	4921505C17RIK	1.13221457	0.0002995	0.01489206	0.0002995	0.01335371	6.62E-07	0.0001602	0.00000201	0.000441415	1	6.16514E-06	0.0021257
ILMN_1226239	INPP4B	1.132137878	0.00176049	0.0385938	0.00176049	0.03635757	0.00019583	0.01059928	0.00041	0.01769293	1	0.000540669	0.02850419
ILMN_2613422	ITK	1.131935528	0.0006399	0.0211535	0.0006399	0.01941092	0.0000104	0.00127268	0.000056	0.004602895	1	0.000140602	0.01273272
ILMN_2593554	IGTP	1.131930192	0.0002995	0.01489206	0.0002995	0.01335371	0.0000358	0.00314503	0.00000464	0.000834646	1	1.67194E-05	0.0037966
ILMN_1229804	LTA	1.131546852	0.00583002	0.08214635	0.00252097	0.04602629	0.00028612	0.01388718	0.00121075	0.035093672	0	0.008450429	0.12538799
ILMN_2607160	PUM2	1.130377924	0.0006399	0.0211535	0.0002995	0.01335371	0.00000172	0.00033587	0.00000121	0.000298774	1	2.76434E-05	0.00493033
ILMN_3072437	6330500D04RIK	1.130133265	0.00301234	0.05440113	0.00252097	0.04602629	0.00089012	0.02903745	0.0012227	0.035284033	0	0.00210642	0.05997046
ILMN_1251170	AFF4	1.130045502	0.0002995	0.01489206	0.0002995	0.01335371	0.0000524	0.00405887	0.00011408	0.007718214	1	7.0054E-05	0.00846962
ILMN_1246582	LOC436541	1.129946125	0.0006399	0.0211535	0.0006399	0.01941092	0.0000207	0.00211087	0.0000462	0.004074421	1	0.000176289	0.01444673
ILMN_1244891	CST7	1.1298809	0.0002995	0.01489206	0.0002995	0.01335371	0.0000285	0.00265981	0.0000142	0.001859837	1	0.000146035	0.01292295
ILMN_2697693	GRHPR	1.129040446	0.0006399	0.0211535	0.0006399	0.01941092	0.0000147	0.00164902	0.0000385	0.003609572	1	0.000157497	0.01350097
ILMN_1233402	LOC100045981	1.129004666	0.0002995	0.01489206	0.0002995	0.01335371	1.69E-07	0.0000566	0.00000129	0.000312387	1	7.03988E-06	0.00230764
ILMN_1258864	BC106179	1.128947865	0.0002995	0.01489206	0.0006399	0.01941092	0.00043723	0.01861404	0.00059166	0.022629221	1	0.000317538	0.02078825
ILMN_1234796	HSD17B12	1.128868369	0.0002995	0.01489206	0.0002995	0.01335371	0.0000811	0.00570333	0.00021329	0.011819303	1	0.000197175	0.01547711
ILMN_1236595	SLA2	1.128559293	0.00176049	0.0385938	0.00122569	0.02881591	0.0000231	0.00227606	0.00024722	0.012817366	1	0.001344859	0.0469611
ILMN_1241952	6230415M23RIK	1.12852499	0.0002995	0.01489206	0.0006399	0.01941092	0.00028485	0.01386905	0.00098023	0.030953762	1	0.00148248	0.04961268
ILMN_2657980	FAAH	1.128386309	0.0002995	0.01489206	0.0002995	0.01335371	0.0000342	0.00304391	0.00014089	0.009031912	1	0.00040803	0.02413289
ILMN_2745614	FAM134B	1.127986989	0.0002995	0.01489206	0.0002995	0.01335371	0.0000227	0.00225688	0.000034	0.003321814	1	0.000264806	0.01861919
ILMN_1240415	LOC234987	1.127947991	0.0002995	0.01489206	0.0002995	0.01335371	0.0000121	0.00143247	0.0000715	0.005501275	1	0.000163531	0.01374071
ILMN_2552490	6720463L11RIK	1.127757726	0.0006399	0.0211535	0.0002995	0.01335371	0.0000289	0.00267683	0.00012185	0.008102409	1	0.000317538	0.02078825
ILMN_2729447	9030612M13RIK	1.12772184	0.0002995	0.01489206	0.0002995	0.01335371	0.00000038	0.0001073	0.00000154	0.000360893	1	1.24004E-05	0.00322459
ILMN_1233293	GBP1	1.127491461	0.0002995	0.01489206	0.0002995	0.01335371	0.00077034	0.02679885	0.0000253	0.002787107	1	3.39668E-05	0.00570777
ILMN_1236105	FYB	1.127293421	0.00176049	0.0385938	0.0020184	0.03953445	0.00046297	0.01920664	0.00246862	0.053569604	0	0.003663228	0.08166297
ILMN_3009860	SELL	1.126985915	0.0006399	0.0211535	0.0002995	0.01335371	0.00000258	0.00046493	0.0000101	0.001437553	1	6.47271E-05	0.00803428
ILMN_1217929	LOC667005	1.126757091	0.00094262	0.02607154	0.00094262	0.02430167	0.00030191	0.01442973	0.00061251	0.023197995	1	0.000763113	0.03482416
ILMN_1219896	LOC623121	1.126643386	0.0002995	0.01489206	0.0006399	0.01941092	0.00017252	0.00966351	0.00036014	0.0162751	1	0.000169803	0.01418997
ILMN_2808485	GBP10	1.126535797	0.00094262	0.02607154	0.00094262	0.02430167	0.00089514	0.02913696	0.00032196	0.015214362	1	0.000903743	0.03800048
ILMN_2628271	LOC100045967	1.126530154	0.00122569	0.03054155	0.0006399	0.01941092	0.00000934	0.00118696	0.00014347	0.009094873	1	0.001741399	0.05411008
ILMN_2959272	RNU6	1.126488141	0.0002995	0.01489206	0.0002995	0.01335371	0.0001271	0.00781497	0.0000785	0.005894011	1	5.52021E-05	0.00760204
ILMN_1240857	COX7A1	1.126488113	0.0002995	0.01489206	0.0002995	0.01335371	0.00000712	0.00098878	0.0000106	0.001479487	1	1.08955E-05	0.00300091
ILMN_1248837	TBXA2R	1.126308353	0.0006399	0.0211535	0.0006399	0.01941092	0.0000496	0.00391795	0.0000556	0.004598957	1	0.00010758	0.01083883
ILMN_1213986	LOC380617	1.126269609	0.0002995	0.01489206	0.0002995	0.01335371	0.0000257	0.00247459	0.0000184	0.002261567	1	2.24533E-05	0.00440984
ILMN_2565089	A830083H19RIK	1.126188031	0.0002995	0.01489206	0.0002995	0.01335371	0.0000412	0.00347143	0.000057	0.00465062	1	3.99889E-05	0.00618393
ILMN_2756628	CMC1	1.125722229	0.0002995	0.01489206	0.0002995	0.01335371	1.76E-09	0.00000151	6.75E-09	0.00000872	1	3.51784E-07	0.00045485

ILMN_2689307	SPNB2	1.125305551	0.0002995	0.01489206	0.0002995	0.01335371	0.0000723	0.00525285	0.00023598	0.01249632	1	0.000284828	0.01943975
ILMN_2551741	0610010I05RIK	1.124918642	0.0006399	0.0211535	0.0006399	0.01941092	0.00046313	0.01920664	0.00104266	0.032055996	1	0.000540698	0.02850419
ILMN_1343062	control_ILMN_134	1.124836817	0.06518171	0.31189827	0.05958295	0.29527363	0.05222749	0.29304733	0.05948765	0.305188121	0	0.168994973	0.52187414
ILMN_2749364	LOC382646	1.124580104	0.00276802	0.05133777	0.00176049	0.03635757	0.0000498	0.00393109	0.00024533	0.01278781	0	0.002963349	0.07240684
ILMN_2784272	IFNGR2	1.124504822	0.0020184	0.04200694	0.00176049	0.03635757	0.0008594	0.02845121	0.0016295	0.041583456	1	0.000903743	0.03800048
ILMN_1225348	FAM102A	1.12448091	0.0002995	0.01489206	0.0002995	0.01335371	0.00000714	0.00098905	0.0000248	0.002755904	1	0.000189968	0.01508948
ILMN_2630739	PHF20L1	1.124360297	0.00094262	0.02607154	0.00149689	0.03284241	0.00051915	0.02079588	0.00041925	0.017887063	1	0.000393771	0.02371136
ILMN_2418366	6720427H10RIK	1.124259069	0.0002995	0.01489206	0.0002995	0.01335371	0.00014538	0.00867674	0.00026831	0.013575239	1	0.000540727	0.02850419
ILMN_2846731	1110059E24RIK	1.124049613	0.0002995	0.01489206	0.0002995	0.01335371	2.88E-08	0.0000149	1.45E-07	0.0000709	1	2.15328E-05	0.0043202
ILMN_2594855	PDPK1	1.124023837	0.0002995	0.01489206	0.0002995	0.01335371	0.0000466	0.00377453	0.0000558	0.004598957	1	0.000189956	0.01508948
ILMN_1246543	SIAT7C	1.124022495	0.0002995	0.01489206	0.0002995	0.01335371	0.00032001	0.01501554	0.00045251	0.018972559	1	0.000737547	0.0342621
ILMN_2614884	PJA1	1.123647984	0.0002995	0.01489206	0.0002995	0.01335371	0.00000151	0.00030356	0.00000406	0.000761994	1	1.74444E-05	0.00390377
ILMN_1250418	NPC1	1.123625032	0.0002995	0.01489206	0.0002995	0.01335371	1.39E-07	0.0000492	2.76E-07	0.000109783	1	2.27642E-06	0.00127663
ILMN_2706514	LOC100046608	1.123601186	0.00094262	0.02607154	0.0006399	0.01941092	0.00010046	0.00665173	0.00032888	0.015447583	1	0.001581552	0.05126497
ILMN_1241924	ARL6IP5	1.123582409	0.0002995	0.01489206	0.0002995	0.01335371	0.0000299	0.00274992	0.00010627	0.007284834	1	0.00027463	0.01910904
ILMN_2750265	ELMO1	1.123418741	0.00122569	0.03054155	0.00122569	0.02881591	0.0004071	0.01774258	0.00092146	0.02980611	1	0.001915982	0.05666025
ILMN_2712895	4631423B10RIK	1.123402203	0.0002995	0.01489206	0.0002995	0.01335371	0.0000422	0.0035217	0.00010479	0.007215102	1	0.000176289	0.01444673
ILMN_2719139	AB124611	1.122548641	0.00122569	0.03054155	0.0006399	0.01941092	0.00023483	0.0121047	0.00029401	0.014330244	1	0.001581552	0.05126497
ILMN_2692986	2810410P22RIK	1.122423336	0.00149689	0.03478153	0.00122569	0.02881591	0.00015964	0.00916202	0.00016472	0.009931795	1	0.0007895	0.03543767
ILMN_1228333	PRF1	1.122189532	0.00276802	0.05133777	0.00122569	0.02881591	0.0001055	0.00691623	0.00012319	0.008156571	0	0.002173782	0.0607706
ILMN_2940446	DGKA	1.122160698	0.00176049	0.0385938	0.00149689	0.03284241	0.0000799	0.00565291	0.00044116	0.018633965	1	0.003449313	0.07881943
ILMN_3034381	NUP88	1.121988063	0.00094262	0.02607154	0.00094262	0.02430167	0.00011496	0.00735062	0.00016939	0.010108673	1	0.000246108	0.01770572
ILMN_2668387	CGGBP1	1.121946931	0.0002995	0.01489206	0.0002995	0.01335371	0.00000034	0.0000984	1.68E-07	0.000078	1	3.59866E-06	0.00158025
ILMN_2592953	SCG5	1.121946089	0.0002995	0.01489206	0.0002995	0.01335371	0.000026	0.00247667	0.0000347	0.003370075	1	0.000228655	0.01684049
ILMN_1228867	A130082M07RIK	1.121929865	0.00094262	0.02607154	0.0002995	0.01335371	0.00000462	0.00072482	0.0000117	0.001595024	1	0.00042277	0.02466001
ILMN_2785454	HIST2H2AB	1.121848738	0.00898742	0.10547593	0.00965407	0.10652405	0.00889504	0.11365284	0.01039259	0.121665992	0	0.049191671	0.30108149
ILMN_2773900	GLIPR2	1.1217158	0.0006399	0.0211535	0.0006399	0.01941092	6.02E-07	0.00014992	0.00000244	0.000512485	1	3.83949E-05	0.00609954
ILMN_2647102	ACTL6A	1.121390591	0.0002995	0.01489206	0.0002995	0.01335371	0.00000884	0.00115212	0.0000424	0.00387062	1	7.57938E-05	0.00879794
ILMN_1256574	NCOA2	1.121377157	0.00094262	0.02607154	0.00094262	0.02430167	0.0000493	0.00391165	0.00022291	0.012129499	1	0.00040803	0.02413289
ILMN_1243902	A530024C08RIK	1.12112103	0.0020184	0.04200694	0.00176049	0.03635757	0.00045505	0.01903079	0.00088129	0.029196677	1	0.00305523	0.07341857
ILMN_1343048	control_ILMN_134	1.120964441	0.0006399	0.0211535	0.0006399	0.01941092	0.00023856	0.01218903	0.00027171	0.013620278	1	0.000934606	0.03863507
ILMN_2878979	TNFAIP8L2	1.120676477	0.0002995	0.01489206	0.0002995	0.01335371	4.64E-07	0.00012621	0.00000373	0.000722783	1	1.74444E-05	0.00390377
ILMN_2887619	RPS25	1.120128473	0.03241096	0.21464233	0.03788279	0.2312766	0.02525122	0.20197503	0.03226412	0.224678675	0	0.039320852	0.27099019
ILMN_2678724	DUSP10	1.119986223	0.0006399	0.0211535	0.0002995	0.01335371	0.00021616	0.0114725	0.00028764	0.014123395	1	0.000379979	0.02324165
ILMN_1244135	ARPC5	1.119864481	0.00301234	0.05440113	0.00373333	0.05925673	0.00703752	0.09959734	0.00558971	0.086699245	0	0.00450999	0.09155774
ILMN_2651297	4632428N05RIK	1.119607561	0.0002995	0.01489206	0.0002995	0.01335371	0.00000985	0.00121884	0.00000212	0.00045645	1	1.35117E-05	0.00345566
ILMN_2822842	1600014C10RIK	1.119392625	0.0002995	0.01489206	0.0002995	0.01335371	0.0000173	0.00183184	0.00000567	0.00097714	1	1.97985E-05	0.00420804
ILMN_2665545	RIN3	1.119281709	0.0002995	0.01489206	0.0002995	0.01335371	0.0000502	0.0039435	0.0000406	0.003782136	1	0.000246108	0.01770572
ILMN_2703321	ANKZF1	1.119253	0.0002995	0.01489206	0.0002995	0.01335371	0.0000123	0.0014435	0.0000441	0.003965776	1	0.000146035	0.01292295
ILMN_1259917	D330001F19RIK	1.119179745	0.0002995	0.01489206	0.0002995	0.01335371	0.0002103	0.01126316	0.00034508	0.015942739	1	0.000353739	0.02219068
ILMN_1237871	AMPD1	1.119072973	0.0002995	0.01489206	0.0002995	0.01335371	0.000036	0.00314503	0.0000107	0.001483462	1	4.70154E-05	0.00688184
ILMN_1256950	NEURL2	1.11903277	0.0006399	0.0211535	0.0006399	0.01941092	0.0000169	0.00180017	0.0000192	0.002327044	1	3.83949E-05	0.00609954
ILMN_1228020	1500010G04RIK	1.118985348	0.00122569	0.03054155	0.00094262	0.02430167	0.0000866	0.00599563	0.00023442	0.012474872	1	0.000934606	0.03863507
ILMN_1227573	IRF7	1.118870281	0.00397039	0.064249	0.00397039	0.06196763	0.0023181	0.05257933	0.00445393	0.076746435	0	0.002387591	0.06387081
ILMN_2726315	SDF4	1.118652369	0.00149689	0.03478153	0.0006399	0.01941092	0.00000926	0.00118448	0.0000286	0.002983758	1	0.000621233	0.03109305
ILMN_1227907	GMFG	1.11859249	0.0002995	0.01489206	0.0002995	0.01335371	1.41E-08	0.00000898	1.13E-07	0.0000599	1	7.03866E-06	0.00230764
ILMN_2881296	TMEM66	1.118516263	0.0002995	0.01489206	0.0002995	0.01335371	0.00000179	0.00034718	0.00000451	0.000814057	1	1.29447E-05	0.00332893

ILMN_3043245	JMJD1A	1.118512017	0.0002995	0.01489206	0.0002995	0.01335371	2.31E-07	0.0000712	5.23E-07	0.000168884	1	3.93982E-06	0.00166715
ILMN_2580159	C230070D10RIK	1.118494179	0.00094262	0.02607154	0.00094262	0.02430167	0.00055671	0.02167037	0.00125356	0.035750241	1	0.000712779	0.03334445
ILMN_1252472	ITK	1.118457931	0.00227139	0.04545806	0.00276802	0.04845153	0.0005963	0.02265805	0.00234006	0.051632853	0	0.001856078	0.05577459
ILMN_2828916	FRMD6	1.11830741	0.0006399	0.0211535	0.0006399	0.01941092	0.00017343	0.00968113	0.0000582	0.004700168	1	0.000182997	0.01471244
ILMN_1257771	LOC638301	1.118249781	0.00149689	0.03478153	0.00094262	0.02430167	0.0000793	0.00561815	0.0000839	0.006145701	1	0.001218998	0.0445025
ILMN_3002095	IL27RA	1.118221343	0.0006399	0.0211535	0.0006399	0.01941092	0.0000149	0.00166378	0.0000489	0.004233399	1	0.000621233	0.03109305
ILMN_2658961	DGKA	1.118166028	0.00176049	0.0385938	0.00176049	0.03635757	0.00026591	0.01325214	0.00064942	0.024048059	1	0.002314175	0.06295874
ILMN_2038771	control_ILMN_203	1.118161322	0.06020572	0.30013907	0.05480359	0.28252036	0.05700779	0.30549638	0.05139364	0.283318913	0	0.154914896	0.50337338
ILMN_2981801	HIST1H2AG	1.118065046	0.00094262	0.02607154	0.0006399	0.01941092	0.00037162	0.01664832	0.00020154	0.011374568	1	0.000504223	0.02732257
ILMN_1249710	E030038D23RIK	1.117909411	0.0002995	0.01489206	0.0002995	0.01335371	0.0000376	0.00323771	0.00015401	0.009469751	1	0.000306263	0.02048222
ILMN_2943057	ARL5C	1.117880079	0.00176049	0.0385938	0.00094262	0.02430167	0.00042833	0.01837561	0.00044307	0.018680938	1	0.000763113	0.03482416
ILMN_1235856	8030474H12RIK	1.117810336	0.0002995	0.01489206	0.0002995	0.01335371	0.00024238	0.01232448	0.00022154	0.012090825	1	0.000103509	0.01061242
ILMN_1247942	MGEA5	1.117806646	0.0006399	0.0211535	0.0006399	0.01941092	0.00030694	0.0145343	0.00051962	0.020743454	1	0.00042277	0.02466001
ILMN_1258509	SIT1	1.117432738	0.0006399	0.0211535	0.0002995	0.01335371	0.00015174	0.00888405	0.00010235	0.007068104	1	0.000237231	0.01736224
ILMN_2544056	HBB-B1	1.117213432	0.03914176	0.23704991	0.03599148	0.22485976	0.02097723	0.18409981	0.02922158	0.212190751	0	0.025142431	0.2182235
ILMN_2729953	PPM1B	1.117036433	0.00537037	0.07818332	0.00537037	0.07497442	0.00364121	0.06763268	0.00503541	0.081966426	0	0.008450429	0.12538799
ILMN_1219602	LOC100048770	1.116440805	0.00397039	0.064249	0.00373333	0.05925673	0.00631793	0.09355312	0.00215116	0.049131535	0	0.004251793	0.08882774
ILMN_2681670	VPS37B	1.116406759	0.0002995	0.01489206	0.0002995	0.01335371	0.00000824	0.0010961	0.0000174	0.002162819	1	6.22097E-05	0.00789013
ILMN_1234955	6430573H23RIK	1.116406299	0.00176049	0.0385938	0.0020184	0.03953445	0.00074236	0.02603981	0.00161499	0.04134913	1	0.002387591	0.06387081
ILMN_2582659	E030040J04RIK	1.116391042	0.0002995	0.01489206	0.0002995	0.01335371	0.0000266	0.00251609	0.000057	0.00465062	1	0.00010758	0.01083883
ILMN_1252514	C920016N10RIK	1.116354745	0.00176049	0.0385938	0.00122569	0.02881591	0.00015113	0.00885986	0.00043359	0.018347587	1	0.0007895	0.03543767
ILMN_2617433	ACTB	1.11629809	0.00397039	0.064249	0.0032539	0.05432415	0.00287066	0.05995273	0.00272663	0.057221177	0	0.00553098	0.10148188
ILMN_2738972	CNOT4	1.116182237	0.0002995	0.01489206	0.0002995	0.01335371	0.0000107	0.00130299	0.00000896	0.00133296	1	2.65234E-05	0.00480383
ILMN_2553280	4732470M22RIK	1.1161327	0.0002995	0.01489206	0.0002995	0.01335371	0.00000649	0.00093461	0.0000318	0.003192608	1	0.000111802	0.01097902
ILMN_1238725	C130086J11RIK	1.116108151	0.01382667	0.13456133	0.01513108	0.13869555	0.01286055	0.14095122	0.01459273	0.14770671	0	0.023367993	0.21022612
ILMN_2998813	SEMA4F	1.115968974	0.0002995	0.01489206	0.0002995	0.01335371	0.0000207	0.00211087	0.0000384	0.003609572	1	8.86334E-05	0.00982291
ILMN_1236445	9130227N12RIK	1.115922245	0.00252097	0.04839275	0.00176049	0.03635757	0.00096123	0.03064541	0.0019356	0.046142977	1	0.00305523	0.07341857
ILMN_1220893	ZFP281	1.115869505	0.0002995	0.01489206	0.0002995	0.01335371	0.00000124	0.00026043	8.12E-07	0.000227616	1	5.3945E-06	0.00202516
ILMN_2417398	IGHV10S1_AF0644	1.115826459	0.00490758	0.07399298	0.0056004	0.0767776	0.00919199	0.1159197	0.0048635	0.080501378	0	0.005531174	0.10148188
ILMN_1238893	LIME1	1.115781178	0.00176049	0.0385938	0.0006399	0.01941092	0.00012493	0.00775324	0.00014361	0.009094873	1	0.001141234	0.04287409
ILMN_2706550	SUSD3	1.115744092	0.00122569	0.03054155	0.00094262	0.02430167	0.00014378	0.00861922	0.00016702	0.010005399	1	0.000816732	0.0360345
ILMN_1242235	TOP2A	1.115488708	0.0006399	0.0211535	0.0002995	0.01335371	9.23E-07	0.00020859	0.00000447	0.00081037	1	7.28705E-05	0.00865281
ILMN_2638923	RN18S	1.115471516	0.00301234	0.05440113	0.00583002	0.07876136	0.00712305	0.10016817	0.00794885	0.10463667	0	0.002620495	0.06768933
ILMN_1232894	ZNRF1	1.115445926	0.0002995	0.01489206	0.0002995	0.01335371	0.0000217	0.00217431	0.0000588	0.004724126	1	0.00010758	0.01083883
ILMN_1218799	EMB	1.115430499	0.0002995	0.01489206	0.0002995	0.01335371	0.00000545	0.00080859	0.00000586	0.00100296	1	1.04339E-05	0.00292569
ILMN_1257209	WIPF1	1.115376002	0.0002995	0.01489206	0.0002995	0.01335371	0.0000145	0.00163779	0.000022	0.002530716	1	9.57926E-05	0.01018072
ILMN_2711705	DUSP11	1.115060696	0.0002995	0.01489206	0.0002995	0.01335371	1.99E-07	0.0000652	0.0000011	0.000288061	1	3.99889E-05	0.00618393
ILMN_1236202	C430015M08RIK	1.114991211	0.0006399	0.0211535	0.00094262	0.02430167	0.00030256	0.01442973	0.00099864	0.031261571	1	0.002041151	0.05875662
ILMN_1233474	IL2RA	1.114952254	0.00809366	0.10014632	0.00537037	0.07497442	0.00167948	0.04316669	0.00320035	0.063041403	0	0.005220076	0.09861156
ILMN_2762863	AI606181	1.114892504	0.0006399	0.0211535	0.0002995	0.01335371	0.00019259	0.01044808	0.00019794	0.011235697	1	0.0007895	0.03543767
ILMN_2644350	THY1	1.114766581	0.00094262	0.02607154	0.0006399	0.01941092	0.0000457	0.00372533	0.00012026	0.008031164	1	0.002463032	0.06525142
ILMN_1255860	KLRD1	1.114664485	0.0002995	0.01489206	0.0002995	0.01335371	0.00017959	0.00990444	0.0000691	0.005350074	1	0.000183009	0.01471244
ILMN_2636403	AXUD1	1.11465607	0.0002995	0.01489206	0.0002995	0.01335371	9.85E-07	0.00021734	6.14E-07	0.00018775	1	1.08955E-05	0.00300091
ILMN_2918002	GBP3	1.114435116	0.00094262	0.02607154	0.0002995	0.01335371	0.00057359	0.02206532	0.00015319	0.009469751	1	0.0007895	0.03543767
ILMN_2649068	IRF1	1.114384547	0.00719408	0.09294718	0.00719408	0.09003289	0.00662885	0.09604617	0.00588889	0.088967898	0	0.006383386	0.10923805
ILMN_1215939	TRBV13-1_M1561	1.114252325	0.0006399	0.0211535	0.0006399	0.01941092	0.0000276	0.00258118	0.000058	0.004693744	1	0.000111795	0.01097902
ILMN_2520264	2010016I18RIK	1.114246403	0.00122569	0.03054155	0.00149689	0.03284241	0.00065214	0.02390174	0.00109041	0.033000801	1	0.001389407	0.04772897

ILMN_1220889	NBR1	1.114192214	0.0002995	0.01489206	0.0002995	0.01335371	0.00041846	0.01811912	0.0000489	0.004233399	1	0.000103509	0.01061242
ILMN_2706803	CRLF3	1.114104774	0.0002995	0.01489206	0.0002995	0.01335371	0.00012104	0.00760514	0.0000382	0.003601497	1	0.000212368	0.01604723
ILMN_2751603	PHXR4	1.11385586	0.00876432	0.1043511	0.01317262	0.12756294	0.00581702	0.08937751	0.01967293	0.172805787	0	0.017311316	0.18018556
ILMN_1240030	NFATC3	1.113638258	0.0002995	0.01489206	0.0002995	0.01335371	0.0000187	0.00193661	0.000099	0.006922165	1	0.000204639	0.01592867
ILMN_1242390	ACTN4	1.113610426	0.0067415	0.08943186	0.00537037	0.07497442	0.00225911	0.05195492	0.00310551	0.061933186	0	0.002963349	0.07240684
ILMN_2510694	NME7	1.113521644	0.0020184	0.04200694	0.00176049	0.03635757	0.00018011	0.0099096	0.0002504	0.012926945	1	0.002540648	0.06640064
ILMN_1258466	LOC380623	1.113479163	0.00149689	0.03478153	0.00122569	0.02881591	0.0000863	0.00599563	0.00049148	0.020138305	1	0.001104095	0.04229821
ILMN_1252089	NFATC3	1.113257321	0.00094262	0.02607154	0.0006399	0.01941092	0.00044981	0.01893053	0.00134792	0.037368442	1	0.001344859	0.0469611
ILMN_2522571	SETD7	1.113080478	0.0002995	0.01489206	0.0002995	0.01335371	0.0001715	0.00962449	0.0000765	0.005770769	1	1.81978E-05	0.00399552
ILMN_2593002	ST6GAL2	1.113003988	0.0002995	0.01489206	0.0002995	0.01335371	0.0000472	0.00380085	0.00000968	0.001390556	1	4.89467E-05	0.00705363
ILMN_1248379	EG434077	1.112625375	0.0002995	0.01489206	0.0002995	0.01335371	0.0000115	0.00138752	0.000012	0.001629081	1	1.53585E-05	0.00359242
ILMN_1223119	BCL11B	1.112508226	0.0020184	0.04200694	0.00122569	0.02881591	0.0000582	0.00441806	0.00012873	0.008463041	1	0.000688785	0.03268184
ILMN_3059798	FOXK1	1.112313504	0.00122569	0.03054155	0.00094262	0.02430167	0.00017816	0.00984919	0.00028068	0.013898949	1	0.000688785	0.03268184
ILMN_2534794	LOC236170	1.112310228	0.0051392	0.07619761	0.00420612	0.06441203	0.00030301	0.01443599	0.00092888	0.029963118	0	0.002620495	0.06768933
ILMN_2618549	RAB7	1.112121637	0.0006399	0.0211535	0.00122569	0.02881591	0.00187936	0.0464569	0.00194545	0.046343295	1	0.001915982	0.05666025
ILMN_2468405	5630401D06RIK	1.111986642	0.0002995	0.01489206	0.0002995	0.01335371	2.09E-07	0.0000667	0.00000224	0.00047443	1	9.99008E-06	0.00281847
ILMN_1258587	LOC100044376	1.111863958	0.0002995	0.01489206	0.0002995	0.01335371	0.00000504	0.00076175	0.00000641	0.001075031	1	2.76413E-05	0.00493033
ILMN_1218732	2810426P10RIK	1.11161873	0.0002995	0.01489206	0.0002995	0.01335371	0.0000688	0.00501883	0.00013675	0.008839595	1	0.000183009	0.01471244
ILMN_1237980	0610010D24RIK	1.111616983	0.0006399	0.0211535	0.0002995	0.01335371	0.00014715	0.00873651	0.00031915	0.015112162	1	0.000486846	0.02669165
ILMN_1214874	LOC386068	1.111606821	0.00094262	0.02607154	0.0002995	0.01335371	0.0000748	0.0053805	0.0001023	0.007068104	1	0.000844832	0.03651291
ILMN_1220418	HCST	1.111606518	0.0002995	0.01489206	0.0002995	0.01335371	0.0001505	0.00885642	0.0000483	0.004205779	1	5.74463E-05	0.00761864
ILMN_2550291	2310032M22RIK	1.111606315	0.0006399	0.0211535	0.0006399	0.01941092	0.00010944	0.00711397	0.00012106	0.008061295	1	0.000135361	0.01242731
ILMN_1234202	PHIP	1.111345184	0.0002995	0.01489206	0.00094262	0.02430167	0.00090293	0.02928231	0.00134681	0.037363102	1	0.000486846	0.02669165
ILMN_2426949	TNFRSF14	1.111293325	0.0006399	0.0211535	0.0006399	0.01941092	0.0000768	0.00547429	0.00020508	0.011473406	1	0.000453754	0.02560106
ILMN_2497395	9130004J05RIK	1.111130133	0.00122569	0.03054155	0.00122569	0.02881591	0.00027804	0.01366608	0.00076296	0.026721993	1	0.002106509	0.05997046
ILMN_2715730	SPN	1.111033738	0.0002995	0.01489206	0.0006399	0.01941092	0.0000115	0.00138491	0.0000749	0.005687727	1	0.000284828	0.01943975
ILMN_1217606	1500005K14RIK	1.110936144	0.00122569	0.03054155	0.00149689	0.03284241	0.00036072	0.01628339	0.00024226	0.012705581	1	0.000737547	0.0342621
ILMN_1216313	STIM1	1.110931979	0.00122569	0.03054155	0.00227139	0.0429166	0.00138944	0.03799885	0.00340804	0.065686921	0	0.004644684	0.0931111
ILMN_1252761	DRG1	1.110887212	0.00122569	0.03054155	0.0006399	0.01941092	0.00096761	0.03080362	0.00048307	0.019916078	1	0.000486846	0.02669165
ILMN_2710312	LOC100046650	1.110811588	0.0002995	0.01489206	0.0002995	0.01335371	0.00000957	0.00121086	0.0000519	0.004442214	1	0.000470003	0.02607676
ILMN_1232668	MAD	1.110784132	0.0002995	0.01489206	0.0002995	0.01335371	0.00026552	0.01324676	0.00020949	0.011649906	1	0.000212368	0.01604723
ILMN_2593774	1190002H23RIK	1.110704	0.0002995	0.01489206	0.0002995	0.01335371	0.00000489	0.00074347	0.00000986	0.001403781	1	6.47271E-05	0.00803428
ILMN_2559724	A130014O09RIK	1.110598097	0.01142074	0.12058349	0.00876432	0.10074305	0.00510266	0.08281496	0.00678734	0.095622483	0	0.005692914	0.10310819
ILMN_3002505	ABHD8	1.110530454	0.00094262	0.02607154	0.00094262	0.02430167	0.0000941	0.00638343	0.00019979	0.011313575	1	0.000540727	0.02850419
ILMN_2441724	9430014F16RIK	1.110454831	0.0006399	0.0211535	0.0006399	0.01941092	0.00023109	0.01201016	0.00020319	0.011438788	1	0.000665544	0.03206944
ILMN_1229005	TRAT1	1.110421282	0.0006399	0.0211535	0.0006399	0.01941092	0.00027271	0.01347531	0.00019498	0.011135911	1	0.001104043	0.04229821
ILMN_1229518	B230340J04RIK	1.11037499	0.0002995	0.01489206	0.0002995	0.01335371	0.00000774	0.0010503	0.00000785	0.001234703	1	9.56597E-06	0.00274856
ILMN_2576568	D130062J21RIK	1.110225832	0.00176049	0.0385938	0.00276802	0.04845153	0.0029345	0.06072226	0.00575947	0.08793067	0	0.004510321	0.09155774
ILMN_2491182	A130010C12RIK	1.110126472	0.01426212	0.13687557	0.0179397	0.15296839	0.0043924	0.07547173	0.01868086	0.168124091	0	0.037563093	0.2654797
ILMN_2484278	9930031P18RIK	1.110094091	0.00252097	0.04839275	0.00301234	0.05155233	0.00533746	0.08531507	0.00968357	0.116862873	0	0.012022966	0.14955451
ILMN_2827780	D14ERTD668E	1.11008286	0.0006399	0.0211535	0.0002995	0.01335371	0.0000427	0.00354604	0.0000575	0.004674319	1	0.000642967	0.03157309
ILMN_3112873	TXNIP	1.110031984	0.00094262	0.02607154	0.0006399	0.01941092	0.0001431	0.00861123	0.0002438	0.012729351	1	0.001141234	0.04287409
ILMN_3117602	CD6	1.109929013	0.0002995	0.01489206	0.0002995	0.01335371	0.00000663	0.00094987	0.0000261	0.002837023	1	0.000197163	0.01547711
ILMN_1233424	LBCL1	1.109898196	0.0002995	0.01489206	0.0002995	0.01335371	0.0000427	0.00354604	0.0000532	0.004485638	1	0.000103509	0.01061242
ILMN_2681601	SLC44A2	1.109888463	0.010097	0.11285122	0.00854165	0.09937884	0.00254344	0.05584417	0.0068766	0.096328868	0	0.009432203	0.13248473
ILMN_2762956	SPCS2	1.109838126	0.0002995	0.01489206	0.0002995	0.01335371	0.0000411	0.00346811	0.00013104	0.00859081	1	0.000189968	0.01508948
ILMN_2632230	D2ERTD391E	1.109720922	0.0002995	0.01489206	0.0002995	0.01335371	0.00000699	0.00098038	0.0000266	0.002856495	1	0.000317538	0.02078825

ILMN_3052501	CD27	1.109550882	0.00094262	0.02607154	0.0006399	0.01941092	0.0000571	0.00436027	0.00011777	0.007898943	1	0.000966442	0.03935694
ILMN_2615096	DPP4	1.109531905	0.0002995	0.01489206	0.0002995	0.01335371	4.24E-07	0.00011758	2.57E-07	0.000106375	1	2.49646E-06	0.00133566
ILMN_2629112	ASAH3L	1.109478785	0.0002995	0.01489206	0.0002995	0.01335371	0.0000279	0.00260327	0.0000227	0.002596277	1	3.53859E-05	0.0058616
ILMN_1254916	LOC386508	1.109314765	0.00094262	0.02607154	0.0006399	0.01941092	0.0000268	0.00252935	0.00029189	0.014258943	1	0.002106509	0.05997046
ILMN_2686115	RAPGEF6	1.109219239	0.00605844	0.08369461	0.00444101	0.06659434	0.00124244	0.03553479	0.00286075	0.059099453	0	0.008219665	0.12365894
ILMN_3153010	SLITRK5	1.109095163	0.0002995	0.01489206	0.0002995	0.01335371	0.00015814	0.00910989	0.0000467	0.004101953	1	5.97852E-05	0.00777325
ILMN_2571616	C430002D13RIK	1.109088506	0.00122569	0.03054155	0.0020184	0.03953445	0.00120471	0.03493809	0.00223335	0.05034168	0	0.001797898	0.05487657
ILMN_1231785	HDAC2	1.10904434	0.0006399	0.0211535	0.0002995	0.01335371	0.00011044	0.00713983	0.00026456	0.013458572	1	0.000237231	0.01736224
ILMN_1221831	TCFAP2E	1.10874714	0.00176049	0.0385938	0.00176049	0.03635757	0.0006064	0.02285402	0.00061856	0.023351316	1	0.001344859	0.0469611
ILMN_2646456	RNPC2	1.108556752	0.00397039	0.064249	0.00349473	0.0566205	0.00206214	0.04929958	0.00297458	0.060346491	0	0.003346676	0.07734743
ILMN_2504686	MT-ATP6	1.108358727	0.00876432	0.1043511	0.00898742	0.10212898	0.006843	0.09776578	0.01129715	0.127943658	0	0.013719454	0.16025079
ILMN_2712867	TIMP2	1.108193843	0.0002995	0.01489206	0.0002995	0.01335371	0.0000462	0.00374496	0.0000657	0.005137493	1	5.74504E-05	0.00761864
ILMN_1234006	LOC277136	1.10816163	0.00373333	0.06184411	0.0020184	0.03953445	0.0000764	0.00546326	0.00034246	0.015876812	0	0.001104095	0.04229821
ILMN_2965669	XLR4A	1.108141291	0.0002995	0.01489206	0.0002995	0.01335371	0.00000486	0.00074167	0.00000396	0.000746457	1	1.23984E-05	0.00322459
ILMN_2447538	2210018M11RIK	1.10801562	0.0006399	0.0211535	0.0006399	0.01941092	0.0000305	0.00278714	0.0000653	0.005121772	1	0.000306263	0.02048222
ILMN_1222917	PTPN1	1.107980121	0.0032539	0.05678214	0.00373333	0.05925673	0.00204418	0.04892042	0.00362761	0.068031509	0	0.007351981	0.11667667
ILMN_1255086	2810454F19RIK	1.107686745	0.0067415	0.08943186	0.00651414	0.08446632	0.00242497	0.05418861	0.00487539	0.080587642	0	0.007351739	0.11667667
ILMN_1233554	PBRM1	1.107679536	0.00149689	0.03478153	0.00094262	0.02430167	0.00059016	0.02253483	0.00040478	0.01751061	1	0.000844832	0.03651291
ILMN_1234072	PDLIM1	1.107488297	0.0002995	0.01489206	0.0002995	0.01335371	0.0000459	0.00372562	0.0000726	0.005551592	1	0.000306263	0.02048222
ILMN_1216561	CAMK2D	1.107443275	0.00122569	0.03054155	0.00094262	0.02430167	0.00076398	0.02667747	0.00134692	0.037363102	1	0.00305523	0.07341857
ILMN_2590638	BCL2L11	1.107365706	0.00122569	0.03054155	0.00122569	0.02881591	0.0000915	0.00623516	0.0000482	0.004197605	1	0.000176289	0.01444673
ILMN_2705878	LIMD2	1.107359113	0.0002995	0.01489206	0.0002995	0.01335371	0.00000896	0.00116209	0.00000904	0.001335714	1	6.22097E-05	0.00789013
ILMN_1227100	E130112E08RIK	1.107230432	0.0020184	0.04200694	0.00276802	0.04845153	0.00208027	0.04952957	0.00135255	0.037429791	1	0.00040803	0.02413289
ILMN_1254814	CLK1	1.10722634	0.00094262	0.02607154	0.00094262	0.02430167	0.00022953	0.01197773	0.00041438	0.017763501	1	0.00099928	0.0402366
ILMN_2986458	TERF2IP	1.107174717	0.0002995	0.01489206	0.0002995	0.01335371	0.00036565	0.01644438	0.00025266	0.013009278	1	0.000470028	0.02607676
ILMN_1239729	KIF1B	1.106854993	0.0006399	0.0211535	0.0006399	0.01941092	0.00137718	0.03773799	0.00099268	0.031178183	1	0.000504223	0.02732257
ILMN_1217480	LOC100046080	1.106833133	0.00696781	0.09115463	0.00490758	0.07073729	0.00117721	0.03450589	0.00170887	0.042787906	0	0.004379328	0.09051712
ILMN_2652857	IFI47	1.106721599	0.00943231	0.10825947	0.0076447	0.09407685	0.0082845	0.10948849	0.00387692	0.070436711	0	0.006383169	0.10923805
ILMN_1257107	LOC100043821	1.106674621	0.0006399	0.0211535	0.0006399	0.01941092	0.00054092	0.02135537	0.00117775	0.034456682	1	0.001179524	0.04378253
ILMN_2636339	IRF9	1.106644992	0.00252097	0.04839275	0.00094262	0.02430167	0.00029795	0.01428302	0.00076743	0.026757653	1	0.00270263	0.06885567
ILMN_2479848	SIF1	1.106503824	0.0002995	0.01489206	0.0002995	0.01335371	3.51E-08	0.0000177	2.82E-07	0.000110081	1	5.89482E-06	0.0021116
ILMN_1226514	GAB3	1.106492684	0.0002995	0.01489206	0.0002995	0.01335371	0.00000136	0.0002775	2.37E-07	0.000101599	1	6.1646E-06	0.0021257
ILMN_3141048	SEPP1	1.106305884	0.00149689	0.03478153	0.00094262	0.02430167	0.00028342	0.01382831	0.0001664	0.009994028	1	0.000643	0.03157309
ILMN_2635871	3110057O12RIK	1.106105831	0.0006399	0.0211535	0.0006399	0.01941092	0.00015081	0.00885986	0.00020499	0.011473406	1	0.000643034	0.03157309
ILMN_2829625	NTNG1	1.106001213	0.00122569	0.03054155	0.00122569	0.02881591	0.00014765	0.00875482	0.00041707	0.017826837	1	0.000438006	0.02507734
ILMN_2635132	FOXP3	1.105736567	0.0006399	0.0211535	0.0002995	0.01335371	0.00000368	0.00061365	0.00000951	0.00136995	1	0.001301631	0.04617913
ILMN_1225109	2310047C21RIK	1.105585656	0.00094262	0.02607154	0.00094262	0.02430167	0.00023145	0.01201016	0.00046395	0.019281568	1	0.000559888	0.02915112
ILMN_1228653	ZBP1	1.105391357	0.00898742	0.10547593	0.00809366	0.09652695	0.00364092	0.06763268	0.00593208	0.089284737	0	0.011706901	0.14779526
ILMN_1236585	LOC634213	1.105197029	0.0032539	0.05678214	0.00149689	0.03284241	0.00010667	0.00697357	0.00036187	0.016337543	0	0.001797898	0.05487657
ILMN_1227803	LOC674707	1.105118227	0.0006399	0.0211535	0.00094262	0.02430167	0.00000863	0.00113739	0.0000456	0.00403955	1	7.28705E-05	0.00865281
ILMN_2706631	LOC100048076	1.105000545	0.0002995	0.01489206	0.0002995	0.01335371	5.68E-07	0.00014379	0.00000114	0.000290477	1	7.68769E-06	0.0024016
ILMN_2949605	UBAC2	1.104949833	0.0002995	0.01489206	0.0002995	0.01335371	4.68E-07	0.00012677	0.00000161	0.000370841	1	7.28705E-05	0.00865281
ILMN_2651054	LOC100047173	1.104947245	0.00122569	0.03054155	0.00094262	0.02430167	0.0001854	0.01012894	0.00020712	0.011559512	1	0.002540648	0.06640064
ILMN_2691192	RBBP7	1.104851088	0.00741953	0.09487807	0.01186014	0.12006629	0.0247443	0.19992306	0.01812994	0.165469515	0	0.013719454	0.16025079
ILMN_2569336	A130024J05RIK	1.104817015	0.00965407	0.11001819	0.01031818	0.11074271	0.00502937	0.08208706	0.00762986	0.10204046	0	0.012022966	0.14955451
ILMN_1222346	A630097D09RIK	1.104807842	0.0002995	0.01489206	0.0002995	0.01335371	0.00000699	0.00098038	0.0000116	0.001590823	1	1.60246E-05	0.00367466
ILMN_1255419	ZFPN1A1	1.10475855	0.00122569	0.03054155	0.00122569	0.02881591	0.00023251	0.01202516	0.00046107	0.019196381	1	0.001633268	0.05224999

ILMN_2704822	ACAA2	1.104751617	0.00227139	0.04545806	0.00227139	0.0429166	0.00073722	0.02599654	0.00150758	0.039781954	1	0.002041151	0.05875662
ILMN_2595967	LOC100039532	1.104726083	0.0002995	0.01489206	0.0002995	0.01335371	0.0000399	0.00337413	0.0000835	0.006132211	1	0.000353719	0.02219068
ILMN_2628174	ACSS1	1.104609544	0.0002995	0.01489206	0.0002995	0.01335371	0.00000673	0.00095405	0.00000329	0.000651535	1	5.51982E-05	0.00760204
ILMN_2457324	TSSC1	1.10460787	0.00094262	0.02607154	0.0006399	0.01941092	0.00037762	0.01683607	0.00068122	0.024908549	1	0.001301631	0.04617913
ILMN_2631994	HEXIM1	1.104522651	0.0002995	0.01489206	0.0002995	0.01335371	7.94E-07	0.00018585	5.26E-07	0.000168884	1	3.28593E-06	0.00154495
ILMN_2544890	PDE4B	1.104516163	0.00276802	0.05133777	0.0032539	0.05432415	0.00144645	0.03905335	0.00467606	0.078680163	0	0.00388914	0.08408165
ILMN_2696491	PHF11	1.104484414	0.00420612	0.06669083	0.00252097	0.04602629	0.00130622	0.0366489	0.00177754	0.04368478	0	0.003888993	0.08408165
ILMN_2775098	CYB5	1.104384533	0.0002995	0.01489206	0.0002995	0.01335371	0.00000016	0.0000542	3.92E-07	0.00013797	1	5.89742E-06	0.0021116
ILMN_2880467	LASS4	1.104325447	0.00227139	0.04545806	0.00176049	0.03635757	0.00059615	0.02265805	0.00077485	0.026955849	1	0.00099928	0.0402366
ILMN_2812244	MPPE1	1.104198108	0.0002995	0.01489206	0.0002995	0.01335371	0.00000576	0.00084519	0.00000933	0.001361621	1	4.33673E-05	0.0065968
ILMN_3133748	GAB3	1.1040901	0.00149689	0.03478153	0.00122569	0.02881591	0.000026	0.00247667	0.0001002	0.00697141	1	0.000600122	0.03046224
ILMN_2518828	ZNF24	1.104037027	0.0006399	0.0211535	0.0002995	0.01335371	0.0000988	0.00658207	0.00018603	0.010797146	1	0.00011618	0.0112429
ILMN_1258965	TMEM66	1.10397338	0.00094262	0.02607154	0.00094262	0.02430167	0.00012203	0.00760514	0.00027116	0.013615504	1	0.0007895	0.03543767
ILMN_1237886	ENC1	1.103956093	0.0006399	0.0211535	0.0002995	0.01335371	0.0000265	0.00250371	0.0000379	0.003587283	1	0.000135361	0.01242731
ILMN_2492102	LOC630408	1.103768241	0.0006399	0.0211535	0.0002995	0.01335371	0.00021339	0.0113638	0.00034026	0.015806312	1	0.000966442	0.03935694
ILMN_2710166	DDX3X	1.103764891	0.0051392	0.07619761	0.00467484	0.06877491	0.00321609	0.06358903	0.00370058	0.068707988	0	0.004510321	0.09155774
ILMN_2742928	FXYD5	1.103662574	0.0198742	0.16408427	0.01491383	0.13744404	0.00597998	0.09087506	0.00889638	0.111496961	0	0.013017012	0.15603988
ILMN_3071525	CD226	1.103645914	0.00349473	0.05908105	0.0020184	0.03953445	0.00073613	0.02597776	0.00079434	0.027327392	0	0.001977658	0.05767797
ILMN_2720820	A230046K03RIK	1.103616266	0.0002995	0.01489206	0.0002995	0.01335371	0.00000291	0.00051317	0.0000077	0.001222667	1	8.19763E-05	0.00930671
ILMN_1238069	4930539E08RIK	1.103535154	0.0002995	0.01489206	0.0002995	0.01335371	0.0000032	0.00055228	0.0000217	0.002508955	1	0.000157497	0.01350097
ILMN_1238285	DCUN1D5	1.103527312	0.00149689	0.03478153	0.00149689	0.03284241	0.00034811	0.01594848	0.00028909	0.014164441	1	0.000284812	0.01943975
ILMN_1234133	3632431M01RIK	1.103406406	0.00094262	0.02607154	0.0006399	0.01941092	0.00032	0.01501554	0.00049525	0.020203747	1	0.000284828	0.01943975
ILMN_1217705	C130002K18RIK	1.103274712	0.00831809	0.10165638	0.01031818	0.11074271	0.00823865	0.10909937	0.01077307	0.124430321	0	0.015225452	0.168939
ILMN_2614966	RAB27A	1.103217796	0.0002995	0.01489206	0.0002995	0.01335371	8.05E-08	0.0000329	2.31E-08	0.0000187	1	1.2393E-06	0.00090134
ILMN_1245393	STXBP3	1.103174614	0.00397039	0.064249	0.00227139	0.0429166	0.0011527	0.03421849	0.00190089	0.045691414	0	0.00553098	0.10148188
ILMN_1239726	SNAI3	1.103168493	0.0006399	0.0211535	0.0006399	0.01941092	0.0000322	0.00289276	0.00011372	0.007705253	1	0.000665544	0.03206944
ILMN_1223028	D230020C06RIK	1.103101789	0.0006399	0.0211535	0.0006399	0.01941092	0.00028646	0.01388928	0.00034886	0.016068328	1	0.000873828	0.03714525
ILMN_2854878	NECAP1	1.10302603	0.0002995	0.01489206	0.0002995	0.01335371	0.00000877	0.00114799	0.0000229	0.002603706	1	8.19763E-05	0.00930671
ILMN_1242802	RAB6	1.103017062	0.00094262	0.02607154	0.0006399	0.01941092	0.00061393	0.02295322	0.00031887	0.015112162	1	0.001856078	0.05577459
ILMN_2535779	LOC383706	1.102993315	0.0006399	0.0211535	0.00094262	0.02430167	0.00056898	0.02197867	0.00070261	0.025381142	1	0.001104095	0.04229821
ILMN_1228235	CTSE	1.102982206	0.00094262	0.02607154	0.00094262	0.02430167	0.00029079	0.01407	0.00018402	0.010707081	1	0.00057968	0.02968357
ILMN_3163163	A430078G23RIK	1.102898707	0.0006399	0.0211535	0.0006399	0.01941092	0.00013728	0.00835294	0.00013654	0.008839388	1	0.00022037	0.01646479
ILMN_2732190	PBRM1	1.102890972	0.00605844	0.08369461	0.00397039	0.06196763	0.00114065	0.03401276	0.00113476	0.033772175	0	0.013719454	0.16025079
ILMN_1246770	YBX3	1.102889488	0.00349473	0.05908105	0.00301234	0.05155233	0.00358474	0.06731425	0.00316517	0.062746493	0	0.005858095	0.10440833
ILMN_1250135	A930005H10RIK	1.102857125	0.0006399	0.0211535	0.0006399	0.01941092	0.0000434	0.00359485	0.0000827	0.006091257	1	0.000329201	0.02128238
ILMN_3114641	PIK3R1	1.102785193	0.0002995	0.01489206	0.0002995	0.01335371	0.00000126	0.00026389	0.00000119	0.00029799	1	1.47177E-05	0.00353126
ILMN_2677056	LOC100048622	1.102768087	0.0020184	0.04200694	0.0020184	0.03953445	0.00246416	0.05479494	0.00260119	0.055447887	0	0.003774648	0.08295494
ILMN_1243042	LOC381214	1.102758352	0.0002995	0.01489206	0.0002995	0.01335371	0.00000214	0.0004015	0.0000184	0.002263583	1	4.33673E-05	0.0065968
ILMN_2917338	RNF125	1.102623511	0.00373333	0.06184411	0.0032539	0.05432415	0.00210043	0.0498312	0.00339438	0.065464235	0	0.00270263	0.06885567
ILMN_2433964	GIGYF1	1.102596985	0.00176049	0.0385938	0.00149689	0.03284241	0.00047117	0.01946027	0.00035965	0.0162751	1	0.00057968	0.02968357
ILMN_2566477	C730026O12RIK	1.102585731	0.00854165	0.10305721	0.01076004	0.11338366	0.00953803	0.11842272	0.01173891	0.130774947	0	0.012346572	0.15195555
ILMN_2704823	ACAA2	1.102584401	0.00176049	0.0385938	0.00122569	0.02881591	0.00099103	0.03127428	0.00059447	0.022718354	1	0.001104095	0.04229821
ILMN_1249863	SLAMF1	1.102358687	0.00149689	0.03478153	0.00094262	0.02430167	0.00053162	0.02111665	0.00018539	0.010773166	1	0.001686537	0.05325863
ILMN_2617820	PPP3CC	1.10232076	0.0002995	0.01489206	0.0002995	0.01335371	5.55E-07	0.00014264	7.19E-07	0.000216047	1	1.47177E-05	0.00353126
ILMN_1238109	PP11R	1.102299387	0.0002995	0.01489206	0.0002995	0.01335371	0.00010362	0.00682223	0.0000106	0.001483462	1	3.68613E-05	0.00599924
ILMN_1257463	BCL11A	1.102238843	0.00094262	0.02607154	0.00094262	0.02430167	0.00064732	0.02378135	0.00029644	0.014423405	1	0.001531347	0.05016159
ILMN_2634157	ZFP654	1.102206279	0.0002995	0.01489206	0.0002995	0.01335371	0.00000507	0.00076441	0.0000188	0.002298746	1	3.12891E-05	0.00543437

ILMN_1238869	LOC278666	1.102138175	0.00467484	0.07150478	0.00276802	0.04845153	0.0006377	0.02359297	0.00121677	0.035196389	0	0.00305523	0.07341857
ILMN_1215910	FOXN2	1.102070233	0.0002995	0.01489206	0.0002995	0.01335371	0.00074588	0.02612373	0.00053798	0.021230722	1	0.000438006	0.02507734
ILMN_2621086	FLI1	1.102009965	0.00122569	0.03054155	0.00149689	0.03284241	0.00147107	0.03946627	0.00070355	0.025381142	1	0.000873828	0.03714525
ILMN_2699531	RGS10	1.101989172	0.00227139	0.04545806	0.00149689	0.03284241	0.00051989	0.02080755	0.00041221	0.017749337	1	0.001068077	0.04167292
ILMN_2603187	MECP2	1.101917508	0.0002995	0.01489206	0.0002995	0.01335371	0.00000123	0.00026043	0.00000145	0.000347545	1	5.39498E-06	0.00202516
ILMN_2982781	KCNK5	1.101765886	0.0006399	0.0211535	0.0006399	0.01941092	0.0000223	0.00222523	0.0000282	0.002959627	1	0.000151664	0.01324485
ILMN_1244845	NARF	1.101738476	0.00094262	0.02607154	0.00094262	0.02430167	0.0000508	0.00397491	0.00015962	0.00973796	1	0.000453754	0.02560106
ILMN_2680398	ZC3H12D	1.10146721	0.0002995	0.01489206	0.0002995	0.01335371	0.000001	0.00021909	9.52E-07	0.000257701	1	7.03927E-06	0.00230764
ILMN_2605031	E330018D03RIK	1.101463118	0.0056004	0.08017457	0.00831809	0.09800443	0.00453712	0.07707642	0.01264569	0.136133148	0	0.010802479	0.14220108
ILMN_2617477	8030476J24	1.101261776	0.00122569	0.03054155	0.00122569	0.02881591	0.00015789	0.00910673	0.00017366	0.010310561	1	0.00010758	0.01083883
ILMN_2901284	ADD3	1.101189602	0.0002995	0.01489206	0.0002995	0.01335371	0.0000471	0.00380085	0.0000359	0.003445285	1	4.70154E-05	0.00688184
ILMN_2506499	UBE3A	1.101180473	0.00252097	0.04839275	0.00252097	0.04602629	0.00053169	0.02111665	0.00192593	0.046019516	1	0.004924874	0.09535694
ILMN_2605960	RAB19	1.101133403	0.0020184	0.04200694	0.00149689	0.03284241	0.00042802	0.01837561	0.00058953	0.022608846	1	0.001141234	0.04287409
ILMN_2562725	C730013O11RIK	1.101075632	0.00094262	0.02607154	0.00094262	0.02430167	0.00087707	0.02868922	0.00102151	0.031783537	1	0.000504223	0.02732257
ILMN_2567626	PUM1	1.101065176	0.00628646	0.08573635	0.0056004	0.0767776	0.00642098	0.09434264	0.0045757	0.077674245	0	0.012677869	0.1539569
ILMN_2954868	OASL2	1.101052773	0.00227139	0.04545806	0.00301234	0.05155233	0.00495144	0.08123106	0.00212755	0.048753581	0	0.0010331	0.04099743
ILMN_1233894	DTNB	1.100978861	0.0002995	0.01489206	0.0002995	0.01335371	0.00039474	0.01735374	0.00100272	0.0313667	1	0.00057968	0.02968357
ILMN_1229245	INPP4B	1.100970899	0.00227139	0.04545806	0.00176049	0.03635757	0.0004464	0.01884113	0.00062485	0.023550381	1	0.000873828	0.03714525
ILMN_1256639	CD247	1.100911079	0.0006399	0.0211535	0.0002995	0.01335371	0.0000315	0.0028568	0.0000379	0.003587283	1	0.000353739	0.02219068
ILMN_2631192	VPS26B	1.100779486	0.00467484	0.07150478	0.00227139	0.0429166	0.00243729	0.05437251	0.00115122	0.03404972	0	0.006029251	0.10646569
ILMN_2038768	control_ILMN_203	1.1007468	0.10926429	0.40116694	0.10721317	0.39734945	0.11759807	0.4332967	0.11009287	0.413665062	0	0.246250128	0.60756362
ILMN_2668977	1500032D16RIK	1.100684878	0.0002995	0.01489206	0.0002995	0.01335371	0.00000386	0.00063236	0.00000566	0.00097714	1	1.1877E-05	0.00314139
ILMN_1248211	SIDT1	1.100585537	0.00301234	0.05440113	0.0020184	0.03953445	0.00115892	0.03427199	0.00160715	0.041193921	0	0.002873882	0.07130914
ILMN_2534151	IRGB10	1.100553681	0.00537037	0.07818332	0.00628646	0.0826602	0.03005319	0.22151796	0.00905543	0.112610992	0	0.007994557	0.1216087
ILMN_1224211	EPB4.1L2	1.100521767	0.0051392	0.07619761	0.00696781	0.08825299	0.00796439	0.10708222	0.01547157	0.152284836	0	0.023367993	0.21022612
ILMN_2771182	H2-Q8	1.100477595	0.00252097	0.04839275	0.00176049	0.03635757	0.00048225	0.01977742	0.00025467	0.01309856	1	0.003246828	0.07602117
ILMN_2890357	2610027C15RIK	1.10037148	0.0006399	0.0211535	0.0006399	0.01941092	0.00044866	0.01889928	0.00040132	0.017418469	1	0.000559888	0.02915112
ILMN_2705732	INADL	1.100280563	0.00094262	0.02607154	0.00122569	0.02881591	0.00026059	0.01305657	0.00022306	0.012129499	1	0.000559888	0.02915112
ILMN_2422023	C330006D17RIK	1.10024538	0.00397039	0.064249	0.0051392	0.07282894	0.00529378	0.08485181	0.00649293	0.093577766	0	0.007351981	0.11667667
ILMN_1239398	LOC100046817	1.100205887	0.02094553	0.16993323	0.02372203	0.17961616	0.01005217	0.12237853	0.02557207	0.198492675	0	0.044021446	0.28499962
ILMN_1256245	RLF	1.100181996	0.00537037	0.07818332	0.00786961	0.09521779	0.00363532	0.06760424	0.00863224	0.109699863	0	0.005373592	0.10008988
ILMN_1243846	NRF1	1.100170193	0.00122569	0.03054155	0.00149689	0.03284241	0.00073562	0.02597776	0.00238171	0.052317743	0	0.001633268	0.05224999
ILMN_1242769	AKAP8L	1.100071536	0.00349473	0.05908105	0.00349473	0.0566205	0.00184551	0.04588832	0.0029622	0.060255548	0	0.002387591	0.06387081
ILMN_2993109	DDIT4	1.100038813	0.0002995	0.01489206	0.0002995	0.01335371	0.00000961	0.00121195	0.000042	0.003865058	1	0.000146035	0.01292295
ILMN_1245987	HNRNPK	1.100037736	0.01965954	0.16292455	0.0283949	0.19737141	0.02738494	0.21092553	0.04211374	0.257862175	0	0.051397104	0.30742496

~~~~~~

Probes with |Fold Change| < 1.10 not displayed

~~~~~~

ILMN_2715198	GOLGA2	-1.100056844	0.00719408	0.09294718	0.0076447	0.09407685	0.01094039	0.12840196	0.00946169	0.115200974	0	0.008929411	0.12924045
ILMN_2711045	SIRT5	-1.100175511	0.0006399	0.0211535	0.00094262	0.02430167	0.00048467	0.01985889	0.00026924	0.013583836	1	0.000264806	0.01861919
ILMN_2734856	A930009M04RIK	-1.10024481	0.0002995	0.01489206	0.0002995	0.01335371	0.00000347	0.00058692	0.00000793	0.001243596	1	1.97985E-05	0.00420804
ILMN_1253854	TGIF1	-1.100335725	0.01098073	0.11810945	0.00898742	0.10212898	0.00427564	0.07428823	0.00643797	0.093170157	0	0.017311316	0.18018556
ILMN_2769945	BC014795	-1.10036431	0.00094262	0.02607154	0.00094262	0.02430167	0.0000879	0.00605816	0.00020323	0.011438788	1	0.000522178	0.02800209
ILMN_2954195	SCYL1	-1.100565484	0.00809366	0.10014632	0.00651414	0.08446632	0.00354956	0.06694553	0.00616438	0.090932677	0	0.009177877	0.1304834
ILMN_2988572	EZH2	-1.100604827	0.00628646	0.08573635	0.00605844	0.08075955	0.00163133	0.0423263	0.00517364	0.083183907	0	0.011706901	0.14779526

ILMN_2728118	RRP12	-1.100673522	0.0002995	0.01489206	0.0002995	0.01335371	0.0000058	0.00084519	0.00000976	0.001398167	1	2.34093E-05	0.00450296
ILMN_1235470	HNRNPC	-1.100796556	0.0002995	0.01489206	0.0002995	0.01335371	2.8E-08	0.0000148	1.42E-07	0.0000709	1	4.50906E-06	0.00180983
ILMN_2774410	STFA1	-1.100797564	0.0336756	0.21913659	0.03956153	0.23673264	0.03507069	0.2404769	0.03975572	0.250087018	0	0.058515334	0.32634954
ILMN_2856095	ZC3H12A	-1.101004577	0.00149689	0.03478153	0.00094262	0.02430167	0.00016088	0.00919959	0.00033043	0.015473218	1	0.001259689	0.04555924
ILMN_2985447	CMTM7	-1.101081672	0.00094262	0.02607154	0.00094262	0.02430167	0.00036007	0.01627185	0.00030875	0.014785413	1	0.000438006	0.02507734
ILMN_2870487	CPNE2	-1.101235208	0.00301234	0.05440113	0.00176049	0.03635757	0.00015688	0.0090601	0.00033839	0.015735209	0	0.002173782	0.0607706
ILMN_2736762	IFRD2	-1.101280587	0.00943231	0.10825947	0.00898742	0.10212898	0.00427352	0.07427913	0.00677904	0.095608851	0	0.021171123	0.19968637
ILMN_2620061	TBCB	-1.101312875	0.0002995	0.01489206	0.0002995	0.01335371	0.0000296	0.00274031	0.0000316	0.003180587	1	9.5799E-05	0.01018072
ILMN_1252016	ABHD2	-1.101327755	0.00176049	0.0385938	0.0006399	0.01941092	0.0000452	0.003705	0.00013411	0.00874694	1	0.000453754	0.02560106
ILMN_2683802	NUDT9	-1.101534614	0.0002995	0.01489206	0.0002995	0.01335371	0.00000633	0.00091523	0.0000127	0.001692242	1	7.57938E-05	0.00879794
ILMN_2758087	CCNT1	-1.101561438	0.00349473	0.05908105	0.0032539	0.05432415	0.00117117	0.034443	0.00137359	0.03783222	0	0.003663228	0.08166297
ILMN_1222246	ADFP	-1.101682242	0.00943231	0.10825947	0.00854165	0.09937884	0.00289096	0.0601428	0.00520412	0.083438223	0	0.013017012	0.15603988
ILMN_1250149	DNMT3L	-1.101692032	0.00094262	0.02607154	0.0002995	0.01335371	0.0000385	0.00328743	0.000081	0.006004936	1	0.000135361	0.01242731
ILMN_2855423	UBE3B	-1.101823534	0.00467484	0.07150478	0.00444101	0.06659434	0.00097578	0.0310015	0.00261887	0.055581773	0	0.011096803	0.14428014
ILMN_3093150	NPM3	-1.101881244	0.01076004	0.11696153	0.01207886	0.12146481	0.00748853	0.10343287	0.01078041	0.12447925	0	0.018679697	0.18694557
ILMN_2887208	RAB1B	-1.1018898	0.00741953	0.09487807	0.00467484	0.06877491	0.00211426	0.04998366	0.00162135	0.041443788	0	0.005220076	0.09861156
ILMN_1246622	9130204C11RIK	-1.102206651	0.00094262	0.02607154	0.0006399	0.01941092	0.0000832	0.0058247	0.0000636	0.005055758	1	0.000246108	0.01770572
ILMN_1237507	DLST	-1.102242835	0.0002995	0.01489206	0.0002995	0.01335371	0.0000239	0.0023395	0.0000261	0.002837023	1	4.70154E-05	0.00688184
ILMN_2615739	GM459	-1.102378757	0.01750895	0.15321051	0.0211598	0.1686674	0.01902429	0.17493554	0.02815215	0.207735874	0	0.018679697	0.18694557
ILMN_2605767	REPIN1	-1.102407679	0.0002995	0.01489206	0.0002995	0.01335371	0.00047865	0.01968165	0.0000565	0.004637506	1	0.000120719	0.01149104
ILMN_2675033	SLC9A7	-1.102560823	0.0002995	0.01489206	0.0002995	0.01335371	0.0000189	0.00194817	0.0000376	0.003576126	1	9.5799E-05	0.01018072
ILMN_2531520	GM962	-1.10277933	0.0006399	0.0211535	0.0006399	0.01941092	0.0000441	0.00363684	0.0000899	0.006457872	1	0.000393771	0.02371136
ILMN_2952587	INTS5	-1.102916647	0.01469649	0.13944707	0.01273594	0.12533842	0.00646817	0.09479661	0.00750925	0.101343301	0	0.008450429	0.12538799
ILMN_3162081	WDR6	-1.103000504	0.00149689	0.03478153	0.0020184	0.03953445	0.00047402	0.01954308	0.00088629	0.029245777	1	0.000934606	0.03863507
ILMN_2823778	SC4MOL	-1.103118657	0.00094262	0.02607154	0.0002995	0.01335371	0.00060963	0.02290284	0.00035368	0.016129651	1	0.000176289	0.01444673
ILMN_2637804	4933407P14RIK	-1.103123645	0.02775949	0.19711952	0.02881875	0.19934791	0.03335811	0.23451441	0.02708149	0.204795156	0	0.03192796	0.24475474
ILMN_2944939	CAMK1	-1.103291951	0.0002995	0.01489206	0.0002995	0.01335371	0.0000298	0.00274909	0.0000185	0.002263791	1	3.12891E-05	0.00543437
ILMN_2966386	RCL1	-1.10330649	0.0006399	0.0211535	0.0006399	0.01941092	0.00000151	0.00030356	0.00000531	0.000929895	1	8.86216E-05	0.00982291
ILMN_2721208	MSTO1	-1.103313852	0.0076447	0.09672601	0.0051392	0.07282894	0.00087596	0.02868922	0.00154119	0.04014427	0	0.005531174	0.10148188
ILMN_1243107	CENTD3	-1.103383796	0.0006399	0.0211535	0.0006399	0.01941092	0.0000139	0.00158406	0.0000239	0.002688276	1	0.000204639	0.01592867
ILMN_2491232	USP36	-1.103389463	0.0020184	0.04200694	0.00149689	0.03284241	0.00016003	0.00916202	0.00017844	0.010486465	1	0.003055109	0.07341857
ILMN_1259100	PILRB1	-1.103436708	0.00490758	0.07399298	0.00301234	0.05155233	0.00088463	0.0288961	0.0019523	0.046411523	0	0.002173782	0.0607706
ILMN_1225747	ZNFN1A3	-1.103489414	0.0002995	0.01489206	0.0002995	0.01335371	0.0000072	0.00099242	0.0000278	0.002931426	1	0.000453754	0.02560106
ILMN_2721149	ARL11	-1.103567882	0.0032539	0.05678214	0.00349473	0.0566205	0.00078155	0.02696727	0.00091667	0.029754618	0	0.002620495	0.06768933
ILMN_1244424	ADORA2B	-1.103675196	0.00420612	0.06669083	0.00252097	0.04602629	0.00070446	0.02533145	0.00066071	0.024405921	0	0.00388914	0.08408165
ILMN_1228832	NGP	-1.103786521	0.01295435	0.12973001	0.01076004	0.11338366	0.00414593	0.07330247	0.00400122	0.071825953	0	0.011398229	0.14607747
ILMN_1260456	HSD3B7	-1.103805679	0.0002995	0.01489206	0.0002995	0.01335371	8.64E-07	0.00019904	0.00000221	0.000472255	1	2.15311E-05	0.0043202
ILMN_1227235	TIMM10	-1.103984846	0.00122569	0.03054155	0.00122569	0.02881591	0.0003537	0.01612517	0.0005671	0.0219992	1	0.000763113	0.03482416
ILMN_2871749	EAR6	-1.10402136	0.0032539	0.05678214	0.00490758	0.07073729	0.00962513	0.11918617	0.00641492	0.093049294	0	0.005220076	0.09861156
ILMN_2911344	PLSCR1	-1.104115255	0.0002995	0.01489206	0.0002995	0.01335371	0.00055175	0.02156346	0.00011527	0.007776142	1	0.000329201	0.02128238
ILMN_2698494	NFU1	-1.104319371	0.00122569	0.03054155	0.00122569	0.02881591	0.00101187	0.03167407	0.00130944	0.036673066	1	0.001301631	0.04617913
ILMN_2603898	CLEC4B1	-1.104355951	0.00149689	0.03478153	0.0006399	0.01941092	0.00047724	0.01964113	0.00013919	0.008973291	1	0.00042277	0.02466001
ILMN_2749448	PLEKHO2	-1.104536512	0.00149689	0.03478153	0.00149689	0.03284241	0.0000583	0.00441806	0.00017802	0.010475755	1	0.000600091	0.03046224
ILMN_2631014	PIAS3	-1.104655312	0.0002995	0.01489206	0.0002995	0.01335371	0.00000858	0.00113395	0.0000309	0.003134864	1	3.39668E-05	0.00570777
ILMN_2595863	CHST10	-1.10468852	0.0002995	0.01489206	0.0002995	0.01335371	0.00011639	0.00743132	0.00030356	0.01463008	1	0.00022037	0.01646479
ILMN_1231439	AATK	-1.104893386	0.00227139	0.04545806	0.00149689	0.03284241	0.0000541	0.00416529	0.00025931	0.013278569	1	0.003449313	0.07881943
ILMN_2923607	PHLDA3	-1.105141716	0.0006399	0.0211535	0.0002995	0.01335371	0.0000359	0.00314503	0.0000324	0.003223303	1	5.09531E-05	0.00725295

ILMN_2544343	STAB1	-1.105676499	0.0002995	0.01489206	0.0002995	0.01335371	0.00000574	0.00084519	0.0000233	0.002627969	1	0.000176289	0.01444673
ILMN_1238102	IDE	-1.105690099	0.04959771	0.27047142	0.05750633	0.289534	0.03780343	0.24938156	0.06636787	0.321827801	0	0.059776798	0.32882056
ILMN_2834198	SCN1B	-1.106256844	0.0002995	0.01489206	0.0006399	0.01941092	0.00023767	0.01217572	0.00044649	0.018790822	1	0.000453754	0.02560106
ILMN_1236702	LILRB4	-1.10637923	0.01076004	0.11696153	0.00898742	0.10212898	0.00224068	0.05176032	0.00387533	0.070435395	0	0.017311316	0.18018556
ILMN_2979639	H2-DMB2	-1.106407472	0.00227139	0.04545806	0.0020184	0.03953445	0.00172654	0.04391533	0.00099673	0.031258932	1	0.00270263	0.06885567
ILMN_1256817	SLPI	-1.106517473	0.04395927	0.25213143	0.03008794	0.20371558	0.01322187	0.14325168	0.01654123	0.157798872	0	0.038432634	0.26812964
ILMN_2590350	UBE2F	-1.106602174	0.00094262	0.02607154	0.00094262	0.02430167	0.00030653	0.0145343	0.000065	0.005110515	1	0.000157497	0.01350097
ILMN_1240318	SLC7A7	-1.106705276	0.0002995	0.01489206	0.0002995	0.01335371	0.00037337	0.01669711	0.0005249	0.020878705	1	0.000470028	0.02607676
ILMN_1215893	ALG3	-1.106724214	0.0002995	0.01489206	0.0002995	0.01335371	0.00000791	0.00106049	0.00000859	0.001299233	1	0.000212368	0.01604723
ILMN_2780915	PRMT7	-1.106850835	0.00149689	0.03478153	0.00122569	0.02881591	0.00043663	0.01861404	0.0006752	0.024769672	1	0.002387492	0.06387081
ILMN_2746830	BC022224	-1.106855817	0.00397039	0.064249	0.0020184	0.03953445	0.00012205	0.00760514	0.000874	0.02899696	0	0.005858907	0.10440833
ILMN_3120014	SRI	-1.106861448	0.00876432	0.1043511	0.01053919	0.11232356	0.01267052	0.13982328	0.01322195	0.139651023	0	0.029744406	0.23776625
ILMN_2959292	UPP1	-1.107047972	0.0067415	0.08943186	0.00831809	0.09800443	0.00616669	0.09223153	0.00737179	0.100219589	0	0.008450429	0.12538799
ILMN_2633457	1110057K04RIK	-1.107066336	0.0006399	0.0211535	0.0006399	0.01941092	0.0002621	0.01311825	0.00018047	0.010552902	1	0.000688785	0.03268184
ILMN_2646209	CDK10	-1.107106695	0.01164003	0.12184595	0.00965407	0.10652405	0.00837294	0.10993164	0.00597071	0.089478328	0	0.004644853	0.0931111
ILMN_2680440	ATP6V1B2	-1.107126066	0.00122569	0.03054155	0.00094262	0.02430167	0.0000173	0.00183184	0.00015479	0.009492492	1	0.00057965	0.02968357
ILMN_2691706	SAC3D1	-1.107157088	0.00227139	0.04545806	0.0020184	0.03953445	0.00059451	0.02265805	0.00187868	0.045262385	1	0.001531347	0.05016159
ILMN_1241909	ATL3	-1.107229662	0.00176049	0.0385938	0.0002995	0.01335371	0.0001247	0.00774952	0.0002357	0.012495554	1	0.001301631	0.04617913
ILMN_2686975	FAM129B	-1.107251043	0.00094262	0.02607154	0.0006399	0.01941092	0.00010016	0.00665043	0.0000642	0.005083088	1	0.000621233	0.03109305
ILMN_2900557	KIF15	-1.107418991	0.0056004	0.08017457	0.00252097	0.04602629	0.00050815	0.02053213	0.00073746	0.02616347	0	0.002314175	0.06295874
ILMN_2745954	1110007L15RIK	-1.107513874	0.0006399	0.0211535	0.0002995	0.01335371	0.00025743	0.01295401	0.00026747	0.013561762	1	0.000284828	0.01943975
ILMN_1226372	RFFL	-1.107562515	0.00094262	0.02607154	0.0006399	0.01941092	0.0000159	0.00174469	0.000055	0.004568149	1	0.000379979	0.02324165
ILMN_3084818	NR2C2AP	-1.107652217	0.00227139	0.04545806	0.00122569	0.02881591	0.00042107	0.01819815	0.00097889	0.030933174	1	0.001797898	0.05487657
ILMN_1248733	PA2G4	-1.107667438	0.00176049	0.0385938	0.00252097	0.04602629	0.00225915	0.05195492	0.0039532	0.071295686	0	0.002787111	0.07008733
ILMN_2630605	FSCN1	-1.107768152	0.00809366	0.10014632	0.00605844	0.08075955	0.0018293	0.04560708	0.00255619	0.054799056	0	0.007351981	0.11667667
ILMN_3003631	PPAP2C	-1.10780952	0.00276802	0.05133777	0.00276802	0.04845153	0.00099443	0.03132914	0.00125441	0.035750241	0	0.000934606	0.03863507
ILMN_2598775	STARD3NL	-1.107943892	0.00176049	0.0385938	0.00149689	0.03284241	0.0000601	0.00452448	0.00015332	0.009469751	1	0.000737547	0.0342621
ILMN_2588995	RAB8B	-1.108140051	0.00467484	0.07150478	0.0020184	0.03953445	0.00061097	0.02292456	0.00068182	0.024911182	0	0.00099904	0.0402366
ILMN_2936468	5730596K20RIK	-1.108252625	0.0002995	0.01489206	0.0002995	0.01335371	8.48E-07	0.00019639	0.00000286	0.000576709	1	3.26018E-05	0.00557911
ILMN_2699522	DYRK3	-1.108291839	0.0002995	0.01489206	0.0002995	0.01335371	0.0000346	0.00306253	0.0000326	0.003233591	1	2.65234E-05	0.00480383
ILMN_2611431	PPP3CA	-1.108544093	0.01901516	0.15981086	0.02308219	0.177055	0.0308581	0.22442614	0.03082622	0.218815938	0	0.043046488	0.28193118
ILMN_2908056	TMBIM1	-1.108696543	0.0002995	0.01489206	0.0002995	0.01335371	0.00035559	0.01617837	0.00070271	0.025381142	1	0.000844832	0.03651291
ILMN_2744909	GMPPB	-1.108708169	0.0006399	0.0211535	0.0006399	0.01941092	0.00218189	0.05098423	0.00160119	0.041131766	0	0.001218998	0.0445025
ILMN_2925281	ABCD3	-1.108715146	0.0002995	0.01489206	0.0002995	0.01335371	1.43E-07	0.0000497	1.83E-07	0.0000833	1	1.89069E-06	0.00111411
ILMN_2988931	STFA1	-1.108889714	0.01426212	0.13687557	0.01621381	0.14443088	0.0128175	0.14064502	0.01425719	0.145852642	0	0.022800994	0.20757243
ILMN_2819679	TMEM50A	-1.108893575	0.01664564	0.14895372	0.01858605	0.1562833	0.01881688	0.17402897	0.01897851	0.169539906	0	0.032684998	0.24738569
ILMN_2698430	BCL2L1	-1.109012645	0.00094262	0.02607154	0.00094262	0.02430167	0.00084298	0.02806722	0.00013557	0.008801061	1	0.000621233	0.03109305
ILMN_3137980	ZFP41	-1.109014707	0.0002995	0.01489206	0.0002995	0.01335371	0.0000051	0.00076551	0.00000777	0.001225434	1	3.25994E-05	0.00557911
ILMN_2914507	NDUFB6	-1.109148444	0.00628646	0.08573635	0.00628646	0.0826602	0.00323475	0.06374761	0.00475793	0.079602307	0	0.007994557	0.1216087
ILMN_1243329	DAB2	-1.109426935	0.01186014	0.12340942	0.00809366	0.09652695	0.0024452	0.05450994	0.00311624	0.062120568	0	0.029045675	0.23488344
ILMN_2640453	WDR55	-1.109433931	0.0006399	0.0211535	0.0006399	0.01941092	0.0000894	0.00612683	0.00012054	0.008038629	1	0.000237231	0.01736224
ILMN_2867696	CDC42BPB	-1.110032665	0.0002995	0.01489206	0.0002995	0.01335371	0.0000456	0.00372478	0.0001107	0.007522402	1	0.000379979	0.02324165
ILMN_2795412	TMEM176A	-1.110201644	0.0006399	0.0211535	0.0006399	0.01941092	0.0000316	0.00285855	0.0000256	0.002802168	1	8.86334E-05	0.00982291
ILMN_2727273	SWAP70	-1.110366306	0.0002995	0.01489206	0.0002995	0.01335371	0.00000132	0.00027167	0.0000026	0.000530708	1	2.44059E-05	0.00463717
ILMN_2620767	ABHD2	-1.110517436	0.0002995	0.01489206	0.0002995	0.01335371	0.00000256	0.00046493	0.00000565	0.00097714	1	4.33673E-05	0.0065968
ILMN_1221920	PLEKHG3	-1.110664539	0.0002995	0.01489206	0.0002995	0.01335371	0.0000203	0.00208152	0.0000086	0.001299233	1	1.74444E-05	0.00390377
ILMN_2764607	1810009N02RIK	-1.110668063	0.0002995	0.01489206	0.0002995	0.01335371	0.00000473	0.0007333	0.0000076	0.001211113	1	4.89467E-05	0.00705363

ILMN_2842767	NACC1	-1.110682075	0.0006399	0.0211535	0.0006399	0.01941092	0.00023804	0.01217572	0.00022635	0.012236289	1	0.00042277	0.02466001
ILMN_2755399	PPP2R4	-1.110682315	0.00276802	0.05133777	0.00227139	0.0429166	0.0021293	0.05017441	0.00148083	0.039320129	0	0.001531347	0.05016159
ILMN_1256354	GCLM	-1.110765414	0.00149689	0.03478153	0.00094262	0.02430167	0.0000865	0.00599563	0.0000573	0.004664893	1	7.57938E-05	0.00879794
ILMN_2981689	NUP133	-1.11080026	0.0002995	0.01489206	0.0002995	0.01335371	0.000013	0.00149316	0.00000117	0.000296995	1	7.35697E-06	0.00232956
ILMN_2915303	TREM3	-1.110935528	0.00094262	0.02607154	0.00122569	0.02881591	0.0001306	0.00798826	0.00024248	0.012705581	1	0.000228655	0.01684049
ILMN_1215209	CLECSF9	-1.110943822	0.0198742	0.16408427	0.02030331	0.16494841	0.01231585	0.13728535	0.01456125	0.147630924	0	0.016448649	0.17608907
ILMN_2841290	TNFAIP2	-1.110987512	0.0002995	0.01489206	0.0006399	0.01941092	0.00030838	0.01458748	0.00018794	0.010853565	1	0.000212368	0.01604723
ILMN_2959293	UPP1	-1.111007524	0.00149689	0.03478153	0.00149689	0.03284241	0.00100994	0.03163507	0.00135104	0.037410476	1	0.002314272	0.06295874
ILMN_2614563	APOL7C	-1.111021044	0.0002995	0.01489206	0.0002995	0.01335371	0.0000444	0.00364378	0.00014172	0.009037925	1	0.000237231	0.01736224
ILMN_3160203	ZFP213	-1.11102856	0.0006399	0.0211535	0.0006399	0.01941092	0.0000159	0.00174469	0.0000628	0.00500903	1	0.000306263	0.02048222
ILMN_2601379	MGC18837	-1.111045685	0.0002995	0.01489206	0.0002995	0.01335371	0.0000105	0.00128151	0.0000199	0.002370654	1	4.51564E-05	0.00675851
ILMN_1235000	YIF1A	-1.111117156	0.0006399	0.0211535	0.0006399	0.01941092	0.0000128	0.00148603	0.00000478	0.000855756	1	3.83949E-05	0.00609954
ILMN_2640995	SPIRE2	-1.111127251	0.0002995	0.01489206	0.0002995	0.01335371	1.43E-07	0.0000497	5.59E-07	0.000175929	1	5.39498E-06	0.00202516
ILMN_2884098	GORASP2	-1.111288278	0.0002995	0.01489206	0.0002995	0.01335371	0.00000784	0.00105936	0.0000312	0.003152395	1	7.28705E-05	0.00865281
ILMN_1256142	MARCKS	-1.111356803	0.00537037	0.07818332	0.00176049	0.03635757	0.0000741	0.00534833	0.00026102	0.01332625	0	0.000643034	0.03157309
ILMN_2601147	KDELC1	-1.111368149	0.00176049	0.0385938	0.0020184	0.03953445	0.00053473	0.0212191	0.00078635	0.027233771	1	0.001581552	0.05126497
ILMN_2680549	PIK3CB	-1.111474769	0.0002995	0.01489206	0.0002995	0.01335371	0.00025023	0.01267398	0.00045843	0.01916188	1	0.001686462	0.05325863
ILMN_1218181	IFITM6	-1.111515015	0.00122569	0.03054155	0.00122569	0.02881591	0.00094292	0.03016485	0.00037738	0.0167608	1	0.000643034	0.03157309
ILMN_2550095	6230425C21RIK	-1.111625769	0.00149689	0.03478153	0.00176049	0.03635757	0.00081345	0.02755739	0.00195454	0.046417419	1	0.003149701	0.07483876
ILMN_1224390	1700129I04RIK	-1.111956114	0.0002995	0.01489206	0.0002995	0.01335371	0.00000447	0.00070872	0.0000272	0.002888688	1	0.000189968	0.01508948
ILMN_1240746	MCPT4	-1.111968534	0.00444101	0.0691859	0.00176049	0.03635757	0.0001123	0.0072499	0.00028643	0.014095117	0	0.002173782	0.0607706
ILMN_1230423	SH3PXD2B	-1.111999219	0.0002995	0.01489206	0.0002995	0.01335371	0.0000155	0.00171658	0.00000487	0.000862773	1	7.88278E-05	0.00901523
ILMN_1247468	FTSJ3	-1.112004157	0.0002995	0.01489206	0.0002995	0.01335371	0.0000521	0.00404506	0.0000644	0.005086516	1	6.47271E-05	0.00803428
ILMN_3140913	TBC1D2	-1.112150855	0.0002995	0.01489206	0.0002995	0.01335371	0.0000389	0.00330101	0.00000834	0.001279253	1	0.000111802	0.01097902
ILMN_2519673	VWF	-1.112341924	0.0006399	0.0211535	0.0002995	0.01335371	0.0000321	0.00289074	0.0000414	0.003820132	1	9.21504E-05	0.00995203
ILMN_2617468	CHAC1	-1.112462827	0.00122569	0.03054155	0.00094262	0.02430167	0.00100678	0.03156276	0.00039234	0.017180018	1	0.000665544	0.03206944
ILMN_2450031	SCL0004020.1_31	-1.112588927	0.00094262	0.02607154	0.00094262	0.02430167	0.0000097	0.00121762	0.0000309	0.003134864	1	3.68613E-05	0.00599924
ILMN_2450155	IGLC2_J00595_IG_	-1.112608245	0.0002995	0.01489206	0.0002995	0.01335371	0.00000122	0.00025882	0.0000337	0.003292439	1	0.000317448	0.02078825
ILMN_1218104	5630401D24RIK	-1.112744368	0.0002995	0.01489206	0.0002995	0.01335371	0.00000346	0.00058692	0.00000396	0.000746457	1	5.74504E-05	0.00761864
ILMN_1237743	LOC382813	-1.112766578	0.0002995	0.01489206	0.0002995	0.01335371	6.54E-07	0.0001601	0.00000198	0.000436891	1	1.08955E-05	0.00300091
ILMN_1222872	WBSCR16	-1.112770151	0.00373333	0.06184411	0.00397039	0.06196763	0.00137828	0.03773799	0.00362037	0.068005458	0	0.004924874	0.09535694
ILMN_2687828	ARID3A	-1.112982962	0.00965407	0.11001819	0.00786961	0.09521779	0.00278855	0.05902629	0.00354237	0.067235737	0	0.011096803	0.14428014
ILMN_2772492	TSR1	-1.11339113	0.0002995	0.01489206	0.0002995	0.01335371	0.0000835	0.00583655	0.0000958	0.0067548	1	0.000146035	0.01292295
ILMN_2868480	EAR4	-1.11346403	0.06828805	0.3184975	0.07366518	0.32882749	0.05977412	0.31205079	0.07952314	0.35376248	0	0.035872649	0.25847743
ILMN_1219168	GPN1	-1.113513745	0.0002995	0.01489206	0.0002995	0.01335371	0.00012201	0.00760514	0.00045913	0.01916188	1	0.000621233	0.03109305
ILMN_1214608	PLXND1	-1.113701478	0.00094262	0.02607154	0.0006399	0.01941092	0.0000167	0.00178763	0.0000626	0.00500138	1	0.000816732	0.0360345
ILMN_2723708	FNBP1	-1.113872343	0.0002995	0.01489206	0.0002995	0.01335371	0.00000754	0.00103182	0.0000151	0.001938491	1	5.09531E-05	0.00725295
ILMN_2806180	CSF3R	-1.11399081	0.00176049	0.0385938	0.00149689	0.03284241	0.00012188	0.00760514	0.00034547	0.015942739	1	0.001977658	0.05767797
ILMN_2652172	NAGK	-1.114167208	0.00094262	0.02607154	0.00094262	0.02430167	0.0000386	0.00328743	0.00010625	0.007284834	1	0.000212368	0.01604723
ILMN_2859847	PYGL	-1.114288643	0.00149689	0.03478153	0.00149689	0.03284241	0.00010118	0.00668036	0.0003779	0.0167608	1	0.001482614	0.04961268
ILMN_2470360	BC037704	-1.11448614	0.0002995	0.01489206	0.0002995	0.01335371	0.00000126	0.00026389	0.00000148	0.000354054	1	9.15833E-06	0.00268109
ILMN_1217040	1300001I01RIK	-1.114584244	0.00276802	0.05133777	0.00349473	0.0566205	0.00171808	0.04381888	0.00349757	0.066721909	0	0.005373592	0.10008988
ILMN_1223285	HSPA2	-1.115046275	0.0002995	0.01489206	0.0002995	0.01335371	7.97E-07	0.00018585	0.00000345	0.000677604	1	4.70154E-05	0.00688184
ILMN_2950814	GM962	-1.115047143	0.0067415	0.08943186	0.00537037	0.07497442	0.00081444	0.02757069	0.00231909	0.051476756	0	0.004783009	0.09409752
ILMN_1214952	CLN3	-1.115097709	0.0002995	0.01489206	0.0002995	0.01335371	0.00000672	0.00095405	0.0000454	0.004033989	1	6.73408E-05	0.00831435
ILMN_3163504	METTL4	-1.115151964	0.0002995	0.01489206	0.0002995	0.01335371	0.0000174	0.00183259	0.00000733	0.001185124	1	5.09531E-05	0.00725295
ILMN_2822552	UBTD2	-1.115214016	0.00094262	0.02607154	0.00122569	0.02881591	0.00077456	0.02682534	0.00233485	0.051588335	0	0.00270263	0.06885567

ILMN_2717678	MUC13	-1.115246667	0.0006399	0.0211535	0.0002995	0.01335371	0.00000878	0.00114799	0.0000157	0.00199766	1	5.97852E-05	0.00777325
ILMN_2433547	TRP53BP1	-1.115309394	0.0002995	0.01489206	0.0002995	0.01335371	0.00000111	0.00024093	0.00000369	0.000720943	1	3.53859E-05	0.0058616
ILMN_2788073	HMOX1	-1.115323968	0.00696781	0.09115463	0.00397039	0.06196763	0.00102352	0.0318673	0.00150214	0.039708565	0	0.001977658	0.05767797
ILMN_1219208	NOL6	-1.115717769	0.0002995	0.01489206	0.0002995	0.01335371	0.00000446	0.00070872	0.0000167	0.00209125	1	0.000146035	0.01292295
ILMN_1237430	PHLPP	-1.11586422	0.00122569	0.03054155	0.00122569	0.02881591	0.00024063	0.01226772	0.00035426	0.016129651	1	0.000844832	0.03651291
ILMN_2723483	POLR1B	-1.115874714	0.00122569	0.03054155	0.0006399	0.01941092	0.0000273	0.00256319	0.0000442	0.003965776	1	0.000228655	0.01684049
ILMN_2958484	NLRX1	-1.115901939	0.0006399	0.0211535	0.0006399	0.01941092	0.00000911	0.00116871	0.0000192	0.002327044	1	0.000157497	0.01350097
ILMN_2661367	HYOU1	-1.11593221	0.0006399	0.0211535	0.0006399	0.01941092	0.00036758	0.01649923	0.00030102	0.014549744	1	0.000306263	0.02048222
ILMN_2608145	HDHD3	-1.116104843	0.0002995	0.01489206	0.0002995	0.01335371	0.000037	0.00321551	0.000031	0.003134864	1	0.000228655	0.01684049
ILMN_2791028	APP	-1.116183137	0.00276802	0.05133777	0.00276802	0.04845153	0.00012627	0.00779232	0.00047158	0.019546304	0	0.000540727	0.02850419
ILMN_2524865	CHI3L4	-1.11643408	0.0056004	0.08017457	0.00420612	0.06441203	0.00119938	0.03484851	0.00116094	0.034266489	0	0.012346572	0.15195555
ILMN_1254929	RCL1	-1.116461155	0.0002995	0.01489206	0.0002995	0.01335371	6.64E-07	0.0001602	0.00000774	0.001225133	1	9.21504E-05	0.00995203
ILMN_2939185	BC056474	-1.116523763	0.0002995	0.01489206	0.0002995	0.01335371	0.0000038	0.00063012	0.00000221	0.000472255	1	1.1878E-05	0.00314139
ILMN_1228613	PYCRL	-1.116634512	0.0002995	0.01489206	0.0002995	0.01335371	0.00014129	0.00855208	0.000044	0.003965776	1	5.74504E-05	0.00761864
ILMN_2877507	PIGQ	-1.11667927	0.00094262	0.02607154	0.0006399	0.01941092	0.0000468	0.00378362	0.00015238	0.009444363	1	0.000621233	0.03109305
ILMN_2868131	PIGT	-1.117024219	0.00122569	0.03054155	0.00122569	0.02881591	0.0000929	0.00632166	0.00031205	0.014897503	1	0.0007895	0.03543767
ILMN_1221943	SDF2L1	-1.117198955	0.00301234	0.05440113	0.0020184	0.03953445	0.00040857	0.01777361	0.00080514	0.027596907	0	0.001389407	0.04772897
ILMN_2750515	FOS	-1.11747226	0.0002995	0.01489206	0.0002995	0.01335371	0.0000144	0.00162351	0.000053	0.004480844	1	0.000643034	0.03157309
ILMN_3005873	SORT1	-1.11753372	0.0002995	0.01489206	0.0002995	0.01335371	4.28E-07	0.00011785	5.34E-07	0.000169129	1	3.83949E-05	0.00609954
ILMN_2934448	TJAP1	-1.118088782	0.0002995	0.01489206	0.0002995	0.01335371	0.0000041	0.00066517	0.00000738	0.001188627	1	2.54447E-05	0.00475651
ILMN_1240615	OLFM1	-1.118125635	0.0020184	0.04200694	0.00122569	0.02881591	0.0000124	0.00144846	0.000029	0.003012161	1	0.000284828	0.01943975
ILMN_2706231	SYNGR2	-1.118544974	0.0020184	0.04200694	0.00227139	0.0429166	0.00192157	0.04705068	0.00155324	0.040345167	1	0.00270263	0.06885567
ILMN_2776850	GAS7	-1.118567525	0.0006399	0.0211535	0.0006399	0.01941092	0.0000167	0.00178977	0.0000788	0.005900268	1	0.000140602	0.01273272
ILMN_2815889	GRWD1	-1.118687936	0.0002995	0.01489206	0.0002995	0.01335371	0.0000165	0.00178433	0.0000203	0.002399815	1	3.99889E-05	0.00618393
ILMN_1258394	AARS	-1.118724378	0.0002995	0.01489206	0.0002995	0.01335371	1.52E-08	0.00000944	3.84E-08	0.0000267	1	2.99936E-06	0.00146959
ILMN_2685565	TYMP	-1.119134268	0.0002995	0.01489206	0.0002995	0.01335371	5.95E-07	0.00014895	0.00000154	0.000360893	1	6.47271E-05	0.00803428
ILMN_1249313	RABL3	-1.119280075	0.0002995	0.01489206	0.0002995	0.01335371	0.00000837	0.00111027	0.0000181	0.002239201	1	8.19708E-05	0.00930671
ILMN_2630459	CXCR4	-1.119486645	0.01664564	0.14895372	0.010097	0.10925562	0.00150846	0.04007654	0.00513661	0.082861032	0	0.009432496	0.13248473
ILMN_2736690	2210411K11RIK	-1.119517225	0.0002995	0.01489206	0.0002995	0.01335371	0.00000361	0.00060385	3.36E-07	0.00012714	1	1.64374E-06	0.00107762
ILMN_2987863	PER2	-1.119751625	0.0020184	0.04200694	0.00122569	0.02881591	0.00049047	0.0200248	0.00050945	0.020548879	1	0.001389407	0.04772897
ILMN_2600744	RGS16	-1.119945836	0.0006399	0.0211535	0.0002995	0.01335371	0.0000862	0.00599563	0.0000228	0.002596277	1	5.09531E-05	0.00725295
ILMN_3091641	DAB2	-1.120084423	0.0056004	0.08017457	0.00349473	0.0566205	0.00031561	0.01486932	0.00066486	0.024504648	0	0.003346676	0.07734743
ILMN_2684651	MSI2H	-1.120151943	0.0006399	0.0211535	0.00094262	0.02430167	0.00015616	0.00905564	0.00017757	0.010475755	1	0.00036664	0.02284602
ILMN_2425415	UBE3B	-1.12020859	0.0002995	0.01489206	0.0002995	0.01335371	2.27E-07	0.0000712	3.67E-07	0.000134456	1	2.65234E-05	0.00480383
ILMN_2733733	TLR2	-1.120344381	0.00176049	0.0385938	0.00149689	0.03284241	0.0000662	0.00487536	0.00022244	0.012123944	1	0.000844832	0.03651291
ILMN_2855261	SLC25A39	-1.120465978	0.0002995	0.01489206	0.0002995	0.01335371	0.00020524	0.01103168	0.00039608	0.017311138	1	0.00042277	0.02466001
ILMN_2713464	ABHD4	-1.120638738	0.0002995	0.01489206	0.0002995	0.01335371	0.0000249	0.00241551	0.000033	0.003242217	1	2.76455E-05	0.00493033
ILMN_2574165	SLC7A11	-1.120754955	0.0002995	0.01489206	0.0002995	0.01335371	0.00000981	0.00121884	0.0000519	0.004442214	1	7.0054E-05	0.00846962
ILMN_1259069	F730003H07RIK	-1.121065354	0.02201501	0.1748995	0.01513108	0.13869555	0.00567109	0.08820028	0.00888129	0.111410421	0	0.019156396	0.19024382
ILMN_1252157	A330102K04RIK	-1.121227393	0.0006399	0.0211535	0.0006399	0.01941092	0.0000632	0.00470014	0.0000802	0.005962962	1	0.001179468	0.04378253
ILMN_2935032	FBXL6	-1.121239133	0.0002995	0.01489206	0.0002995	0.01335371	5.04E-07	0.00013181	0.00000874	0.00131658	1	0.000120719	0.01149104
ILMN_1253919	CCL3	-1.121443309	0.00965407	0.11001819	0.00965407	0.10652405	0.00345725	0.06587166	0.00570388	0.08745013	0	0.009432496	0.13248473
ILMN_1260585	STFA2	-1.121655647	0.00094262	0.02607154	0.0006399	0.01941092	0.00017433	0.00971369	0.00029736	0.01443844	1	0.000816732	0.0360345
ILMN_3136638	SNCA	-1.121686063	0.00786961	0.09842737	0.00373333	0.05925673	0.00040521	0.01771	0.00102328	0.031817359	0	0.033458819	0.25026637
ILMN_2936380	SGPL1	-1.121901135	0.0002995	0.01489206	0.0002995	0.01335371	0.0000477	0.00381669	0.0000543	0.004539402	1	6.47271E-05	0.00803428
ILMN_2673211	PFN1	-1.121964524	0.00467484	0.07150478	0.00176049	0.03635757	0.0006747	0.02461215	0.00083935	0.02829048	0	0.005858907	0.10440833
ILMN_1221451	DHRSX	-1.12290417	0.00176049	0.0385938	0.00176049	0.03635757	0.00051556	0.02072359	0.0008206	0.027941947	1	0.0007895	0.03543767

ILMN_2450491	TRFR2	-1.122926835	0.0002995	0.01489206	0.0002995	0.01335371	0.00000472	0.0007333	0.0000173	0.002159478	1	5.74504E-05	0.00761864
ILMN_2790512	1110039B18RIK	-1.123368404	0.0002995	0.01489206	0.0002995	0.01335371	0.00000228	0.0004265	0.0000102	0.001443157	1	9.21504E-05	0.00995203
ILMN_2894450	SNX15	-1.123418502	0.0002995	0.01489206	0.0002995	0.01335371	8.55E-08	0.0000344	6.06E-07	0.000186894	1	1.89821E-05	0.00409055
ILMN_1223966	LOC385959	-1.123693602	0.00122569	0.03054155	0.00149689	0.03284241	0.00280154	0.05916668	0.00128214	0.036191446	0	0.001686537	0.05325863
ILMN_2736347	PRMT7	-1.123726664	0.0002995	0.01489206	0.0002995	0.01335371	1.05E-09	0.00000111	1.93E-09	0.00000357	1	2.60834E-07	0.00041187
ILMN_2491099	XPOT	-1.123742439	0.0002995	0.01489206	0.0002995	0.01335371	0.00000196	0.00037186	0.00000128	0.000311058	1	3.43889E-06	0.00156931
ILMN_1232396	EAR2	-1.123772011	0.06497475	0.31131649	0.06269472	0.3035407	0.05031812	0.28834804	0.06049753	0.307739355	0	0.057275046	0.32303182
ILMN_1218602	G6PC3	-1.124251076	0.00276802	0.05133777	0.00176049	0.03635757	0.00027886	0.01369203	0.00034532	0.015942739	0	0.001531347	0.05016159
ILMN_3135781	ANXA3	-1.124627391	0.00122569	0.03054155	0.0006399	0.01941092	0.0000259	0.00247667	0.0000866	0.006296133	1	0.000873828	0.03714525
ILMN_2892376	DHX29	-1.124906344	0.0002995	0.01489206	0.0002995	0.01335371	9.49E-07	0.00021238	0.0000258	0.000529628	1	2.384E-06	0.00130551
ILMN_3161878	BID	-1.124973739	0.0002995	0.01489206	0.0002995	0.01335371	0.0000056	0.00014321	5.74E-07	0.000179299	1	2.61375E-06	0.00136712
ILMN_2847787	EMR1	-1.125484607	0.00420612	0.06669083	0.00276802	0.04845153	0.0000979	0.00654917	0.0007017	0.025381142	0	0.00388914	0.08408165
ILMN_2646878	BLMH	-1.12551314	0.0002995	0.01489206	0.0002995	0.01335371	0.00000334	0.00057008	0.0000062	0.001053987	1	1.41013E-05	0.00347315
ILMN_2706853	SCAMP1	-1.125532389	0.0002995	0.01489206	0.0002995	0.01335371	4.75E-07	0.00012706	8.24E-07	0.00022965	1	1.41024E-05	0.00347315
ILMN_2843698	SMARCAL1	-1.125762101	0.0002995	0.01489206	0.0002995	0.01335371	0.00000964	0.00121216	0.0000268	0.002862094	1	2.65234E-05	0.00480383
ILMN_2717366	OASL1	-1.125975138	0.0006399	0.0211535	0.0002995	0.01335371	0.00013051	0.00798826	0.0000663	0.005180373	1	0.000317538	0.02078825
ILMN_2546073	WDR68	-1.126231678	0.0002995	0.01489206	0.0002995	0.01335371	1.61E-07	0.0000542	2.79E-07	0.000110081	1	2.86522E-06	0.00144964
ILMN_3128907	CD63	-1.126420996	0.00176049	0.0385938	0.00149689	0.03284241	0.00054271	0.02136604	0.00067862	0.02485273	1	0.002387591	0.06387081
ILMN_2678355	AMIGO2	-1.126430355	0.0002995	0.01489206	0.0002995	0.01335371	9.13E-08	0.000036	1.85E-07	0.0000834	1	2.65193E-05	0.00480383
ILMN_2655895	AKT2	-1.126825454	0.0002995	0.01489206	0.0002995	0.01335371	2.01E-07	0.0000653	1.53E-07	0.0000735	1	1.49656E-06	0.00102442
ILMN_2625114	CDKAL1	-1.127010951	0.00122569	0.03054155	0.00094262	0.02430167	0.00000936	0.00118696	0.0000213	0.002477176	1	5.52021E-05	0.00760204
ILMN_2667384	SLC6A9	-1.127361604	0.0002995	0.01489206	0.0002995	0.01335371	0.00000932	0.00118696	0.0000174	0.002159478	1	0.000246108	0.01770572
ILMN_1222988	LOC386268	-1.127498485	0.00094262	0.02607154	0.0002995	0.01335371	0.00058613	0.02242668	0.00019971	0.011313575	1	0.001104095	0.04229821
ILMN_2895557	GFI1B	-1.127535545	0.0006399	0.0211535	0.0002995	0.01335371	0.00000674	0.00095405	0.0000135	0.001778728	1	7.88278E-05	0.00901523
ILMN_2925653	EAR2	-1.127584391	0.04792887	0.26510337	0.0460496	0.2570874	0.02741967	0.21097335	0.04301028	0.260513034	0	0.073688628	0.36208008
ILMN_1216972	CLEC4E	-1.128106489	0.00373333	0.06184411	0.0020184	0.03953445	0.00014878	0.00879971	0.0003322	0.01550964	0	0.007560831	0.11797989
ILMN_2652385	BAZ2A	-1.128274842	0.0020184	0.04200694	0.0020184	0.03953445	0.00086242	0.02851067	0.00124947	0.035680505	1	0.001482614	0.04961268
ILMN_3158659	C230096C10RIK	-1.128382209	0.00149689	0.03478153	0.00094262	0.02430167	0.00022091	0.01167139	0.00013978	0.008999194	1	0.000966442	0.03935694
ILMN_2675223	CD33	-1.128563646	0.00094262	0.02607154	0.0006399	0.01941092	0.00001	0.00123611	0.0000453	0.004030508	1	0.000844832	0.03651291
ILMN_2609073	MGAT4B	-1.128660345	0.0002995	0.01489206	0.0002995	0.01335371	0.0000179	0.00186806	0.00013637	0.008839388	1	0.000204627	0.01592867
ILMN_2588055	ACTB	-1.128864948	0.31306407	0.64308013	0.32394852	0.65411508	0.24448523	0.59865801	0.32099244	0.661760783	0	0.345393797	0.69414296
ILMN_2680686	ALG12	-1.129043435	0.0002995	0.01489206	0.0002995	0.01335371	0.0000444	0.00364378	0.0000134	0.001768921	1	1.29447E-05	0.00332893
ILMN_1219820	1200002N14RIK	-1.129152823	0.0006399	0.0211535	0.0006399	0.01941092	0.00000983	0.00121884	0.0000795	0.0059192	1	9.95836E-05	0.01046347
ILMN_2589893	TBRG4	-1.129261707	0.0006399	0.0211535	0.0006399	0.01941092	0.00017752	0.00983669	0.00024679	0.012817366	1	0.00036664	0.02284602
ILMN_2975312	FCER2A	-1.129737894	0.00252097	0.04839275	0.0020184	0.03953445	0.00029365	0.01413478	0.00040153	0.017418469	1	0.002243019	0.06192516
ILMN_2667033	ZXDC	-1.130679589	0.0002995	0.01489206	0.0002995	0.01335371	0.00000237	0.0004394	0.0000082	0.00127918	1	2.34111E-05	0.00450296
ILMN_2622671	ACSL1	-1.131479516	0.0006399	0.0211535	0.0002995	0.01335371	3.66E-07	0.00010462	8.01E-07	0.000227328	1	9.15833E-06	0.00268109
ILMN_2674324	THOP1	-1.131537424	0.0006399	0.0211535	0.0006399	0.01941092	0.0000524	0.00405887	0.00013374	0.008742949	1	0.000486846	0.02669165
ILMN_1259290	AKT3	-1.131538979	0.0002995	0.01489206	0.0002995	0.01335371	6.4E-08	0.0000271	2.38E-07	0.000101599	1	3.28593E-06	0.00154495
ILMN_2932539	RING1	-1.132133147	0.0002995	0.01489206	0.0002995	0.01335371	6.86E-07	0.00016462	7.68E-07	0.000219738	1	2.15328E-05	0.0043202
ILMN_1253819	PRKAR2B	-1.132148923	0.0002995	0.01489206	0.0002995	0.01335371	2.15E-08	0.0000125	1.03E-07	0.0000554	1	5.39498E-06	0.00202516
ILMN_2995688	EG433016	-1.132494728	0.01944482	0.1617544	0.01642977	0.14555842	0.00686131	0.09782114	0.01011034	0.119739224	0	0.023367993	0.21022612
ILMN_1238640	2310003H01RIK	-1.132552815	0.0006399	0.0211535	0.0006399	0.01941092	0.0000179	0.00186806	0.0000445	0.003979315	1	0.000264806	0.01861919
ILMN_2696696	GYPA	-1.132817135	0.00276802	0.05133777	0.00149689	0.03284241	0.0000495	0.00391795	0.00035484	0.016129651	0	0.00270263	0.06885567
ILMN_1225192	NFKBID	-1.133066098	0.0002995	0.01489206	0.0002995	0.01335371	3.36E-10	5.04E-07	1.02E-09	0.00000206	1	6.54696E-08	0.00025395
ILMN_1243066	IL1A	-1.133413071	0.02008879	0.16526703	0.01426212	0.13387405	0.00667676	0.09657646	0.00714689	0.098567729	0	0.046032778	0.29096791
ILMN_2937320	SCHIP1	-1.133751669	0.00094262	0.02607154	0.0002995	0.01335371	0.0000122	0.00143971	0.0000348	0.003370933	1	0.000176289	0.01444673

ILMN_2731237	D8ERTD82E	-1.134047583	0.0002995	0.01489206	0.0002995	0.01335371	0.0000214	0.00216179	0.0000762	0.005756583	1	0.00040803	0.02413289
ILMN_2599657	FMNL3	-1.134171185	0.0002995	0.01489206	0.0002995	0.01335371	0.00000477	0.00073484	0.00000943	0.00136995	1	1.81963E-05	0.00399552
ILMN_2731550	BRP17	-1.134397653	0.0002995	0.01489206	0.0002995	0.01335371	0.00000353	0.00059317	0.00000482	0.000858959	1	6.47271E-05	0.00803428
ILMN_2762189	GPATCH1	-1.134466014	0.0002995	0.01489206	0.0002995	0.01335371	3.36E-08	0.0000172	7.56E-07	0.000219513	1	9.99092E-06	0.00281847
ILMN_2959291	UPP1	-1.13449718	0.00094262	0.02607154	0.00094262	0.02430167	0.00041631	0.01805945	0.00028061	0.013898949	1	0.001301631	0.04617913
ILMN_2841289	TNFAIP2	-1.134827216	0.0002995	0.01489206	0.0002995	0.01335371	0.00000721	0.00099242	0.00000322	0.00064069	1	3.26018E-05	0.00557911
ILMN_2671411	KLC1	-1.135147123	0.0002995	0.01489206	0.0002995	0.01335371	5.07E-08	0.0000227	3.61E-07	0.00013352	1	5.15939E-06	0.0020181
ILMN_2596522	MT1	-1.135343672	0.0002995	0.01489206	0.0002995	0.01335371	0.00000371	0.00061709	4.42E-07	0.000148085	1	1.41024E-05	0.00347315
ILMN_2939681	LYZS	-1.136569055	0.0056004	0.08017457	0.00444101	0.06659434	0.00127184	0.03607589	0.0015594	0.040474884	0	0.004644853	0.0931111
ILMN_2964076	TSPAN31	-1.137017412	0.00122569	0.03054155	0.00094262	0.02430167	0.00012131	0.00760514	0.00019258	0.011039321	1	0.000504223	0.02732257
ILMN_2692797	SLC11A1	-1.137029044	0.00094262	0.02607154	0.0002995	0.01335371	0.0000212	0.00215125	0.0000583	0.004702444	1	0.001259689	0.04555924
ILMN_2693124	RAB1B	-1.137428102	0.01965954	0.16292455	0.01142074	0.11750706	0.00353484	0.06674889	0.00513165	0.08285783	0	0.015623977	0.17119804
ILMN_1220769	LMAN2L	-1.138159096	0.0002995	0.01489206	0.0002995	0.01335371	1.01E-08	0.00000691	8.75E-08	0.0000485	1	5.74828E-07	0.00056929
ILMN_2701271	PLSCR1	-1.138607055	0.0002995	0.01489206	0.0002995	0.01335371	1.15E-07	0.0000431	5.41E-08	0.0000344	1	5.21457E-07	0.00053938
ILMN_2778289	CXCL2	-1.140544603	0.0264868	0.19288541	0.01404445	0.132649	0.0022071	0.05134118	0.00600321	0.089676275	0	0.041147128	0.27693959
ILMN_1249362	LOC386298	-1.141212883	0.00122569	0.03054155	0.00122569	0.02881591	0.00022562	0.01181303	0.00059102	0.022623651	1	0.001141234	0.04287409
ILMN_2661287	AKP2	-1.141256855	0.0002995	0.01489206	0.0002995	0.01335371	4.01E-07	0.00011255	7.15E-07	0.000216027	1	3.59833E-06	0.00158025
ILMN_2846865	ACTB	-1.141333782	0.27330516	0.60657516	0.28441349	0.61784421	0.20397044	0.5535661	0.27555162	0.619558566	0	0.257914169	0.61888498
ILMN_2925711	DUSP6	-1.142932506	0.0006399	0.0211535	0.0002995	0.01335371	9.85E-07	0.00021734	0.0000039	0.000746457	1	4.70154E-05	0.00688184
ILMN_2803674	S100A9	-1.1442518	0.0020184	0.04200694	0.00094262	0.02430167	0.00032351	0.01511104	0.00019143	0.01098713	1	0.001633268	0.05224999
ILMN_2866276	CSF2RA	-1.144341375	0.0002995	0.01489206	0.0002995	0.01335371	0.0000165	0.00178433	0.0000775	0.005835203	1	0.00040803	0.02413289
ILMN_2524986	EAR3	-1.144901605	0.01707784	0.15127561	0.01858605	0.1562833	0.01561583	0.15736519	0.02212242	0.183826235	0	0.019643695	0.19233384
ILMN_2479717	PILRA	-1.145002307	0.0002995	0.01489206	0.0002995	0.01335371	0.00000201	0.000379	0.00000853	0.001297207	1	2.65234E-05	0.00480383
ILMN_2987709	SLC15A3	-1.145009054	0.0002995	0.01489206	0.0002995	0.01335371	0.0000166	0.00178433	0.0000144	0.001881854	1	0.000212368	0.01604723
ILMN_1224736	ZMIZ1	-1.145106147	0.0002995	0.01489206	0.0002995	0.01335371	0.0000174	0.00183609	0.0000098	0.001399019	1	2.34111E-05	0.00450296
ILMN_3155815	9830134C10RIK	-1.145114089	0.0002995	0.01489206	0.0002995	0.01335371	2.35E-07	0.0000718	0.00000169	0.000383182	1	6.16514E-06	0.0021257
ILMN_2654754	GP38	-1.146427658	0.00854165	0.10305721	0.0056004	0.0767776	0.00506605	0.08253748	0.00357137	0.067411346	0	0.016875145	0.17884503
ILMN_3104139	RBM12	-1.147016272	0.0002995	0.01489206	0.0002995	0.01335371	1.43E-08	0.00000901	1.38E-08	0.0000126	1	1.15863E-07	0.00029962
ILMN_2712986	CHI3L3	-1.147371605	0.00122569	0.03054155	0.00149689	0.03284241	0.00087512	0.02868922	0.00073982	0.026207168	1	0.004127635	0.08709385
ILMN_2757368	CRELD2	-1.147374056	0.0002995	0.01489206	0.0002995	0.01335371	2.3E-09	0.00000185	1.18E-08	0.0000119	1	2.74304E-07	0.00041187
ILMN_2491202	VNN3	-1.148153174	0.0002995	0.01489206	0.0002995	0.01335371	4.51E-09	0.00000339	8.56E-09	0.0000105	1	2.24455E-07	0.00037313
ILMN_1230766	SCMH1	-1.148605754	0.0002995	0.01489206	0.0002995	0.01335371	4.83E-08	0.0000223	1.86E-07	0.0000834	1	4.93323E-06	0.00196262
ILMN_2771380	CD52	-1.149676808	0.00898742	0.10547593	0.0076447	0.09407685	0.00210007	0.0498312	0.00636866	0.092696085	0	0.009693098	0.13460577
ILMN_1230708	CLEC4A1	-1.149760601	0.0002995	0.01489206	0.0002995	0.01335371	1.72E-07	0.0000571	0.00000112	0.000290018	1	1.53585E-05	0.00359242
ILMN_2684575	PCYOX1L	-1.149890332	0.00122569	0.03054155	0.0006399	0.01941092	0.0000565	0.00431632	0.00014233	0.009037925	1	0.000438006	0.02507734
ILMN_1218347	MYLK	-1.151233811	0.0002995	0.01489206	0.0002995	0.01335371	1.44E-09	0.00000136	5.7E-09	0.00000758	1	3.34766E-07	0.00044521
ILMN_2601946	5033414K04RIK	-1.151524006	0.0002995	0.01489206	0.0002995	0.01335371	0.0000259	0.00046493	0.0000166	0.000379819	1	0.000317538	0.02078825
ILMN_2835117	CCL7	-1.1516614	0.0002995	0.01489206	0.0002995	0.01335371	7.99E-07	0.00018585	0.00000889	0.001330104	1	9.5799E-05	0.01018072
ILMN_2759484	C3	-1.152531108	0.0006399	0.0211535	0.0002995	0.01335371	0.0000685	0.00500185	0.0000487	0.004231895	1	0.000438006	0.02507734
ILMN_1223416	FER1L3	-1.152772279	0.0002995	0.01489206	0.0002995	0.01335371	4.78E-08	0.0000223	3.94E-07	0.00013797	1	3.99889E-05	0.00618393
ILMN_2901626	TNFRSF21	-1.153121675	0.00094262	0.02607154	0.0006399	0.01941092	0.00000168	0.00033087	0.0000142	0.001859837	1	0.00010758	0.01083883
ILMN_2693895	ACTA2	-1.153348714	0.0002995	0.01489206	0.0002995	0.01335371	0.00000119	0.00025415	0.00000516	0.00090936	1	0.000151664	0.01324485
ILMN_2777498	IL1B	-1.153646647	0.0056004	0.08017457	0.00420612	0.06441203	0.00135477	0.03741307	0.0017555	0.043416704	0	0.004379328	0.09051712
ILMN_2789900	CD177	-1.154670942	0.0006399	0.0211535	0.0002995	0.01335371	0.00000234	0.00043645	0.00000186	0.000412865	1	1.89821E-05	0.00409055
ILMN_2618423	ZXDC	-1.15475865	0.0002995	0.01489206	0.0002995	0.01335371	9.02E-10	9.76E-07	2E-09	0.00000357	1	1.04496E-07	0.00029962
ILMN_1242457	FPR2	-1.155423301	0.00301234	0.05440113	0.0020184	0.03953445	0.00012201	0.00760514	0.00017744	0.010475755	0	0.00093456	0.03863507
ILMN_2742075	CD14	-1.15645531	0.0032539	0.05678214	0.00252097	0.04602629	0.00038681	0.01712826	0.00049905	0.020287507	0	0.001389407	0.04772897

ILMN_2868220	INHBA	-1.156681552	0.00122569	0.03054155	0.00094262	0.02430167	0.00016524	0.00937956	0.00024079	0.012650343	1	0.000470028	0.02607676
ILMN_2674884	SLC11A1	-1.157414839	0.00094262	0.02607154	0.00094262	0.02430167	0.0000257	0.00247314	0.0000641	0.00508011	1	0.000438006	0.02507734
ILMN_3161601	SNCA	-1.157946415	0.0020184	0.04200694	0.00149689	0.03284241	0.00000758	0.00103523	0.0000792	0.005917903	1	0.007351981	0.11667667
ILMN_2705860	TPSAB1	-1.160383394	0.00094262	0.02607154	0.0006399	0.01941092	0.000013	0.00149316	0.00006	0.004813511	1	0.000934606	0.03863507
ILMN_2710905	S100A8	-1.163713512	0.0006399	0.0211535	0.0002995	0.01335371	0.0000119	0.00142619	0.0000147	0.001907372	1	0.000182986	0.01471244
ILMN_2595732	LOC100046232	-1.165021501	0.0002995	0.01489206	0.0002995	0.01335371	0.000001	0.00021909	2.17E-07	0.0000944	1	5.15939E-06	0.0020181
ILMN_2719732	MS4A3	-1.166305486	0.0006399	0.0211535	0.0002995	0.01335371	0.00000901	0.00116445	0.0000212	0.002477176	1	0.000540727	0.02850419
ILMN_1221700	ELA2	-1.167300103	0.0002995	0.01489206	0.0002995	0.01335371	3.27E-09	0.00000253	1.67E-09	0.00000324	1	2.02641E-08	0.00018865
ILMN_1224472	CCL4	-1.167531549	0.00094262	0.02607154	0.0006399	0.01941092	0.00000658	0.0009452	0.0000352	0.003396141	1	0.000255296	0.01817016
ILMN_2728038	ARHGAP24	-1.167825732	0.0002995	0.01489206	0.0002995	0.01335371	4.47E-11	8.33E-08	1.1E-10	0.000000395	1	6.03547E-07	0.00057333
ILMN_1252819	PRMT7	-1.167997376	0.0002995	0.01489206	0.0002995	0.01335371	8.95E-10	9.76E-07	7.15E-10	0.00000159	1	4.07147E-08	0.00024945
ILMN_2538531	LOC386360	-1.168408104	0.0002995	0.01489206	0.0002995	0.01335371	0.00012104	0.00760514	0.00051103	0.020559076	1	0.001104095	0.04229821
ILMN_2493826	UGT1A10	-1.169469481	0.0002995	0.01489206	0.0002995	0.01335371	1.33E-08	0.0000087	8.14E-08	0.0000468	1	3.13951E-06	0.00152224
ILMN_2834379	TGFBI	-1.172758284	0.00176049	0.0385938	0.00122569	0.02881591	0.00093773	0.03009876	0.00024903	0.012894089	1	0.001068077	0.04167292
ILMN_1222543	UGT1A10	-1.173528971	0.0002995	0.01489206	0.0002995	0.01335371	1.46E-09	0.00000136	4.37E-08	0.0000295	1	3.59866E-06	0.00158025
ILMN_2983516	SLC11A1	-1.17650384	0.00122569	0.03054155	0.0006399	0.01941092	0.0000123	0.00144253	0.0000539	0.004524583	1	0.000643034	0.03157309
ILMN_2426480	3830612M24	-1.17764975	0.0006399	0.0211535	0.0002995	0.01335371	7.32E-08	0.0000304	0.00000104	0.000275608	1	9.21504E-05	0.00995203
ILMN_2592554	REXO1	-1.182418789	0.0002995	0.01489206	0.0002995	0.01335371	2.19E-09	0.00000179	2.9E-08	0.0000218	1	7.3257E-07	0.00068198
ILMN_1220548	LRRC41	-1.183163213	0.0002995	0.01489206	0.0002995	0.01335371	2.09E-08	0.0000125	3.74E-07	0.000136	1	9.99092E-06	0.00281847
ILMN_1233336	LOC386199	-1.183990269	0.0006399	0.0211535	0.0006399	0.01941092	0.0000163	0.00177372	0.0000255	0.00279613	1	0.00040803	0.02413289
ILMN_2646985	ATF5	-1.18420231	0.0002995	0.01489206	0.0002995	0.01335371	1.22E-07	0.0000454	0.00000123	0.000301817	1	2.15311E-05	0.0043202
ILMN_2925094	MPO	-1.185131735	0.0002995	0.01489206	0.0002995	0.01335371	0.00000078	0.0001843	0.00000115	0.000292494	1	1.89821E-05	0.00409055
ILMN_1252076	LYZ2	-1.187850205	0.00349473	0.05908105	0.00149689	0.03284241	0.0000728	0.00528008	0.00032836	0.015438431	0	0.002314272	0.06295874
ILMN_2948143	SLC7A11	-1.188505133	0.0002995	0.01489206	0.0002995	0.01335371	5.44E-07	0.00014066	0.00000159	0.000369569	1	0.000120719	0.01149104
ILMN_1219230	LOC100043402	-1.188859089	0.0002995	0.01489206	0.0002995	0.01335371	0.00000132	0.00027167	0.00000103	0.000274668	1	2.15328E-05	0.0043202
ILMN_1230140	LOC385923	-1.189899209	0.0002995	0.01489206	0.0002995	0.01335371	0.00000304	0.00052754	0.0000057	0.000979318	1	3.53859E-05	0.0058616
ILMN_3162125	GRM6	-1.194679101	0.0002995	0.01489206	0.0002995	0.01335371	2.08E-07	0.0000667	1.2E-08	0.0000119	1	4.9657E-07	0.00052531
ILMN_2771176	CCL7	-1.196175382	0.0002995	0.01489206	0.0002995	0.01335371	1.02E-11	2.5E-08	4.19E-10	0.000000976	1	4.07997E-07	0.00049976
ILMN_2847115	GPR109A	-1.199553298	0.0006399	0.0211535	0.0006399	0.01941092	0.00000482	0.0007403	0.0000154	0.001964139	1	0.00011618	0.0112429
ILMN_2758029	PRTN3	-1.205025411	0.0002995	0.01489206	0.0002995	0.01335371	1.53E-10	2.64E-07	3.43E-10	0.00000084	1	5.59305E-08	0.00024945
ILMN_2722996	SIRPA	-1.209776171	0.0002995	0.01489206	0.0002995	0.01335371	1.66E-09	0.00000149	3.18E-09	0.00000462	1	3.28593E-06	0.00154495
ILMN_2657828	RHBDF1	-1.216435604	0.0002995	0.01489206	0.0002995	0.01335371	2.44E-12	9.46E-09	1.36E-10	0.000000429	1	5.89499E-08	0.00024945
ILMN_2712075	LCN2	-1.216711224	0.0006399	0.0211535	0.0002995	0.01335371	0.00000262	0.00046695	0.00000288	0.000577427	1	2.44059E-05	0.00463717
ILMN_2878071	LYZ	-1.22864681	0.0002995	0.01489206	0.0002995	0.01335371	5.41E-08	0.000024	0.00000139	0.000336099	1	7.57938E-05	0.00879794
ILMN_2705628	CLEC4D	-1.228745779	0.0006399	0.0211535	0.0006399	0.01941092	0.00000062	0.00015362	0.0000039	0.000746457	1	7.0054E-05	0.00846962
ILMN_1233982	LOC270589	-1.22932799	0.0002995	0.01489206	0.0002995	0.01335371	0.00000694	0.00097871	0.00000394	0.000746457	1	4.51564E-05	0.00675851
ILMN_1377923	ACTB	-1.230879389	0.07635019	0.3372365	0.05022289	0.26966529	0.00918324	0.11590351	0.03014873	0.215797746	0	0.014835081	0.16656218
ILMN_1249030	MPO	-1.231747939	0.0002995	0.01489206	0.0002995	0.01335371	0.0000017	0.00033473	0.0000195	0.002344998	1	0.00022037	0.01646479
ILMN_2538242	LOC386144	-1.239189285	0.0002995	0.01489206	0.0002995	0.01335371	0.000015	0.00166908	0.0000107	0.001483462	1	0.000135361	0.01242731
ILMN_2993314	CLEC4N	-1.244916169	0.0006399	0.0211535	0.0002995	0.01335371	0.00000115	0.00024883	0.000015	0.001929689	1	0.000163541	0.01374071
ILMN_2750842	2610027L16RIK	-1.245539485	0.0002995	0.01489206	0.0002995	0.01335371	0.00000254	0.00046434	0.00000545	0.000950899	1	6.44447E-06	0.00218957
ILMN_2600421	MPO	-1.29293209	0.0002995	0.01489206	0.0002995	0.01335371	8.09E-10	9.34E-07	2.37E-08	0.0000187	1	8.76731E-06	0.00261597
ILMN_2763245	CXCL1	-1.3	0.0006399	0.0211535	0.0002995	0.01335371	2.37E-07	0.0000718	0.00000181	0.000406	1	9.95836E-05	0.01046347
ILMN_1253874	SERPINB2	-1.31	0.0002995	0.01489206	0.0002995	0.01335371	6.55E-10	8.24E-07	9.01E-09	0.0000105	1	2.38377E-06	0.00130551
ILMN_1215076	F830002E14RIK	-1.32	0.0020184	0.04200694	0.00122569	0.02881591	0.00115136	0.03420847	0.000886	0.0292	1	0.001344859	0.0469611
ILMN_2725259	IL2	-1.37	0.0002995	0.01489206	0.0002995	0.01335371	5.55E-14	6.46E-10	1.82E-11	0.000000121	1	9.40233E-09	0.00012222
