# Supplementary material for: Comparing the Biological Impact of Glatiramer Acetate with the Biological Impact of a Generic
Source: PLoS One. 2014 Jan 8;9(1):e83757. doi: 10.1371/journal.pone.0083757 (PMC3885444; doi:10.1371/journal.pone.0083757)
Supplement: Table S6 — MSigDB enrichment results for the list of genes with significantly different expression between GA and generic by the Wilcoxon rank sum test, including FoxP3 targets among the enriched signatures for genes higher in GA than generic, and TLR and LPS pathways among the enriched signatures for genes higher in generic than GA. (PDF) [file pone.0083757.s014.pdf]

TABLE S6							
Reference Set	User Set	N	K	n	k	P	BHP_P
BLALOCK_ALZHEIMERS_DISEASE_UP	wilcox_up_in_GA	31847	1691	392	54	0	0
PILON_KLF1_TARGETS_DN	wilcox_up_in_GA	31847	1972	392	80	0	0
MEMBRANE	wilcox_up_in_GA	31847	1994	392	65	0	0
GRAESSMANN_APOPTOSIS_BY_DOXORUBICIN_DN	wilcox_up_in_GA	31847	1781	392	60	0	0
GGGAGGRR_V$MAZ_Q6	wilcox_up_in_GA	31847	2274	392	69	0	0
SIGNAL_TRANSDUCTION	wilcox_up_in_GA	31847	1634	392	60	0	0
TTGTTT_V$FOXO4_01	wilcox_up_in_GA	31847	2061	392	64	0	0
DUTERTRE_ESTRADIOL_RESPONSE_24HR_DN	wilcox_up_in_GA	31847	505	392	30	0	0
GNF2_MATK	wilcox_up_in_GA	31847	25	392	10	0	0
PID_TCR_PATHWAY	wilcox_up_in_GA	31847	66	392	14	3.55E-12	8.55E-10
MEMBRANE_PART	wilcox_up_in_GA	31847	1670	392	53	8.99E-12	1.97E-09
BIOPOLYMER_METABOLIC_PROCESS	wilcox_up_in_GA	31847	1684	392	53	1.10E-11	2.20E-09
PID_CD8TCRDOWNSTREAMPATHWAY	wilcox_up_in_GA	31847	65	392	12	4.02E-11	7.45E-09
REACTOME_TCR_SIGNALING	wilcox_up_in_GA	31847	54	392	13	4.93E-11	8.47E-09
PID_CD8TCRPATHWAY	wilcox_up_in_GA	31847	53	392	12	7.51E-11	1.21E-08
SMID_BREAST_CANCER_NORMAL_LIKE_UP	wilcox_up_in_GA	31847	476	392	48	9.86E-11	1.44E-08
PUJANA_ATM_PCC_NETWORK	wilcox_up_in_GA	31847	1442	392	52	1.02E-10	1.44E-08
GNF2_PTPN4	wilcox_up_in_GA	31847	51	392	11	1.17E-10	1.57E-08
MODULE_64	wilcox_up_in_GA	31847	518	392	28	1.30E-10	1.59E-08
PID_IL12_STAT4PATHWAY	wilcox_up_in_GA	31847	33	392	9	1.37E-10	1.59E-08
ZHENG_BOUND_BY_FOXP3	wilcox_up_in_GA	31847	491	392	56	1.42E-10	1.59E-08
DIAZ_CHRONIC_MEYLOGENOUS_LEUKEMIA_UP	wilcox_up_in_GA	31847	1382	392	51	1.46E-10	1.59E-08
CHEN_METABOLIC_SYNDROM_NETWORK	wilcox_up_in_GA	31847	1210	392	46	1.72E-10	1.79E-08
MODULE_75	wilcox_up_in_GA	31847	399	392	29	1.86E-10	1.80E-08
MODULE_84	wilcox_up_in_GA	31847	549	392	38	1.87E-10	1.80E-08
MARSON_BOUND_BY_FOXP3_UNSTIMULATED	wilcox_up_in_GA	31847	1229	392	66	2.04E-10	1.80E-08
LINDGREN_BLADDER_CANCER_CLUSTER_2B	wilcox_up_in_GA	31847	392	392	29	2.12E-10	1.80E-08
SANSOM_APC_TARGETS_DN	wilcox_up_in_GA	31847	366	392	25	2.16E-10	1.80E-08
MODULE_46	wilcox_up_in_GA	31847	395	392	29	2.27E-10	1.80E-08
SMID_BREAST_CANCER_LUMINAL_B_DN	wilcox_up_in_GA	31847	564	392	30	2.32E-10	1.80E-08
GCACTTT,MIR-17-5P,MIR-20A,MIR-106A,MIR-106B,MI	wilcox_up_in_GA	31847	595	392	33	2.46E-10	1.80E-08
NUYTTEN_EZH2_TARGETS_UP	wilcox_up_in_GA	31847	1037	392	44	2.46E-10	1.80E-08
KUMAR_TARGETS_OF_MLL_AF9_FUSION	wilcox_up_in_GA	31847	405	392	26	2.47E-10	1.80E-08
MARSON_BOUND_BY_FOXP3_STIMULATED	wilcox_up_in_GA	31847	1022	392	54	2.65E-10	1.83E-08
GOBERT_OLIGODENDROCYTE_DIFFERENTIATION_DN	wilcox_up_in_GA	31847	1080	392	44	2.67E-10	1.83E-08
REACTOME_IMMUNE_SYSTEM	wilcox_up_in_GA	31847	933	392	51	2.82E-10	1.88E-08

MODULE_45	wilcox_up_in_GA	31847	583	392	33	2.99E-10	1.95E-08
~~							
Reference Set	User Set	N	K	n	k	P	BHP_P
CYTOPLASM	wilcox_up_in_generic	31847	2131	439	68	0	0
BLALOCK_ALZHEIMERS_DISEASE_UP	wilcox_up_in_generic	31847	1691	439	59	0	0
PUJANA_BRCA1_PCC_NETWORK	wilcox_up_in_generic	31847	1652	439	57	0	0
PILON_KLF1_TARGETS_DN	wilcox_up_in_generic	31847	1972	439	81	0	0
GRAESSMANN_APOPTOSIS_BY_DOXORUBICIN_DN	wilcox_up_in_generic	31847	1781	439	68	0	0
DIAZ_CHRONIC_MEYLOGENOUS_LEUKEMIA_UP	wilcox_up_in_generic	31847	1382	439	56	6.56E-11	2.51E-08
KRIGE_RESPONSE_TO_TOSEDOSTAT_6HR_UP	wilcox_up_in_generic	31847	953	439	46	1.20E-10	3.57E-08
KRIGE_RESPONSE_TO_TOSEDOSTAT_24HR_DN	wilcox_up_in_generic	31847	1011	439	51	1.24E-10	3.57E-08
GRAESSMANN_APOPTOSIS_BY_DOXORUBICIN_UP	wilcox_up_in_generic	31847	1142	439	46	2.24E-10	5.64E-08
REACTOME_IMMUNE_SYSTEM	wilcox_up_in_generic	31847	933	439	41	2.48E-10	5.64E-08
KRIGE_RESPONSE_TO_TOSEDOSTAT_6HR_DN	wilcox_up_in_generic	31847	911	439	45	2.74E-10	5.64E-08
CHEN_METABOLIC_SYNDROM_NETWORK	wilcox_up_in_generic	31847	1210	439	48	2.95E-10	5.64E-08
PROTEIN_METABOLIC_PROCESS	wilcox_up_in_generic	31847	1231	439	48	3.86E-10	6.58E-08
LEE_BMP2_TARGETS_DN	wilcox_up_in_generic	31847	882	439	52	4.01E-10	6.58E-08
WEI_MYCN_TARGETS_WITH_E_BOX	wilcox_up_in_generic	31847	795	439	39	5.50E-10	8.42E-08
MODULE_5	wilcox_up_in_generic	31847	434	439	26	6.51E-10	9.34E-08
GGGCGGR_V$SP1_Q6	wilcox_up_in_generic	31847	2940	439	95	7.24E-10	9.78E-08
BYSTRYKH_HEMATOPOIESIS_STEM_CELL_QTL_TRANS	wilcox_up_in_generic	31847	882	439	38	1.16E-09	1.48E-07
ENK_UV_RESPONSE_EPIDERMIS_UP	wilcox_up_in_generic	31847	293	439	21	1.31E-09	1.59E-07
MONNIER_POSTRADIATION_TUMOR_ESCAPE_UP	wilcox_up_in_generic	31847	393	439	24	1.69E-09	1.94E-07
BROWN_MYELOID_CELL_DEVELOPMENT_UP	wilcox_up_in_generic	31847	165	439	16	1.91E-09	2.09E-07
SENESE_HDAC3_TARGETS_UP	wilcox_up_in_generic	31847	501	439	27	2.47E-09	2.58E-07
BIOPOLYMER_METABOLIC_PROCESS	wilcox_up_in_generic	31847	1684	439	55	2.71E-09	2.71E-07
GNF2_CARD15	wilcox_up_in_generic	31847	70	439	11	3.41E-09	3.26E-07
DODD_NASOPHARYNGEAL_CARCINOMA_DN	wilcox_up_in_generic	31847	1375	439	48	4.41E-09	4.05E-07
SCGGAAGY_V$ELK1_02	wilcox_up_in_generic	31847	1199	439	44	4.79E-09	4.23E-07
VERHAAK_AML_WITH_NPM1_MUTATED_UP	wilcox_up_in_generic	31847	183	439	16	6.55E-09	5.57E-07
CAIRO_HEPATOBLASTOMA_CLASSES_UP	wilcox_up_in_generic	31847	605	439	29	8.42E-09	6.91E-07
GNF2_CD1D	wilcox_up_in_generic	31847	45	439	9	9.51E-09	7.53E-07
RUTELLA_RESPONSE_TO_CSF2RB_AND_IL4_UP	wilcox_up_in_generic	31847	338	439	21	1.29E-08	9.69E-07
LI_INDUCED_T_TO_NATURAL_KILLER_UP	wilcox_up_in_generic	31847	307	439	20	1.31E-08	9.69E-07
BOYLAN_MULTIPLE_MYELOMA_C_D_DN	wilcox_up_in_generic	31847	252	439	18	1.73E-08	1.24E-06
KEGG_TOLL_LIKE_RECEPTOR_SIGNALING_PATHWAY	wilcox_up_in_generic	31847	102	439	12	1.83E-08	1.27E-06

MODULE_6	wilcox_up_in_generic	31847	416	439	23	2.30E-08	1.55E-06
RUTELLA_RESPONSE_TO_HGF_UP	wilcox_up_in_generic	31847	418	439	23	2.51E-08	1.65E-06
MODULE_3	wilcox_up_in_generic	31847	385	439	22	2.59E-08	1.65E-06
CHARAFE_BREAST_CANCER_LUMINAL_VS_BASAL_DN	wilcox_up_in_generic	31847	455	439	24	2.78E-08	1.73E-06
MARTENS_BOUND_BY_PML_RARA_FUSION	wilcox_up_in_generic	31847	456	439	24	2.90E-08	1.75E-06
GNF2_CD33	wilcox_up_in_generic	31847	52	439	9	3.62E-08	2.13E-06
NUCLEOBASENUCLEOSIDENUCLEOTIDE_AND_NUCLEIC	wilcox_up_in_generic	31847	1244	439	43	3.88E-08	2.23E-06
TONKS_TARGETS_OF_RUNX1_RUNX1T1_FUSION_ERYT	wilcox_up_in_generic	31847	157	439	14	4.29E-08	2.41E-06
FOSTER_KDM1A_TARGETS_DN	wilcox_up_in_generic	31847	211	439	16	4.64E-08	2.54E-06
NUYTTEN_EZH2_TARGETS_UP	wilcox_up_in_generic	31847	1037	439	38	5.75E-08	3.05E-06
MARKEY_RB1_ACUTE_LOF_UP	wilcox_up_in_generic	31847	215	439	16	6.02E-08	3.05E-06
CASORELLI_ACUTE_PROMYELOCYTIC_LEUKEMIA_DN	wilcox_up_in_generic	31847	663	439	29	6.04E-08	3.05E-06
MODULE_16	wilcox_up_in_generic	31847	511	439	25	6.11E-08	3.05E-06
BRUINS_UVC_RESPONSE_LATE	wilcox_up_in_generic	31847	1137	439	40	7.46E-08	3.65E-06
ZHANG_TLX_TARGETS_36HR_UP	wilcox_up_in_generic	31847	221	439	16	8.79E-08	4.21E-06
RODWELL_AGING_KIDNEY_UP	wilcox_up_in_generic	31847	487	439	24	9.84E-08	4.61E-06
SEKI_INFLAMMATORY_RESPONSE_LPS_UP	wilcox_up_in_generic	31847	77	439	10	1.08E-07	4.96E-06
HESS_TARGETS_OF_HOXA9_AND_MEIS1_DN	wilcox_up_in_generic	31847	77	439	10	1.08E-07	4.96E-06
IVANOVA_HEMATOPOIESIS_EARLY_PROGENITOR	wilcox_up_in_generic	31847	532	439	25	1.31E-07	5.77E-06
CELLULAR_PROTEIN_METABOLIC_PROCESS	wilcox_up_in_generic	31847	1117	439	39	1.33E-07	5.77E-06
ACEVEDO_LIVER_TUMOR_VS_NORMAL_ADJACENT_TI	wilcox_up_in_generic	31847	863	439	33	1.69E-07	7.21E-06
WANG_IMMORTALIZED_BY_HOXA9_AND_MEIS1_UP	wilcox_up_in_generic	31847	31	439	7	1.78E-07	7.36E-06
RYTTCCTG_V$ETS2_B	wilcox_up_in_generic	31847	1085	439	38	1.80E-07	7.36E-06
CELLULAR_MACROMOLECULE_METABOLIC_PROCESS	wilcox_up_in_generic	31847	1131	439	39	1.83E-07	7.36E-06
KOINUMA_TARGETS_OF_SMAD2_OR_SMAD3	wilcox_up_in_generic	31847	824	439	32	1.86E-07	7.37E-06
KRIGE_RESPONSE_TO_TOSEDOSTAT_24HR_UP	wilcox_up_in_generic	31847	783	439	31	1.91E-07	7.45E-06
MODULE_45	wilcox_up_in_generic	31847	583	439	26	2.03E-07	7.76E-06
RASHI_RESPONSE_TO_IONIZING_RADIATION_2	wilcox_up_in_generic	31847	127	439	12	2.09E-07	7.88E-06
RESPONSE_TO_STRESS	wilcox_up_in_generic	31847	508	439	24	2.13E-07	7.88E-06
LENAOUR_DENDRITIC_CELL_MATURATION_DN	wilcox_up_in_generic	31847	128	439	12	2.28E-07	8.31E-06
MODULE_84	wilcox_up_in_generic	31847	549	439	25	2.37E-07	8.49E-06
GROSS_HYPOXIA_VIA_ELK3_UP	wilcox_up_in_generic	31847	209	439	15	2.48E-07	8.76E-06
GTGCCAA,MIR-96	wilcox_up_in_generic	31847	303	439	18	2.73E-07	9.49E-06
MARTENS_TRETINOIN_RESPONSE_DN	wilcox_up_in_generic	31847	841	439	32	2.92E-07	1.00E-05
MGGAAGTG_V$GABP_B	wilcox_up_in_generic	31847	757	439	30	2.96E-07	1.00E-05
ACEVEDO_LIVER_CANCER_UP	wilcox_up_in_generic	31847	973	439	35	3.01E-07	1.00E-05
NEGATIVE_REGULATION_OF_BIOLOGICAL_PROCESS	wilcox_up_in_generic	31847	677	439	28	3.18E-07	1.04E-05
MODULE_118	wilcox_up_in_generic	31847	410	439	21	3.33E-07	1.08E-05

GAL_LEUKEMIC_STEM_CELL_DN	wilcox_up_in_generic	31847	244	439	16	3.38E-07	1.08E-05
MOOTHA_MITOCHONDRIA	wilcox_up_in_generic	31847	447	439	22	3.44E-07	1.08E-05
LINDSTEDT_DENDRITIC_CELL_MATURATION_A	wilcox_up_in_generic	31847	67	439	9	3.49E-07	1.08E-05
MEMBRANE	wilcox_up_in_generic	31847	1994	439	56	3.55E-07	1.09E-05
TARTE_PLASMA_CELL_VS_PLASMABLAST_DN	wilcox_up_in_generic	31847	309	439	18	3.64E-07	1.10E-05
NEGATIVE_REGULATION_OF_CELLULAR_PROCESS	wilcox_up_in_generic	31847	646	439	27	4.22E-07	1.25E-05
GRAESSMANN_RESPONSE_TO_MC_AND_DOXORUBICI	wilcox_up_in_generic	31847	770	439	30	4.23E-07	1.25E-05
GNF2_CD14	wilcox_up_in_generic	31847	35	439	7	4.35E-07	1.26E-05
RPS14_DN.V1_UP	wilcox_up_in_generic	31847	192	439	14	5.14E-07	1.47E-05
PUJANA_CHEK2_PCC_NETWORK	wilcox_up_in_generic	31847	779	439	30	5.39E-07	1.53E-05
BERENJENO_TRANSFORMED_BY_RHOA_UP	wilcox_up_in_generic	31847	536	439	24	5.57E-07	1.56E-05
CELL_DEVELOPMENT	wilcox_up_in_generic	31847	577	439	25	5.92E-07	1.64E-05
ONDER_CDH1_TARGETS_2_DN	wilcox_up_in_generic	31847	464	439	22	6.44E-07	1.76E-05
REACTOME_ACTIVATED_TLR4_SIGNALLING	wilcox_up_in_generic	31847	93	439	10	6.52E-07	1.76E-05
MTOR_UP.N4.V1_UP	wilcox_up_in_generic	31847	196	439	14	6.59E-07	1.76E-05
CYTOPLASMIC_PART	wilcox_up_in_generic	31847	1383	439	43	6.89E-07	1.82E-05
NEGATIVE_REGULATION_OF_DEVELOPMENTAL_PROC	wilcox_up_in_generic	31847	197	439	14	7.00E-07	1.83E-05
GNF2_HCK	wilcox_up_in_generic	31847	94	439	10	7.21E-07	1.86E-05
COFACTOR_METABOLIC_PROCESS	wilcox_up_in_generic	31847	54	439	8	7.31E-07	1.87E-05
SIGNAL_TRANSDUCTION	wilcox_up_in_generic	31847	1634	439	48	7.57E-07	1.91E-05
PROGRAMMED_CELL_DEATH	wilcox_up_in_generic	31847	432	439	21	7.76E-07	1.94E-05
