# Supplementary material for: Comparing the Biological Impact of Glatiramer Acetate with the Biological Impact of a Generic
Source: PLoS One. 2014 Jan 8;9(1):e83757. doi: 10.1371/journal.pone.0083757 (PMC3885444; doi:10.1371/journal.pone.0083757)
Supplement: Table S7 — Output of the ANOVA pattern matching method utilized to identify genes upregulated or downregulated only in generic or only in GA and reference standard. (PDF) [file pone.0083757.s015.pdf]

TABLE S7

UP IN generic ONLY

Gene	ID	Medium vs. Reference Standard pval	Medium vs. GA pval	Medium vs generic pval	Reference Standard vs GA pval	Reference Standard vs generic pval	GA vs. generic pval	AVG Medium	AVG Reference Standard	AVG GA	AVG generic					
2610027L16RIK	ILMN_2750842	0.2331641	0.0599436	2.273E-07	0.6840404	7.939E-05	5.454E-06	7.383123333	7.472973684	7.497494118	7.814259091					
F830002E14RIK	ILMN_1215076	0.6197995	0.9755702	0.0061612	0.5402122	0.001953	0.000886	7.757403333	7.689255263	7.754048529	8.152786364					
TPSAB1	ILMN_2705860	0.8399944	0.1109056	1.247E-05	0.0501649	1.809E-06	5.998E-05	6.547536667	6.53815	6.616708824	6.831309091					
G6PC3	ILMN_1218602	0.3328515	0.0783064	0.0002343	0.4706485	0.0003809	0.0003453	7.870143333	7.920968421	7.948063235	8.117036364					
MGAT4B	ILMN_2609073	0.1690924	0.1032948	4.541E-07	0.7011403	1.508E-05	0.0001364	6.956693333	7.005913158	7.019420588	7.194036364		Pattern Represented by Data:			
GM962	ILMN_2950814	0.4674311	0.0839329	0.0001513	0.2810843	0.0003371	0.0023191	6.877433333	6.909928947	6.952055882	7.109163636					x
SCMH1	ILMN_1230766	0.8582599	0.3394373	6.521E-07	0.4234863	5.525E-07	1.863E-07	7.173013333	7.179326316	7.202801471	7.402677273		x	x	x	
SLC25A39	ILMN_2855261	0.1848461	0.1595645	0.0001106	0.9308916	0.001062	0.0003961	8.332893333	8.396894737	8.393611765	8.557713636					
OLFM1	ILMN_1240615	0.3681708	0.0692345	2.834E-05	0.3872817	5.003E-05	2.899E-05	6.3626	6.397260526	6.422316176	6.583404545		Medium	Reference	GA	generic
SNCA	ILMN_3161601	0.9329083	0.9837928	0.003274	0.9325913	0.0008841	7.921E-05	6.82406	6.8204	6.823285294	7.034854545					
SNCA	ILMN_3136638	0.7854593	0.2568549	0.0032194	0.3721799	0.0020013	0.0010233	7.675763333	7.688563158	7.719423529	7.885086364					
2310003H01RIK	ILMN_1238640	0.1826647	0.5462934	0.0003656	0.2968918	0.0023913	4.452E-05	9.07639	9.134994737	9.099767647	9.279345455					
MARCKS	ILMN_1256142	0.4440551	0.1313548	0.0006546	0.393461	0.0002508	0.000261	9.683343333	9.709323684	9.731372059	9.883690909					
LMAN2L	ILMN_1220769	0.3716363	0.7420142	2.697E-07	0.5049726	8.19E-06	8.75E-08	8.09457	8.121555263	8.103702941	8.290409091					
PDHB	ILMN_3116570	0.0704557	0.0549053	0.0025327	0.8503939	0.0383537	0.0127636	6.709056667	6.794373684	6.787814706	6.90445					
1300001I01RIK	ILMN_1217040	0.1536142	0.4450848	0.0015507	0.4330555	0.0099202	0.0034976	7.676173333	7.747807895	7.714445588	7.870945455					
MARCH2	ILMN_2618329	0.3233988	0.0563239	0.0005482	0.3436832	0.0009105	0.0024831	8.727983333	8.768297368	8.797601471	8.922431818					
REPIN1	ILMN_2605767	0.2862285	0.1495528	0.0001726	0.8071697	8.978E-05	5.649E-05	6.464103333	6.508721053	6.515754412	6.656413636					
ALG12	ILMN_2680686	0.2118823	0.7497599	0.000357	0.1784074	0.0038951	1.338E-05	6.68395	6.743931579	6.696648529	6.871754545					
SMARCAL1	ILMN_2843698	0.3817663	0.686282	1.615E-05	0.5608192	0.0004314	2.681E-05	8.057763333	8.091636842	8.072047059	8.24295					
TRIP10	ILMN_1213117	0.6622554	0.0664756	0.0001387	0.1800981	0.0005739	0.0012161	7.159423333	7.17705	7.219839706	7.344527273					
PHLPP	ILMN_1237430	0.168588	0.5482832	0.0007711	0.3002243	0.0051147	0.0003543	7.11814	7.179828947	7.143044118	7.301213636					
WDR6	ILMN_3162081	0.5273324	0.2902706	0.0003162	0.0559326	1.641E-05	0.0008863	8.723316667	8.696534211	8.763451471	8.904881818					
TRIM28	ILMN_1227225	0.2791056	0.1069045	8.341E-05	0.5627484	0.0002717	0.0011933	11.25953333	11.29731053	11.31465882	11.44073182					
1110039B18RIK	ILMN_2790512	0.9186738	0.710941	0.0001189	0.7716409	2.387E-05	1.023E-05	8.343063333	8.346705263	8.355070588	8.522913636					
CHMP2A	ILMN_2694998	0.0940165	0.0669539	0.0003865	0.8377005	0.0070034	0.023255	9.846833333	9.916607895	9.924004412	10.02553636					
PIGT	ILMN_2868131	0.7670635	0.5978573	0.0001195	0.3869488	6.736E-05	0.0003121	8.51204	8.501307895	8.530822059	8.690481818					
LOC100044636	ILMN_2738554	0.070003	0.11971	4.275E-05	0.7049708	0.0005341	0.0003671	6.805556667	6.867765789	6.857189706	6.983836364					
PLEKHO2	ILMN_2749448	0.8958642	0.2798034	0.0003179	0.2457827	9.769E-06	0.000178	9.02316	9.027347368	9.057944118	9.201381818					
EHBP1L1	ILMN_1227131	0.9593148	0.1198251	0.0015889	0.0903262	0.0004371	0.0099071	7.960713333	7.962668421	8.019623529	8.138863636					
ACSL1	ILMN_2622671	0.365157	0.946755	0.0003272	0.1851208	0.0004249	8.01E-07	7.0019	7.036060526	6.999769118	7.177977273					
2310016E02RIK	ILMN_1214401	0.229809	0.0575322	0.0017387	0.36519	0.0001704	0.0087173	7.304916667	7.353897368	7.379801471	7.479331818					
ZFP42	ILMN_2961604	0.2397652	0.2201366	0.0009048	0.8527101	0.0156875	0.0029835	5.924646667	5.978389474	5.971730882	6.098336364					
LOC100044576	ILMN_2625342	0.1074155	0.1138843	0.0001033	0.9574728	0.0002506	0.0004608	7.004646667	7.055815789	7.054444118	7.175572727					
CLECSF12	ILMN_2663258	0.4492669	0.1316548	0.0007527	0.5144406	0.0024795	0.0017347	6.01422	6.045115789	6.065151471	6.183786364					
RFFL	ILMN_1226372	0.8874501	0.4489858	9.407E-05	0.5463703	0.0001795	5.496E-05	6.479006667	6.483426316	6.499788235	6.647177273					
MEIS1	ILMN_1218266	0.1392653	0.1396504	1.485E-05	0.9115861	0.0003698	0.0010651	6.53368	6.576773684	6.579920588	6.701572727					
EI24	ILMN_2953677	0.2032149	0.0823404	2.761E-05	0.748713	4.686E-05	1.159E-05	8.8673	8.904455263	8.911220588	9.035086364					
DCP1A	ILMN_2783429	0.056901	0.0959854	9.718E-05	0.6071671	0.0077464	0.0009813	8.658053333	8.723913158	8.709516176	8.825331818					
9330147J08RIK	ILMN_1250402	0.120667	0.0713191	8.261E-05	0.955541	0.0053921	0.0011467	6.87715	6.936536842	6.934904412	7.044290909					
NFS1	ILMN_2648867	0.0717225	0.0597558	0.0002784	0.997391	0.0102456	0.0097376	7.170636667	7.236936842	7.236829412	7.337427273					
YWHAG	ILMN_2587515	0.0921654	0.0878016	0.0001853	0.7821902	0.0287325	0.0057803	6.453236667	6.521060526	6.511844118	6.62					
FMNL3	ILMN_2834837	0.6147037	0.2026937	0.0045027	0.4743815	0.0070198	0.0062918	6.104096667	6.130421053	6.1567	6.2686					
ARF3	ILMN_1252195	0.2120794	0.0763958	0.0010909	0.6885059	0.0052983	0.0072429	8.823646667	8.876202632	8.888575	8.987454545					
CSF2RA	ILMN_2866276	0.9670767	0.4561918	0.0068519	0.3656228	0.0020059	7.747E-05	7.395346667	7.397218421	7.364389706	7.558909091					
DHRSX	ILMN_1221451	0.6177083	0.9153302	0.0039053	0.4791138	0.004392	0.0008206	7.434513333	7.457857895	7.429736765	7.596972727					
LOC100046035	ILMN_1242144	0.1676726	0.0515244	0.0010216	0.6672007	0.0210927	0.0309174	5.793826667	5.853584211	5.868417647	5.956231818					
2610019A05RIK	ILMN_2731592	0.7947655	0.2971065	0.0087397	0.1098779	0.0004336	0.0109203	7.04731	7.035371053	7.0915	7.209186364					
SCHIP1	ILMN_2937320	0.4365044	0.5967454	0.0026498	0.7420351	2.214E-05	3.483E-05	6.35611	6.325936842	6.336330882	6.517436364					
4632417N05RIK	ILMN_2886896	0.3358036	0.1749712	0.0003276	0.7062798	0.0008621	0.0016025	6.254436667	6.288605263	6.299548529	6.415672727					
DORZ1	ILMN_2773286	0.5996219	0.3441629	2.367E-05	0.6534002	4.61E-05	0.0001166	6.848643333	6.864202632	6.876232353	7.009645455					
CPNE2	ILMN_2870487	0.3806505	0.505551	0.0005026	0.7748864	0.0013328	0.0003384	6.59805	6.627957895	6.619660294	6.758777273					

GPD1L	ILMN_2991214	0.1997722	0.178415	0.0042358	0.9835298	0.0176093	0.010363	5.962863333	6.012323684	6.011677941	6.122713636					
BC018242	ILMN_2757716	0.1034275	0.1944064	0.0010031	0.4484675	0.0133763	0.0006339	6.208366667	6.270368421	6.249745588	6.367659091					
C230090D14	ILMN_2760468	0.1391611	0.1359473	0.0003776	0.7852333	0.0077452	0.0008987	5.947733333	5.998884211	5.99155	6.106377273					
TSPAN31	ILMN_2964076	0.6180744	0.5437157	0.0117624	0.1745861	0.0079256	0.0001926	6.763363333	6.787294737	6.736430882	6.921695455					
POLB	ILMN_2789425	0.21319	0.1631492	0.0003315	0.8404281	0.0038193	0.009116	6.965993333	7.0099	7.016457353	7.124222727					
SIAH1B	ILMN_2903734	0.1289666	0.5780849	0.0028784	0.1912821	0.0358219	0.0012247	6.37228	6.436939474	6.393475	6.528490909					
LOC383036	ILMN_2536553	0.1482323	0.0774628	0.000893	0.9606867	0.0431433	0.0108233	6.41209	6.474181579	6.472569118	6.567927273					
LYNX1	ILMN_1258942	0.4077937	0.1416243	0.0015633	0.4557018	0.0014763	0.0111326	6.817296667	6.848018421	6.870923529	6.972218182					
ZFP41	ILMN_3137980	0.2677737	0.8515377	0.0002013	0.2293824	0.0012063	7.766E-06	7.414063333	7.450618421	7.419467647	7.568736364					
TSPAN33	ILMN_1219904	0.3878386	0.0719326	0.001134	0.3407473	0.003853	0.0131555	6.582713333	6.613352632	6.641277941	6.735368182					
MEGF8	ILMN_2685985	0.0926907	0.2228606	0.0001841	0.2853957	0.010053	2.972E-05	7.129433333	7.184897368	7.159761765	7.281790909					
GPAA1	ILMN_1255423	0.8857298	0.2373073	0.0110271	0.1266454	0.0022423	0.0336273	7.391883333	7.385360526	7.441967647	7.544040909					
SLC7A8	ILMN_2598877	0.4887311	0.3156157	0.0025282	0.7614574	0.0036332	0.0037823	6.23386	6.259007895	6.268110294	6.384822727					
RHOF	ILMN_2992047	0.7851793	0.0647153	0.0010468	0.067057	0.0001766	0.005019	9.890486667	9.899973684	9.947642647	10.04121364					
2810453I06RIK	ILMN_2510771	0.1564431	0.1008447	0.0032368	0.9907679	0.0184046	0.0061613	8.4568	8.506107895	8.506408824	8.607172727					
PCDHAC1	ILMN_2980110	0.0595308	0.2312302	0.0003196	0.3053608	0.0147271	0.0005897	5.65515	5.716521053	5.689845588	5.804577273					
SCN1B	ILMN_2834198	0.8275725	0.9374337	0.0010911	0.8649437	0.0010695	0.0004465	5.966606667	5.975315789	5.969526471	6.115209091					
CDC42BPB	ILMN_2867696	0.2957513	0.9359791	0.0007209	0.2637103	8.89E-06	0.0001107	7.155186667	7.118955263	7.152523529	7.303131818					
BC037704	ILMN_2470360	0.677388	0.7421918	0.0001022	0.3876608	0.0001132	1.483E-06	7.20203	7.214631579	7.193013235	7.3494					
ADAR	ILMN_3021272	0.0580027	0.0512931	0.0005744	0.9625952	0.0334299	0.0278485	8.79254	8.859055263	8.857651471	8.939627273					
UACA	ILMN_2492716	0.3533152	0.1224782	0.0003107	0.627983	0.0021344	0.0012063	6.05639	6.087271053	6.099835294	6.203304545					
HIST2H2AA1	ILMN_1228849	0.0884511	0.1005373	0.0004786	0.9320942	0.0153242	0.0150984	6.169153333	6.226644737	6.224104412	6.315718182					
PRDX2	ILMN_2773554	0.1385282	0.1338746	0.0002502	0.8935302	0.0062484	0.0019358	11.56810333	11.61388947	11.61041618	11.71427273					
TFRC	ILMN_2619848	0.085162	0.0916788	0.0022929	0.6998758	0.016039	0.0059997	9.730386667	9.798923684	9.788595588	9.876177273					
MTAP1S	ILMN_2657432	0.6904967	0.2003168	0.0006583	0.0590439	8.877E-05	0.0020646	8.951766667	8.937260526	8.991608824	9.097072727					
D11WSU47E	ILMN_2700833	0.225963	0.1430469	0.000121	0.8628336	0.0036318	0.0031777	7.024173333	7.063092105	7.067933824	7.168595455					
ADPRHL2	ILMN_2639063	0.6840518	0.3473478	0.0053945	0.1186338	0.0008991	0.0054003	6.74877	6.730905263	6.782579412	6.892372727					
SYMPK	ILMN_1245437	0.7197743	0.5936817	0.0010108	0.891984	0.0060061	0.002917	8.383586667	8.397528947	8.402226471	8.52715					
GYPA	ILMN_2696696	0.7389014	0.3632769	0.0251901	0.4987125	0.0031931	0.0003548	6.934123333	6.9204	6.897686765	7.0776					
KLHDC3	ILMN_2851023	0.783022	0.2437231	0.0037638	0.269885	0.0004288	0.0080675	7.81348	7.822594737	7.853058824	7.955713636					
3110070M22RIK	ILMN_2696066	0.1073796	0.2050209	0.0014511	0.4866036	0.0398205	0.0035237	6.120523333	6.180081579	6.160217647	6.262686364					
TRUB1	ILMN_1240735	0.2953657	0.0734389	9.239E-05	0.5610317	0.0008692	0.0006353	8.182053333	8.2163	8.230364706	8.323472727					
AKT2	ILMN_2655895	0.8442971	0.2577229	4.546E-05	0.3437085	4.35E-05	1.532E-07	5.950246667	5.944173684	5.919175	6.091436364					
RSC1A1	ILMN_1230668	0.6840115	0.0539208	0.0004297	0.0858665	0.0001366	0.0020164	7.647993333	7.659592105	7.697304412	7.787868182					
FRY	ILMN_2623288	0.4184333	0.1716296	0.0008519	0.5808814	0.0035019	0.005631	5.699756667	5.725107895	5.740185294	5.839477273					
TJAP1	ILMN_2934448	0.4648067	0.4800185	0.0005054	0.1039589	0.0031371	7.38E-06	8.189586667	8.215047368	8.167745588	8.328781818					
CNGA4	ILMN_2875896	0.4128026	0.838024	0.0042111	0.4856524	0.0262685	0.0031015	5.99744	6.028623684	6.004719118	6.136186364					
SGSM3	ILMN_2987294	0.2852907	0.1799385	0.000553	0.8524482	0.0023816	0.0020158	7.17779	7.212339474	7.217123529	7.316372727					
9430078K10RIK	ILMN_2500041	0.5504554	0.1164212	0.0017546	0.3083079	0.0041732	0.0165627	6.076453333	6.095631579	6.124530882	6.214954545					
SLC26A6	ILMN_2668261	0.3512318	0.3748397	0.0020554	0.9305283	0.0138219	0.0112631	6.420846667	6.4567	6.453741176	6.5591					
LYSMD4	ILMN_2701435	0.3263893	0.7070625	0.0012552	0.3678655	0.0028218	6.468E-05	6.801406667	6.835131579	6.812473529	6.939245455					
PCBD2	ILMN_2876775	0.838579	0.0685734	0.0009368	0.0565928	7.067E-05	0.0052888	10.26506	10.27223684	10.32058088	10.40271364					
UAP1	ILMN_2502542	0.2165725	0.9854187	0.0009075	0.1340454	0.0318819	0.0002473	8.167053333	8.216581579	8.167673529	8.304695455					
RNF40	ILMN_1242623	0.211788	0.099174	0.0001084	0.6579531	0.0001075	0.0007106	6.104593333	6.138042105	6.147527941	6.241636364					
LOC277049	ILMN_1240399	0.2934571	0.1060159	0.0003221	0.5755921	0.0029932	0.005663	5.871153333	5.901181579	5.915414706	6.006836364					
LOC100047603	ILMN_2697057	0.5317771	0.4972328	0.0027997	0.9033529	0.0040861	0.000286	6.163923333	6.187207895	6.184048529	6.299472727					
BCL2L12	ILMN_2935909	0.4501663	0.1507583	0.0008312	0.5408152	0.0049174	0.0069966	6.95393	6.980221053	6.997483824	7.088572727					
HMGCL	ILMN_1256234	0.2979737	0.3874272	0.0028899	0.8735481	0.0135305	0.0202339	7.923806667	7.963534211	7.958	8.0583					
TRPV4	ILMN_2466284	0.2514505	0.3380365	0.0003131	0.6827345	0.0188364	0.002112	5.866016667	5.907376316	5.895151471	5.999986364					
9030624J02RIK	ILMN_2872639	0.782004	0.4170399	0.0001144	0.603075	0.0003567	0.0003189	9.494316667	9.503005263	9.516620588	9.627690909					
1110020P15RIK	ILMN_3142224	0.1212065	0.0502281	3.997E-06	0.9858039	0.0010157	5.228E-05	12.83764667	12.8778	12.87814853	12.97065					
SNCA	ILMN_3059393	0.2626604	0.2088617	4.536E-05	0.8934317	0.0030156	0.0039725	5.838986667	5.871242105	5.874923529	5.971345455					
9130011J15RIK	ILMN_2593484	0.2364945	0.4354212	0.0007902	0.4843251	0.0049498	0.0001633	8.423743333	8.460226316	8.444064706	8.556095455					
ATP9B	ILMN_1233770	0.8250675	0.1176255	0.0007899	0.1465289	0.0004474	0.0031797	5.876853333	5.884023684	5.920125	6.008981818					
KCNC3	ILMN_2681742	0.372855	0.7150701	0.0018064	0.4859011	0.0082036	0.0005546	5.950906667	5.980818421	5.961808824	6.082136364					
LOC100045460	ILMN_1216433	0.5101012	0.3577678	0.0010288	0.0673625	3.894E-05	0.0009365	5.620116667	5.600318421	5.645004412	5.749863636					
MAGEE2	ILMN_2477690	0.1710845	0.05934	0.0011835	0.6011098	0.007276	0.0102496	5.760393333	5.796631579	5.808379412	5.889972727					
LOC100047194	ILMN_1217481	0.1593328	0.0981729	1.405E-05	0.9398443	0.0118878	0.0069427	5.874716667	5.918439474	5.920526471	6.00425					
MS4A7	ILMN_3091003	0.5729752	0.4820176	0.0137711	0.8972357	0.0231728	0.0210323	6.47951	6.501352632	6.505692647	6.608881818					
EFCAB6	ILMN_2928498	0.7632342	0.3221721	0.0043956	0.4863548	0.0034302	0.002517	5.580346667	5.591255263	5.609620588	5.709145455					

CEBPE	ILMN_3029849	0.8078374	0.1732849	0.0030771	0.2318701	0.002828	0.0162265	6.161636667	6.170128947	6.20425	6.289590909							
LOC100046781	ILMN_1249142	0.6583194	0.8334352	0.0014968	0.468952	0.0038563	0.0003991	6.09563	6.110731579	6.088780882	6.223009091							
C920004C08RIK	ILMN_1222071	0.3850288	0.0700544	0.0038178	0.3880992	0.0125303	0.0225901	11.32737	11.35911842	11.38204412	11.45473636							
TNXB	ILMN_2444411	0.1496148	0.1054688	0.0005018	0.8805468	0.002138	0.0019133	5.663876667	5.699578947	5.702611765	5.790745455							
ADAL	ILMN_2948663	0.2369654	0.1716393	0.0033817	0.9717648	0.0463896	0.0180219	6.324213333	6.367818421	6.366766176	6.450545455							
INTS7	ILMN_2726174	0.2732407	0.586879	0.0058704	0.4179798	0.0040954	0.0010741	9.293426667	9.331626316	9.311188235	9.41915							
FOXP4	ILMN_2650317	0.7459436	0.5627063	0.0170549	0.7551376	0.0023531	0.005287	6.264146667	6.276007895	6.284380882	6.389745455							
Ageneric5L	ILMN_1217612	0.8329932	0.9146392	0.0004805	0.6953516	3.709E-05	1.483E-05	8.109116667	8.115305263	8.106152941	8.234490909							
CD44	ILMN_2474229	0.2692964	0.1478198	0.0055493	0.9255312	0.044715	0.0173939	5.908876667	5.950365789	5.953	6.034186364							
ZMAT3	ILMN_2418426	0.2565844	0.8631643	0.0022322	0.0994823	0.026959	8.304E-05	7.620363333	7.659939474	7.615426471	7.745318182							
PRPF3	ILMN_2941431	0.480392	0.0666872	0.0019168	0.1846098	0.0003537	0.0072291	9.9884	10.01081053	10.04008088	10.11331818							
RPUSD3	ILMN_1214740	0.2304027	0.2327958	0.0025548	0.8325719	0.0169034	0.004917	6.720803333	6.761342105	6.755892647	6.845586364							
ZBTB6	ILMN_2525819	0.1308718	0.1225869	0.001417	0.6993416	0.0195411	0.0014442	5.991976667	6.036765789	6.028576471	6.116695455							
MAGEA9	ILMN_1252512	0.8309473	0.0845974	0.0006887	0.1188421	0.0013866	0.0180155	5.76786	5.7748	5.817098529	5.892554545							
PTPRF	ILMN_1219189	0.2190425	0.0867244	0.0005635	0.5640646	0.0042956	0.0157663	5.6626	5.695144737	5.709207353	5.786977273							
NSF	ILMN_2760629	0.1931859	0.360736	0.0008575	0.5595835	0.0051887	0.0009574	9.924636667	9.963881579	9.949961765	10.04892273							
SARDH	ILMN_2903455	0.7960502	0.7785859	0.0101605	0.51361	0.0013035	0.0028363	5.826933333	5.817136842	5.836519118	5.949331818							
XPO5	ILMN_1216198	0.7050569	0.679202	0.0238434	0.3306391	0.042049	0.0005737	7.6071	7.625202632	7.592019118	7.729186364							
VGLL2	ILMN_2495753	0.3493614	0.2615197	0.0061246	0.8419134	0.0101935	0.024173	5.813743333	5.846973684	5.852779412	5.935795455							
HSD3B2	ILMN_1233611	0.3857508	0.8465226	0.0001419	0.1997045	0.0047757	4.855E-06	5.780146667	5.805763158	5.775658824	5.9017							
CAPZB	ILMN_1230100	0.1875435	0.0603499	0.0021735	0.6758285	0.0277333	0.0323886	5.8003	5.843094737	5.853869118	5.921709091							
PKIB	ILMN_3052870	0.7009613	0.2792825	0.0002337	0.4501659	0.0004146	0.0035889	5.772683333	5.783521053	5.802783824	5.894004545							
A230005A19RIK	ILMN_2564767	0.6380251	0.1891714	2.648E-05	0.0553869	1.159E-05	8.044E-05	5.803403333	5.79155	5.830422059	5.924704545							
USP10	ILMN_1217931	0.7946592	0.8388046	0.0096273	0.934	0.0095534	0.0064299	6.42393	6.434371053	6.431577941	6.544827273							
NCDN	ILMN_1231405	0.8169843	0.416902	0.0066776	0.2035153	0.0002209	0.0077846	8.381273333	8.373278947	8.408173529	8.501877273							
SYN1	ILMN_1235133	0.6694849	0.2578995	0.0045018	0.0779335	0.0008223	0.0099512	5.950843333	5.935671053	5.984697059	6.071295455							
ARVCF	ILMN_2573293	0.4469461	0.7566684	0.0010282	0.6294586	0.003341	0.0008631	5.718253333	5.738763158	5.726697059	5.838531818							
SMG7	ILMN_2953017	0.4258797	0.3806893	0.0188944	0.9166145	0.0218454	0.0080269	9.624156667	9.658326316	9.655355882	9.744204545							
5730472N09RIK	ILMN_2765936	0.4083926	0.6082371	0.0019425	0.6700575	0.0125348	0.0017913	7.63479	7.660244737	7.648992647	7.754604545							
ZMIZ1	ILMN_1224736	0.3763442	0.055049	0.0136689	0.3984977	0.0022425	9.798E-06	7.174863333	7.131644737	7.099030882	7.294513636							
ZFP162	ILMN_1251244	0.2014047	0.7434902	0.003566	0.2275352	1.516E-05	0.0001463	7.66155	7.620365789	7.651876471	7.781118182							
DUS2L	ILMN_1256843	0.8561644	0.446428	0.0038192	0.2785569	0.0008641	0.0017291	6.93203	6.926063158	6.953073529	7.050854545							
BZRPL1	ILMN_2635718	0.6185068	0.57423	0.0190054	0.9031811	0.0430169	0.0044684	6.187326667	6.206431579	6.203076471	6.306068182							
ARFGAP2	ILMN_2853409	0.8237825	0.8515888	0.001996	0.9447898	0.0067887	0.0013	9.42326	9.430544737	9.428569118	9.541945455							
9330183F02RIK	ILMN_2558866	0.3261468	0.1733477	0.0005571	0.6332639	0.0058003	0.0168183	5.949093333	5.973960526	5.98625	6.067668182							
DBF4	ILMN_2952661	0.6240393	0.8384865	0.0122907	0.3985923	0.0191309	0.0016656	8.57731	8.598042105	8.569597059	8.695836364							
ATG3	ILMN_2595188	0.6213315	0.9031596	0.0084176	0.5772204	0.0012036	0.0002088	5.596373333	5.578073684	5.592863235	5.714631818							
ISCA1	ILMN_2888842	0.3595758	0.7809579	0.0076209	0.4365809	0.0372539	0.0050363	9.365886207	9.401326316	9.377892647	9.484090909							
4930544L04RIK	ILMN_1256258	0.1212117	0.1444176	0.0006835	0.7308269	0.0319887	0.0066889	5.835473333	5.881492105	5.873111765	5.953290909							
2610209A20RIK	ILMN_2744228	0.222251	0.0503501	0.0038665	0.6307725	0.0388641	0.030015	5.954303333	5.9957	6.007576471	6.072068182							
2810046M22RIK	ILMN_2714278	0.7932571	0.3413647	0.0069021	0.4306555	0.0021317	0.0072284	5.965796667	5.974528947	5.994625	6.08355							
DHDH	ILMN_1234086	0.7472445	0.5905975	0.0017523	0.8697156	0.0002079	4.284E-06	7.466183333	7.457073684	7.453639706	7.583509091							
ALOX15	ILMN_2789023	0.4945088	0.5363894	0.005707	0.910931	0.0080107	0.005256	5.948553333	5.970678947	5.967667647	6.065718182							
RNF183	ILMN_2753931	0.2692191	0.1884299	0.0068692	0.88654	0.0201168	0.0253897	5.951953333	5.990247368	5.994061765	6.069068182							
LOC385989	ILMN_2538010	0.186073	0.2002142	0.0019194	0.7581627	0.0262803	0.0047078	5.9626	6.003389474	5.996039706	6.079709091							
RBPMS	ILMN_3026557	0.9676317	0.5622495	0.0034885	0.5434605	0.0007213	8.217E-06	6.082373333	6.081173684	6.068086765	6.199422727							
MTRR	ILMN_2969314	0.138014	0.2808236	0.0027479	0.4487716	0.0454004	0.0033295	6.4451	6.491657895	6.473463235	6.562140909							
5930400G23RIK	ILMN_1252245	0.4046753	0.4652826	0.0034776	0.8465238	0.0314305	0.0114345	5.852196667	5.880894737	5.875044118	5.968995455							
CD151	ILMN_2492403	0.9211922	0.6734808	0.0126171	0.5508714	0.0052828	0.0014538	7.20143	7.205263158	7.185635294	7.318127273							
LOC380636	ILMN_2533773	0.1214529	0.0880914	0.0005543	0.9948424	0.0233225	0.0116027	5.523523333	5.565797368	5.565647059	5.639413636							
ITFG2	ILMN_1252621	0.7616105	0.4091743	0.0012221	0.1959224	0.0002063	0.0008307	8.126836667	8.117552632	8.148073529	8.242622727							
PALM	ILMN_2815626	0.2721038	0.6017592	0.0070254	0.0526414	9.931E-05	0.0046269	6.404466667	6.3666	6.420661765	6.519804545							
ZFP563	ILMN_2642938	0.193144	0.3057901	0.0038908	0.6058345	0.0392958	0.0064416	5.780793333	5.821705263	5.808876471	5.894754545							
LOC382813	ILMN_1237743	0.3244602	0.1328203	0.0011393	0.6695764	6.759E-05	1.98E-06	5.826046667	5.796476316	5.785617647	5.939763636							
BC017612	ILMN_2631514	0.8355212	0.8501323	0.0180931	0.6600593	0.0099305	0.0082377	7.401256667	7.409202632	7.393575	7.514886364							
CBARA1	ILMN_2686513	0.8613971	0.9466614	0.0179951	0.8857374	0.0071062	0.0037732	7.51929	7.512352632	7.516942647	7.632227273							
COQ9	ILMN_2774456	0.5907585	0.1375329	0.0008612	0.3490039	0.0062178	0.027967	8.24214	8.258726316	8.284261765	8.355045455							
USP36	ILMN_2491232	0.5694926	0.3860962	0.0302749	0.073112	0.0419255	0.0001784	8.529063333	8.552176316	8.499982353	8.641940909							
1700063H04RIK	ILMN_1244141	0.6351553	0.1080972	0.0089017	0.2270816	0.0088517	0.0343723	5.77347	5.789544737	5.819770588	5.886254545							
JPH1	ILMN_1217728	0.3436531	0.0971616	0.0031993	0.5357723	0.0137897	0.0215332	5.62534	5.656123684	5.671236765	5.737745455							

CLEC4A3	ILMN_3161652	0.1128241	0.6222873	0.048441	0.1166056	0.0002655	0.002175	6.565526667	6.496394737	6.547061765	6.6777					
R74862	ILMN_2745522	0.4313981	0.3826213	0.0063334	0.0565743	0.0001985	0.0157434	6.187246667	6.161294737	6.214616176	6.299313636					
LOC332300	ILMN_2531139	0.2162829	0.4508863	0.0005121	0.6320164	0.012255	0.0042856	6.22264	6.254697368	6.24265	6.334690909					
SPEN	ILMN_2592059	0.1706673	0.9670643	0.0234085	0.0626456	0.0001212	0.003662	6.88912	6.833476316	6.890566176	7.000954545					
CHRM2	ILMN_2971508	0.5509973	0.4033312	0.0014326	0.1197492	0.0025528	5.038E-05	5.612146667	5.627805263	5.589489706	5.723913636					
PSG22	ILMN_3078396	0.1576577	0.4661479	0.0017338	0.4117763	0.0316097	0.0031665	5.674963333	5.713052632	5.693373529	5.786663636					
E330037I15RIK	ILMN_2584027	0.1399458	0.9010308	0.0011689	0.0754758	0.0156828	7.005E-05	6.253253333	6.295521053	6.256348529	6.364609091					
SCAVENGER RECEP	ILMN_2449412	0.0642316	0.5114602	0.0108453	0.0895074	2.556E-07	4.55E-05	6.180263333	6.122484211	6.160936765	6.291545455					
UBE2E2	ILMN_2792485	0.6643433	0.9521774	0.0065048	0.6481448	0.0020282	0.0010459	6.42196	6.408394737	6.420322059	6.533227273					
PALLD	ILMN_1246642	0.1687838	0.2770729	0.0007381	0.6821295	0.0331192	0.0098346	5.6114	5.651594737	5.640960294	5.721868182					
REXO1	ILMN_1245568	0.5580595	0.8330207	0.014408	0.3357191	0.0019289	0.0069221	6.846273333	6.8226	6.853654412	6.956654545					
9330104G04RIK	ILMN_1255975	0.3930281	0.0522333	0.0001905	0.2719844	0.0027408	0.0173777	5.66529	5.685615789	5.709814706	5.775472727					
PKIA	ILMN_2674122	0.6729327	0.4629903	0.0055646	0.1917034	0.001088	0.0121554	6.33296	6.318071053	6.356127941	6.442677273					
1200003C05RIK	ILMN_2943319	0.5962227	0.1430227	0.0119258	0.2948523	0.008537	0.0423517	9.058593333	9.076478947	9.102913235	9.168109091					
C130040D06RIK	ILMN_2746366	0.8027254	0.2275198	0.002847	0.3644168	0.0099522	0.0157612	5.67662	5.685352632	5.710569118	5.78585					
MOCOS	ILMN_2906489	0.7554293	0.3290696	0.0035014	0.4976038	0.0012892	6.205E-05	6.452413333	6.442544737	6.424154412	6.561495455					
PCOLN3	ILMN_2847502	0.6632454	0.5060699	0.0423528	0.151621	0.0012584	0.0315441	7.14645	7.129347368	7.171211765	7.2555					
DACH1	ILMN_3072197	0.336216	0.3550348	0.0011032	0.918376	0.0090052	0.0066616	5.84774	5.876168421	5.873570588	5.956586364					
B930062B16RIK	ILMN_1220808	0.3279033	0.0849729	0.0021703	0.4370566	0.0072341	0.0282052	5.9928	6.020252632	6.038070588	6.1015					
UGP2	ILMN_1244631	0.7981753	0.5705628	0.0036586	0.762371	0.0048909	0.0038064	7.75135	7.759273684	7.767085294	7.859927273					
RALGPS2	ILMN_2884869	0.8377346	0.6456649	0.0075916	0.7776449	0.0017181	0.0021237	8.849346667	8.85575	8.862479412	8.957890909					
MFSD7A	ILMN_2766105	0.606939	0.5851336	0.0336777	0.9448507	0.030403	0.0121466	5.942556667	5.962721053	5.960813235	6.050986364					
KREMEN2	ILMN_2951946	0.975727	0.8670627	0.0142853	0.8263083	0.0101001	0.0023333	6.228356667	6.229486842	6.222888235	6.336777273					
LOC223628	ILMN_1216043	0.2465434	0.8890929	0.0015627	0.2386608	0.0095206	0.0008551	5.593906667	5.627542105	5.597889706	5.702259091					
RASAL2	ILMN_1233634	0.1387857	0.2168454	0.0007663	0.6639452	0.0174822	0.003335	5.9777	6.015571053	6.006291176	6.085986364					
OLFR1155	ILMN_1226210	0.6000859	0.2672726	0.0004658	0.5671312	0.0016383	0.0023021	5.764156667	5.776971053	5.789191176	5.872309091					
1700021P22RIK	ILMN_1237313	0.7232012	0.4362857	0.008366	0.6174392	0.0039999	0.0189846	6.012316667	6.023542105	6.037217647	6.120245455					
9430088N01RIK	ILMN_1251471	0.1831025	0.1656123	0.0028294	0.9647848	0.0093246	0.0154928	6.635966667	6.672292105	6.673269118	6.743536364					
MKNK1	ILMN_2547867	0.3374949	0.3062801	0.0131083	0.8878885	0.0368585	0.0132006	6.082133333	6.116702632	6.113097059	6.189422727					
FAHD1	ILMN_1218891	0.6092822	0.6533548	0.0055864	0.9562869	0.0062643	0.006276	7.16378	7.178176316	7.176764706	7.270995455					
4930432K21RIK	ILMN_2672745	0.6492195	0.8623249	0.0152987	0.6952291	0.0204309	0.0032181	6.587463333	6.603065789	6.592614706	6.694659091					
KRTCAP2	ILMN_2605694	0.4817597	0.2651441	0.0010779	0.7277237	0.0009224	0.0005065	12.50929	12.52731053	12.5338	12.61621818					
LY96	ILMN_2743003	0.6024023	0.371568	0.0249588	0.1045283	0.004204	0.038316	7.739873333	7.721665789	7.768026471	7.846718182					
TAF3	ILMN_3160989	0.4251126	0.8744385	0.0050661	0.2755327	0.0121976	0.0014985	7.20446	7.230068421	7.199411765	7.310913636					
1200011M11RIK	ILMN_3105117	0.8028801	0.6793745	0.0190643	0.4689948	0.0304991	0.0036746	6.336033333	6.345536842	6.321513235	6.442459091					
4930430K04RIK	ILMN_1250290	0.3754528	0.7207378	0.0076223	0.3831359	0.0119278	0.0003522	5.489846667	5.518073684	5.499239706	5.596013636					
FGR	ILMN_1244821	0.9510677	0.4158214	0.0056185	0.4422159	0.0107364	0.0274257	6.94495	6.947236842	6.971538235	7.051018182					
5830457O10RIK	ILMN_2697766	0.361932	0.537811	0.0281625	0.699919	6.469E-05	0.0008658	9.167493333	9.133463158	9.144754412	9.273427273					
FBXL21	ILMN_2518214	0.1497671	0.0756538	0.0016251	0.8671486	0.0255282	0.0205123	5.61257	5.652573684	5.656242647	5.718277273					
BC031853	ILMN_2764549	0.0506383	0.6472627	0.0043154	0.0929544	4.811E-06	0.0007184	6.96577	6.904671053	6.951757353	7.071477273					
LOC546088	ILMN_2533350	0.7282267	0.4448711	0.0092865	0.688774	0.0170651	0.015125	5.760926667	5.771278947	5.781745588	5.866559091					
control_ILMN_137	ILMN_1378661	0.5828834	0.5621896	0.0011555	0.9872119	0.0006434	0.0002255	5.73423	5.717705263	5.718148529	5.839836364					
HTATIP2	ILMN_2603834	0.4745323	0.2765483	0.0014177	0.7640664	0.002103	0.0016185	8.76101	8.781044737	8.787325	8.866609091					
CENTD3	ILMN_1243107	0.3065887	0.184697	0.0070268	0.9053401	0.0005143	2.391E-05	7.796676667	7.763389474	7.760257353	7.902195455					
DNAHC17	ILMN_1215848	0.7856079	0.9473589	0.000427	0.7754273	0.0010472	2.065E-05	5.836333333	5.843681579	5.837757353	5.941763636					
5330403O16RIK	ILMN_1241724	0.2308837	0.145484	0.0038019	0.904373	0.0419075	0.0260954	5.679523333	5.713576316	5.716461765	5.784904545					
CCL2	ILMN_1245710	0.6811594	0.351608	0.0180609	0.1548173	0.0060155	0.0445802	5.806643333	5.794926316	5.833814706	5.911954545					
C230062L16RIK	ILMN_2582211	0.7945817	0.8305364	0.0073561	0.9112243	0.0102627	0.0014034	6.18329	6.191994737	6.189144118	6.288490909					
1700029G01RIK	ILMN_1250435	0.7618226	0.9723702	0.0103286	0.6868261	0.0101158	0.0025475	6.738916667	6.749055263	6.737847059	6.844031818					
9330158H04RIK	ILMN_2660943	0.6451259	0.5227084	0.0015255	0.9536512	0.0075022	0.0008851	5.684246667	5.697526316	5.698854412	5.789281818					
ENAH	ILMN_2636853	0.0686846	0.3305665	0.0016337	0.3484794	0.0462945	0.0081829	5.894096667	5.941060526	5.919310294	5.999045455					
SLC13A5	ILMN_1229923	0.9450998	0.6092523	0.0184531	0.4930353	0.004652	0.0070172	5.689653333	5.687397368	5.704489706	5.794518182					
6720407G21RIK	ILMN_1245282	0.8866404	0.4018841	0.0006808	0.4680024	0.0002287	1.804E-06	5.56161	5.557984211	5.543336765	5.666440909					
BTRC	ILMN_3100042	0.3447294	0.6781052	0.0128209	0.5299481	0.0002986	0.0014399	7.959056667	7.927918421	7.945702941	8.063472727					
COTL1	ILMN_2915232	0.7949203	0.5558348	0.0071832	0.7739003	0.015121	0.0075438	12.2261	12.23490263	12.24276029	12.33050455					
NEK4	ILMN_2634859	0.915262	0.1931111	0.0057833	0.1244512	0.0029297	0.0329518	5.931456667	5.927926316	5.968935294	6.035831818					
6430709H04RIK	ILMN_2609850	0.8978011	0.4153561	0.001214	0.5080157	0.0039779	0.0029373	5.620953333	5.625018421	5.641717647	5.725077273					
TCEAL5	ILMN_2630772	0.9721959	0.442065	0.0055604	0.36221	0.0015002	0.0107663	5.74579	5.744702632	5.768248529	5.849777273					
NFE2L1	ILMN_1251729	0.351511	0.8846935	0.0009589	0.3619295	0.0073121	0.0004048	9.749116667	9.772821053	9.752560294	9.852936364					
THOC1	ILMN_2850527	0.8385566	0.4887052	0.0199078	0.6175407	0.013684	0.0108481	8.791263333	8.7994	8.813451471	8.894681818					

CTNNA3	ILMN_2834525	0.3536454	0.3754631	0.0119809	0.8661483	0.0305254	0.0104644	5.677696667	5.704381579	5.700572059	5.780836364					
9430003J03RIK	ILMN_1258022	0.6557114	0.28191	0.0019528	0.4896149	0.0018663	0.0071396	6.223606667	6.235028947	6.250230882	6.326727273					
NAPRT1	ILMN_2799590	0.9601419	0.3307608	0.0079905	0.2665183	0.006164	0.0003234	6.49092	6.492510526	6.460433824	6.593895455					
SYNGR1	ILMN_3101732	0.9391257	0.3508257	0.009843	0.3004176	0.0132862	0.0303686	5.925626667	5.922781579	5.953997059	6.028540909					
LOC380723	ILMN_1243160	0.3774093	0.6761875	0.0014385	0.516955	0.0077931	0.0002965	5.8033	5.824481579	5.811860294	5.906168182					
D130017N08RIK	ILMN_1247385	0.1666711	0.4830741	0.0101915	0.4217883	0.0440035	0.0140436	6.040376667	6.081463158	6.061413235	6.142936364					
LOC677528	ILMN_2656362	0.4798284	0.945782	0.0071564	0.4145076	0.0002681	0.0010366	9.500013333	9.476392105	9.497989706	9.602481818					
BCL2L2	ILMN_2551387	0.2281858	0.2152442	0.0047362	0.9737233	0.0130062	0.0159752	5.758356667	5.792813158	5.792060294	5.86055					
ZEB2	ILMN_1227793	0.6576569	0.5718117	0.0004937	0.9218797	0.0019558	0.0010995	5.58955	5.600247368	5.602375	5.691709091					
UBE2A	ILMN_2476804	0.8177897	0.9500046	0.0174358	0.7405797	0.0172227	0.0118181	7.649666667	7.640873684	7.651729412	7.751495455					
BC040758	ILMN_3034303	0.2893187	0.3769258	0.005221	0.7981817	0.037212	0.0192599	6.051266667	6.083378947	6.076677941	6.153059091					
FOXE1	ILMN_3163279	0.8430818	0.9489308	0.0179908	0.7493004	0.0116943	0.0050916	5.774953333	5.767334211	5.776966176	5.876681818					
BCKDK	ILMN_1249301	0.2175888	0.9222956	0.0155004	0.1187378	8.623E-05	0.0058096	9.482256667	9.443044737	9.485333824	9.583913636					
TRIM15	ILMN_1246816	0.6897495	0.1295912	0.0020197	0.2301412	0.0022952	0.0177117	5.583353333	5.593910526	5.620164706	5.684890909					
HOXC10	ILMN_2836885	0.8983767	0.7071051	0.0314001	0.5904321	0.0430888	0.0058908	6.14456	6.149455263	6.131916176	6.246045455					
NFKB1	ILMN_2592476	0.7689871	0.9504177	0.0029537	0.6379058	0.0079048	6.316E-05	11.57260667	11.58177368	11.57118529	11.67374091					
OLFR228	ILMN_2818216	0.2773733	0.6197379	0.0024911	0.0611951	6.157E-05	0.0029144	5.64159	5.606563158	5.655326471	5.742690909					
CLCN1	ILMN_2802672	0.4944629	0.9550135	0.0048403	0.4679323	0.0412931	0.0034553	5.78522	5.807239474	5.786810294	5.886245455					
6330579M01RIK	ILMN_1256365	0.375962	0.8389799	0.0018333	0.2222726	0.0075945	0.0001729	5.81892	5.839239474	5.814498529	5.919422727					
SFRS2IP	ILMN_2713448	0.7374842	0.4155913	0.0163379	0.1530212	0.0073823	7.545E-05	9.258286667	9.270178947	9.233055882	9.358495455					
4930543G11RIK	ILMN_1233640	0.8229463	0.6641014	0.0022123	0.4370222	0.0015111	1.198E-05	5.784193333	5.789855263	5.775158824	5.88425					
2900090M10RIK	ILMN_1260170	0.2843888	0.1051492	0.0006685	0.7017619	0.0214413	0.0209832	5.65543	5.685610526	5.694514706	5.755240909					
LOC100048331	ILMN_1242041	0.5890128	0.8478217	0.0260322	0.3738719	0.029814	0.0038906	5.8999	5.918623684	5.893625	5.999568182					
TFIP11	ILMN_2661666	0.9021335	0.1601949	0.0059321	0.0744788	0.0006418	0.042188	8.054953333	8.051084211	8.095283824	8.154390909					
TTC4	ILMN_1220820	0.5058343	0.9987373	0.0157986	0.4538196	0.0009629	0.0059389	8.308693333	8.288452632	8.308645588	8.408109091					
CAMK1	ILMN_2944939	0.1046081	0.1579896	0.0245902	0.5280152	5.12E-05	1.848E-05	6.459003333	6.399568421	6.416069118	6.557886364					
2600005J22RIK	ILMN_1246515	0.8404354	0.1647097	0.0093088	0.1506204	0.0012756	0.0457951	5.692316667	5.698397368	5.732252941	5.790959091					
PSCD2	ILMN_1251874	0.3499468	0.4129054	0.0056646	0.7717056	0.0009164	0.0001789	6.155033333	6.126615789	6.134001471	6.253527273					
control_ILMN_137	ILMN_1378911	0.3266769	0.4247131	0.0042881	0.8607028	0.0134591	0.0075772	5.681283333	5.703731579	5.700030882	5.779386364					
F830116E18RIK	ILMN_2905525	0.4896761	0.3190837	0.0167845	0.9051036	0.009933	0.0007604	5.922473333	5.896713158	5.892973529	6.020572727					
ATPBD3	ILMN_2783557	0.7558553	0.8869908	0.0168345	0.8187708	0.0274611	0.0072203	6.565073333	6.575857895	6.569377941	6.663122727					
SLC22A21	ILMN_1234533	0.1702699	0.8254046	0.0092515	0.0945147	0.0370609	0.0005133	5.769	5.808589474	5.774551471	5.867009091					
FBXL15	ILMN_2792047	0.4234882	0.5212452	0.0145001	0.8581854	0.0008434	0.0015336	7.304583333	7.278686842	7.283870588	7.402495455					
4930481A15RIK	ILMN_1248737	0.6128947	0.6812972	0.0065184	0.8872365	0.0042368	0.0033059	5.86779	5.882173684	5.878910294	5.965627273					
E030016N13RIK	ILMN_1235317	0.7352544	0.3676594	0.0179538	0.5831603	0.0100781	0.001144	6.23018	6.217034211	6.198725	6.328004545					
PDGFB	ILMN_2618714	0.9502168	0.6373694	0.0146617	0.6615403	0.0091513	0.0067522	6.229823333	6.231968421	6.243358824	6.327645455					
4933437N03RIK	ILMN_2774580	0.5990406	0.4084001	0.0399537	0.7236668	0.0224403	0.0412527	6.072506667	6.089789474	6.098586765	6.169759091					
2310046A06RIK	ILMN_2700344	0.0969343	0.3378204	0.0036116	0.4052594	9.837E-06	2.643E-05	5.654603333	5.617594737	5.633589706	5.7515					
MAK10	ILMN_2828599	0.5752463	0.9983123	0.0046274	0.4325795	3.775E-05	8.271E-05	9.515693333	9.500657895	9.515651471	9.612581818					
LOC277236	ILMN_2530354	0.7457257	0.6674914	0.0050773	0.9777173	0.0168366	0.0030544	5.9668	5.976176316	5.976830882	6.063481818					
TMEM43	ILMN_2700505	0.0835943	0.0626718	0.0036365	0.7250175	0.049367	0.0089064	9.091776667	9.138781579	9.132544118	9.1884					
CAMK1	ILMN_2671721	0.4436506	0.5545176	0.0132409	0.7570204	0.0275119	0.0108723	5.496296667	5.521871053	5.513919118	5.592581818					
SPIN2	ILMN_2934522	0.6340554	0.9950055	0.0251077	0.5423285	0.0292125	0.0044587	6.355673333	6.370976316	6.355486765	6.451927273					
V1RC23	ILMN_3004978	0.8737593	0.741798	0.0106524	0.86417	0.0198366	0.018027	5.68207	5.686805263	5.691661765	5.777972727					
ZFP239	ILMN_3127381	0.581699	0.6616131	0.0039776	0.2408011	0.0001224	0.0024932	7.216716667	7.201723684	7.227942647	7.312536364					
COQ6	ILMN_2766720	0.0609297	0.1767637	0.0005158	0.5643333	0.0293836	0.0150091	9.932686667	9.977407895	9.965157353	10.02839545					
CLCNKB	ILMN_2757283	0.5241885	0.7711142	0.0094468	0.329601	0.0020966	0.0219642	5.946433333	5.926278947	5.955783824	6.042031818					
2310051M13RIK	ILMN_1237270	0.9665468	0.7712352	0.010363	0.7144495	0.0083616	0.0005952	5.616203333	5.617539474	5.608520588	5.7118					
RHOT1	ILMN_1242500	0.0880116	0.115869	0.0021147	0.7432917	0.0370818	0.0149865	9.741433333	9.783013158	9.776666176	9.836890909					
GPR106, LGR8, GP	ILMN_1232852	0.6559832	0.1943148	0.0048859	0.3798783	0.0075116	0.018548	5.783516667	5.794997368	5.813719118	5.878945455					
OLFR907	ILMN_2598615	0.808605	0.1558679	0.0060448	0.074934	0.0033194	0.0356122	5.608323333	5.601023684	5.643789706	5.703095455					
TRABD	ILMN_2978533	0.9932761	0.6202648	0.0298695	0.5756887	0.0186853	0.0070355	8.528336667	8.528678947	8.508880882	8.623013636					
BLCAP	ILMN_2705802	0.2000735	0.9166434	0.0072842	0.0669285	4.606E-05	0.0006072	8.161383333	8.124126316	8.163920588	8.255986364					
GM628	ILMN_3160644	0.5486986	0.373997	0.0198613	0.8226972	0.0470765	0.0434019	5.719166667	5.740194737	5.746419118	5.813490909					
DNM3	ILMN_2482131	0.257664	0.2129101	0.0029941	0.9502021	0.0252594	0.0271116	5.678993333	5.708965789	5.710380882	5.771918182					
MCPT6	ILMN_3161036	0.3075769	0.3338097	0.0163978	0.9128994	0.0019187	0.0012382	5.812683333	5.779026316	5.782316176	5.905395455					
MAPKAPK2	ILMN_2614212	0.3405698	0.9789861	0.0144365	0.2270708	0.0002685	0.0036369	10.01880333	9.9891	10.01956471	10.11131818					
FAM123A	ILMN_2456075	0.40995	0.9842072	0.0055417	0.3393686	0.0161379	0.0011625	5.86123	5.882305263	5.861704412	5.953740909					
SAMD10	ILMN_2656359	0.1227068	0.7510529	0.0333693	0.0739749	0.0008446	0.0023264	6.993723333	6.932505263	6.984454412	7.085995455					
ZFP748	ILMN_3083372	0.9505811	0.723013	0.0152283	0.7528248	0.011281	0.0166564	5.80429	5.806384211	5.815305882	5.896513636					

MLANA	ILMN_2624504	0.8366064	0.9717368	0.0342591	0.7724045	0.0094546	0.0116815	5.76753	5.760731579	5.768636765	5.8595					
ITGA2B	ILMN_2911123	0.5523529	0.8240584	0.0280837	0.6767155	0.0063309	0.0116275	6.458176667	6.438010526	6.450829412	6.550131818					
CYGB	ILMN_2801891	0.7970836	0.5797241	0.0284966	0.3738972	0.0169884	0.0497784	6.101066667	6.091292105	6.119488235	6.193004545					
ATP13A1	ILMN_1250382	0.504888	0.5304265	0.0137751	0.956733	0.0278898	0.0261892	8.999603333	9.019186842	9.017792647	9.091486364					
TRIM2	ILMN_1234848	0.8635114	0.7258571	0.0480353	0.5690486	0.0364928	0.0284386	5.767873333	5.761813158	5.777947059	5.859163636					
A230084A06RIK	ILMN_1230144	0.438214	0.7908668	0.017746	0.5822307	0.0008696	0.0054444	5.812173333	5.788897368	5.803954412	5.903431818					
CXCL14	ILMN_2659426	0.3672578	0.6984898	0.0227136	0.5363132	0.0014648	0.0040045	5.63365	5.604478947	5.621769118	5.724809091					
TTC15	ILMN_2502614	0.9478116	0.2793105	0.0146119	0.2251028	0.0204812	0.0003816	7.225596667	7.227939474	7.191466176	7.316681818					
TG737RPW	ILMN_2772847	0.769335	0.5408913	0.0125609	0.3251282	0.0054868	0.032187	6.282606667	6.273515789	6.300575	6.372831818					
AMBRA1	ILMN_3048294	0.4436924	0.3474318	0.0044493	0.897776	0.0232608	0.0215698	5.725223333	5.745826316	5.748851471	5.815154545					
control_ILMN_137	ILMN_1378677	0.9834438	0.4208402	0.0119587	0.3634062	0.0064359	0.0105893	5.97326	5.972618421	5.993672059	6.063127273					
PEF1	ILMN_2643723	0.3190562	0.349924	0.0023136	0.9026663	0.0456968	0.0324455	9.281666667	9.310060526	9.306907353	9.371368182					
D0HXS9928E	ILMN_2856628	0.9868439	0.2430322	0.0119803	0.1947226	0.0068983	0.0411307	9.81716	9.816639474	9.848302941	9.90685					
9/2/13	ILMN_2756696	0.4036944	0.7067578	0.0062425	0.6412731	0.032492	0.0116011	5.95884	5.979671053	5.968436765	6.048472727					
OLFR519	ILMN_2854265	0.6487248	0.8306964	0.0441492	0.4397773	0.0088403	0.0439192	5.661136667	5.643110526	5.669105882	5.750636364					
OLFR312	ILMN_3161363	0.3709362	0.9122832	0.0341618	0.2388347	0.0006155	0.0205264	5.722203333	5.691555263	5.725964706	5.811631818					
AMBRA1	ILMN_3124160	0.1300997	0.3534726	0.0171014	0.387355	2.983E-05	0.0002112	8.782346667	8.732421053	8.754764706	8.87175					
COL4A1	ILMN_2621643	0.4260512	0.741024	0.0226211	0.5854495	0.000579	0.0037227	6.219506667	6.194963158	6.209345588	6.308686364					
AI844366	ILMN_2684007	0.8267983	0.7540866	0.0158645	0.9546165	0.0021707	0.0003333	5.80145	5.794457895	5.793163235	5.890395455					
CAR6	ILMN_1257323	0.6178161	0.3420742	0.0128943	0.0987578	0.0011396	0.0434174	5.65408	5.63995	5.680355882	5.743022727					
PLD1	ILMN_2595408	0.6933796	0.4598863	0.0261827	0.1848692	0.0031361	0.0417093	5.90579	5.893855263	5.927305882	5.994177273					
C230092H20RIK	ILMN_1233960	0.6635724	0.8478167	0.0125377	0.4360673	0.0039406	0.0017189	5.8978	5.885568421	5.901997059	5.986186364					
GSTK1	ILMN_2792924	0.4032401	0.7012269	0.0255146	0.5506278	0.0012396	0.0033184	8.39841	8.369881579	8.386477941	8.48635					
EPM2A	ILMN_2920454	0.8794744	0.8061546	0.0132493	0.959312	0.0124013	0.0011846	5.76621	5.761413158	5.760173529	5.853804545					
LOC383439	ILMN_2539543	0.2952126	0.9774352	0.0025687	0.1846293	0.0001346	0.0003456	5.70715	5.681865789	5.707725	5.794672727					
C730048C13RIK	ILMN_2685022	0.2852307	0.2000217	0.0027292	0.9797444	0.036687	0.0173485	5.911796667	5.940139474	5.940685294	5.9992					
2210010L05RIK	ILMN_2599306	0.7962894	0.7235455	0.0103897	0.9415268	0.0088344	0.0059479	5.589083333	5.596602632	5.598317647	5.676368182					
NAPA	ILMN_2865822	0.765486	0.7354396	0.0219155	0.9886115	0.0101871	0.0021767	8.002653333	7.993915789	7.994260294	8.089840909					
GABRG1	ILMN_1245521	0.8166826	0.7082468	0.0148667	0.8735121	0.0120432	0.0154195	5.539946667	5.546115789	5.549933824	5.626995455					
4930471I01RIK	ILMN_1249396	0.2839252	0.5667521	0.0059659	0.5439773	0.0084971	0.0042064	5.840526667	5.865676316	5.854005882	5.927468182					
COBLL1	ILMN_2782634	0.9710692	0.2118397	0.0255754	0.1196364	0.0050579	4.622E-05	7.115376667	7.116502632	7.080435294	7.202304545					
CNTLN	ILMN_1240584	0.3068149	0.1586001	0.0077427	0.7782586	0.0372153	0.0339742	5.61508	5.641421053	5.647154412	5.701913636					
TSNAXIP1	ILMN_2881498	0.9924676	0.90236	0.0071163	0.9143399	0.0261038	0.0062068	5.814843333	5.814523684	5.811405882	5.901509091					
A4GALT	ILMN_3009373	0.8116153	0.5693332	0.0184883	0.736504	0.021082	0.0257947	5.785286667	5.792531579	5.801111765	5.871859091					
CHGA	ILMN_2814927	0.3483913	0.7266219	0.0096339	0.1381136	0.0005824	0.0091828	5.746556667	5.719539474	5.755676471	5.832945455					
SOX7	ILMN_1234505	0.6093964	0.3749197	0.021001	0.097519	0.0254778	0.0002976	6.187313333	6.202473684	6.163425	6.273668182					
KIF17	ILMN_2704842	0.3924928	0.7016444	0.0148383	0.5102019	0.0442288	0.0077901	5.8875	5.912607895	5.897030882	5.973745455					
CDCA1	ILMN_2755241	0.9154914	0.5353693	0.0386027	0.3891859	0.0087424	0.0495258	5.64207	5.638189474	5.662566176	5.727986364					
TCF7L2	ILMN_1231177	0.6368615	0.7232551	0.0093115	0.7890029	0.0050373	0.0004892	5.724723333	5.710573684	5.716716176	5.810431818					
OLFR506	ILMN_2813771	0.2185635	0.1278011	0.0257981	0.934357	0.0002361	2.424E-05	5.67991	5.637868421	5.635769118	5.765595455					
WNK2	ILMN_1214402	0.9320074	0.6358419	0.0308033	0.662214	0.0141372	0.0324535	5.703856667	5.706834211	5.718935294	5.789340909					
8030455F02RIK	ILMN_2551098	0.5985382	0.1917938	0.0111058	0.4126604	0.0153009	0.0482003	5.73374	5.748163158	5.765951471	5.819077273					
D430013G08RIK	ILMN_1241865	0.4394908	0.7898042	0.0137003	0.5944586	0.0174799	0.0124872	5.650593333	5.670342105	5.657922059	5.735872727					
NRXN1	ILMN_2550623	0.4470251	0.6243021	0.0095758	0.7062275	0.0001669	0.0003535	5.979573333	5.959471053	5.967466176	6.064813636					
WIZ	ILMN_3095005	0.3430532	0.7241176	0.0455579	0.3711582	0.0016648	0.0030671	7.21713	7.182071053	7.206314706	7.301809091					
BPHL	ILMN_2747284	0.7341613	0.6190805	0.0071592	0.3685003	0.0121313	0.0012581	5.726646667	5.735092105	5.714341176	5.811322727					
EG665903	ILMN_2624523	0.867418	0.9975489	0.0130084	0.8205651	0.0023711	0.0012322	5.54671	5.541965789	5.546788235	5.631263636					
GPSN2	ILMN_2606579	0.6369265	0.6787878	0.0467409	0.2847033	0.0127211	0.0376058	11.36477667	11.34527632	11.37852794	11.44931364					
V1RG5	ILMN_2838831	0.824207	0.9841046	0.013051	0.8081059	0.0152872	0.0055188	5.585696667	5.592194737	5.586235294	5.669590909					
LOC668779	ILMN_1248416	0.6353751	0.7961937	0.0193804	0.3512141	0.008817	0.0011392	5.82907	5.841984211	5.822419118	5.91275					
AW538212	ILMN_2688996	0.1153576	0.0942852	0.004006	0.992924	0.0001064	3.481E-05	5.662	5.618852632	5.619076471	5.745609091					
D930029H24RIK	ILMN_1228871	0.4741864	0.4712487	0.0066788	0.986283	0.001112	0.000874	5.586836667	5.5675	5.567926471	5.670409091					
control_ILMN_137	ILMN_1379309	0.7754473	0.6056504	0.0167735	0.8189121	0.0339137	0.0412178	5.740433333	5.749018421	5.755252941	5.823736364					
MFSD7C	ILMN_1248190	0.921069	0.2732591	0.0172727	0.216696	0.0190112	0.0492927	5.92774	5.924468421	5.955697059	6.011031818					
EFNA5	ILMN_2662536	0.3652588	0.5570784	0.0099712	0.1089818	0.0016573	0.0402057	5.71599	5.688497368	5.732614706	5.799154545					
DMXL1	ILMN_2429071	0.549142	0.9045195	0.0143862	0.5220237	0.0117717	0.0018762	5.96065	5.975723684	5.963417647	6.043718182					
LOC276803	ILMN_2534316	0.1660366	0.5484472	0.0219723	0.2751194	0.0007734	0.00151	5.940693333	5.897260526	5.924936765	6.02375					
2810043G13RIK	ILMN_2705440	0.8139185	0.2479328	0.0098315	0.326601	0.0071206	0.0171305	5.881486667	5.887989474	5.907816176	5.964436364					
ABCF3	ILMN_2677696	0.0807858	0.6364522	0.0019293	0.1713271	3.673E-05	0.0011514	9.548456667	9.504076316	9.536877941	9.6311					
9830166F08RIK	ILMN_2586977	0.7065803	0.8033313	0.037432	0.8797518	0.0111999	0.0167932	5.70739	5.718118421	5.714625	5.789945455					

PDE4B	ILMN_2574417	0.8976379	0.7676746	0.0064413	0.6259631	0.0012214	0.0039113	5.75419	5.751107895	5.761052941	5.836522727					
GM378	ILMN_1240049	0.6146732	0.4216927	0.0243609	0.8252222	0.0372352	0.0202	5.619333333	5.633768421	5.638488235	5.701636364					
B230378H13RIK	ILMN_1237858	0.9091221	0.3964068	0.0097891	0.2745947	0.0053004	0.0001191	5.648293333	5.651236842	5.628592647	5.730445455					
9530031H08RIK	ILMN_1246046	0.6151298	0.5241143	0.0132179	0.2096074	0.0043847	0.0182887	5.732286667	5.718855263	5.747191176	5.814431818					
4930433I11RIK	ILMN_2599632	0.8000884	0.6807661	0.0319361	0.4476331	0.0381796	0.0032443	5.615196667	5.622955263	5.604207353	5.697304545					
CEBPE	ILMN_1220301	0.8800194	0.9260761	0.0478902	0.9290989	0.0133926	0.0093032	6.23217	6.227065789	6.229376471	6.314113636					
MTA2	ILMN_2595774	0.8956106	0.6229647	0.0280136	0.6619534	0.0018644	0.0022656	9.862493333	9.858439474	9.847454412	9.943504545					
APOC1	ILMN_1228469	0.3661349	0.6711828	0.0058392	0.1642897	0.0015973	0.0193149	5.5881	5.564128947	5.598686765	5.669086364					
NLRP5	ILMN_2696556	0.3619698	0.7254515	0.0207835	0.4886605	0.0048973	0.006104	5.79998	5.772057895	5.790597059	5.880922727					
CYP2D26	ILMN_2642975	0.7949386	0.7066553	0.0070704	0.4978489	0.0054872	0.0160965	5.73149	5.724460526	5.741060294	5.812322727					
control_ILMN_137	ILMN_1378609	0.9854576	0.6965119	0.0164474	0.6483975	0.0078257	0.0114048	5.733256667	5.732771053	5.742875	5.814027273					
OLFR524	ILMN_3160809	0.3391258	0.9105733	0.0146557	0.2807778	0.0023346	0.0052156	5.86197	5.829492105	5.858877941	5.942722727					
LOC384689	ILMN_1227050	0.5628835	0.9166554	0.0099089	0.4908073	0.0008798	0.0002903	5.639723333	5.6252	5.637626471	5.72035					
ADAT1	ILMN_2977193	0.7744021	0.7923739	0.0303593	0.5287289	0.0485827	0.0066179	6.339843333	6.349155263	6.332338235	6.420336364					
CCDC15	ILMN_1250884	0.6209673	0.7809347	0.0239144	0.3680485	0.0038831	0.0153203	5.833546667	5.819647368	5.840797059	5.913731818					
3/8/13	ILMN_1242531	0.6393676	0.5873104	0.0472334	0.252753	0.0454452	0.0075776	5.87245	5.886128947	5.855538235	5.952545455					
C230094L10RIK	ILMN_1230165	0.9574539	0.9788968	0.0150405	0.9730634	0.000603	0.0028253	5.776726667	5.778110526	5.777417647	5.856627273					
E130113E03RIK	ILMN_1235043	0.7063443	0.5129698	0.0198069	0.8524375	0.044119	0.0185723	5.561873333	5.574134211	5.578641176	5.641704545					
C730029N23RIK	ILMN_1255622	0.6494385	0.8090042	0.0080432	0.4129841	0.000774	0.0062236	5.84567	5.8332	5.851813235	5.925118182					
3/6/13	ILMN_1242572	0.9942194	0.3218922	0.0083005	0.2640059	0.0034265	0.0001178	7.65466	7.654852632	7.630813235	7.734104545					
LOC384155	ILMN_2537186	0.9405523	0.8907799	0.0405563	0.9417188	0.0217712	0.0246912	5.67926	5.681310526	5.683101471	5.758663636					
MTMR6	ILMN_2687053	0.2368739	0.2300567	0.0267162	0.8271908	0.0008638	0.0012294	5.577	5.549068421	5.543748529	5.656304545					
9790357_3511_RC	ILMN_2468115	0.5056116	0.5587752	0.0312021	0.1650359	0.0074549	0.0404245	5.689263333	5.670352632	5.704086765	5.768504545					
OLFR11	ILMN_2619903	0.5196682	0.949838	0.0157061	0.4415391	0.0363056	0.0026979	5.660956667	5.678615789	5.662433824	5.740054545					
2510025K24RIK	ILMN_1235980	0.1703446	0.2143821	0.0022974	0.8388626	0.0498275	0.0358644	5.582343333	5.614260526	5.610119118	5.661422727					
SLC7A14	ILMN_2679229	0.4024276	0.9621712	0.0324826	0.3801255	0.0048549	0.0226588	5.839583333	5.813355263	5.838160294	5.918436364					
5830448L21RIK	ILMN_1254606	0.303375	0.1374351	0.0141593	0.8176851	0.0004035	7.262E-06	5.74232	5.713471053	5.708772059	5.821154545					
4631434O19RIK	ILMN_1251169	0.8285775	0.4492532	0.0375851	0.2566836	0.0121536	0.0331252	6.00784	6.001673684	6.026148529	6.086531818					
C230011P08RIK	ILMN_1258653	0.7136662	0.5163122	0.024265	0.2799267	0.0156805	0.032123	5.752406667	5.742231579	5.767427941	5.831013636					
OLFR483	ILMN_2590828	0.4492508	0.8062285	0.0449422	0.2432488	0.0073063	0.0282144	5.625586667	5.602889474	5.632177941	5.704077273					
LOC382853	ILMN_2536029	0.7787541	0.9684398	0.0097594	0.7519858	0.0216359	0.0027299	5.963353333	5.971465789	5.964307353	6.041727273					
SETD6	ILMN_1223358	0.5720042	0.747874	0.0161152	0.6603476	0.0049421	0.0008943	8.83772	8.819034211	8.829725	8.916090909					
CBFA2T2	ILMN_2752204	0.5662482	0.8989732	0.0322854	0.3878596	0.0047507	0.0130204	6.07727	6.059334211	6.080733824	6.1556					
LOC100045448	ILMN_2776734	0.6213216	0.8787078	0.0212333	0.4037411	0.002945	0.0031453	5.673983333	5.660994737	5.677317647	5.752286364					
OLFR535	ILMN_2535079	0.3286244	0.1620139	0.0077861	0.8088199	0.0355564	0.0223754	5.571573333	5.596031579	5.600444118	5.649868182					
ATP2C1	ILMN_1252963	0.4909422	0.6596181	0.0486347	0.1701837	0.0033312	0.0431696	6.559026667	6.536521053	6.572148529	6.637072727					
KLHDC8B	ILMN_1216669	0.9704039	0.7411117	0.0232253	0.7020326	0.0283416	0.0293612	5.822313333	5.821168421	5.831194118	5.899663636					
ADAMDEC1	ILMN_1246807	0.9459073	0.9293539	0.0264493	0.9837397	0.0143685	0.0098232	5.72808	5.726289474	5.725811765	5.80525					
UNK	ILMN_2509928	0.1747852	0.7541904	0.0387304	0.0508536	0.001094	0.0333703	8.188016667	8.146186842	8.196697059	8.2651					
5730460C07RIK	ILMN_1249562	0.87299	0.3660751	0.0246919	0.2604235	0.0362752	0.0006832	6.136043333	6.140568421	6.114717647	6.213077273					
KLHL26	ILMN_2647313	0.9727738	0.7196289	0.0261983	0.7548075	0.0390019	0.0029538	9.295173333	9.293997368	9.285769118	9.371863636					
LIG3	ILMN_2828819	0.7359932	0.6755234	0.0459519	0.9248678	0.0127639	0.0118872	8.005243333	7.995089474	7.992510294	8.081459091					
PAM	ILMN_2626294	0.8689483	0.5171595	0.0253536	0.6355637	0.0316773	0.0276871	5.65448	5.659834211	5.671320588	5.730622727					
V2R15	ILMN_1242397	0.4121998	0.1147882	0.0436721	0.4236069	0.0042818	0.0002785	5.637346667	5.614844737	5.596172059	5.713459091					
6430605C03RIK	ILMN_2733534	0.8540093	0.8809848	0.0194935	0.6707513	0.0106462	0.0010346	5.550003333	5.554918421	5.54665	5.625927273					
8430429K09RIK	ILMN_2441482	0.5533616	0.5810811	0.0359835	0.1670382	0.0018934	0.0439161	6.45922	6.441084211	6.474969118	6.535031818					
C430004E15RIK	ILMN_2625047	0.3697792	0.9825949	0.0179686	0.2636391	0.0045869	0.0060148	8.70302	8.674578947	8.702492647	8.778827273					
LOC244335	ILMN_1238450	0.7967468	0.3306504	0.020946	0.4910858	0.0309612	0.0329997	5.711056667	5.718365789	5.732983824	5.786463636					
DOXL2	ILMN_2987657	0.2640558	0.0917789	0.0379656	0.6544394	0.0026876	0.0002439	5.788483333	5.74895	5.735679412	5.863831818					
4930554K12RIK	ILMN_2549226	0.9004539	0.710907	0.0442598	0.7992425	0.0372571	0.0394321	5.8731	5.877186842	5.883825	5.948418182					
TGFBR2	ILMN_1215171	0.8079876	0.4435111	0.037859	0.6033996	0.0126369	0.0007095	6.004693333	5.996826316	5.984408824	6.079877273					
HPX	ILMN_2830333	0.6694536	0.4208039	0.0381786	0.805516	0.0243485	0.0017441	5.89602	5.880847368	5.874075	5.971186364					
2900052P03RIK	ILMN_1240268	0.4306129	0.7911564	0.0187692	0.5697159	0.0369631	0.0172313	6.166873333	6.185847368	6.173483824	6.241986364					
CPXCR1	ILMN_2846457	0.8637141	0.9550009	0.0316079	0.7726182	0.0124467	0.0059329	5.67251	5.677502632	5.671010294	5.747422727					
TAS2R106	ILMN_2702928	0.9187074	0.6724644	0.0153849	0.58289	0.0169758	0.0250608	5.694626667	5.691915789	5.704780882	5.769118182					
4632404B13RIK	ILMN_1217530	0.8107201	0.8371732	0.0395081	0.9662793	0.0466484	0.0411893	5.94389	5.950968421	5.949844118	6.018218182					
1600012K10RIK	ILMN_1254674	0.9523282	0.6085668	0.0069853	0.5398244	0.0086333	0.0005093	5.759546667	5.761076316	5.748283824	5.833790909					
OLFR121	ILMN_2979343	0.8832505	0.847904	0.0323481	0.9536961	0.0323323	0.0387364	5.667883333	5.671623684	5.673066176	5.741809091					
6130401J04RIK	ILMN_2604105	0.6675924	0.6066087	0.0125862	0.9803695	0.0463431	0.0125091	5.64243	5.654536842	5.653991176	5.716331818					
CLPTM1L	ILMN_2681221	0.8649116	0.7220966	0.0250452	0.8409751	0.0142349	0.0231143	11.29636667	11.30115	11.30577794	11.36985					

4732490P12	ILMN_2453810	0.7377437	0.5585768	0.0133897	0.3061863	0.0054102	0.0337014	5.554813333	5.5452	5.569813235	5.628077273					
LOC545289	ILMN_1260380	0.941619	0.6075387	0.022309	0.5177888	0.0132134	0.0491619	6.18028	6.178142105	6.194416176	6.253086364					
ATP6V0D1	ILMN_2928320	0.6900076	0.728914	0.0443055	0.3806752	0.0151146	0.0235403	10.56093333	10.54983158	10.56906471	10.63357727					
EG433144	ILMN_2755355	0.8756617	0.8096957	0.0443824	0.9559132	0.0418823	0.0156166	5.7232	5.727760526	5.728964706	5.795736364					
RAB11FIP5	ILMN_3139693	0.9990641	0.2126248	0.0441351	0.1797303	0.0351911	0.0007083	6.24462	6.244602632	6.211269118	6.316795455					
OLFR94	ILMN_3160479	0.9760098	0.4830532	0.0374765	0.4333955	0.0459114	0.0112953	5.866656667	5.867705263	5.842164706	5.938768182					
LOC245453	ILMN_1258169	0.9814367	0.5976572	0.0442265	0.5396395	0.026517	0.0174204	5.83364	5.832957895	5.845691176	5.905668182					
V1RD7	ILMN_2446884	0.9909554	0.8056549	0.018954	0.775485	0.0113766	0.003005	5.525103333	5.525413158	5.519311765	5.597113636					
ANKRD35	ILMN_1248776	0.7017476	0.1546409	0.0378699	0.2822763	0.0157341	0.0006009	5.805983333	5.794584211	5.766820588	5.877945455					
SETD6	ILMN_2983669	0.3325257	0.8331455	0.0479502	0.2962771	0.0003693	0.005126	8.39426	8.365178947	8.388533824	8.466163636					
SQLE	ILMN_2573600	0.9365985	0.8853935	0.0325657	0.8004337	0.0164647	0.0242576	5.744813333	5.742528947	5.748705882	5.816636364					
A330004N04RIK	ILMN_1217986	0.6714169	0.4101952	0.0274477	0.72872	0.0111155	0.001351	5.65038	5.638315789	5.630204412	5.722040909					
USP22	ILMN_2494618	0.4753104	0.218617	0.024228	0.6585392	0.0022193	0.0001606	8.500313333	8.479518421	8.469433824	8.571968182					
control_ILMN_137	ILMN_1378808	0.7918108	0.7914904	0.020449	0.9850515	0.0358714	0.0203257	5.613206667	5.619639474	5.619225	5.684513636					
AK122498	ILMN_2524245	0.8573697	0.941512	0.0237754	0.7723948	0.013784	0.0111714	5.750016667	5.754710526	5.748085294	5.821009091					
LOC381546	ILMN_1248921	0.7966801	0.7069903	0.0450686	0.4392662	0.0082922	0.0276737	5.912206667	5.904615789	5.922085294	5.983063636					
ATP7B	ILMN_2731102	0.6249321	0.3773685	0.0129853	0.7770016	0.0138625	0.0013353	5.68384	5.670181579	5.663311765	5.754631818					
RBM41	ILMN_2719569	0.934483	0.6404748	0.0416178	0.52394	0.0213442	0.0039933	5.543303333	5.5455	5.531645588	5.613786364					
0610007L01RIK	ILMN_1232042	0.8559407	0.4379169	0.0285673	0.2891227	0.0280934	0.002647	5.754013333	5.758968421	5.733432353	5.82365					
2610528H13RIK	ILMN_1212831	0.3930556	0.9599698	0.03971	0.3004828	0.0036936	0.0097935	5.603936667	5.579705263	5.602720588	5.673463636					
D030018H12RIK	ILMN_1215334	0.8440724	0.8154191	0.0318366	0.9802364	0.0425326	0.0373534	5.641683333	5.647418421	5.648041176	5.711190909					
CDH12	ILMN_1253923	0.5943313	0.7770197	0.0416686	0.3255477	0.0453507	0.0074321	5.66794	5.682739474	5.660370588	5.737386364					
E430014N10RIK	ILMN_1240823	0.3435904	0.6148169	0.0212175	0.5334167	0.0009554	0.0020686	5.662576667	5.636118421	5.650170588	5.731740909					
LRRC17	ILMN_2511456	0.9551469	0.8335749	0.0290847	0.7659924	0.0143345	0.0224821	5.906216667	5.904839474	5.911247059	5.975331818					
4931417G12RIK	ILMN_2710573	0.9128157	0.3890973	0.0242012	0.2864651	0.0213295	0.0009605	5.614283333	5.617	5.594630882	5.683309091					
OLFR615	ILMN_1231317	0.5436493	0.8015431	0.0273447	0.3266204	0.0145782	0.026873	5.721326667	5.703034211	5.727417647	5.790331818					
LOC631010	ILMN_1240227	0.9468688	0.2610293	0.0330551	0.262045	0.0237321	0.0007251	5.73648	5.73475	5.710307353	5.805286364					
PPP1CA	ILMN_2691613	0.0979511	0.5950614	0.0250473	0.206645	0.0001477	0.0023255	12.43433667	12.39944474	12.42297941	12.50304545					
E130009J12RIK	ILMN_2985193	0.4747768	0.8104448	0.0311078	0.6361709	0.0048768	0.026781	5.743916667	5.723594737	5.736714706	5.812536364					
CD209A	ILMN_2617210	0.2203565	0.4677138	0.0421166	0.5177875	0.0033906	0.0041365	5.65628	5.624876316	5.639332353	5.724654545					
TFPI	ILMN_2691522	0.471803	0.919021	0.034195	0.4349633	0.0085984	0.0156076	5.627863333	5.604692105	5.625113235	5.696140909					
5730530J16RIK	ILMN_1249981	0.5888761	0.6782263	0.0171141	0.8480426	0.0473222	0.0234928	5.65864	5.672378947	5.668261765	5.726768182					
EG666609	ILMN_2742647	0.4604068	0.4812998	0.0243882	0.9116043	0.0381232	0.0348145	13.11461333	13.13349737	13.13128971	13.18201818					
VPS13D	ILMN_1230695	0.9970315	0.9277966	0.0225157	0.9307211	0.028746	0.0073633	5.825393333	5.825310526	5.823529412	5.892627273					
BCL11A	ILMN_1234185	0.5462021	0.579227	0.0125649	0.923978	0.004601	0.0038714	5.64446	5.628544737	5.630797059	5.711472727					
PVRL2	ILMN_1218242	0.7716648	0.4925211	0.0328843	0.2548968	0.0100569	0.0391744	5.80492	5.796597368	5.821182353	5.87185					
GM392	ILMN_1240780	0.4822211	0.8134091	0.0216292	0.2876355	0.0008447	0.0175371	5.49587	5.480410526	5.501230882	5.562286364					
3110012M05RIK	ILMN_1219662	0.9736899	0.6487079	0.0378024	0.6487488	0.027784	0.0075749	5.661996667	5.661102632	5.650423529	5.728168182					
6330437I11RIK	ILMN_1228346	0.5405555	0.4208121	0.0385954	0.8446392	0.0104827	0.0056542	5.88513	5.868713158	5.863848529	5.950863636					
4921537F17RIK	ILMN_1247508	0.6199852	0.8688351	0.0413671	0.4606361	0.0087602	0.0345781	5.829483333	5.817065789	5.833544118	5.894563636					
MAK10	ILMN_2612403	0.1008528	0.7863311	0.0344079	0.09093	6.992E-05	0.0080898	8.8104	8.765121053	8.803444118	8.874963636					
FTS	ILMN_1219649	0.2509636	0.5051484	0.0263481	0.5904826	0.0037326	0.0064183	5.683446667	5.655231579	5.667736765	5.747927273					
PHXR5	ILMN_2669458	0.1790029	0.1746902	0.021961	0.9576737	0.0003671	0.0008675	5.872406667	5.83705	5.835738235	5.936231818					
genericLS	ILMN_2563949	0.1738504	0.2213433	0.0498602	0.7129671	0.0003577	0.0003645	6.13706	6.099334211	6.107116176	6.200122727					
DEFCR-RS1	ILMN_2524936	0.4313533	0.9437088	0.0406799	0.397069	0.0062888	0.0225731	5.621336667	5.599907895	5.619592647	5.683168182					
TSSK6	ILMN_2650022	0.7404492	0.8955475	0.0268206	0.8288781	0.0195886	0.028708	5.885473333	5.876747368	5.882098529	5.946995455					
LOC100044218	ILMN_1245604	0.6206067	0.9395966	0.0380872	0.619008	0.0037023	0.0173649	5.70159	5.688944737	5.699707353	5.762777273					
GULO	ILMN_2631948	0.0975262	0.9195211	0.0401329	0.0553495	0.0003251	0.0130822	5.675643333	5.633252632	5.673354412	5.736640909					
LOC381636	ILMN_1232330	0.4217793	0.2693052	0.0426346	0.7692322	0.0056184	0.0018109	5.679483333	5.660081579	5.653820588	5.740377273					
1810019D21RIK	ILMN_1227679	0.2805957	0.2048988	0.0414469	0.8986591	0.0049656	0.0017564	5.926816667	5.899373684	5.896448529	5.987313636					
LOC100039649	ILMN_1215436	0.9062475	0.9964174	0.0463069	0.890633	0.0384995	0.0383708	5.650293333	5.647105263	5.650401471	5.710781818					
2900097C17RIK	ILMN_1227592	0.5687382	0.422504	0.0341477	0.8328788	0.0067311	0.0035324	5.539346667	5.523955263	5.519045588	5.599572727					
SEC11A	ILMN_2652936	0.6501775	0.199283	0.0456572	0.3753999	0.0077768	0.0004197	11.49351	11.48228158	11.46473971	11.55365909					
BOC	ILMN_1227184	0.8337988	0.6393165	0.0461802	0.4270286	0.0116962	0.0437442	6.039113333	6.034384211	6.049230882	6.099004545					
EG224576	ILMN_1215517	0.1428426	0.3883635	0.0427359	0.5908423	0.0018102	0.0065772	5.743756667	5.712397368	5.723908824	5.802577273					
E030045A12RIK	ILMN_2572276	0.7533502	0.2556227	0.0398761	0.3980358	0.0393183	0.0063534	5.811073333	5.802289474	5.779011765	5.86955					
TCF7L2	ILMN_2704212	0.4065981	0.2024417	0.0385675	0.7023388	0.0071444	0.0012342	5.820176667	5.799102632	5.790647059	5.877909091					
3732412D22RIK	ILMN_1216840	0.8198465	0.7019254	0.032674	0.9152516	0.0393518	0.0100702	5.46195	5.456131579	5.453826471	5.518868182					
SCL0002508.1_73	ILMN_2460618	0.0730489	0.4033591	0.0350052	0.2434024	0.0006494	0.0033275	5.615476667	5.575942105	5.598632353	5.671372727					
D030002E05RIK	ILMN_2443894	0.9871681	0.3086396	0.0441244	0.2759631	0.0485315	0.0022505	5.522803333	5.523189474	5.500327941	5.577595455					

**DOWN IN generic ONLY**																
Gene	ID	Medium vs. Reference Standard pval	Medium vs. GA pval	Medium vs generic pval	Reference Standard vs GA pval	Reference Standard vs generic pval	GA vs. generic pval	AVG Medium	AVG Reference Standard	AVG GA	AVG generic					
SATB1	ILMN_1240428	0.0791153	0.3714491	1.835E-08	0.3208173	1.239E-06	3.352E-08	8.554353333	8.466363158	8.510626471	8.184818182					
ANKRD37	ILMN_2595857	0.8560334	0.1198731	5.06E-05	0.1157346	2.414E-06	1.235E-08	7.88428	7.8945	7.976713235	7.532354545					
PDK1	ILMN_1229318	0.052532	0.2345219	3.018E-07	0.3207414	7.394E-06	5.696E-08	7.99527	7.908131579	7.945248529	7.658463636					
A830055I09RIK	ILMN_1252200	0.116183	0.490765	1.653E-05	0.2728292	0.0018525	3.344E-05	9.037106667	8.940439474	8.998422059	8.727586364		Pattern Represented by Data:			
CD2	ILMN_2753697	0.270814	0.2043282	1.132E-07	0.9832463	7.864E-08	1.036E-10	10.80060333	10.76561316	10.76611912	10.51960455					
IFIT3	ILMN_2944666	0.4738633	0.8180193	4.572E-05	0.1879099	1.54E-09	2.845E-08	10.83471	10.87340526	10.82387647	10.55753636		x	x	x	
TMSB10	ILMN_3145331	0.2102965	0.9700824	5.927E-07	0.1104946	4.191E-06	1.586E-10	11.75164	11.70039474	11.75035	11.47584545					x
PELI1	ILMN_1239770	0.8649088	0.0987008	7.669E-08	0.1038477	7.97E-09	6.23E-08	8.998133333	8.992394737	8.946526471	8.738936364		Medium	Reference	GA	generic
LOC665425	ILMN_1213664	0.2329765	0.2512096	3.148E-05	0.7859726	0.0006117	2.187E-05	8.158676667	8.102307895	8.112635294	7.902613636					
5830468F06RIK	ILMN_1247281	0.3090019	0.1726043	6.043E-07	0.8702872	1.912E-05	7.593E-07	7.97065	7.928986842	7.923582353	7.718622727					
BCL11B	ILMN_2611022	0.6154167	0.1641067	0.0007887	0.3499901	4.703E-05	1.398E-07	10.96429667	10.98868158	11.02490882	10.71570909					
SAMHD1	ILMN_1232707	0.0581447	0.1358425	0.0001731	0.2106154	0.0002072	2.725E-07	10.10535667	10.01243684	10.04689412	9.859695455					
IGFBP4	ILMN_1258988	0.8247493	0.2971467	0.0024654	0.3158236	0.0002821	0.0017553	9.80274	9.791734211	9.751577941	9.567136364					
OTTMUSG0000000	ILMN_3123441	0.1107087	0.0854703	0.0020082	0.9832071	0.0466722	0.048594	8.43207	8.3223	8.323457353	8.198613636					
A530024C08RIK	ILMN_1243902	0.417339	0.2352186	5.851E-05	0.7744948	0.0014881	0.0008813	7.640853333	7.602289474	7.590460294	7.425509091					
D330001F19RIK	ILMN_1259917	0.5414338	0.2492855	2.541E-05	0.5960725	0.0001179	0.0003451	7.24253	7.214571053	7.194076471	7.031618182					
C230075M21RIK	ILMN_1215649	0.2004857	0.6675291	0.002003	0.3600934	8.767E-06	0.0004134	9.102993333	9.175807895	9.128044118	8.894036364					
TERF2IP	ILMN_2986458	0.1759167	0.1139061	0.0001531	0.9108214	0.0029932	0.0002527	9.187036667	9.121160526	9.125073529	8.978186364					
ARID5B	ILMN_3031781	0.4803951	0.6356029	0.0001525	0.199053	0.0001587	0.0003784	7.844363333	7.88215	7.823404412	7.636695455					
VPS13A	ILMN_2561533	0.1737487	0.0543399	8.773E-05	0.6359344	0.000694	0.0013197	7.34862	7.289765789	7.273944118	7.143786364					
PPM1B	ILMN_2729953	0.2153906	0.4019662	0.0040908	0.5770257	0.0140321	0.0050354	6.483146667	6.413578947	6.438429412	6.27875					
ZFP281	ILMN_1220893	0.1462227	0.1782067	7.192E-06	0.6818773	3.04E-05	8.117E-07	9.17416	9.124147368	9.134429412	8.976263636					
2610027C15RIK	ILMN_2890357	0.1739387	0.1051192	0.0001863	0.8704277	0.0003711	0.0004013	7.341113333	7.286360526	7.281451471	7.143454545					
BC031575	ILMN_2703242	0.051041	0.0844921	0.0006558	0.727889	0.0136067	0.0071818	6.823543333	6.739026316	6.751310294	6.626427273					
PELI1	ILMN_2627441	0.6999333	0.6283901	0.0001402	0.9488944	4.637E-06	1.129E-06	9.126876667	9.144215789	9.146523529	8.930227273					
RNF125	ILMN_2917338	0.7175175	0.2110159	0.0008181	0.3032209	0.0001785	0.0033944	6.7446	6.727952632	6.689019118	6.548077273					
1500005K14RIK	ILMN_1217606	0.1006607	0.2662728	0.0007852	0.3023917	0.0132617	0.0002423	6.298223333	6.218907895	6.253997059	6.102204545					
1110059E24RIK	ILMN_2846731	0.0550847	0.2941685	4.421E-06	0.1574077	0.000366	1.447E-07	10.46984	10.40856579	10.44254265	10.27383636					
GSN	ILMN_2605645	0.1187801	0.1097083	0.0034143	0.7165015	0.0173612	0.0033002	6.616233333	6.532494737	6.545022059	6.421909091					
ARL6IP5	ILMN_1241924	0.1994807	0.5099584	0.0003607	0.3943777	0.0014221	0.0001063	10.5927	10.53820789	10.56679853	10.39869545					
NOTCH1	ILMN_1241915	0.1836487	0.9476014	0.0007254	0.0762828	1.45E-06	3.334E-06	8.46399	8.526157895	8.466470588	8.270063636					
9130422G05RIK	ILMN_2633062	0.0735943	0.055311	0.0001049	0.8371191	0.0215943	0.0041584	8.094413333	8.017089474	8.024388235	7.903022727					
LOC333818	ILMN_1244672	0.1473867	0.1788265	0.0016716	0.8353485	0.0196396	0.0096587	6.493086667	6.426	6.434104412	6.302809091					
PTPRC	ILMN_1212836	0.6066218	0.2797467	0.0086558	0.5160165	0.0036739	0.0246544	7.315573333	7.284710526	7.254329412	7.125622727					
KMO	ILMN_1224155	0.19403	0.1377385	0.0001681	0.7560324	0.00118	0.0057941	7.087556667	7.038618421	7.02755	6.898859091					
WIPF1	ILMN_1257209	0.5186512	0.3859468	0.0003857	0.8411962	1.898E-05	2.197E-05	10.24916333	10.22402105	10.21850441	10.06097727					
FOXK1	ILMN_3059798	0.2214689	0.3593198	8.76E-05	0.6920932	0.0012885	0.0002807	7.912773333	7.866897368	7.879917647	7.726354545					
2310032M22RIK	ILMN_2550291	0.5900809	0.3728974	0.0002003	0.1046556	3.064E-05	0.0001211	7.250013333	7.273057895	7.21885	7.0662					
RPS25	ILMN_2887619	0.3462766	0.7768594	0.030345	0.1550161	0.0043889	0.0322641	11.92878333	12.00342368	11.90966324	11.74598636					
LOC665446	ILMN_1257496	0.1932919	0.1101307	0.005035	0.8101603	1.097E-05	1.852E-06	8.40817	8.473381579	8.482916176	8.2254					
LOC237512	ILMN_2533952	0.1018504	0.0664387	0.0003858	0.9941641	0.0042691	0.0014531	12.71109333	12.65093947	12.65114265	12.53201364					
LOC386068	ILMN_1214874	0.0814176	0.4623673	0.0002713	0.14381	0.022137	0.0001023	6.033483333	5.962060526	6.008877941	5.856231818					
ZFP429	ILMN_1254717	0.0733232	0.1467533	0.0001571	0.5701116	0.0121388	0.0015129	7.935246667	7.8677	7.885716176	7.758968182					
BCL2L11	ILMN_2590638	0.304465	0.4004953	0.0001761	0.6930002	0.0006019	4.816E-05	7.933673333	7.893531579	7.905207353	7.758081818					
CUGBP2	ILMN_2594575	0.8726752	0.1578596	0.006068	0.1445108	0.0011724	0.0217475	8.703653333	8.694839474	8.636048529	8.528254545					
SLFN1	ILMN_2663930	0.3929347	0.5121897	0.0017059	0.7094926	4.92E-05	8.355E-06	10.68426	10.72121053	10.70865294	10.50982727					
KLHDC1	ILMN_2649635	0.8418996	0.0776056	0.00156	0.0847928	0.0009887	0.0318545	7.695726667	7.686955263	7.622123529	7.521354545					
CRAMP1L	ILMN_1247318	0.2014354	0.2364492	0.0010129	0.9589302	0.0007811	0.0011266	8.978753333	8.936526316	8.937939706	8.805404545					
CTSE	ILMN_1228235	0.9991113	0.4106553	0.0007046	0.3611638	0.0002081	0.000184	9.17455	9.174597368	9.144792647	9.003390909					
CGGBP1	ILMN_2668387	0.5794682	0.8887484	8.642E-05	0.5460508	2.289E-05	1.676E-07	10.34921667	10.33062895	10.34524412	10.17924545					
A130075K23RIK	ILMN_1217850	0.8153718	0.3224503	0.0005107	0.1656484	3.419E-05	0.0030816	7.857343333	7.866847368	7.817598529	7.687945455					
CDKN1B	ILMN_2923960	0.1295077	0.1319564	2.689E-06	0.8975346	0.0004217	0.000107	5.826616667	5.781607895	5.784983824	5.658118182					

HLTF	ILMN_2604086	0.1043547	0.0745037	0.0008757	0.9698134	0.015693	0.0119087	6.093073333	6.029134211	6.027913235	5.924654545					
FNBP4	ILMN_1260410	0.8308135	0.2444176	0.0026371	0.3010981	0.00159	0.0094423	7.02603	7.015605263	6.975920588	6.858595455					
LOC623121	ILMN_2526938	0.1192934	0.3685915	0.0126513	0.3275128	6.554E-05	0.0001654	7.308336667	7.396323684	7.352626471	7.142136364					
3300001P08RIK	ILMN_2798797	0.2603699	0.0906179	0.0011878	0.7227731	0.0025688	0.0005881	10.70506	10.66184211	10.65289118	10.53989545					
LOC100048622	ILMN_2677056	0.8875675	0.6150394	0.0061871	0.4324741	0.000341	0.0026012	9.917543333	9.924692105	9.894410294	9.753277273					
C430005L07RIK	ILMN_1231989	0.058126	0.0629128	0.0006098	0.8300766	0.0137559	0.0072494	6.22569	6.156028947	6.162217647	6.062545455					
ZFP654	ILMN_2634157	0.0690439	0.4576866	7.378E-06	0.2158423	0.0004514	1.882E-05	7.83237	7.779128947	7.811010294	7.670627273					
E030040J04RIK	ILMN_2582659	0.6078898	0.9458919	0.0006524	0.5975726	1.549E-05	5.698E-05	6.016863333	5.998997368	6.014388235	5.85555					
ARL4C	ILMN_1232537	0.7411904	0.1262729	0.0022258	0.0511661	0.0052297	3.71E-06	8.98283	8.969447368	9.037364706	8.822018182					
ARID4A	ILMN_1249368	0.6347784	0.2253888	0.0129741	0.4512613	0.0144791	0.0290618	7.24665	7.221260526	7.191375	7.086068182					
LOC546629	ILMN_2514130	0.1422128	0.1149133	0.0001395	0.842507	0.0031759	0.0067875	6.201816667	6.155281579	6.149257353	6.041940909					
TDP1	ILMN_1222101	0.2047179	0.2160393	0.000427	0.7660188	0.009534	0.0015996	7.001043333	6.9467	6.956392647	6.841454545					
CUL1	ILMN_1226323	0.0934383	0.0689027	0.0020377	0.7614183	0.0417649	0.0066512	8.832946667	8.763292105	8.772133824	8.675431818					
D030041G07RIK	ILMN_1242969	0.1886471	0.0625491	0.0009933	0.6143277	0.0080014	0.01659	7.062546667	7.013210526	6.998004412	6.907159091					
E130014H08RIK	ILMN_2680388	0.0669437	0.0611037	6.369E-05	0.9737956	0.0118435	0.0114303	6.120063333	6.056794737	6.057810294	5.964904545					
TRAF5	ILMN_2440604	0.5245563	0.240113	0.0069051	0.5272163	0.002895	0.021429	6.81995	6.790965789	6.768398529	6.664904545					
PPP2R5C	ILMN_2742461	0.1137921	0.0822204	0.000379	0.9294784	0.0116573	0.003355	6.077013333	6.021755263	6.024205882	5.922486364					
MTF2	ILMN_2703913	0.2003631	0.2985865	0.0037587	0.69841	0.0338986	0.0131918	10.53759667	10.48152632	10.49541324	10.38590909					
WDR20B	ILMN_1235032	0.2378139	0.4785172	0.0001924	0.4562457	6.383E-05	6.788E-06	5.85008	5.813589474	5.830157353	5.698718182					
RNF125	ILMN_1232600	0.9821989	0.0966035	0.0004117	0.0954952	0.0011395	5.261E-07	7.012443333	7.011668421	7.063036765	6.861245455					
9330168G06RIK	ILMN_1246293	0.541902	0.4831022	0.0002415	0.9455771	0.001393	0.0014771	6.250186667	6.227921053	6.225692647	6.100836364					
LAMP2	ILMN_2577206	0.8191765	0.0539446	0.0008346	0.0790161	0.0013531	0.0184912	6.187146667	6.178439474	6.124277941	6.037936364					
JMJD1A	ILMN_3043245	0.3184575	0.6524928	0.0002081	0.0721281	0.0003896	5.234E-07	8.215583333	8.183889474	8.228458824	8.066877273					
ST8SIA4	ILMN_2784230	0.1349801	0.4757493	0.0010337	0.2606324	0.0282489	0.0007833	9.773773333	9.715015789	9.749726471	9.625213636					
LRBA	ILMN_2659960	0.1799484	0.133935	0.0017445	0.9397744	0.0020409	0.0009886	9.108856667	9.059176316	9.061001471	8.960695455					
TDE2	ILMN_2775381	0.9569371	0.1312657	0.0031592	0.0902878	0.0004224	0.023742	9.271143333	9.2688	9.211947059	9.123181818					
9/6/13	ILMN_2681293	0.6376283	0.2496409	0.013978	0.4764143	0.0147676	0.042388	7.035323333	7.010981579	6.982983824	6.888654545					
D830015B12RIK	ILMN_1234367	0.3056546	0.0722794	0.000227	0.5230609	0.0080097	0.0113831	6.263736667	6.227584211	6.208301471	6.117745455					
PARD6G	ILMN_1237631	0.6758842	0.678385	0.0008684	0.9647959	0.0005219	0.0001265	7.15756	7.144507895	7.145636765	7.011668182					
4933400A11RIK	ILMN_1250324	0.1935489	0.4946985	0.0004042	0.3406698	0.0187275	0.0002919	5.711286667	5.661910526	5.690280882	5.565481818					
2210016L21RIK	ILMN_1251123	0.0684124	0.16045	0.0003539	0.64567	0.0044914	0.0025111	5.983086667	5.929236842	5.940947059	5.837568182					
MDM1	ILMN_2754667	0.2519943	0.245198	0.0003565	0.8734112	0.0067575	0.0011209	5.990166667	5.953171053	5.957473529	5.8456					
CDC37L1	ILMN_1242724	0.432965	0.761838	0.0018235	0.5048792	0.0099697	0.0004164	6.163896667	6.133318421	6.153929412	6.019554545					
B230215E14RIK	ILMN_1229011	0.592917	0.1953042	0.0001056	0.4836819	0.0009696	0.0011241	6.061116667	6.044002632	6.024869118	5.917009091					
POM121	ILMN_1232596	0.4266327	0.8235093	0.0023101	0.2179086	5.406E-05	0.0005903	7.310896667	7.342360526	7.30295	7.167040909					
FOXN3	ILMN_2844097	0.263597	0.2044031	0.0046681	0.8933949	0.0175122	0.0032148	8.376153333	8.329744737	8.333529412	8.232445455					
A130021K16RIK	ILMN_1251811	0.8195515	0.4426985	8.698E-05	0.2763052	6.792E-05	1.399E-05	5.984683333	5.9924	5.964948529	5.84155					
DAG1	ILMN_2853957	0.4252381	0.0602344	0.0057935	0.2406018	0.0046716	0.021198	7.180286667	7.147947368	7.115838235	7.037163636					
BIRC6	ILMN_1249233	0.4536278	0.3353887	0.0003783	0.8309255	0.0019878	0.0030886	6.940266667	6.915202632	6.908766176	6.797263636					
4930529C04RIK	ILMN_2725583	0.0698677	0.1711347	0.0008444	0.3333943	0.0233341	0.0005803	6.085303333	6.024965789	6.048104412	5.942704545					
SLC16A6	ILMN_1258950	0.2276815	0.1853384	0.0017589	0.9140867	0.0359606	0.0474072	7.43912	7.390255263	7.386244118	7.296981818					
LRRC40	ILMN_2647476	0.4646448	0.0601038	0.0005519	0.2406174	0.0007111	0.0022764	6.164913333	6.141073684	6.113097059	6.023013636					
BC051070	ILMN_1231143	0.2116225	0.6464236	0.0013109	0.3151572	0.0076044	0.0004444	5.77394	5.731926316	5.759560294	5.632954545					
LOC100045804	ILMN_1234370	0.3382488	0.1790407	0.0002281	0.8746705	0.0040019	0.0007338	6.080636667	6.046034211	6.041739706	5.939822727					
HSH2D	ILMN_1239119	0.1471192	0.5429422	0.0124357	0.2283693	0.0322786	0.0041383	7.86715	7.806244737	7.842983824	7.726418182					
VPS26B	ILMN_2631192	0.4877351	0.9609904	0.034198	0.3065398	0.0480956	0.0011512	6.30113	6.266228947	6.299214706	6.160686364					
ST6GAL2	ILMN_2593002	0.9558336	0.6548532	0.0032893	0.6389678	0.00014	9.679E-06	5.81399	5.816073684	5.828707353	5.674245455					
TMPRSS11A	ILMN_2890069	0.2867067	0.0781389	0.0083851	0.5779337	0.0331219	0.0413402	5.94694	5.900107895	5.882957353	5.808109091					
RAB27A	ILMN_2614966	0.3536785	0.847606	4.128E-05	0.1417117	0.0003182	2.305E-08	9.618303333	9.593155263	9.622351471	9.480636364					
LOC674087	ILMN_1227317	0.7428081	0.5889824	0.0356504	0.8518412	0.0432128	0.0485043	8.063523333	8.043044737	8.033966176	7.926122727					
1600012H06RIK	ILMN_2718861	0.8735272	0.6882422	0.0058169	0.8010036	0.0030669	0.003194	7.32814	7.321276316	7.312647059	7.190854545					
4733401O04RIK	ILMN_1241389	0.0677838	0.3952881	0.0014767	0.2311688	0.0481461	0.0036164	6.47715	6.413797368	6.449102941	6.340095455					
OLFR504	ILMN_3161078	0.7901314	0.3313358	0.0005974	0.1655799	9.368E-05	0.0013106	5.77475	5.783405263	5.745994118	5.638240909					
ADRA1B	ILMN_2606415	0.0567563	0.2886452	0.0007337	0.1648395	0.04211	0.0004266	5.788323333	5.726486842	5.760369118	5.652722727					
RAB6B	ILMN_2761430	0.4774153	0.1361146	1.386E-05	0.4558055	0.0008766	0.0024321	5.805626667	5.7864	5.766961765	5.670081818					
HBB-B1	ILMN_2545963	0.4662621	0.3162032	0.0349087	0.8666362	0.0474617	0.0308178	13.32452667	13.28905789	13.28345588	13.18936818					
ARHGAP12	ILMN_1227256	0.4817401	0.3480794	0.0002106	0.8572983	0.0007674	0.0003831	5.6783	5.657913158	5.653547059	5.543509091					
TYKI	ILMN_1240864	0.7589136	0.2384231	0.0348989	0.0519785	0.0036441	0.0175894	7.718213333	7.733778947	7.673405882	7.583704545					
LOC100048616	ILMN_1241963	0.7162173	0.3954892	0.0060337	0.6395898	0.0057424	0.0043046	6.623486667	6.608734211	6.594576471	6.489290909					
2600011C06RIK	ILMN_1220104	0.868067	0.3631006	0.0049484	0.2111005	0.0004285	0.0132494	6.318883333	6.324634211	6.286382353	6.185263636					

BIRC6	ILMN_1246660	0.0672901	0.104734	0.0013114	0.7687882	0.0121322	0.0153815	6.316916667	6.256421053	6.264130882	6.184036364				
RGS19	ILMN_2752044	0.3386985	0.0513674	0.0017904	0.4615514	0.0384581	0.0478533	6.648676667	6.608102632	6.584333824	6.516268182				
C130064E22RIK	ILMN_1221892	0.273743	0.2907843	0.0008524	0.7721263	0.0288794	0.0045581	7.445486667	7.401763158	7.410882353	7.313245455				
ITK	ILMN_2613422	0.2426571	0.1995844	0.0033648	0.9026044	0.0001104	5.597E-05	10.31404333	10.35670789	10.36085	10.18205455				
LCP2	ILMN_1216191	0.922014	0.3769241	0.0037673	0.2893479	0.0026778	0.0129815	6.589333333	6.593478947	6.556810294	6.45785				
RBM18	ILMN_2978585	0.8463194	0.3389752	0.0343126	0.1444854	0.0040046	0.0240736	7.331313333	7.341076316	7.292019118	7.200204545				
ZAP70	ILMN_1223542	0.9044306	0.705428	0.0456215	0.540508	0.0105488	0.0188293	7.124003333	7.130486842	7.106404412	6.993336364				
control_ILMN_137	ILMN_1378612	0.9737192	0.4660604	0.0008993	0.4172436	0.0008174	0.0016872	5.722833333	5.724013158	5.700172059	5.592318182				
MAT2A	ILMN_1258415	0.9394328	0.9420671	0.0351956	0.8585218	0.0147359	0.0087062	8.35585	8.352134211	8.359186765	8.225568182				
2210018M11RIK	ILMN_2521686	0.4267156	0.2939019	0.0047618	0.9461691	0.0249755	0.0079197	6.638536667	6.604639474	6.602507353	6.508409091				
ASH1L	ILMN_1245687	0.9747923	0.1188567	0.0053809	0.0649297	0.0009757	0.0241636	5.996946667	5.998094737	5.946575	5.8675				
LOXL3	ILMN_2665272	0.0917949	0.3127426	0.0005144	0.2454262	0.0442597	0.0005322	5.826726667	5.771368421	5.800639706	5.697527273				
UTX	ILMN_2506528	0.478826	0.3668646	0.0141063	0.8451567	0.0035826	0.0094907	9.849873333	9.822334211	9.816958824	9.722554545				
A730082L10RIK	ILMN_1220032	0.4032959	0.0666118	0.0020628	0.3220654	0.0062779	0.0220739	6.309363333	6.281157895	6.255379412	6.182745455				
ITGA6	ILMN_1223670	0.4066823	0.4327231	0.0030597	0.9994881	0.0018074	0.0045386	6.613506667	6.588739474	6.588763235	6.48725				
control_ILMN_137	ILMN_1378679	0.7876358	0.0526071	0.0035316	0.0717594	0.0028401	0.033764	5.660046667	5.650981579	5.603908824	5.534195455				
4930449I04RIK	ILMN_1238088	0.2051735	0.2326247	3.072E-05	0.8975991	0.0010693	0.0008706	5.92482	5.89145	5.894451471	5.800354545				
TSC1	ILMN_2423383	0.671018	0.2294959	0.0045867	0.4010538	0.0045924	0.01817	6.022403333	6.007705263	5.983791176	5.898672727				
MSH3	ILMN_3068452	0.9862047	0.388591	0.0029183	0.3099282	0.0003842	0.0101609	6.317296667	6.317907895	6.287680882	6.193777273				
9330171B01RIK	ILMN_1234726	0.0550221	0.4614803	0.0013359	0.1264928	0.0450841	0.001052	5.83447	5.777323684	5.814472059	5.710968182				
UGT2A1	ILMN_2434333	0.7105059	0.721824	0.004318	0.4166797	0.0006941	0.0029961	5.621583333	5.633452632	5.610448529	5.498127273				
C130078D09RIK	ILMN_1244755	0.1278604	0.3250882	0.0031383	0.4791575	0.001815	0.0022079	6.053696552	6.004831579	6.019788235	5.930827273				
RHBDF2	ILMN_1221129	0.8418475	0.5974591	0.0208933	0.7956291	0.0490314	0.0162212	6.356373333	6.346123684	6.336120588	6.233854545				
INPP4B	ILMN_1226239	0.0845058	0.2023733	0.0305842	0.5569054	8.807E-05	0.00041	9.272156667	9.351981579	9.328983824	9.149936364				
NIPSNAP1	ILMN_2619861	0.5290402	0.461368	0.0011652	0.113783	7.689E-05	0.0007688	9.28918	9.308102632	9.269794118	9.167031818				
LPIN2	ILMN_1220680	0.8290993	0.4643068	0.0118867	0.6218323	0.0180038	0.0137632	7.60717	7.597921053	7.581547059	7.485290909				
4833420G17RIK	ILMN_1242705	0.1105861	0.9985108	0.0014004	0.0558918	0.0298299	0.000163	6.1815	6.133973684	6.181452941	6.059827273				
NT5C	ILMN_1223097	0.9686998	0.1511051	0.0050398	0.0966739	0.0004879	0.0482836	10.14017667	10.13889474	10.09159412	10.0186				
D730033A03RIK	ILMN_1237794	0.1053282	0.0793496	0.0011131	0.9966108	0.0192989	0.0159084	5.94283	5.894176316	5.894086765	5.821409091				
1700011H14RIK	ILMN_1240624	0.4264888	0.3197876	0.0026488	0.9921006	0.006664	0.0011802	5.766446667	5.738492105	5.738245588	5.645190909				
4921505C17RIK	ILMN_2552030	0.3260702	0.0961802	0.0023143	0.5557595	0.0192574	0.0277537	6.429206667	6.39755	6.381835294	6.309031818				
ZSCAN2	ILMN_2481127	0.1016669	0.5049158	0.0025841	0.1920094	0.0493325	0.0019485	5.832116667	5.778686842	5.812569118	5.711959091				
ATL1	ILMN_2689284	0.0990851	0.0909213	0.0022863	0.97929	0.0491835	0.0478299	5.71863	5.665681579	5.666389706	5.5989				
COPZ1	ILMN_1223544	0.5564383	0.2108712	0.0079137	0.4514929	0.0094923	0.0431359	6.3019	6.281852632	6.259785294	6.182704545				
4921506M07RIK	ILMN_1226012	0.767214	0.1867961	0.004993	0.2845122	0.0061465	0.0237657	5.789413333	5.778978947	5.748717647	5.670604545				
4921506J03RIK	ILMN_2541863	0.058196	0.2388861	0.000234	0.3718193	0.0199308	0.0033724	9.92524	9.871423684	9.893135294	9.807481818				
GADD45GIP1	ILMN_2830860	0.7473828	0.3343774	0.0065388	0.1661634	0.0033004	0.035656	5.926886667	5.9394	5.892926471	5.809209091				
RDH1	ILMN_1230677	0.813449	0.2978746	0.0008105	0.3661283	0.000248	0.0023484	5.70993	5.703157895	5.681833824	5.592604545				
IKZF5	ILMN_2963412	0.4399219	0.4429187	0.0164708	0.8589593	0.024909	0.0071278	7.25512	7.225368421	7.230170588	7.138109091				
LOC385046	ILMN_2541806	0.430148	0.1400567	0.0010304	0.4703621	0.0012962	0.0068437	5.73528	5.711797368	5.694930882	5.618386364				
BC057371	ILMN_2676745	0.3145201	0.5342862	0.0025901	0.4806966	0.0001558	1.217E-05	5.73612	5.768484211	5.751522059	5.619290909				
9830118G07RIK	ILMN_1214091	0.1906198	0.1768389	0.0059989	0.8446158	0.0326653	0.0100365	6.56715	6.525489474	6.530077941	6.450931818				
MRPS31	ILMN_2723162	0.1588756	0.3209264	0.0008342	0.4173279	0.0486679	0.0016695	5.696653333	5.649865789	5.670588235	5.580590909				
1600029O10RIK	ILMN_2546461	0.0997031	0.9814076	0.0169889	0.0514996	8.173E-05	0.0130412	7.692803333	7.769444737	7.691802941	7.576986364				
OLFR125	ILMN_2730288	0.1313929	0.5800322	0.0003406	0.2929673	0.0309284	0.001393	5.710643333	5.670455263	5.696926471	5.594827273				
SPCS2	ILMN_2762956	0.1761219	0.3131761	0.0115366	0.6079863	6.658E-05	0.000131	5.935356667	5.986028947	5.970344118	5.819986364				
BCL10	ILMN_1215785	0.5150295	0.3431271	0.0067767	0.8020252	0.0235364	0.0239616	6.291966667	6.268534211	6.260922059	6.176790909				
BCL11B	ILMN_1223119	0.1978185	0.243168	0.0250749	0.6949597	0.0005304	0.0001287	6.466986667	6.517447368	6.505741176	6.351922727				
control_ILMN_137	ILMN_1379152	0.217537	0.0923344	0.0022973	0.6192088	0.016625	0.0433882	5.677653333	5.641942105	5.629272059	5.562736364				
DPPA4	ILMN_3155408	0.1739081	0.2702328	0.0008807	0.4273251	0.0264242	0.0002288	5.814133333	5.774526316	5.790995588	5.699431818				
D12WSU95E	ILMN_1224518	0.2458634	0.989498	0.0016895	0.1792432	2.626E-05	0.0006446	9.425403333	9.463215789	9.425804412	9.310904545				
1700045I19RIK	ILMN_2967255	0.1534703	0.2851101	0.0006986	0.543461	0.0309232	0.0036094	5.913596667	5.870136842	5.885326471	5.799536364				
MKI67	ILMN_1222340	0.2184501	0.1058261	0.0015179	0.6875769	0.0138791	0.0219347	5.71464	5.682371053	5.672839706	5.600581818				
HIRA	ILMN_1255703	0.6950285	0.4770547	0.0026956	0.1888999	0.0015154	1.642E-05	5.71061	5.698692105	5.730233824	5.596768182				
BC016608	ILMN_2652989	0.5083434	0.2407567	0.0072926	0.5462652	0.0029047	0.0137972	5.873616667	5.852886842	5.838388235	5.7599				
HIVEP3	ILMN_3151547	0.6221573	0.5784284	0.0063473	0.9821112	0.02066	0.0080704	5.8143	5.795202632	5.795891176	5.700604545				
CDKN1B	ILMN_2638428	0.917025	0.2609223	0.0009443	0.1910727	0.0007663	0.004622	6.319963333	6.323197368	6.290032353	6.206463636				
TMEM146	ILMN_1234738	0.5596329	0.2605099	0.0104122	0.5389246	0.0080401	0.0295507	6.1685	6.148844737	6.132217647	6.056054545				
ELF2	ILMN_1251296	0.6285277	0.9913975	0.0172094	0.5581871	0.0130433	0.0035537	6.475283333	6.457747368	6.474922059	6.362845455				
LOC100046343	ILMN_1230976	0.9248882	0.4685428	0.0162763	0.4630621	0.0043081	0.0178511	8.632353333	8.628797368	8.607308824	8.521077273				

INSL6	ILMN_2677859	0.6412477	0.6050936	0.0176684	0.9547704	0.0058919	0.0015237	9.453976667	9.436828947	9.438182353	9.34275					
TSSC1	ILMN_2457324	0.5346915	0.410146	0.011202	0.1038773	0.0344106	0.0006812	7.696453333	7.670657895	7.728852941	7.585313636					
APOM	ILMN_2769418	0.7480536	0.1164096	0.0016309	0.1694297	0.001166	0.0114258	6.10942	6.101231579	6.071398529	5.998286364					
BCORL1	ILMN_2755424	0.5064533	0.1306181	0.0057532	0.4880663	0.0243868	0.0204965	6.14217	6.119342105	6.101613235	6.031768182					
KAT2B	ILMN_1215305	0.8074212	0.6843084	0.014961	0.4354379	0.0025428	0.0064719	6.576886667	6.58595	6.563575	6.466563636					
DPPA1	ILMN_2656079	0.7292075	0.963383	0.0039038	0.7264915	0.0030607	0.0015125	5.75981	5.748876316	5.758391176	5.649590909					
LOC675899	ILMN_1225187	0.6992463	0.5575826	0.0012713	0.9400753	0.0002217	7.695E-07	11.88372667	11.89631579	11.89802353	11.77363636					
CDH23	ILMN_1252019	0.5014447	0.4307809	0.0049608	0.9860706	0.0304386	0.0154897	5.970186667	5.945952632	5.945426471	5.860313636					
LOC384240	ILMN_1231241	0.2925775	0.1219751	0.0013544	0.6701582	0.0059351	0.0082697	5.845473333	5.816294737	5.807029412	5.735695455					
PHIP	ILMN_1234202	0.0592684	0.3520945	0.0352473	0.2092254	1.647E-05	0.0013468	8.148903333	8.24305	8.191986765	8.039681818					
ZCCHC11	ILMN_1231967	0.7880921	0.5355275	0.0122213	0.7373265	0.0118006	0.0075116	5.953363333	5.943892105	5.934814706	5.844218182					
C030004B10RIK	ILMN_1259439	0.7356044	0.8161726	0.0048939	0.8086789	0.0113031	0.0002193	5.714183333	5.7017	5.707923529	5.605068182					
SH3BP2	ILMN_1255766	0.6289259	0.1060738	0.0005954	0.2239723	0.0008508	0.0113141	10.99547	10.98236842	10.95497941	10.88651364					
9030003C19RIK	ILMN_2556478	0.3612766	0.2266165	0.0017932	0.7949485	0.012478	0.019561	6.30629	6.278178947	6.271342647	6.197777273					
PTP4A2	ILMN_2647282	0.3416223	0.6921419	0.0495571	0.0973134	0.0026303	0.0369895	8.967936667	9.012752632	8.951845588	8.859495455					
B230120H23RIK	ILMN_2595189	0.7192865	0.7721243	0.0055388	0.4541691	0.0084984	0.0005516	5.60376	5.592065789	5.612216176	5.495790909					
LOC630421	ILMN_1221849	0.0597593	0.2054214	0.0017667	0.409467	0.0443924	0.0065254	6.025783333	5.977681579	5.995302941	5.917995455					
MYO5C	ILMN_1236641	0.7752801	0.095816	0.0021753	0.1881018	0.0106405	0.0488772	5.774123333	5.765497368	5.730282353	5.666459091					
A130027D22RIK	ILMN_1233685	0.2387366	0.5718522	0.0027726	0.4099722	0.0154264	0.0014254	6.198296667	6.165173684	6.183789706	6.090895455					
TMPO	ILMN_1220051	0.5256872	0.5067008	0.0105314	0.1571467	0.0055601	0.0389159	8.557416667	8.585160526	8.533298529	8.450218182					
HSD3B2	ILMN_1214838	0.3015491	0.3966672	0.0133709	0.8320674	0.0276584	0.0232042	5.771093333	5.73905	5.744586765	5.663945455					
NDRG1	ILMN_1250195	0.7393745	0.8440065	0.0138642	0.8707552	0.0126874	0.0126154	7.336333333	7.323686842	7.329026471	7.229204545					
RAB4A	ILMN_1212902	0.6265947	0.8422009	0.0252261	0.7357147	0.0086563	0.0096978	6.843623333	6.862610526	6.850907353	6.736786364					
RPL32	ILMN_1232890	0.5442472	0.2599497	5.347E-05	0.5956239	0.0002718	0.0006143	13.83211333	13.81921842	13.80899412	13.72541364					
A930025D01RIK	ILMN_1223523	0.5576374	0.6523893	0.0140034	0.1994279	0.0219357	0.0003644	6.41673	6.396328947	6.430195588	6.310840909					
NUFIP2	ILMN_2498813	0.5694042	0.1232094	0.0029603	0.3705824	0.0147044	0.0303234	6.071443333	6.053294737	6.030458824	5.966290909					
LOC236831	ILMN_1253568	0.8342139	0.4698155	0.0014248	0.2468924	5.101E-05	9.813E-05	12.44257667	12.44817105	12.42664118	12.33750455					
GPR12	ILMN_1221410	0.3734723	0.1790159	0.0027823	0.721614	0.0155188	0.0178744	5.7837	5.755857895	5.747101471	5.679290909					
GLRA1	ILMN_2657254	0.1164174	0.0615803	0.0008728	0.7330262	0.0218002	0.0460066	5.690036667	5.6508	5.643054412	5.585631818					
TRPM1	ILMN_3162921	0.5179639	0.3317461	0.0045132	0.0694575	0.0100765	5.237E-05	5.66866	5.650436842	5.694383824	5.564486364					
MIS12	ILMN_2592174	0.6893694	0.6094327	0.0311427	0.9283434	0.0061832	0.0024893	5.971716667	5.986276316	5.988970588	5.867713636					
USP47	ILMN_2455217	0.2820285	0.0819176	0.0014364	0.4680971	0.0103573	0.0483787	6.113853333	6.0853	6.068192647	6.010090909					
DEFB14	ILMN_3161733	0.215456	0.4928357	0.0066859	0.4878044	0.0389184	0.0087454	5.79444	5.758081579	5.775191176	5.690909091					
1600032L17RIK	ILMN_2524058	0.4028161	0.6061978	0.0061233	0.1193815	0.0179857	0.0002792	6.07718	6.053210526	6.091069118	5.97365					
HEATR5B	ILMN_2625626	0.574324	0.276723	0.0046491	0.6474795	0.01455	0.0132789	6.279636667	6.262047368	6.250626471	6.176159091					
EP400	ILMN_2575376	0.7467958	0.2364198	0.0061003	0.3534152	0.0009459	2.344E-05	5.911806667	5.921736842	5.944972059	5.808472727					
C130075H21RIK	ILMN_1217814	0.4372626	0.8272666	0.0029509	0.2466091	0.0309872	0.0004333	5.863153333	5.838913158	5.868792647	5.76005					
MYB	ILMN_2683910	0.3349069	0.6143999	0.0060498	0.5851071	0.0408895	0.0095484	5.96478	5.935865789	5.950458824	5.861781818					
2810402E24RIK	ILMN_1251628	0.1019773	0.1865506	0.0019535	0.6416586	0.0311513	0.0141334	6.330663333	6.285539474	6.296026471	6.227672727					
DLGAP2	ILMN_2937486	0.7642321	0.7416106	0.0200202	0.4795674	0.0100723	0.014501	5.728533333	5.739528947	5.718060294	5.625627273					
COL23A1	ILMN_2650447	0.7700803	0.9975686	0.0090815	0.7133976	0.0005494	0.000973	7.128406667	7.137160526	7.128491176	7.025504545					
FAAH	ILMN_1233816	0.3635331	0.0935749	0.006972	0.4402115	0.0206574	0.0464402	5.98956	5.964115789	5.946645588	5.886959091					
DLC1	ILMN_2648569	0.4902596	0.5212585	0.0257642	0.8837077	0.0464994	0.022319	5.824196667	5.799747368	5.803795588	5.721713636					
E230016F22RIK	ILMN_2571134	0.0980216	0.0810981	0.0016428	0.9918235	0.0420266	0.0412482	5.729066667	5.684757895	5.684539706	5.627031818					
1700073E17RIK	ILMN_2449074	0.7104372	0.5882121	0.0012296	0.8795209	0.0005211	0.0004859	5.641803333	5.631934211	5.628777941	5.539877273					
MDM2	ILMN_1250774	0.1070369	0.1149383	0.0015467	0.8567834	0.0393432	0.0273539	10.50170333	10.45636579	10.46051324	10.39990909					
E530016P10RIK	ILMN_1224376	0.2990389	0.7400947	0.00936	0.298036	0.0488758	0.0016525	6.104856667	6.070465789	6.095805882	6.003086364					
ZFP212	ILMN_2521753	0.9554209	0.6328582	0.0128533	0.5837145	0.0215432	0.0014743	6.38679	6.384781579	6.401176471	6.285086364					
IFI204	ILMN_2623314	0.2394054	0.1406812	0.0036127	0.8595867	0.0187063	0.0137932	5.521196667	5.489834211	5.486161765	5.420104545					
TRPV3	ILMN_2817797	0.2402655	0.2562584	0.0070217	0.9649527	0.0241875	0.0320415	5.62817	5.598486842	5.597452941	5.527354545					
SEC1	ILMN_2733335	0.7407323	0.8997303	0.0491737	0.7662316	0.0375697	0.0126434	5.754276667	5.740523684	5.749694118	5.653522727					
9330133O14RIK	ILMN_2511527	0.8767403	0.318911	0.0196879	0.4675145	0.048507	0.0344143	6.23197	6.226260526	6.205191176	6.131222727					
GALNT1	ILMN_2625681	0.9714869	0.2975368	0.0232723	0.2390968	0.0181203	0.0003745	7.85888	7.857452632	7.895080882	7.758145455					
FOXJ3	ILMN_2650040	0.736441	0.4666574	0.014201	0.7106411	0.0127129	0.0117895	8.670526667	8.657994737	8.647885294	8.569918182					
1810005K13RIK	ILMN_1246137	0.4055711	0.1510678	0.007145	0.5801246	0.0198345	0.033473	5.77925	5.75325	5.740070588	5.678704545					
CLASP1	ILMN_1254008	0.731198	0.5150611	0.0017794	0.3022383	0.0017634	0.0230209	6.05413	6.064752632	6.034072059	5.95395					
6030408K21RIK	ILMN_1218028	0.9050204	0.1935505	0.0009579	0.2122895	0.0008454	0.0038344	5.669393333	5.666268421	5.640398529	5.569368182					
A930008A22RIK	ILMN_2423730	0.0803392	0.6588565	0.0074583	0.1208183	8.172E-06	0.0004926	8.380963333	8.430597368	8.393158824	8.281059091					
1810007M14RIK	ILMN_2681311	0.9867738	0.3264536	0.0153026	0.2792479	0.0105098	0.0498006	6.890543333	6.891115789	6.859664706	6.790654545					
BC003331	ILMN_2719794	0.254712	0.5875327	0.0120195	0.5413595	0.0224451	0.0181672	7.384963333	7.351997368	7.367855882	7.285531818					

LOC382061	ILMN_1245034	0.9446984	0.3669927	0.0214387	0.351454	0.0090476	0.0014338	10.37113	10.37368684	10.40468824	10.27182273					
NAT12	ILMN_2623754	0.7368378	0.1485143	0.0155508	0.2500962	0.0144701	0.0293627	8.314633333	8.302757895	8.275223529	8.215740909					
OLFR782	ILMN_3161983	0.4504481	0.9262584	0.0059004	0.2714201	0.0111752	0.0002495	5.737763333	5.716676316	5.74	5.639059091					
CPEB2	ILMN_1236346	0.754902	0.7192569	0.0028115	0.4630443	0.0006974	0.0044823	5.658066667	5.666544737	5.648361765	5.559427273					
2510049I19RIK	ILMN_2778799	0.9146496	0.4162817	0.0081101	0.4380233	0.0055305	0.0264759	6.335453333	6.332197368	6.311452941	6.236859091					
LCN3	ILMN_1236379	0.3962847	0.4056312	0.0085751	0.9270237	0.038493	0.0271877	5.884476667	5.856815789	5.859325	5.786277273					
9930021J03RIK	ILMN_2626694	0.8473104	0.4195328	0.0096859	0.2816464	0.0050583	0.0227078	5.63169	5.637307895	5.609635294	5.53385					
LOC268939	ILMN_1215666	0.9716605	0.2374067	0.0104938	0.1445345	0.0009112	0.0418775	6.025216667	6.026355263	5.989661765	5.928159091					
IGHV14S3_X03573	ILMN_2453303	0.2247471	0.1772146	0.0113145	0.9609478	9.704E-05	1.92E-05	6.005066667	6.045584211	6.044322059	5.908227273					
4930524L23RIK	ILMN_2610591	0.9763316	0.4440562	0.0031664	0.4414658	0.00327	0.0050724	5.51084	5.509984211	5.492057353	5.414113636					
NANP	ILMN_2629922	0.4439226	0.3354253	0.0027216	0.8669722	0.0134795	0.0132829	5.710083333	5.689892105	5.685992647	5.613513636					
PCSK2	ILMN_1244247	0.9827126	0.5580062	0.0171803	0.5454144	0.0122208	0.0150089	5.669273333	5.668607895	5.653467647	5.572977273					
C87414	ILMN_1243166	0.8301125	0.5806195	0.0205123	0.3878184	0.004923	0.0367684	5.990493333	5.997397368	5.972451471	5.8942					
TMEFF2	ILMN_2518100	0.5540378	0.307364	0.0054996	0.6778876	0.0212317	0.0347081	5.677973333	5.660439474	5.649566176	5.581804545					
MIA3	ILMN_2600646	0.8629119	0.5002241	0.0100169	0.619891	0.0060729	0.0003258	5.843996667	5.849473684	5.862173529	5.747836364					
EG546913	ILMN_2531690	0.8300353	0.834077	0.0010234	0.9661145	0.0006485	0.0001001	5.761706667	5.767334211	5.766423529	5.665590909					
A630040J04RIK	ILMN_1252015	0.4837888	0.87064	0.003201	0.5272259	0.0004243	0.0008928	5.993063333	6.012613158	5.997267647	5.897040909					
HARS	ILMN_2769030	0.4861468	0.3140142	0.0113082	0.8091924	0.0132759	0.0117812	6.238436667	6.216931579	6.211557353	6.142640909					
HRK	ILMN_1229407	0.2096328	0.1223179	0.0028847	0.8953488	0.0322491	0.0274662	5.75883	5.723236842	5.720327941	5.6631					
LOC100045437	ILMN_1228965	0.7505552	0.926885	0.0100705	0.7929019	0.0077785	0.0061808	5.742806667	5.732802632	5.739961765	5.647127273					
AFMID	ILMN_2702894	0.7349138	0.5055376	0.0152111	0.7727553	0.0480428	0.0461388	6.246823333	6.234655263	6.225633824	6.151559091					
PMS1	ILMN_1250517	0.6367352	0.7197332	0.0222742	0.8956758	0.023488	0.0225456	5.817356667	5.801978947	5.805682353	5.722281818					
ID2	ILMN_2887239	0.8702895	0.4751141	0.0279837	0.5421713	0.0158696	0.0431245	6.11898	6.112697368	6.094567647	6.024290909					
FABP2	ILMN_2706493	0.8033747	0.7866504	0.0134488	0.9845572	0.0017729	0.0017791	5.574673333	5.581957895	5.582427941	5.48005					
ATG12	ILMN_2663570	0.2765947	0.6693377	0.0022197	0.4153576	0.0299606	0.0036453	8.973293333	8.94255	8.962186765	8.87895					
FBXW13	ILMN_2625152	0.5506756	0.6042237	0.0143692	0.8820256	0.037303	0.0150896	5.958916667	5.941	5.944770588	5.864777273					
TDH	ILMN_1244724	0.2991268	0.2202766	0.001097	0.8646909	0.0280882	0.037229	5.632846667	5.605236842	5.600979412	5.538713636					
CALCRL	ILMN_1235390	0.6192884	0.959516	0.0388553	0.5187058	0.0344821	0.0042841	5.57102	5.552502632	5.569420588	5.476895455					
IL1F9	ILMN_2658704	0.9599679	0.362586	0.0027602	0.326151	0.004563	0.0107344	5.66582	5.667273684	5.643604412	5.571768182					
SYT10	ILMN_2724250	0.367285	0.7220084	0.0144716	0.4300129	0.0404325	0.0041577	5.726353333	5.6979	5.716591176	5.632418182					
MEX3A	ILMN_2479681	0.3380093	0.6957226	0.0209027	0.4889153	0.0224868	0.0151692	6.931541379	6.896444737	6.913239706	6.837772727					
PRICKLE2	ILMN_3015864	0.8467823	0.7874096	0.0209157	0.9691369	0.0147291	0.0034501	5.850416667	5.857647368	5.858801471	5.756763636					
5830415L20RIK	ILMN_1257675	0.976909	0.9407398	0.0148166	0.9090173	0.0213148	0.0025673	7.799203333	7.800326316	7.79705	7.705868182					
GAPVD1	ILMN_1238871	0.6367536	0.1355577	0.0054484	0.2719336	0.0050197	0.029116	9.875493333	9.862042105	9.838317647	9.782222727					
LOC381770	ILMN_2539337	0.4275937	0.7376624	0.0151302	0.5639629	0.0383306	0.0148801	6.123156667	6.095863158	6.112223529	6.03					
TATDN3	ILMN_2647204	0.7744865	0.2357237	0.0013057	0.2930066	0.0004448	0.0243529	5.724313333	5.717452632	5.693994118	5.631331818					
KRT32	ILMN_2895346	0.8325026	0.5156987	0.0065035	0.639505	0.0011419	0.0001087	5.993876667	5.999742105	6.010070588	5.901004545					
LRRC18	ILMN_2728504	0.8949315	0.5910011	0.005234	0.6690196	0.003179	0.0007924	5.526923333	5.530942105	5.542358824	5.434140909					
NIN	ILMN_2778195	0.6803254	0.9897238	0.0093726	0.5882718	0.0017088	0.0014083	8.5343	8.546615789	8.533970588	8.442313636					
5830411J07RIK	ILMN_2679267	0.9168752	0.5707688	0.0080081	0.6350218	0.0095103	0.0108144	5.85217	5.849081579	5.837338235	5.760313636					
EEFSEC	ILMN_2587281	0.7897267	0.9248816	0.0102229	0.6720642	0.0051822	0.0032708	5.65683	5.664715789	5.654432353	5.565031818					
SPDYB	ILMN_2756787	0.4188197	0.3298557	0.0127321	0.9302795	0.0425628	0.0364783	5.668073333	5.642892105	5.640694118	5.576868182					
RNF150	ILMN_3032501	0.1040409	0.2494973	0.0228449	0.3483212	8.548E-05	7.229E-05	5.896763333	5.955207895	5.930469118	5.805768182					
SYNGR4	ILMN_3070294	0.5860337	0.7467844	0.0167894	0.7784429	0.0481033	0.0195099	5.656433333	5.638892105	5.646776471	5.565595455					
6030408B16RIK	ILMN_3125764	0.3754552	0.3898573	0.0040291	0.8844397	0.036486	0.0149594	6.25216	6.227231579	6.230682353	6.161786364					
C130039E17RIK	ILMN_2494727	0.2570242	0.9444619	0.0074785	0.1965718	0.0186735	0.0012731	5.731136667	5.703355263	5.729467647	5.640990909					
KRIT1	ILMN_2701155	0.2975452	0.1242828	0.0152567	0.6635597	0.0009445	8.52E-05	5.64008	5.672247368	5.683764706	5.549959091					
control_ILMN_137	ILMN_1378774	0.9307556	0.2861095	0.0362832	0.2053394	0.0299291	0.0019757	5.669826667	5.666792105	5.707342647	5.580013636					
PAPPA	ILMN_2630646	0.3757235	0.2469496	0.0127078	0.7949767	0.0313073	0.0437685	5.948626667	5.924163158	5.918261765	5.858904545					
1700016L04RIK	ILMN_2499229	0.9068536	0.3496515	0.003728	0.2877742	0.0068198	0.0356119	5.716706667	5.719863158	5.692752941	5.627036364					
C130072C03RIK	ILMN_1233936	0.8083855	0.8398444	0.0117163	0.6209926	0.004497	0.0141431	6.081323333	6.089007895	6.075160294	5.992027273					
OLFR564	ILMN_2771303	0.6059235	0.3617566	0.0038621	0.7522095	0.0085074	0.0036343	5.610586667	5.597744737	5.591648529	5.521359091					
1700016G05RIK	ILMN_2637903	0.7065492	0.586252	0.0380929	0.8980731	0.0087043	0.0024676	5.763153333	5.776560526	5.780139706	5.674022727					
RgenericRIP1	ILMN_2681694	0.5272695	0.4436967	0.0030417	0.9475417	0.0227677	0.0158192	5.954443333	5.936397368	5.934783824	5.865727273					
K20D4	ILMN_1240743	0.9620794	0.4376337	0.0016224	0.3884916	0.0019114	0.0055894	5.705186667	5.706357895	5.688022059	5.616690909					
4922502D21RIK	ILMN_2593944	0.4155873	0.2608502	0.0014164	0.8094367	0.0287873	0.0357882	5.802656667	5.779581579	5.773558824	5.714322727					
GM949	ILMN_2531100	0.7554924	0.5739886	0.0258405	0.812046	0.0276837	0.025157	5.67855	5.668557895	5.662460294	5.590413636					
DLG4	ILMN_2710764	0.8894548	0.6313459	0.0098786	0.719497	0.0092212	0.0139952	6.05743	6.053318421	6.044314706	5.969368182					
PTPN22	ILMN_2766313	0.8914719	0.3978819	0.0059982	0.2959911	0.0071583	0.0002047	5.784923333	5.781565789	5.804307353	5.697240909					
MED1	ILMN_2695175	0.6978466	0.3155412	0.0061145	0.5136009	0.0126153	0.0489572	5.69954	5.688523684	5.672022059	5.612195455					

HPN	ILMN_1240252	0.7037546	0.8400336	0.0251248	0.4693431	0.011654	0.0031729	5.78737	5.775389474	5.793452941	5.700045455					
USP47	ILMN_1256774	0.8866563	0.0842761	0.0014934	0.0608501	0.0099391	2.783E-05	6.331733333	6.327818421	6.377101471	6.24445					
CTDSPL2	ILMN_3154869	0.8735733	0.4599275	0.0147125	0.5616849	0.0151192	0.0017049	6.688386667	6.693771053	6.710814706	6.601177273					
PARP11	ILMN_1221241	0.8903692	0.6638582	0.0011354	0.5632186	0.0047832	0.0101482	5.620256667	5.624031579	5.609260294	5.533118182					
LOC381561	ILMN_2536925	0.1723748	0.216863	0.0075951	0.6464934	0.0439059	0.0093054	13.19958667	13.16311842	13.17164853	13.1126					
V1RD9	ILMN_2959825	0.9788191	0.1317526	0.0069015	0.0979927	0.0057014	5.894E-06	5.658806667	5.657992105	5.697108824	5.572222727					
SPN	ILMN_1255969	0.5596473	0.7111714	0.0075162	0.8578705	0.0095021	0.0137105	5.89838	5.884368421	5.888555882	5.811963636					
KCNK4	ILMN_2625370	0.6927491	0.8658761	0.0447239	0.5016589	0.0064063	0.025991	5.664273333	5.677852632	5.658711765	5.577881818					
PAX6	ILMN_2504978	0.6902548	0.2519094	0.0135426	0.0834441	0.0035471	0.0395486	5.669473333	5.681165789	5.640964706	5.583190909					
COPS3	ILMN_1218678	0.5169321	0.6368832	0.0237309	0.2024763	0.0050189	0.0265416	6.04268	6.063476316	6.029414706	5.956536364					
OLFR894	ILMN_2631775	0.5583272	0.9984709	0.0439596	0.4213578	0.0033098	0.0079279	5.7664	5.789839474	5.766469118	5.6803					
9530053H22	ILMN_2617639	0.4188925	0.3540549	0.0035781	0.054257	0.0003069	0.011957	5.551996667	5.571878947	5.531242647	5.465995455					
5830472H07RIK	ILMN_2755293	0.3650164	0.6660751	0.0204663	0.1182634	0.0003978	0.0274633	6.253593333	6.282686842	6.240239706	6.167636364					
D930007M19RIK	ILMN_2581178	0.7633954	0.6060461	0.0224101	0.335377	0.0045154	0.0172931	6.347126667	6.356386842	6.333030882	6.261340909					
D130078K04RIK	ILMN_2569683	0.6663672	0.2573497	0.0413511	0.0513247	0.028787	0.0001493	5.92102	5.905407895	5.957402941	5.835468182					
LOC384287	ILMN_1226110	0.5438828	0.5814111	0.0033333	0.1920168	0.0174853	0.0001765	5.600046667	5.584278947	5.612377941	5.514790909					
LOC100048049	ILMN_2708610	0.9521813	0.2559068	0.011481	0.2112315	0.0108032	0.0493348	5.83809	5.839768421	5.810255882	5.752986364					
1700112H15RIK	ILMN_2743541	0.6486149	0.3201502	0.0108812	0.1093398	0.0211543	0.0002793	5.602223333	5.589636842	5.628055882	5.517240909					
RNF17	ILMN_2741929	0.7134597	0.3039073	0.0055782	0.1747023	0.0115691	0.0273409	5.560576667	5.571157895	5.537982353	5.475609091					
NARF	ILMN_1244845	0.3281369	0.0721462	0.0176127	0.4176605	0.0027905	0.0001596	7.168896667	7.19995	7.223792647	7.084013636					
HSPA1B	ILMN_2530672	0.771466	0.9805427	0.0373104	0.7019187	0.0198453	0.017804	5.990493333	6.0012	5.989733824	5.905659091					
B930071N01RIK	ILMN_1247868	0.9632328	0.6442669	0.0062749	0.6522583	0.0040953	0.0014718	5.752686667	5.753815789	5.764302941	5.668054545					
SEMA3E	ILMN_1228525	0.6303928	0.3243436	0.00681	0.6054607	0.0058388	0.0090843	5.622603333	5.610747368	5.600951471	5.538277273					
GALNT6	ILMN_2627386	0.8144688	0.7918429	0.0284545	0.5404713	0.0260369	0.0019673	5.865516667	5.857652632	5.872851471	5.781409091					
NCOA2	ILMN_2573793	0.8386801	0.9791901	0.0473111	0.8113465	0.0387878	0.0101734	5.96424	5.957523684	5.963505882	5.880186364					
LOC385027	ILMN_1255900	0.6575389	0.4828937	0.0095649	0.2241955	0.0282209	0.0016544	5.646326667	5.633852632	5.665861765	5.562377273					
SPRR2K	ILMN_2990390	0.4610699	0.1988329	0.0202078	0.5001156	0.0007792	0.000363	5.623606667	5.644615789	5.662151471	5.539818182					
STAT4	ILMN_2792599	0.9033132	0.9223381	0.0125316	0.814329	0.0093633	0.0156657	5.838816667	5.842352632	5.836001471	5.755045455					
1700006H02RIK	ILMN_1253202	0.7195873	0.4958858	0.0043993	0.7777704	0.0214114	0.0199032	5.87839	5.868286842	5.861352941	5.794681818					
OLFR463	ILMN_2702643	0.941253	0.9542228	0.0460991	0.8736715	0.0244688	0.0162632	5.806456667	5.809223684	5.804622059	5.722768182					
LOC637082	ILMN_1233896	0.9775942	0.7745997	0.0244268	0.7222327	0.0133239	0.0034332	5.796636667	5.795723684	5.804989706	5.712972727					
CHMP7	ILMN_2588610	0.7259657	0.6457792	0.0163974	0.9779851	0.0047052	0.0004421	9.48094	9.490673684	9.491242647	9.397440909					
AGPAT5	ILMN_1250939	0.9614614	0.5703106	0.0238288	0.574637	0.0157558	0.0166591	5.9402	5.938721053	5.925610294	5.856831818					
GPRIN3	ILMN_2722319	0.7193688	0.409426	0.0162081	0.1731738	0.0048228	0.0223676	5.649423333	5.661078947	5.627986765	5.566109091					
R3HDM1	ILMN_2759868	0.5127528	0.8274176	0.0139212	0.3080578	0.043072	0.0024589	6.22318	6.204939474	6.228605882	6.140118182					
GM239	ILMN_2814357	0.8564432	0.3607762	0.0107896	0.2673967	0.0125712	0.0213908	5.75041	5.755331579	5.730616176	5.667736364					
genericC	ILMN_2606088	0.3693444	0.1421332	0.0063459	0.6205897	0.0007251	4.249E-05	5.638906667	5.663857895	5.675654412	5.556236364					
GUCY2C	ILMN_2911551	0.9311439	0.6791989	0.0266953	0.727426	0.0192255	0.0190927	5.75101	5.748381579	5.739895588	5.668413636					
DNAJC28	ILMN_2592571	0.7952743	0.9228959	0.0229884	0.6610701	0.006099	0.0044932	5.995306667	5.987768421	5.998058824	5.912995455					
A630057J21RIK	ILMN_1222357	0.2314964	0.6982961	0.0121469	0.0568623	0.0004558	0.0115815	5.72068	5.759102632	5.710363235	5.638481818					
PDK2	ILMN_2737658	0.9107416	0.6160621	0.0448763	0.491808	0.0248585	0.0440472	5.95138	5.955163158	5.936645588	5.869513636					
1110032A03RIK	ILMN_1215043	0.6042382	0.7715371	0.0053992	0.7763488	0.0169362	0.0057653	5.859373333	5.846365789	5.852595588	5.777881818					
2310016E02RIK	ILMN_1254063	0.7048363	0.6541008	0.0108308	0.9525587	0.0146772	0.0200185	5.7544	5.743484211	5.742011765	5.672968182					
C330011J12RIK	ILMN_2615978	0.8586984	0.9201107	0.0156124	0.7486874	0.0019331	0.0052056	5.719083333	5.714607895	5.721825	5.637795455					
JRKL	ILMN_1255837	0.9427248	0.8029279	0.0412463	0.8452461	0.023158	0.0194457	5.823186667	5.821102632	5.816498529	5.741981818					
ZFP120	ILMN_2456317	0.8626171	0.8674089	0.0107547	0.9891192	0.0092609	0.0063626	5.540176667	5.535910526	5.536211765	5.459018182					
KISS1	ILMN_2735829	0.7658177	0.2161865	0.007277	0.1117005	0.0202354	0.0001909	5.697123333	5.689555263	5.726642647	5.6162					
RTN3	ILMN_2572492	0.5310507	0.7196946	0.0224205	0.7369927	0.0375872	0.019464	5.90647	5.888605263	5.896657353	5.825581818					
AW822252	ILMN_2744956	0.9110759	0.6628267	0.0259589	0.4876944	0.0014865	0.0119086	5.76074	5.763905263	5.74905	5.679863636					
LRRC39	ILMN_1230043	0.7845283	0.8889288	0.0136074	0.8897264	0.0015873	0.00431	5.708436667	5.715042105	5.711988235	5.627659091					
SCNN1B	ILMN_2618383	0.8982054	0.8224606	0.0343777	0.668188	0.0192109	0.0030956	5.610303333	5.606213158	5.616464706	5.529563636					
DLX6	ILMN_1213404	0.9865922	0.6995028	0.0138319	0.6976892	0.0132859	0.0016274	5.75542	5.755936842	5.765739706	5.674818182					
GM438	ILMN_1248574	0.2999972	0.5505015	0.0105011	0.6519213	0.0408515	0.0249861	5.745906667	5.720597368	5.730714706	5.665440909					
LOC236397	ILMN_1239998	0.6819703	0.8225891	0.0234194	0.4609324	0.0237734	0.0055854	5.832173333	5.820278947	5.838497059	5.751754545					
LOC381448	ILMN_2528515	0.7862356	0.3355951	0.0078061	0.517919	0.0070339	0.000228	12.88779333	12.89468947	12.90875882	12.80749091					
CCDC104	ILMN_2643580	0.5512377	0.7068019	0.0074226	0.2389928	0.0024549	0.0036015	5.84063	5.859457895	5.831667647	5.760527273					
LOC279638	ILMN_2588075	0.6434951	0.8289066	0.0190066	0.4385002	0.0044609	0.0166612	6.0283	6.041955263	6.022392647	5.948613636					
MADD	ILMN_1256376	0.8841383	0.5873814	0.0049344	0.6978806	0.0174898	0.0249892	5.59808	5.593768421	5.583701471	5.518418182					
LOC333749	ILMN_2530361	0.9961254	0.9454999	0.0089106	0.9434147	0.019264	0.0087053	5.940406667	5.940531579	5.938926471	5.860786364					
AGT	ILMN_1227398	0.9838994	0.7108285	0.0317634	0.6822639	0.0310015	0.0379501	5.643776667	5.644352632	5.633804412	5.564418182					

C77370	ILMN_1218340	0.6355803	0.745389	0.0080885	0.3593414	0.0033986	0.0044323	5.701306667	5.713702632	5.694325	5.621972727					
LOC382454	ILMN_1255829	0.9658817	0.7377271	0.0151115	0.7577817	0.0169871	0.0263888	5.770946667	5.769571053	5.761170588	5.692477273					
9130601C02RIK	ILMN_1216153	0.4110089	0.9474412	0.0052086	0.2671637	0.0448125	0.0007918	5.722513333	5.701268421	5.723889706	5.644172727					
B230216G23RIK	ILMN_2481643	0.5738403	0.4818068	0.0061084	0.1632362	0.0005697	0.0262431	5.642603333	5.6564	5.625255882	5.56435					
BMPR1B	ILMN_1219486	0.4614593	0.6682267	0.013751	0.6987948	0.0050068	0.0054518	5.720253333	5.742613158	5.732064706	5.642009091					
LOC100046568	ILMN_1258903	0.3199598	0.565766	0.0457105	0.5006254	0.0056304	0.0043152	5.87139	5.907021053	5.888016176	5.793381818					
A130003P22RIK	ILMN_1257646	0.7124793	0.8219187	0.0033766	0.8525193	0.0043866	0.0021054	6.037656667	6.046863158	6.042676471	5.959827273					
CFL2	ILMN_1249976	0.6912082	0.4701812	0.0194689	0.7779825	0.0435501	0.0357747	6.263776667	6.252907895	6.246395588	6.18595					
control_ILMN_137	ILMN_1378775	0.2449939	0.2632084	0.032902	0.8118056	0.0016482	0.0006335	5.630993333	5.667786842	5.661682353	5.553213636					
TCFAP2C	ILMN_2783225	0.7620181	0.9408748	0.0280774	0.8084718	0.0113623	0.0264556	5.818516667	5.827960526	5.820864706	5.740859091					
BHLHB3	ILMN_2759227	0.5982206	0.9068422	0.0145963	0.4665519	0.0252029	0.0041333	5.618706667	5.606023684	5.621429412	5.541054545					
UGDH	ILMN_1253090	0.8063706	0.9199699	0.0085069	0.866032	0.0213414	0.0094823	5.862666667	5.856173684	5.860194118	5.78535					
control_ILMN_137	ILMN_1379205	0.7602591	0.9819625	0.0237431	0.7182357	0.0403374	0.014538	5.71972	5.711386842	5.720311765	5.642781818					
CRLF1	ILMN_2747811	0.7979725	0.2898963	0.002394	0.1858294	0.0073264	0.039054	5.672033333	5.679189474	5.647736765	5.595127273					
SOX2	ILMN_1248711	0.77498	0.6449247	0.0219339	0.8646521	0.0241069	0.0305053	5.68709	5.678613158	5.674401471	5.610327273					
LOC229152	ILMN_1240991	0.7580748	0.5264719	0.0070411	0.8449809	0.0341744	0.0058696	5.61709	5.608715789	5.604654412	5.540372727					
2210008F06RIK	ILMN_2429915	0.9158323	0.9785725	0.0492365	0.8718555	0.0341603	0.0179371	5.65113	5.654563158	5.650383824	5.574586364					
9530013H16RIK	ILMN_2559067	0.5771389	0.3207617	0.0167735	0.7213419	0.0493211	0.0486017	5.806686667	5.790413158	5.782267647	5.730295455					
MACROD2	ILMN_1226796	0.7170971	0.440629	0.0205588	0.7170822	0.0260174	0.0165478	5.871886667	5.861818421	5.854417647	5.795531818					
ZBTB44	ILMN_2589136	0.4870447	0.6354427	0.0131313	0.7523904	0.0485749	0.0153755	5.652506667	5.634860526	5.641651471	5.576604545					
LOC209474	ILMN_1245275	0.7909858	0.9579306	0.0172214	0.7236567	0.0147319	0.0078102	5.677973333	5.671436842	5.679282353	5.602077273					
OLFR1158	ILMN_1254931	0.7711087	0.8950205	0.0269546	0.6148863	0.0349077	0.005344	5.90849	5.900489474	5.911680882	5.832872727					
LOC383612	ILMN_1245739	0.9608875	0.4944551	0.0133369	0.5140363	0.0123428	0.0078986	5.58247	5.581242105	5.568833824	5.506859091					
NFAT5	ILMN_2732123	0.7220709	0.6373046	0.013798	0.3755303	0.0051969	0.0342599	5.82748	5.836571053	5.815392647	5.751886364					
IGHV5S15_AF2909	ILMN_2432375	0.2332618	0.593919	0.0082754	0.0524827	0.00034	0.0201746	5.96497	5.996957895	5.952066176	5.889468182					
ELMO2	ILMN_3058341	0.1248663	0.2456725	0.0320446	0.6570195	0.0004545	0.0012868	5.873053333	5.918702632	5.906870588	5.79765					
3110079O15RIK	ILMN_2598181	0.691136	0.3535547	0.0016101	0.1785755	0.0025887	0.0184064	5.66052	5.669471053	5.641986765	5.585468182					
GFRA3	ILMN_3029078	0.622644	0.9274573	0.0176204	0.6517359	0.0389289	0.0210445	5.61845	5.603889474	5.615789706	5.543463636					
OSBPL6	ILMN_1219286	0.421244	0.4236816	0.0162348	0.9386201	0.001723	0.0008632	5.831956667	5.852805263	5.8511	5.757227273					
DLG1	ILMN_2763839	0.738961	0.8043745	0.0497966	0.8747949	0.0096557	0.006355	5.726496667	5.737444737	5.733544118	5.652140909					
D130063P19RIK	ILMN_1238936	0.9480958	0.8417107	0.0270242	0.8912641	0.0207869	0.0134556	5.772983333	5.771063158	5.767867647	5.698895455					
EG634762	ILMN_1214492	0.2631925	0.7925924	0.0173523	0.1008914	0.0003285	0.0095864	5.778483333	5.806255263	5.772413235	5.704395455					
4833419K08RIK	ILMN_1250504	0.9049742	0.7421597	0.0204277	0.8107214	0.0042437	0.0019607	5.580176667	5.582989474	5.587622059	5.506263636					
D130061E05RIK	ILMN_2573307	0.7094617	0.6140585	0.0316363	0.9713841	0.0379944	0.0099984	5.734043333	5.723581579	5.722885294	5.660136364					
TMEM219	ILMN_2689569	0.2764255	0.7262618	0.0377142	0.4317587	0.00117	0.0087928	6.148696667	6.176007895	6.157932353	6.074918182					
OA1	ILMN_1242255	0.487166	0.6445263	0.0299857	0.1361413	0.0012152	0.0120236	5.56251	5.581981579	5.551513235	5.488740909					
OLFR1180	ILMN_2649883	0.8222038	0.6501832	0.0333256	0.8396433	0.0449768	0.032169	5.670083333	5.663052632	5.658069118	5.5964					
EFCAB1	ILMN_2917280	0.4077935	0.7317519	0.0074028	0.5983521	0.0051664	0.0105462	5.710406667	5.73445	5.719770588	5.636781818					
control_ILMN_137	ILMN_1378664	0.7399878	0.5731784	0.0487539	0.8437626	0.0373317	0.0133516	5.725506667	5.737144737	5.743244118	5.652136364					
BRPF1	ILMN_2800859	0.10318	0.456572	0.0187149	0.159891	0.0003744	0.0005215	8.94028	8.991239474	8.957891176	8.866990909					
CORO1B	ILMN_2788546	0.6102325	0.911713	0.014794	0.4833145	0.0055325	0.0155444	11.35823333	11.37271842	11.35536912	11.28498636					
LOC235971	ILMN_1247372	0.5222218	0.3455454	0.0144253	0.7525038	0.0262938	0.0444392	5.526783333	5.511610526	5.505255882	5.453613636					
LOC383940	ILMN_2536510	0.7782556	0.9425511	0.0417094	0.6416792	0.0053564	0.0102398	5.806973333	5.816121053	5.804944118	5.733954545					
RNF207	ILMN_1237330	0.1524907	0.7485933	0.0368134	0.2040052	0.0005451	0.009244	5.854813333	5.893897368	5.863382353	5.781881818					
1700081L16RIK	ILMN_2426180	0.4254824	0.3879785	0.0201724	0.9762544	0.0368069	0.0475511	5.581866667	5.561147368	5.560519118	5.509463636					
OLFR750	ILMN_2773535	0.9980588	0.347413	0.0042447	0.3702135	0.0176438	0.030927	5.666133333	5.666073684	5.646426471	5.593809091					
LOC385626	ILMN_1244702	0.8619168	0.9055099	0.0212374	0.740134	0.0197902	0.0142778	6.048793333	6.053842105	6.045866176	5.977095455					
LOC384513	ILMN_1230132	0.9775506	0.7832103	0.0364382	0.7837895	0.0196173	0.0179293	5.711216667	5.710447368	5.704545588	5.639831818					
LOC385059	ILMN_2542073	0.8332255	0.6486756	0.0130752	0.8179112	0.020155	0.0150889	5.548423333	5.543284211	5.538563235	5.477331818					
4930571C24RIK	ILMN_1228034	0.8721725	0.4482687	0.0370055	0.5462199	0.0375868	0.0367313	5.43153	5.426878947	5.413977941	5.360531818					
PPP1R14D	ILMN_1253015	0.8287285	0.4494114	0.0208985	0.2516059	0.0029559	0.0360052	5.628493333	5.633902632	5.610638235	5.557659091					
GJA7	ILMN_1253346	0.8083451	0.7516472	0.0289331	0.9468602	0.0459395	0.0421959	5.87617	5.869497368	5.867842647	5.8054					
OLFR1085	ILMN_1248151	0.6199621	0.8646509	0.0498615	0.6971271	0.0319696	0.0362017	5.636943333	5.654255263	5.642222059	5.566422727					
NEUROD4	ILMN_2766939	0.9468943	0.9900661	0.0155689	0.9553691	0.0171478	0.0130846	5.671153333	5.669668421	5.670869118	5.60065					
LTBP1	ILMN_1224967	0.9042909	0.6064432	0.0498021	0.6815483	0.0444347	0.0151168	5.68037	5.683613158	5.694298529	5.610181818					
control_ILMN_137	ILMN_1378571	0.9954505	0.7386233	0.0475198	0.7209843	0.0451922	0.0218043	5.820813333	5.820989474	5.812435294	5.750686364					
PIK3R4	ILMN_2543088	0.7555027	0.7071606	0.0334829	0.4513792	0.0199806	0.0449248	5.89115	5.900139474	5.881458824	5.821327273					
2210003I03RIK	ILMN_2710359	0.7059347	0.8516575	0.0226146	0.8690002	0.033072	0.0328199	5.69808	5.689707895	5.693454412	5.628409091					
2900003A17RIK	ILMN_2449674	0.964805	0.6037174	0.048409	0.6149156	0.0353241	0.0051105	6.01923	6.020481579	6.032035294	5.949854545					
EZH2	ILMN_2606429	0.8838703	0.652619	0.0490933	0.7713393	0.0337646	0.0059032	5.613896667	5.618357895	5.625351471	5.544813636					

BTF3L4	ILMN_2658543	0.9205529	0.7966262	0.0240899	0.8624027	0.0129405	0.0187044	5.68815	5.685586842	5.681852941	5.619090909					
LOC676672	ILMN_1236130	0.7936679	0.4266963	0.0324377	0.5855754	0.0184708	0.0031912	5.804406667	5.811518421	5.824616176	5.735581818					
control_ILMN_137	ILMN_1379333	0.1288857	0.9453906	0.0359332	0.1059414	7E-05	0.0131002	5.61851	5.653657895	5.620225	5.549695455					
B230323D24RIK	ILMN_1213297	0.744828	0.9553043	0.025918	0.6176776	0.0321656	0.0037381	5.808683333	5.799486842	5.809979412	5.739909091					
3110052M02RIK	ILMN_2463970	0.8511269	0.5432352	0.0246487	0.3797416	0.0130756	0.0262494	5.663506667	5.668044737	5.650769118	5.595422727					
ENSMUSG0000007	ILMN_1231109	0.8721125	0.9892634	0.0443327	0.85279	0.0362554	0.0186731	5.765793333	5.770668421	5.766132353	5.697813636					
LOC384215	ILMN_2537640	0.1770364	0.7258164	0.0235057	0.1615915	0.0009221	0.0029855	5.820696667	5.862371053	5.829138235	5.752890909					
D530031C13RIK	ILMN_1217757	0.9847102	0.6778231	0.0454001	0.6685376	0.0361697	0.0486874	5.739493333	5.738907895	5.728495588	5.672031818					
LOC277422	ILMN_2527881	0.8943305	0.7573356	0.0298433	0.8656131	0.0412251	0.0344878	5.73676	5.733102632	5.729133824	5.669509091					
A530088H08RIK	ILMN_2601705	0.7311173	0.3941153	0.0449897	0.651595	0.0309327	0.0035692	5.813696667	5.825318421	5.837829412	5.746622727					
HEATR5A	ILMN_2694279	0.7621217	0.7825505	0.0455636	0.9263604	0.0291276	0.0100253	5.68794	5.696721053	5.694547059	5.621004545					
6430527G18RIK	ILMN_2754183	0.1397432	0.6642442	0.0285862	0.1764046	0.0003064	0.0039778	5.60759	5.649342105	5.618372059	5.540731818					
TRPC6	ILMN_2446212	0.9219198	0.7161972	0.0256304	0.6217501	0.0329805	0.0056644	5.624033333	5.621573684	5.632232353	5.557395455					
GM362	ILMN_1222972	0.664285	0.4459988	0.0342508	0.7715016	0.0104932	0.0019287	5.620813333	5.632378947	5.638714706	5.554177273					
PCDHGA8	ILMN_2705452	0.8644894	0.9781795	0.0357837	0.8341874	0.0330759	0.0387445	5.78089	5.776236842	5.781673529	5.714854545					
LOC620009	ILMN_2635082	0.4641503	0.2266315	0.0344629	0.527073	0.0003329	0.0002557	5.591003333	5.607339474	5.618951471	5.525318182					
LOC100043389	ILMN_1256414	0.3365112	0.7213683	0.0117085	0.429638	0.0017856	0.0020938	5.668326667	5.691036842	5.675438235	5.602663636					
SMARCA2	ILMN_1252932	0.682514	0.8805993	0.0116025	0.5392936	0.0120917	0.0206458	5.619723333	5.630702632	5.616189706	5.554095455					
A230066D03RIK	ILMN_2694557	0.7478269	0.6233261	0.030824	0.8742501	0.0379034	0.032595	5.923233333	5.915707895	5.912572059	5.857631818					
UTX	ILMN_2579537	0.8990562	0.9841417	0.0461225	0.8593128	0.0244771	0.0144272	5.6994	5.696263158	5.699866176	5.633977273					
OLFR1230	ILMN_2662149	0.9133439	0.6853267	0.0214246	0.5803146	0.0300035	0.0044939	5.64594	5.643160526	5.655157353	5.580931818					
CREBZF	ILMN_1220787	0.6419102	0.5658142	0.0344117	0.2355857	0.0043483	0.0499252	5.673313333	5.683676316	5.660316176	5.608613636					
RARRES1	ILMN_2605971	0.9575019	0.5198941	0.0496096	0.4359723	0.0341128	0.0052245	5.584843333	5.583405263	5.601169118	5.52025					
D130050A01RIK	ILMN_1215837	0.8581824	0.6589701	0.0075837	0.7938892	0.014337	0.016564	5.641893333	5.638021053	5.632995588	5.577468182					
LOC382783	ILMN_1258648	0.7954538	0.8399388	0.0269605	0.9175459	0.0194251	0.0086414	5.531793333	5.538615789	5.536345588	5.467895455					
C430020E23RIK	ILMN_1221998	0.3414776	0.6328869	0.0417478	0.6185967	0.0027265	0.0162138	5.892203333	5.918694737	5.905788235	5.829490909					
ADAMTSL3	ILMN_1239668	0.3318312	0.7572362	0.0145444	0.4640049	0.0040962	0.0129587	5.798876667	5.823873684	5.806304412	5.736231818					
SCL0070954.1_214	ILMN_2425193	0.8386057	0.6389773	0.0461288	0.429835	0.0331145	0.0051338	5.810163333	5.804763158	5.821651471	5.747618182					
PTCHD2	ILMN_1239751	0.9888121	0.8602277	0.0441889	0.8573384	0.0257898	0.0162493	5.826433333	5.826802632	5.830785294	5.763954545					
CNTN2	ILMN_1249156	0.6646656	0.5252117	0.025749	0.8338597	0.0053485	0.0037035	5.713903333	5.724568421	5.729119118	5.651654545					
6720487G11RIK	ILMN_2649435	0.9648146	0.7236504	0.0305665	0.7335576	0.0149237	0.0083789	5.956016667	5.954921053	5.948966176	5.895059091					
SERPINB3D	ILMN_3009747	0.7532656	0.8360982	0.0413928	0.5101054	0.008756	0.0122501	5.745123333	5.752734211	5.740894118	5.685531818					
CRAT	ILMN_1220786	0.4082949	0.9446803	0.0479651	0.3916915	0.0105247	0.0423267	5.918913333	5.942655263	5.92075	5.859636364					
4921522P10RIK	ILMN_1218156	0.5574131	0.5167879	0.0402099	0.9089929	0.0285183	0.0058245	5.777803333	5.795607895	5.792822059	5.719963636					
A830021M18	ILMN_2595299	0.8494349	0.9223596	0.0452205	0.8981181	0.0297573	0.0158022	5.659886667	5.664492105	5.661938235	5.602090909					
RTN2	ILMN_1250904	0.0915044	0.4321175	0.0320957	0.2950548	0.0010381	0.0072122	5.9209	5.963881579	5.939469118	5.864890909					
RPL10A	ILMN_2902115	0.8282107	0.7101946	0.0090699	0.8779802	0.0149085	0.0182474	14.66292333	14.65870789	14.65606176	14.60712273					
LOC382760	ILMN_2535747	0.925786	0.7432541	0.0422821	0.6572119	0.046817	0.0425333	5.657563333	5.659857895	5.650822059	5.601886364					
4930543E05RIK	ILMN_2584057	0.6327314	0.9142441	0.0320809	0.6454348	0.0201074	0.0164555	5.678906667	5.690528947	5.681104412	5.6236					
5730599A22RIK	ILMN_1212946	0.4472623	0.9680857	0.0428562	0.410001	0.0125654	0.0265242	5.674163333	5.691197368	5.674958824	5.620590909					
**UP IN GA AND REF ONLY**																
Gene	ID	Medium vs. Reference Standard pval	Medium vs. GA pval	Medium vs generic pval	Reference Standard vs GA pval	Reference Standard vs generic pval	GA vs. generic pval	AVG Medium	AVG Reference Standard	AVG GA	AVG generic					
C130079K02RIK	ILMN_2568571	1.041E-12	1.162E-10	0.1281005	0.7257423	9.988E-07	2.012E-05	7.22574	7.544463158	7.531626471	7.306454545					
ALS2CL	ILMN_1240592	1.675E-05	7.071E-06	0.2529273	0.9728509	0.0108725	0.0080487	7.116023333	7.394581579	7.392775	7.205686364					
2810410P22RIK	ILMN_2692986	1.128E-08	1.338E-09	0.0803272	0.3464283	9.052E-05	0.0001647	7.714383333	8.011573684	7.979019118	7.8124		Pattern Represented by Data:			
HNRNPK	ILMN_1245987	4.289E-05	0.0002067	0.1413562	0.6256313	0.0041426	0.0421137	9.7988	10.07715	10.05100735	9.913459091					
TRAT1	ILMN_2652867	1.048E-09	5.461E-10	0.6941578	0.5000227	1.272E-07	3.259E-08	7.813176667	8.078084211	8.055941176	7.793545455			x	x	
LSG1	ILMN_1248340	2.039E-05	7.602E-07	0.8259239	0.634177	0.0001259	1.579E-05	7.035183333	7.257968421	7.277522059	7.047777273		x			x
RAB6	ILMN_1242802	0.0001967	7.59E-09	0.0753563	0.3691737	0.0440001	0.0003189	7.261916667	7.466060526	7.498622059	7.357177273		Medium	Reference	GA	generic
ARL5A	ILMN_1221102	9.169E-07	2.131E-06	0.139763	0.1192096	0.000692	0.006183	7.797843333	8.091231579	8.028401471	7.893445455					
OTTMUSG0000000	ILMN_2847773	3.794E-06	7.556E-08	0.516434	0.819258	2.055E-05	4.112E-06	6.737806667	6.952839474	6.959511765	6.774009091					
RN18S	ILMN_2638923	3.929E-05	0.000642	0.4398246	0.091513	4.139E-05	0.0079489	9.738126667	10.04179474	9.958561765	9.800913636					
A830083H19RIK	ILMN_2565089	3.773E-08	4.608E-07	0.323377	0.1306119	3.816E-07	5.705E-05	7.416416667	7.6885	7.635472059	7.464036364					
FYB	ILMN_1236105	6.783E-06	3.137E-05	0.4465201	0.8846087	0.0004118	0.0024686	8.031866667	8.256457895	8.250267647	8.0774					

PUM2	ILMN_2607160	1.834E-07	1.03E-10	0.3654261	0.5669367	7.737E-05	1.208E-06	8.49848	8.733373684	8.716505882	8.539704545					
SCL0002368.1_75	ILMN_1214650	0.0061628	0.0005403	0.8018885	0.6002032	4.876E-05	2.217E-05	8.83479	9.028768421	9.052716176	8.814618182					
COX7A1	ILMN_1240857	0.0003356	4.324E-08	0.3145874	0.0611814	0.0056518	1.055E-05	6.94444	7.100284211	7.161101471	6.989281818					
NPC2	ILMN_2744380	2.733E-06	6.581E-08	0.0829615	0.660283	0.0065729	0.0018198	9.368056667	9.561731579	9.575010294	9.4518					
SAMHD1	ILMN_1221736	1.42E-06	6.451E-07	0.1235508	0.3860393	0.0001021	0.001305	11.84208667	12.07217895	12.04837794	11.93060909					
CLCN3	ILMN_1221919	1.302E-06	1.969E-06	0.1671821	0.9353394	0.000792	0.0029833	8.224926667	8.427963158	8.430619118	8.2973					
PTPN1	ILMN_1222917	3.577E-07	1.066E-05	0.2605158	0.0980524	0.0001364	0.0036276	9.086276667	9.362913158	9.291763235	9.143836364					
CBFB	ILMN_2671644	4.874E-05	9.419E-07	0.0925495	0.5793726	0.0099041	0.0023062	6.44466	6.629710526	6.648410294	6.525068182					
5830457O10RIK	ILMN_1249269	0.0051915	0.0001913	0.3258661	0.6242513	0.0391047	0.0065255	6.626373333	6.803589474	6.824932353	6.691472727					
HIST1H2AG	ILMN_2981801	0.0014204	4.276E-06	0.5091729	0.1896142	0.0106585	0.0002015	9.87061	10.02252105	10.067625	9.906631818					
LOC383706	ILMN_2535779	0.0002793	1.68E-06	0.1930403	0.2544232	0.0124523	0.0007026	8.164186667	8.320342105	8.360444118	8.219022727					
TPM4	ILMN_1223539	6.066E-06	2.605E-05	0.0918537	0.135282	0.0023364	0.0357186	7.28479	7.536536842	7.481042647	7.387468182					
CYB5	ILMN_2775098	1.34E-08	6.241E-13	0.1162492	0.1205256	0.0001373	3.917E-07	11.01746667	11.17962895	11.21147353	11.06824091					
TOP2A	ILMN_1255436	7.891E-10	1.572E-08	0.0936923	0.2373449	3.871E-05	0.0009104	6.89295	7.115813158	7.084679412	6.964609091					
DCUN1D5	ILMN_1238285	4.199E-06	1.874E-06	0.3808523	0.3750355	0.0001445	0.0002891	9.349493333	9.563021053	9.534638235	9.392522727					
PPP1CB	ILMN_2647628	1.281E-05	1.161E-05	0.1588769	0.1818483	0.0045437	0.0174578	7.532633333	7.763778947	7.717295588	7.616245455					
PJA1	ILMN_2516312	4.754E-05	5.509E-05	0.1330899	0.4609369	0.0082031	0.0301125	6.55579	6.763297368	6.736463235	6.640472727					
CD247	ILMN_1256639	9.093E-07	6.486E-10	0.3133538	0.6696727	0.0016499	3.792E-05	7.05133	7.215657895	7.226701471	7.088009091					
RNF8	ILMN_1214110	0.0001013	2.758E-05	0.1329161	0.8730459	0.0160342	0.019529	6.676843333	6.857478947	6.852086765	6.755077273					
TRMT2A	ILMN_2682887	0.0034073	0.0002859	0.4813778	0.7996107	0.0204651	0.0056935	6.52321	6.684289474	6.694272059	6.565359091					
ARL1	ILMN_1219577	2.543E-06	5.309E-06	0.0628551	0.252179	0.001757	0.0180858	9.743456667	9.9474	9.913035294	9.828659091					
4930539E08RIK	ILMN_1238069	3.208E-08	9.794E-09	0.4673532	0.7903331	4.063E-05	2.167E-05	6.088663333	6.262042105	6.255341176	6.113204545					
CXCR6	ILMN_1221846	0.0001942	3.581E-05	0.3671141	0.8541377	0.0084059	0.0045185	6.669376667	6.827515789	6.833716176	6.712486364					
TMPO	ILMN_2471359	0.0001538	1.421E-06	0.1449316	0.8139868	0.0179909	0.0026101	8.46599	8.623305263	8.629933824	8.530504545					
IRF1	ILMN_2649068	0.0182535	0.008745	0.9479639	0.9043042	0.0048358	0.0058889	10.31889	10.48616842	10.48073382	10.32447727					
ACTN4	ILMN_1242390	0.0043415	0.0010632	0.9291751	0.9191823	0.0073692	0.0031055	6.448333333	6.604534211	6.608894118	6.453640909					
WDR45L	ILMN_1242852	0.0001397	1.014E-05	0.4396148	0.8937027	0.0072572	0.0019591	7.205136667	7.360331579	7.364545588	7.240713636					
ZFP281	ILMN_2477243	1.384E-06	4.322E-06	0.3188726	0.1898452	0.0001716	0.0018034	8.814456667	9.007431579	8.973852941	8.863868182					
D6WSU163E	ILMN_1224433	1.163E-07	2.49E-06	0.0702723	0.1647996	0.0020446	0.0255861	6.423556667	6.621960526	6.581777941	6.499395455					
NUDCD2	ILMN_2751228	0.0021746	0.0023554	0.4881067	0.5065572	0.0233342	0.0387743	6.944946667	7.134152632	7.101964706	6.987381818					
H2-T23	ILMN_1223179	0.0048276	0.0004926	0.385449	0.6647609	0.023522	0.0104316	10.62381	10.76490263	10.77933382	10.67458636					
LOC667005	ILMN_1217929	0.0009143	0.0005654	0.7436009	0.7376516	0.0008964	0.0006125	6.864946667	7.033302632	7.019588235	6.847404545					
EIF3B	ILMN_1233746	0.0007598	0.0001797	0.3382327	0.8693315	0.0125905	0.0099399	6.22973	6.388731579	6.382969118	6.276936364					
ELAC2	ILMN_1255921	0.0013267	0.0001292	0.6032269	0.9503948	0.008666	0.0023259	6.199506667	6.348668421	6.350842647	6.22455					
GTL7	ILMN_2753314	3.067E-06	4.55E-06	0.2576346	0.165215	8.345E-05	0.0006453	7.284363333	7.47345	7.434189706	7.326722727					
LOC100046320	ILMN_1243283	0.0006338	0.0007498	0.2955672	0.4137896	0.0153852	0.0360649	7.5542	7.737257895	7.703685294	7.607563636					
PARP14	ILMN_2825803	9.76E-07	3.908E-06	0.3720343	0.4890092	9.779E-05	0.0009547	10.98116	11.14566053	11.12864853	11.01771364					
PK3	ILMN_1236323	5.828E-05	1.915E-06	0.1683357	0.7021492	0.0291329	0.0074242	6.16902	6.306352632	6.316370588	6.225122727					
ZZZ3	ILMN_2432040	0.0014965	5.402E-06	0.378144	0.2315133	0.04634	0.0017282	6.03378	6.146386842	6.180801471	6.068531818					
LOC100041450	ILMN_1242660	0.0001568	1.45E-06	0.3931224	0.2140731	0.0049911	0.0002981	6.805983333	6.921857895	6.952891176	6.835990909					
WAPAL	ILMN_1242714	3.611E-05	3.018E-05	0.1528919	0.672663	0.0095934	0.0219708	6.27658	6.434936842	6.423070588	6.340836364					
8030402P03RIK	ILMN_2585137	0.0022227	7.373E-05	0.5863732	0.4333824	0.0221549	0.0025129	7.633246667	7.750686842	7.775892647	7.655845455					
A630098G22RIK	ILMN_2582171	0.0002997	0.0001568	0.1752775	0.4269924	0.0214848	0.0371472	6.98317	7.150939474	7.12385	7.044545455					
CSF1	ILMN_1254561	1.52E-07	5.768E-06	0.1116898	0.107214	0.0007031	0.0130531	6.24299	6.430692105	6.383498529	6.296836364					
FBXO33	ILMN_3162369	0.0001389	5.808E-07	0.3215115	0.5062504	0.0167325	0.000955	8.757316667	8.881023684	8.897601471	8.793440909					
SLCO3A1	ILMN_1235635	0.0009188	0.000204	0.7309941	0.9859045	0.0049286	0.0024582	6.011396667	6.150071053	6.150582353	6.029604545					
PDLIM1	ILMN_1234072	0.0044138	3.993E-05	0.8543867	0.2320581	0.004056	7.263E-05	9.389206667	9.494944737	9.528047059	9.380759091					
2810008M24RIK	ILMN_2486600	0.0001974	8.115E-06	0.3528546	0.8370992	0.0037293	0.0008125	9.577263333	9.719655263	9.714169118	9.612672727					
1700025G04RIK	ILMN_2669982	7.521E-06	1.338E-05	0.1133944	0.3562127	0.0011588	0.0116783	7.13209	7.290965789	7.268439706	7.1935					
BCL11B	ILMN_2443624	0.0015187	0.0018521	0.07205	0.6258324	4.592E-06	2.444E-06	7.475326667	7.627081579	7.608722059	7.374281818					
D630022O22RIK	ILMN_1223311	2.867E-05	3.487E-05	0.3400248	0.8660441	0.0030361	0.0061045	6.197413333	6.334594737	6.330180882	6.235186364					
DMBT1	ILMN_2629239	2.525E-05	9.868E-06	0.1748778	0.8787925	0.0284553	0.0220519	5.65465	5.791228947	5.787077941	5.706268182					
MAD2L1BP	ILMN_2815222	0.0012164	1.37E-06	0.4454937	0.2891597	0.0074512	4.415E-05	9.83867	9.946563158	9.970585294	9.864663636					
IRF1	ILMN_2649067	0.0389177	0.0268373	0.9283349	0.6734148	0.0111936	0.0153172	10.39029333	10.53875526	10.52146618	10.39822727					
ETS1	ILMN_2770739	0.0014202	0.003545	0.7381796	0.3882843	0.0040934	0.0168068	7.680986667	7.843268421	7.811420588	7.700831818					
1810013L24RIK	ILMN_1260569	2.497E-08	2.603E-06	0.1212941	0.1254551	0.0001023	0.0081654	7.528863333	7.691768421	7.658367647	7.581836364					
TOR1A	ILMN_2631144	0.0007978	4.721E-05	0.2655705	0.5500238	0.0416582	0.0108028	7.383143333	7.495473684	7.511541176	7.425604545					
TRAT1	ILMN_1229005	0.0031667	0.0002999	0.6193732	0.7273838	0.0027538	0.000195	6.647076667	6.785913158	6.773917647	6.622813636					
PABPC1	ILMN_1259769	0.0022435	0.0104623	0.9375031	0.1698843	0.0019564	0.0137295	6.85638	7.040826316	6.981691176	6.861254545					
H2AFZ	ILMN_3074985	0.0004716	0.0001041	0.1178648	0.8869298	0.0499257	0.0416554	11.80579667	11.93475	11.93059412	11.86225909					

ZDHHC21	ILMN_2780619	0.0066739	0.0003336	0.7907514	0.4972362	0.0196609	0.0024035	8.437696667	8.5418	8.562170588	8.449168182					
ZNF24	ILMN_2518828	0.021391	0.000404	0.6021228	0.1239823	0.004883	0.000186	6.048016667	6.124986842	6.171314706	6.028518182					
ZWILCH	ILMN_1247268	0.0005599	0.0006099	0.305414	0.6301429	0.0312265	0.0462173	6.168553333	6.305978947	6.289795588	6.210068182					
AP1B1	ILMN_1244906	4.353E-05	2.938E-06	0.1251398	0.8087134	0.023292	0.0132339	10.82644667	10.95238421	10.94691618	10.87835					
2500002A22RIK	ILMN_1255902	9.823E-05	0.0012723	0.7473814	0.3107468	0.0008764	0.0105752	6.55427	6.705039474	6.673707353	6.569454545					
LOC380707	ILMN_1252450	0.0012425	0.0017786	0.9635044	0.7942431	0.0014543	0.0034494	9.90633	10.03282105	10.02428529	9.90435					
TNP2	ILMN_1224330	7.377E-05	0.0001617	0.330308	0.2329272	0.0044444	0.0192587	5.844083333	5.988826316	5.958627941	5.884845455					
GTF3C5	ILMN_1222243	0.0059505	0.0001115	0.6279649	0.404636	0.0404102	0.0029389	5.814863333	5.905071053	5.927685294	5.83125					
HIST1H2AK	ILMN_2847144	0.0019193	0.0051082	0.8806175	0.4091838	0.0026227	0.0069281	12.67005667	12.80842105	12.78147206	12.66135909					
RNGTT	ILMN_2750669	0.0128245	0.0023161	0.9298071	0.7426152	0.0166158	0.0052134	6.190413333	6.291310526	6.301817647	6.194254545					
VPS37B	ILMN_2681670	0.0074366	0.0017717	0.2717552	0.9367849	8.964E-05	1.742E-05	10.44526333	10.55398421	10.55639412	10.39754091					
F830048D03RIK	ILMN_2584985	0.0357552	0.0017199	0.6376489	0.3937319	0.0150219	0.0007896	6.689073333	6.771521053	6.799188235	6.670236364					
USF1	ILMN_2435505	0.0138797	0.009899	0.6581491	0.8509838	0.0412597	0.0471047	6.804973333	6.921971053	6.914854412	6.826927273					
HEATR3	ILMN_2590644	0.0080322	0.0003283	0.7917175	0.6138605	0.0146601	0.0009865	5.744316667	5.838378947	5.853311765	5.734418182					
STIL	ILMN_2654075	0.0362002	0.0003313	0.8132537	0.134671	0.0407928	0.0008118	5.61879	5.685102632	5.726732353	5.610486364					
FEM1C	ILMN_2724409	0.0003443	6.939E-05	0.2502713	0.5010987	0.011721	0.0113217	7.96895	8.091639474	8.076245588	8.010295455					
EG666756	ILMN_1214902	9.451E-05	0.0003847	0.4631473	0.2674416	0.0047458	0.0182487	10.67756	10.81662632	10.78483529	10.70416364					
PIBF1	ILMN_3115640	5.559E-05	0.0003718	0.2499334	0.1444557	0.0044791	0.0448955	6.849893333	6.993676316	6.956576471	6.895045455					
MX2	ILMN_1239219	0.0068612	0.0108891	0.8598659	0.6741755	0.0047833	0.0106972	7.97713	8.098521053	8.082936765	7.968677273					
CHIC2	ILMN_2548974	0.0004283	0.0001532	0.260031	0.5502436	0.0254452	0.0295837	8.378606667	8.498828947	8.484154412	8.42045					
EIF4E3	ILMN_1254878	6.557E-05	0.0025094	0.6206909	0.0708281	2.875E-06	0.0004164	8.776916667	8.931847368	8.881707353	8.754254545					
RPL37	ILMN_1236446	0.0002543	0.0003352	0.2816868	0.5589165	0.0102113	0.0240646	5.6613	5.780355263	5.765086765	5.696131818					
ELF1	ILMN_2659380	0.0148489	0.0011844	0.7138757	0.9039232	0.0069592	0.0003911	10.33958333	10.43980263	10.44328824	10.32341818					
BAT2D	ILMN_1221890	0.0015367	0.0001683	0.8307622	0.5760588	0.0106124	0.0026079	5.830226667	5.947547368	5.932069118	5.837931818					
4930404F20RIK	ILMN_1222969	0.0360605	0.0001718	0.7431989	0.1391011	0.0161718	7.394E-05	6.658073333	6.724336842	6.759348529	6.646877273					
LOC100044190	ILMN_1214731	2.746E-05	0.0018164	0.988103	0.0603332	0.0002041	0.006513	10.56954667	10.72565263	10.67076618	10.57017273					
EPSTI1	ILMN_2707865	0.0071416	0.0014801	0.5480541	0.6847784	0.0363948	0.0198692	8.180176667	8.271594737	8.281332353	8.206504545					
GPI1	ILMN_2692845	3.304E-06	0.000112	0.1541174	0.1930921	0.0029369	0.0470385	11.68305667	11.81179474	11.7829	11.72728182					
4930422G04RIK	ILMN_1243091	8.401E-05	6.98E-05	0.1535267	0.7726701	0.0350024	0.0486999	5.690713333	5.795713158	5.789577941	5.735836364					
RBM14	ILMN_1245292	0.0018364	0.0019379	0.6891648	0.4886926	0.0280755	0.033881	6.059196667	6.178389474	6.157111765	6.07645					
4932413O14RIK	ILMN_2620757	0.0127067	0.0007843	0.9954839	0.4908601	0.0216546	0.0024511	5.634773333	5.714357895	5.731648529	5.634990909					
CD247	ILMN_2828172	0.0095874	0.0020148	0.4593334	0.7912747	0.0055389	0.0007173	6.427023333	6.531918421	6.523489706	6.396290909					
CCR4	ILMN_2753620	0.0017762	0.0006187	0.4406932	0.7358693	0.0181035	0.0161563	5.72549	5.829228947	5.821095588	5.752877273					
ZFP800	ILMN_2760387	0.0448081	0.0075997	0.9676854	0.6623265	0.0439818	0.0101354	6.687236667	6.769447368	6.782586765	6.685263636					
PABPC1	ILMN_1259482	0.0051572	0.0138815	0.6552533	0.3254165	0.0027309	0.006147	11.55015	11.68038684	11.64491176	11.52837273					
D230021E06RIK	ILMN_2569706	0.0001027	0.0107031	0.9348866	0.0663329	0.0001087	0.0197819	7.510763333	7.665360526	7.605245588	7.514104545					
SELP	ILMN_1236889	0.0140687	0.0058444	0.9379626	0.9259121	0.0105505	0.0086371	6.475836667	6.566694737	6.569254412	6.479186364					
TSPAN14	ILMN_1257666	0.0006106	0.0021634	0.9918866	0.6000334	0.0012631	0.0057197	9.755656667	9.861615789	9.848317647	9.756059091					
V1RD18	ILMN_2996714	0.0009999	0.0004902	0.3718426	0.6093331	0.0390579	0.0399917	5.599066667	5.703860526	5.691057353	5.629445455					
CD226	ILMN_1239753	0.0261779	0.0085975	0.6790065	0.9259328	0.0193439	0.005597	6.903246667	6.998228947	6.995114706	6.884318182					
Mgeneric	ILMN_2600747	0.0031996	0.0012523	0.5857151	0.921512	0.0155614	0.0137085	6.73558	6.829271053	6.826967647	6.755563636					
2310016C16RIK	ILMN_2625854	3.934E-05	0.0016327	0.4362885	0.211481	0.0011679	0.0354731	5.732096667	5.854347368	5.823288235	5.757786364					
DCK	ILMN_1227664	0.0051761	0.0184859	0.3369228	0.393242	0.0004955	0.0017168	6.626113333	6.746452632	6.716342647	6.582109091					
PCDHGC5	ILMN_1216284	0.0121025	0.0028888	0.982265	0.8269325	0.0154234	0.005817	5.98469	6.068434211	6.074589706	5.985395455					
UBE2Z	ILMN_2774701	0.0042651	0.0035787	0.8977551	0.802039	0.022089	0.0197221	6.080933333	6.175294737	6.168729412	6.086440909					
PLXNC1	ILMN_2756855	0.0132133	0.0099808	0.7888317	0.8652899	0.043813	0.0427865	6.419863333	6.511336842	6.506238235	6.43105					
PSEN2	ILMN_1213684	0.0189562	0.0056686	0.8398917	0.9864612	0.0430124	0.0193465	5.908983333	5.994313158	5.994827941	5.915718182					
STAT3	ILMN_2698046	0.0009534	0.0115729	0.8152262	0.3037877	0.0034476	0.0393006	10.98784	11.10429737	11.07339706	10.99688182					
LOC628101	ILMN_1213707	0.004533	0.0111088	0.6456473	0.4943089	0.0047831	0.0087798	6.481883333	6.588642105	6.566361765	6.46445					
GREM2	ILMN_2610565	0.0034682	0.0112391	0.9597837	0.5615038	0.0114491	0.0312014	5.9519	6.052036842	6.035844118	5.954177273					
INPP4B	ILMN_2854354	0.0001211	0.0045817	0.8300829	0.214436	0.0007914	0.0208243	6.231216667	6.344565789	6.313639706	6.238772727					
NEO1	ILMN_2579266	0.0118953	0.0023659	0.7127533	0.7455244	0.003547	0.0009012	6.07765	6.151984211	6.158933824	6.063904545					
1700027N10RIK	ILMN_2775220	0.0087824	0.0009903	0.9594418	0.7293236	0.0077204	0.0015392	6.36718	6.440963158	6.448286765	6.3687					
ZFP277	ILMN_3123889	0.04469	0.0040836	0.399287	0.4562136	0.0060016	0.0004105	9.87083	9.932757895	9.951282353	9.841213636					
GNL3L	ILMN_2628122	0.0042936	0.0168829	0.8533817	0.3932727	0.0079319	0.0410368	7.609203333	7.714147368	7.689342647	7.616795455					
LOC100047009	ILMN_1246359	0.0016461	0.0045336	0.6158285	0.5219803	0.0005793	0.0021157	5.947723333	6.043807895	6.027170588	5.933					
PPP2R5C	ILMN_3121851	0.0121003	0.0259879	0.9530225	0.3251563	0.0068232	0.02552	9.96416	10.07191842	10.04338529	9.967009091					
LOC235279	ILMN_1231838	0.0022376	0.0005205	0.8114829	0.9149712	0.0151046	0.0059357	5.738703333	5.820126316	5.817807353	5.745463636					
PLEKHF2	ILMN_1237736	0.0035081	0.0109001	0.9737767	0.2710677	0.0014493	0.0102796	8.16861	8.274002632	8.246895588	8.169977273					
IL1F10	ILMN_2776687	0.0267869	0.0137905	0.9852541	0.8846595	0.0495733	0.0296692	5.98729	6.069436842	6.065126471	5.986545455					

3202002H23RIK	ILMN_1236031	0.012129	0.0092909	0.483435	0.6661905	0.0015716	0.0009907	6.4492	6.538394737	6.526935294	6.422209091					
LOC381093	ILMN_2530674	0.0037258	0.0023124	0.5246847	0.9676352	0.0023089	0.0013058	5.809203333	5.883715789	5.884635294	5.790786364					
KRT25	ILMN_1216096	0.0135579	0.0136597	0.8793285	0.6350166	0.0416839	0.0461866	5.72627	5.813852632	5.800451471	5.73225					
VMN2R81	ILMN_2628699	0.0233293	0.0022967	0.9367462	0.5493774	0.0345394	0.0052882	5.918906667	5.979321053	5.992120588	5.916390909					
FERD3L	ILMN_2724181	0.0328662	0.0320617	0.7059836	0.7516483	0.0172619	0.017198	5.81418	5.896818421	5.887038235	5.798140909					
TELO2	ILMN_2734797	0.0108678	0.0410319	0.5554944	0.4051911	0.0032397	0.0126048	5.800226667	5.898847368	5.872701471	5.773190909					
9430077D24RIK	ILMN_2524699	0.0031698	0.0424885	0.6312243	0.121102	0.0001607	0.0086891	7.0186	7.135728947	7.091011765	6.99615					
SLC30A8	ILMN_2686369	0.0253593	0.0118829	0.8033146	0.706046	0.0185698	0.0114584	5.580283333	5.643439474	5.652214706	5.570672727					
TRAPPC2	ILMN_2438032	0.0128277	0.0167637	0.9903469	0.7905655	0.016867	0.0288908	5.7618	5.839247368	5.832552941	5.76225					
C920025C15RIK	ILMN_1242664	0.025157	0.041818	0.1248153	0.5543993	0.0009658	0.0008659	6.85869	6.949318421	6.929292647	6.796427273					
KLRA1	ILMN_1228875	0.0210432	0.0152911	0.9356955	0.6111696	0.0257936	0.0195506	5.763913333	5.846684211	5.833513235	5.760622727					
EG386506	ILMN_1225532	0.0443727	0.0195343	0.7357978	0.7211962	0.0336209	0.017162	5.675066667	5.7339	5.743238235	5.663213636					
LOC383306	ILMN_1235357	0.000646	0.0212078	0.9585536	0.0838609	0.0013532	0.0400109	5.85391	5.963960526	5.920935294	5.8559					
VMN2R89	ILMN_3151786	0.0333377	0.0437252	0.8447629	0.5683777	0.0304101	0.0386624	6.176576667	6.259428947	6.242405882	6.168013636					
LOC244219	ILMN_2525856	0.0425273	0.0175024	0.5375149	0.8250013	0.0148012	0.0057212	5.892636667	5.952907895	5.958372059	5.870513636					
2610017G09RIK	ILMN_1226459	0.0462486	0.0299381	0.9979677	0.9783931	0.0380499	0.0356214	5.774383333	5.840123684	5.839426471	5.774468182					
LOC386161	ILMN_1257961	0.0100486	0.0131679	0.8190764	0.8131695	0.0322735	0.0481536	5.588033333	5.657231579	5.651886765	5.595286364					
NCDN	ILMN_2664579	0.0342661	0.016931	0.9295063	0.9931828	0.0498308	0.0283885	5.806293333	5.869863158	5.869651471	5.803568182					
SLFN9	ILMN_2688948	0.0325834	0.0292092	0.8362985	0.6502896	0.0335642	0.0471183	5.536233333	5.60855	5.597972059	5.544027273					
LOC331243	ILMN_1253944	0.0010507	0.0069681	0.9739024	0.5800158	0.0053713	0.0203677	5.580336667	5.652857895	5.641372059	5.5795					
RAB33A	ILMN_3161828	0.0416086	0.0465199	0.2932489	0.9090289	0.0047756	0.0042555	6.031763333	6.095615789	6.092541176	5.987954545					
DTYMK	ILMN_2485787	0.0031272	0.0030314	0.7272849	0.5458658	0.013986	0.0194651	10.10785	10.17909737	10.16820147	10.11717727					
LOC100040244	ILMN_1260579	0.0088602	0.020958	0.5320051	0.3202183	0.0050246	0.0067198	7.19639	7.280847368	7.256533824	7.172322727					
OS9	ILMN_1246758	0.001885	0.0232342	0.953676	0.0875945	0.0014605	0.0229501	9.561276667	9.660952632	9.621010294	9.559304545					
D630011N09RIK	ILMN_2580554	0.0458818	0.0133375	0.7643319	0.8662868	0.0308974	0.0091603	5.565416667	5.621328947	5.624910294	5.555445455					
A930014D08RIK	ILMN_2519658	0.0486271	0.0149848	0.7406188	0.7386258	0.0170917	0.0065284	5.644493333	5.696589474	5.703229412	5.633840909					
MYNN	ILMN_1213772	0.005896	0.0112655	0.7696081	0.4883202	0.0167039	0.0408157	5.760286667	5.832828947	5.818648529	5.768645455					
SPNB2	ILMN_2681429	0.0326787	0.0349451	0.494234	0.9290664	0.0183645	0.0165316	5.695196667	5.750415789	5.752666176	5.672931818					
RNF19A	ILMN_2424138	0.0259662	0.0486041	0.5380445	0.2509639	0.0039359	0.0041805	5.905073333	5.992334211	5.962323529	5.879622727					
ARHGAP25	ILMN_3155245	0.0295298	0.0173066	0.6025104	0.3814893	0.0219848	0.0069411	10.10459	10.18453947	10.16155	10.08619091					
LOC383433	ILMN_2539526	0.0047365	0.0187802	0.9159006	0.6490296	0.0237397	0.0465046	5.61099	5.677026316	5.666641176	5.608054545					
MIER2	ILMN_2671319	0.0361367	0.023984	0.4798676	0.8055656	0.0058249	0.0029736	5.829166667	5.889905263	5.884575	5.805927273					
PDIA2	ILMN_1215125	0.0186685	0.0423804	0.7095586	0.4244174	0.0202628	0.0335745	5.79263	5.866986842	5.846214706	5.779913636					
LOC384990	ILMN_1218114	0.011363	0.0354083	0.8320832	0.4519395	0.003319	0.02169	5.856243333	5.924778947	5.908864706	5.849868182					
LOC100043391	ILMN_2740432	0.0095943	0.0466303	0.4380002	0.3030988	0.0011218	0.0060576	14.24296667	14.31736842	14.29466324	14.21495					
LOC100040854	ILMN_1217820	0.0326164	0.0323291	0.2727063	0.8371501	0.0029163	0.0050089	5.568043333	5.614126316	5.618561765	5.542736364					
PIGN	ILMN_2547482	0.0364563	0.0260185	0.5514131	0.6893077	0.0133012	0.0065321	5.77965	5.838668421	5.830154412	5.7613					
BAZ1B	ILMN_1246235	0.0140452	0.0213284	0.8925962	0.4231022	0.0251839	0.0323462	5.625716667	5.692768421	5.676020588	5.621545455					
TTLL4	ILMN_1251995	0.0092136	0.0435202	0.7539064	0.304508	0.0088891	0.0333676	5.922753333	5.996405263	5.973054412	5.912381818					
DHX32	ILMN_2643150	0.008995	0.0495151	0.908182	0.1918829	0.0093641	0.0494344	8.92933	9.010481579	8.979463235	8.9256					
D4ERTD22E	ILMN_3129206	0.0019254	0.0170709	0.9452562	0.2197383	0.0067388	0.0350192	11.32444333	11.39864211	11.37389118	11.32272727					
LOC100048816	ILMN_1253242	0.0063968	0.045496	0.7887721	0.1342978	0.0118965	0.047928	5.880436667	5.965505263	5.928376471	5.871918182					
GPC3	ILMN_1223663	0.0124212	0.0402583	0.8858477	0.4679769	0.0112841	0.0427004	5.648313333	5.711607895	5.695651471	5.644954545					
LOC384585	ILMN_1243863	0.0045598	0.0345773	0.8653368	0.2046214	0.0064519	0.0377219	5.716866667	5.787997368	5.764117647	5.711304545					
OLFR701	ILMN_2652694	0.0185923	0.0489834	0.7172779	0.2030837	0.0160646	0.0278697	5.69437	5.76715	5.738292647	5.683054545					
FANCA	ILMN_1220189	0.0186167	0.0465428	0.8057021	0.3372639	0.0143651	0.0332572	5.56208	5.625584211	5.605772059	5.55515					
**DOWN IN GA AND REF ONLY**																
Gene	ID	Medium vs. Reference Standard pval	Medium vs. GA pval	Medium vs generic pval	Reference Standard vs GA pval	Reference Standard vs generic pval	GA vs. generic pval	AVG Medium	AVG Reference Standard	AVG GA	AVG generic					
LOC386360	ILMN_2538531	0.0018127	1.306E-06	0.2417974	0.0610866	0.0476618	0.000511	9.76067	9.543934211	9.445438235	9.669977273					
CLEC4E	ILMN_1216972	2.388E-08	1.799E-08	0.2913318	0.0720895	0.0001058	0.0003322	7.003983333	6.697260526	6.759897059	6.9338					
LOC385923	ILMN_1230140	0.0023267	9.85E-06	0.8804496	0.3573459	0.0011591	5.702E-06	7.89017	7.690592105	7.650747059	7.901590909		Pattern Represented by Data:			
CSF3R	ILMN_2806180	2.719E-06	2.468E-08	0.19436	0.7656032	0.0026131	0.0003455	6.042593333	5.823492105	5.813885294	5.969613636					
CD33	ILMN_2675223	2.257E-09	2.981E-09	0.3333232	0.1662516	1.498E-05	4.529E-05	6.606853333	6.339052632	6.381583824	6.556072727		x			x
CD63	ILMN_3128907	9.578E-05	4.55E-06	0.4543748	0.9659952	0.0029774	0.0006786	7.70484	7.482515789	7.484204412	7.655954545			x	x	

CXCR4	ILMN_2630459	4.166E-07	2.333E-05	0.4582274	0.1836911	0.0003531	0.0051366	9.36576	9.097465789	9.153819118	9.316654545		Medium	Reference	GA	generic
AA536749	ILMN_1219077	3.09E-09	1.346E-08	0.0549268	0.8219967	0.0002312	0.0010926	8.959366667	8.7437	8.750286765	8.878977273					
LOC386268	ILMN_1222988	0.0190164	8.09E-05	0.6845559	0.2041966	0.0323632	0.0001997	6.859343333	6.705134211	6.655375	6.828495455					
genericLS	ILMN_1250635	3.881E-05	9.224E-09	0.2094099	0.1806939	0.0422933	0.0008516	6.407056667	6.252378947	6.211517647	6.348690909					
GAS7	ILMN_2776850	2.885E-06	1.144E-06	0.5756013	0.9274013	3.971E-05	7.879E-05	6.87571	6.687760526	6.685158824	6.846809091					
KLC4	ILMN_1251301	3.915E-08	6.955E-10	0.1139408	0.8462085	0.0019125	0.0005706	7.976776667	7.781668421	7.786357353	7.905563636					
LOC386288	ILMN_1257784	0.0001885	2.933E-05	0.1046022	0.9683232	0.0425433	0.0459418	7.19565	7.004444737	7.005695588	7.089990909					
TMEM38B	ILMN_2795791	7.322E-07	5.939E-09	0.1176012	0.6402061	0.0038277	0.0004492	8.486633333	8.312155263	8.299532353	8.423818182					
GPR97	ILMN_2666492	1.03E-05	1.079E-06	0.2142425	0.6849131	0.0042059	0.0017393	6.329983333	6.156044737	6.1434	6.272113636					
1100001G20RIK	ILMN_2651743	7.743E-06	9.245E-06	0.2289623	0.5387134	0.0009023	0.003214	6.425286667	6.217157895	6.238989706	6.366827273					
APP	ILMN_2597532	1.738E-06	1.913E-07	0.0563277	0.7760063	0.0043673	0.0041235	7.13296	6.940878947	6.949335294	7.052945455					
4930572J05RIK	ILMN_3065373	0.004566	2.51E-05	0.4441944	0.5165798	0.0407129	0.0014263	10.59237333	10.43747105	10.41293676	10.54924091					
TGIF1	ILMN_1253854	0.0008531	0.0001751	0.5099102	0.8326892	0.0121542	0.006438	8.40255	8.232644737	8.224369118	8.362318182					
LOC243025	ILMN_1252253	2.074E-06	5.102E-08	0.0659736	0.738499	0.0069137	0.002064	6.799403333	6.631713158	6.622926471	6.726436364					
SEC16A	ILMN_1225772	0.0001219	2.523E-08	0.0500137	0.2232897	0.0485284	0.0008313	10.14415	10.00321316	9.968227941	10.07729545					
BC014795	ILMN_2769945	1.451E-06	1.478E-07	0.3549454	0.9218641	0.0006014	0.0002032	8.22279	8.047292105	8.050194118	8.188168182					
CFB	ILMN_1230853	6.194E-05	0.0001335	0.2759896	0.2823461	0.0011429	0.0118492	6.413743333	6.207484211	6.243664706	6.349068182					
TMEM63A	ILMN_2798340	0.0001154	4.004E-06	0.0785413	0.3188699	0.0288569	0.0052776	7.836176667	7.698636842	7.666680882	7.774609091					
DNMT3L	ILMN_1250149	0.000214	7.511E-08	0.4818082	0.1380166	0.0117734	8.102E-05	6.21078	6.081707895	6.041905882	6.181622727					
ACBD4	ILMN_3150296	0.000222	1.276E-06	0.1850789	0.3888446	0.0273003	0.0020199	9.321576667	9.179684211	9.154017647	9.266631818					
ORF19	ILMN_3158633	5.139E-07	1.061E-06	0.0627275	0.2847864	0.0024954	0.0154805	7.046476667	6.849634211	6.879329412	6.964309091					
CREG1	ILMN_1239638	8.391E-06	1.506E-07	0.3754579	0.901707	0.0011917	0.0001639	6.78207	6.618634211	6.615505882	6.743304545					
IRAK3	ILMN_1253972	0.0001809	8.622E-05	0.253004	0.7292535	0.0166538	0.013528	9.18481	9.031655263	9.019070588	9.132704545					
BC065397	ILMN_2869654	5.829E-06	2.907E-06	0.0546624	0.8070999	0.0116688	0.0182155	6.624853333	6.452586842	6.459883824	6.546759091					
ARHGAP22	ILMN_2672380	8.766E-06	5.386E-07	0.0546356	0.2706834	0.0222117	0.0325518	6.35587	6.161063158	6.192420588	6.264263636					
GAA	ILMN_1254199	1.191E-06	3.867E-07	0.1415128	0.7057601	0.0094359	0.0087814	8.179186667	8.011515789	8.021354412	8.115145455					
LOC386117	ILMN_1218079	0.0021792	2.843E-05	0.3134162	0.7582608	0.0352915	0.0033238	7.206693333	7.060794737	7.051636765	7.15335					
5031414D18RIK	ILMN_2615575	2.202E-05	3.713E-05	0.2478778	0.9464672	0.0097416	0.0141411	8.621983333	8.465123684	8.467298529	8.571345455					
IRF3	ILMN_1229320	8.067E-05	1.532E-05	0.4024593	0.3891754	0.0015421	0.0010372	7.902646667	7.720826316	7.748907353	7.866604545					
IGLC2_J00595_IG_I	ILMN_2450155	8.145E-07	7.282E-07	0.9855599	0.9370753	5.83E-05	3.367E-05	13.84993333	13.69477895	13.69675147	13.85070455					
BRD2	ILMN_3154409	0.0002138	1.179E-06	0.0923399	0.6287476	0.0458714	0.0058528	11.40748667	11.26870789	11.25570735	11.342					
SRGAP2	ILMN_1222560	6.014E-09	4.043E-08	0.0553814	0.1020981	0.0005129	0.0063007	7.94865	7.758986842	7.797158824	7.879281818					
ZFP36L1	ILMN_2780424	6.224E-05	2.451E-07	0.6304486	0.3087586	0.0058726	0.0001596	13.96748	13.84238684	13.81608971	13.94897273					
MYO7A	ILMN_1251839	8.979E-06	6.809E-06	0.0603573	0.4475788	0.0125491	0.0294065	6.819533333	6.64505	6.668164706	6.745968182					
LOC382163	ILMN_1237356	2.994E-05	2.225E-05	0.069729	0.3075783	0.0097309	0.0386567	6.892846667	6.712444737	6.742497059	6.811872727					
LOC238836	ILMN_2527694	2.39E-06	1.106E-08	0.1321515	0.9052961	0.0021451	0.0001942	13.98839	13.84113421	13.83827941	13.94409091					
HRSP12	ILMN_2610706	0.000479	0.0002885	0.1980366	0.6597663	0.0250625	0.0430031	8.18713	8.021755263	8.037048529	8.120886364					
GPS2	ILMN_2730784	0.0004475	1.596E-06	0.4350748	0.4181621	0.0063659	0.0001654	9.44959	9.323986842	9.303297059	9.419718182					
DLG3	ILMN_2993836	0.0001248	9.253E-05	0.3256219	0.4930922	0.0013149	0.0041559	7.168773333	7.001178947	7.022592647	7.125568182					
2810402K13RIK	ILMN_2455390	6.719E-08	2.192E-05	0.0913756	0.1673436	0.0016301	0.039087	8.3151	8.126965789	8.169489706	8.250713636					
SHC1	ILMN_2957070	9.867E-06	9.733E-08	0.2366389	0.3299388	0.004558	0.0003176	11.17267667	11.05177105	11.02947353	11.13645					
GALNT10	ILMN_2750011	0.0001616	4.031E-07	0.104016	0.5181983	0.0287723	0.002004	8.239243333	8.110673684	8.096067647	8.178859091					
IVD	ILMN_2728379	1.878E-05	5.595E-07	0.0979153	0.535807	0.0116603	0.0061067	9.353073333	9.195634211	9.211955882	9.293895455					
RING1	ILMN_2932539	0.0166343	1.051E-05	0.3802927	0.0714105	0.0023204	7.68E-07	8.797403333	8.707202632	8.656370588	8.835413636					
LOC100048105	ILMN_2588050	0.0009383	7.735E-08	0.4573259	0.1055718	0.0231541	5.683E-05	13.31449667	13.21344474	13.17428971	13.29137273					
CLEC4B1	ILMN_2603898	0.0006499	8.9E-05	0.9443595	0.5312531	0.0009063	0.0001392	6.123163333	5.964173684	5.98355	6.126754545					
TYROBP	ILMN_2867147	4.99E-05	0.0008134	0.324192	0.0823907	0.0009292	0.0372652	7.546993333	7.352484211	7.408191176	7.491804545					
6230425C21RIK	ILMN_2550095	3.373E-05	0.0011633	0.7674495	0.2234885	0.000104	0.0019545	7.159243333	6.973836842	7.021026471	7.173704545					
2810422O20RIK	ILMN_2625188	0.006333	2.944E-05	0.8078838	0.4390216	0.0151682	0.0001763	7.155133333	7.041215789	7.017935294	7.145077273					
RFFL	ILMN_3048689	0.0021843	9.005E-05	0.946882	0.4492621	0.007551	0.0007013	7.346986667	7.233876316	7.211079412	7.344081818					
CEPT1	ILMN_2762334	0.0001535	3.503E-06	0.1512639	0.263014	0.0316096	0.0044622	10.36051667	10.25173158	10.22485441	10.31255909					
NEURL	ILMN_2625940	1.943E-05	2.977E-06	0.0798984	0.5182001	0.0333122	0.0413976	6.091016667	5.940726316	5.956220588	6.018363636					
ZFP317	ILMN_2481799	0.0004084	2.047E-05	0.174213	0.792748	0.0368688	0.0129862	7.35243	7.224565789	7.217720588	7.297					
SRGAP3	ILMN_2648098	0.0042644	4.08E-06	0.5674557	0.0712274	0.0457434	0.0004358	6.20861	6.119747368	6.074486765	6.187718182					
RARG	ILMN_3132660	0.0002313	0.0001074	0.0961102	0.4564491	0.0249151	0.0481685	6.253086667	6.097884211	6.120816176	6.186990909					
PI4KB	ILMN_2689259	0.0001833	0.0002821	0.9041754	0.561224	0.0010439	0.0018446	9.675126667	9.524628947	9.544104412	9.670277273					
GALGT1	ILMN_1241182	0.00356	0.0002267	0.9638047	0.6243123	0.0045697	0.0005737	7.578576667	7.461855263	7.448160294	7.576309091					
PIK3R6	ILMN_3160107	0.0007543	0.0009593	0.9801863	0.7352984	0.0019019	0.003175	6.473133333	6.331334211	6.342902941	6.471927273					
MREG	ILMN_2976159	0.0115112	0.0012188	0.9705235	0.5417462	0.0076726	0.0014909	9.009966667	8.899078947	8.880347059	9.012027273					
1200002N14RIK	ILMN_1219820	0.0001629	0.0008928	0.3547823	0.7228022	6.796E-06	7.945E-05	7.02541	6.885213158	6.896238235	7.071472727					

2610028H07RIK	ILMN_1256792	6.346E-06	0.0004463	0.5340537	0.1787544	8.318E-05	0.0082396	10.22659	10.05706053	10.09811029	10.20060909					
TGFBI	ILMN_2727663	9.323E-05	0.0050672	0.6968583	0.0791519	0.0001869	0.023865	6.317396667	6.125718421	6.189858824	6.294936364					
NOS3	ILMN_2788593	0.000145	3.643E-05	0.1428581	0.8181436	0.0230466	0.0166585	5.930473333	5.810139474	5.804051471	5.8815					
USP11	ILMN_2802244	0.0008445	0.0003972	0.5473493	0.5743512	0.004636	0.0065853	6.548856667	6.408394737	6.422813235	6.516695455					
FOS	ILMN_2750515	0.0141545	0.0005959	0.5054332	0.2872521	0.0012599	5.304E-05	6.999393333	6.903897368	6.874805882	7.035045455					
LOC636818	ILMN_2434512	0.0003839	1.14E-05	0.1989409	0.6183528	0.0377477	0.0083799	6.108246667	5.997428947	5.985791176	6.062581818					
SCL0004023.1_57	ILMN_1232512	0.0002673	3.147E-05	0.7143189	0.744648	0.0027804	0.0008174	6.11199	5.997631579	5.989891176	6.097618182					
P2RX4	ILMN_2620122	0.0008163	0.0008309	0.3786711	0.7185532	0.0119073	0.0306112	7.48024	7.348384211	7.358327941	7.436886364					
DGKQ	ILMN_2972541	0.0004892	0.0005204	0.2695177	0.5259959	0.021019	0.0477871	7.016946667	6.876660526	6.895120588	6.966136364					
SNX15	ILMN_2894450	0.0093703	4.642E-05	0.2424838	0.0859399	0.0001601	6.063E-07	9.416226667	9.335971053	9.294445588	9.462340909					
SLC11A1	ILMN_2692797	0.0039669	0.0019783	0.2622018	0.8324263	0.0002494	5.829E-05	6.549723333	6.422	6.429011765	6.614281818					
TLR13	ILMN_2735961	2.319E-06	5.548E-05	0.2313394	0.0527093	0.0014045	0.0272525	6.13844	5.968510526	6.018579412	6.089945455					
MKL1	ILMN_2630120	0.0001341	0.0001303	0.2256236	0.6549713	0.0148592	0.0273433	6.422093333	6.290807895	6.302858824	6.376204545					
EG633640	ILMN_2977390	0.0021632	1.049E-05	0.3706096	0.1302664	0.0233458	0.0008458	6.2549	6.167321053	6.135855882	6.224536364					
RASGRF1	ILMN_1233146	6.199E-05	1.771E-05	0.1825115	0.6972638	0.041397	0.038674	5.882463333	5.754576316	5.763907353	5.8296					
TRP53	ILMN_2466845	0.0016928	0.0028691	0.9923206	0.7914076	0.0107981	0.0131019	9.81586	9.688071053	9.697814706	9.816331818					
1300010F03RIK	ILMN_1249480	0.0020903	0.0002448	0.9590157	0.5945485	0.0017402	0.0005547	6.72106	6.618107895	6.603588235	6.719240909					
RNF123	ILMN_2975900	0.0019712	0.0067338	0.6879115	0.5482232	0.0095547	0.0405204	7.61357	7.475247368	7.4966	7.591468182					
LOC386085	ILMN_1258858	0.007037	0.0024479	0.9931001	0.6718008	0.0234216	0.0099114	6.32579	6.223992105	6.209532353	6.326190909					
TIMM8A2	ILMN_2983631	0.0004798	7.984E-05	0.2601703	0.9288078	0.0408017	0.0257747	5.952686667	5.835228947	5.837423529	5.907509091					
CLEC4A1	ILMN_1230708	6.31E-05	0.0006805	0.0795917	0.1389685	6.292E-07	1.122E-06	6.377756667	6.218557895	6.262733824	6.464059091					
SGPL1	ILMN_2936380	0.0044252	0.0043183	0.2442032	0.8397745	1.199E-05	5.429E-05	7.595083333	7.473997368	7.480830882	7.646777273					
TMUB1	ILMN_2932660	3.677E-06	2.867E-06	0.558543	0.1829473	9.601E-05	0.0001669	8.66402	8.519415789	8.549851471	8.647472727					
LOC383099	ILMN_1230327	1.029E-05	3.432E-05	0.1935341	0.3577611	0.0026485	0.0131515	13.25080333	13.11434737	13.13688529	13.20990455					
HMOX1	ILMN_2788073	0.001442	0.0136072	0.5259227	0.119158	0.0001286	0.0015021	6.674133333	6.506121053	6.561204412	6.718672727					
ERCC4	ILMN_1259904	2.964E-05	7.189E-06	0.3072808	0.3937884	0.0020401	0.0019043	8.262506667	8.1301	8.150180882	8.232518182					
SOLH	ILMN_2508416	4.953E-05	0.0003794	0.3919807	0.1710492	0.0051436	0.0319132	8.064173333	7.913865789	7.952263235	8.027909091					
PREP	ILMN_2549494	8.777E-06	7.108E-06	0.0728319	0.9469909	0.0319696	0.0397581	8.029716667	7.916678947	7.918044118	7.973690909					
LOC100048105	ILMN_1220172	0.0052534	5.023E-06	0.6515084	0.1045044	0.0379376	0.0003052	13.58104	13.50723421	13.47030735	13.56905					
GSTM2	ILMN_1251449	1.074E-06	0.0004294	0.6287192	0.0626658	0.000349	0.0162208	6.0557	5.898139474	5.945163235	6.033840909					
EPB4.1L3	ILMN_1244272	0.0004236	0.0002688	0.976221	0.7535251	0.0023433	0.0015975	6.005856667	5.903615789	5.895541176	6.006977273					
USP22	ILMN_2990220	0.0004797	7.218E-05	0.6311193	0.7329412	0.000109	2.996E-05	9.1675	9.065165789	9.057348529	9.184159091					
1700073E17RIK	ILMN_2790902	0.0009735	0.0010071	0.3318609	0.7923655	0.0272899	0.044332	5.778756667	5.660986842	5.668639706	5.740490909					
1110014N23RIK	ILMN_2737979	0.0003219	6.972E-05	0.2435055	0.9731238	0.0164781	0.0102902	9.78732	9.676873684	9.677741176	9.754181818					
RCCD1	ILMN_2838629	0.0085449	0.0014522	0.9523493	0.722719	0.0029863	0.0011083	8.2828	8.183665789	8.173695588	8.285281818					
ANKRD13A	ILMN_2613696	0.0048483	0.0016996	0.6333023	0.6372045	0.0118232	0.0117616	10.35183	10.25657632	10.24345	10.33284091					
SP2	ILMN_2464693	0.0007692	0.0005217	0.6154789	0.8637588	0.0219028	0.0154119	6.97502	6.871657895	6.866919118	6.955763636					
CENTG3	ILMN_2754596	0.0008539	0.0066436	0.763363	0.2648393	0.0048462	0.0324186	6.891463333	6.743584211	6.784157353	6.877472727					
3110004L20RIK	ILMN_3142396	0.0049306	0.0008978	0.8322505	0.8607587	0.0011876	0.000386	6.040683333	5.938431579	5.933769118	6.049186364					
6430526N21RIK	ILMN_2556989	3.656E-06	0.0030831	0.8062834	0.0539703	0.0001224	0.0205043	7.338353333	7.170902632	7.231635294	7.327745455					
2310067B10RIK	ILMN_1245489	0.0001095	0.0021206	0.7045481	0.149667	0.0018865	0.0207657	7.60857	7.454689474	7.501908824	7.593022727					
1810030N24RIK	ILMN_1246756	0.0003314	0.0001049	0.2577743	0.7731788	0.0265102	0.0253525	6.94621	6.833397368	6.840070588	6.905022727					
RBM15	ILMN_2945275	0.0002016	1.119E-05	0.7625653	0.6260856	0.0011444	0.0001507	8.00761	7.891310526	7.901594118	7.997504545					
WWP2	ILMN_1226970	0.0013163	1.969E-05	0.5181541	0.1754847	0.0010361	2.533E-05	8.939	8.862852632	8.833398529	8.9579					
2610024M03RIK	ILMN_1255611	0.0007512	1.988E-05	0.2769578	0.6245423	0.0372041	0.0073064	6.12332	6.027731579	6.018229412	6.085745455					
C430003P19RIK	ILMN_2864393	0.0250093	0.0001025	0.7362257	0.1297357	0.0383753	0.0003539	9.67627	9.606163158	9.571339706	9.665418182					
BC066107	ILMN_2903889	0.0013885	0.0011587	0.5229082	0.6611865	0.000228	0.000209	6.6825	6.565544737	6.577964706	6.707777273					
3110082D06RIK	ILMN_2512770	0.0008126	0.0002459	0.4191441	0.8789691	0.0183839	0.0115793	6.0938	5.993510526	5.989636765	6.068118182					
LYNX1	ILMN_1256369	0.0042057	0.0007327	0.4998935	0.746914	0.0252748	0.0117316	8.635493333	8.522728947	8.531370588	8.607563636					
PRKCZ	ILMN_1221045	0.0008677	0.0080558	0.8168499	0.1401794	0.0001141	0.0031619	7.069753333	6.920815789	6.966716176	7.081818182					
IGK-V33	ILMN_1248308	0.0190094	0.0430061	0.8357281	0.7053464	0.0069368	0.0316055	10.09401333	9.976852632	9.992255882	10.10707727					
SLC11A1	ILMN_2674884	0.0045173	0.0309152	0.1250275	0.1360546	1.786E-05	6.406E-05	6.997713333	6.838144737	6.896435294	7.107336364					
CBLC	ILMN_2738699	0.000129	0.0034848	0.5346577	0.1576133	0.0004127	0.0292504	5.966836667	5.825323684	5.865586765	5.941622727					
B930049N04RIK	ILMN_2567282	0.0082852	0.0004187	0.7132546	0.6213679	0.0314128	0.00413	5.693346667	5.604668421	5.592219118	5.679722727					
CASP1	ILMN_1247592	0.0365454	0.0002782	0.98551	0.1513468	0.0348947	0.0004103	11.61187333	11.54446316	11.51241912	11.61258182					
MBD1	ILMN_2893081	0.0006904	0.0009885	0.9341784	0.4951872	0.0011696	0.0026387	9.107946667	8.991671053	9.009091176	9.104772727					
2510022D24RIK	ILMN_2431257	0.0005423	0.0005419	0.6621285	0.8555194	0.0096387	0.010535	8.239006667	8.136547368	8.141229412	8.224509091					
FRMPD1	ILMN_2625610	0.0078477	8.549E-05	0.6896953	0.175579	0.0487525	0.0022095	5.682146667	5.613552632	5.584472059	5.669695455					
USP54	ILMN_2418324	0.0095547	0.0004016	0.9885197	0.3747777	0.0098927	0.0008142	7.069403333	6.990452632	6.971775	7.068827273					
FA2H	ILMN_2746783	0.0047186	0.0006321	0.4577042	0.9828413	0.0329353	0.0131101	6.074253333	5.976113158	5.976633824	6.047031818					

EG434402	ILMN_2546510	0.0001282	5.523E-05	0.1356377	0.2699795	0.0095784	0.0185516	9.268486667	9.145802632	9.171463235	9.227836364					
SLC9A4	ILMN_2732246	0.0021241	0.0001248	0.3842808	0.8904557	0.0274504	0.0068125	5.812063333	5.718157895	5.715114706	5.784231818					
ATP6V1B2	ILMN_2680440	0.0331736	0.0026486	0.2271266	0.2349906	0.0020564	0.0001548	10.49800333	10.43401842	10.40123971	10.54807273					
2310079N02RIK	ILMN_2635801	0.001275	7.915E-05	0.6000429	0.9085553	0.0144292	0.0029858	9.192623333	9.095097368	9.097661765	9.17595					
ZFPL1	ILMN_1217262	0.0103109	0.0104877	0.4419646	0.8781598	0.0011273	0.0014541	7.48469	7.385313158	7.390122059	7.521240909					
NISCH	ILMN_1237959	0.0004887	0.0022285	0.9947962	0.1362464	0.0013958	0.0054091	7.18114	7.045213158	7.087505882	7.181422727					
FCMD	ILMN_2514924	0.0084295	0.0038963	0.9495455	0.6291816	0.0175612	0.0101721	6.566743333	6.486473684	6.473394118	6.569354545					
1810009N02RIK	ILMN_2764607	0.0134778	0.0012708	0.1392451	0.5690381	0.0002188	7.598E-06	8.202653333	8.123931579	8.109642647	8.261063636					
6720486H14RIK	ILMN_1232302	0.0136264	0.0004247	0.8667953	0.5443658	0.0422079	0.0033688	5.675496667	5.599039474	5.583961765	5.670122727					
AW011738	ILMN_1248890	0.0210894	0.0024042	0.7444499	0.2578455	0.0466497	0.0126033	6.340756667	6.277447368	6.2494	6.329786364					
COgeneric	ILMN_2596412	0.0028243	0.0022236	0.6376439	0.4780665	0.0154442	0.0199386	8.195383333	8.085518421	8.104542647	8.177086364					
PCBP2	ILMN_2599777	0.0007785	0.0001023	0.3157339	0.7419453	0.0163777	0.0094039	12.72348667	12.62733947	12.63373235	12.69256364					
C330019G07RIK	ILMN_1256807	0.018907	0.0045064	0.9564866	0.8735275	0.0177015	0.0058732	6.827063333	6.742765789	6.738351471	6.829163636					
SMARCE1	ILMN_2880246	0.0023537	0.0014714	0.5224221	0.4944281	0.0254248	0.032737	9.420046667	9.316405263	9.331558824	9.392968182					
ABCC4	ILMN_2621708	0.0033419	0.0023598	0.8663787	0.882039	0.0096273	0.0096422	7.844656667	7.752781579	7.756655882	7.838990909					
GTPBP1	ILMN_2510196	0.0094972	0.0163723	0.8623046	0.5356694	0.0083026	0.0160378	9.273093333	9.164928947	9.185194118	9.281127273					
SLC25A42	ILMN_2810937	0.0030623	0.0038466	0.8626579	0.509224	0.007698	0.0080427	6.299283333	6.193821053	6.212104412	6.306677273					
5830415F09RIK	ILMN_1223695	1.314E-05	0.0004369	0.2658477	0.1535108	0.0020511	0.0405941	7.08473	6.966402632	6.997625	7.052472727					
PEO1	ILMN_2913845	0.000207	0.0037761	0.6475825	0.2792846	0.0032424	0.0364357	7.222113333	7.107710526	7.1356	7.205309091					
CRTAC1	ILMN_2764112	0.0314498	0.0050598	0.4815776	0.3443996	0.00865	0.0018008	6.011773333	5.950531579	5.925355882	6.037995455					
CLEC5A	ILMN_3045984	4.595E-05	0.0023644	0.9952611	0.065168	0.0001399	0.0058405	5.95908	5.828018421	5.873202941	5.958854545					
ZFP704	ILMN_1225146	0.0157979	0.0022986	0.436168	0.4060296	0.0051091	0.0008179	5.9147	5.849613158	5.828823529	5.941309091					
TNS4	ILMN_1236029	0.0447347	0.0035216	0.6156233	0.4066194	0.0335541	0.0029568	5.844593333	5.780189474	5.758717647	5.866986364					
2310040G07RIK	ILMN_1232245	0.0014676	0.003644	0.9801709	0.5338841	0.0018196	0.0064955	5.79773	5.696360526	5.712151471	5.798627273					
KRTAP5-4	ILMN_2817498	0.0118382	0.0033168	0.7848286	0.6047325	0.0364951	0.0175473	5.736486667	5.664107895	5.651308824	5.727004545					
D030051N19RIK	ILMN_2760593	0.0176724	0.0088324	0.7626964	0.9526906	0.0035265	0.0028821	8.371213333	8.285613158	8.287195588	8.383390909					
A430043M19RIK	ILMN_2553758	0.0153723	0.001537	0.8701781	0.7000002	0.0216004	0.0037935	6.192146667	6.116665789	6.108377941	6.186131818					
9030025P20RIK	ILMN_2716783	0.0255003	0.0010439	0.910137	0.5226017	0.0247396	0.0013441	8.676186667	8.607128947	8.592552941	8.680018182					
GAS7	ILMN_2924831	0.0002814	0.0155795	0.4890746	0.1450892	7.41E-05	0.0041312	6.734093333	6.607315789	6.6505	6.764486364					
TBC1D25	ILMN_2722583	0.0001741	0.0006019	0.7508305	0.1725744	0.0003484	0.0007655	8.04817	7.934818421	7.965151471	8.058922727					
LOC193563	ILMN_1234524	0.0203014	0.0021195	0.4937336	0.3753691	0.0098347	0.0011714	5.841313333	5.780481579	5.758816176	5.863127273					
PAPSS2	ILMN_2466809	0.0007114	0.0106323	0.5969308	0.1975621	0.0001305	0.0028935	6.744143333	6.628768421	6.661655882	6.768309091					
ELL	ILMN_2751304	0.0043874	0.0021851	0.5671152	0.9478309	0.034593	0.0297732	9.71715	9.633271053	9.634791176	9.697881818					
SHKBP1	ILMN_2734334	0.0053095	0.0435104	0.4581237	0.229169	0.0001639	0.0045941	8.369346667	8.246628947	8.287191176	8.407618182					
RNF10	ILMN_2789888	0.0027989	0.0004689	0.9566584	0.9104826	0.0098144	0.0026066	11.53589	11.45141053	11.45382206	11.53415455					
FBXL20	ILMN_2729097	0.0235441	0.009027	0.9843672	0.9957199	0.0378707	0.0190251	7.886646667	7.80505	7.804917647	7.885722727					
TEAD4	ILMN_3157399	0.0141637	0.0027862	0.9554286	0.7134498	0.0466475	0.0137869	6.07582	6.003681579	5.99445	6.073868182					
5031439G07RIK	ILMN_1252809	0.0362145	0.0054091	0.7584422	0.7313907	0.0207321	0.0032499	8.046333333	7.975907895	7.966855882	8.056904545					
LOC100046003	ILMN_2427178	0.0018293	0.0012967	0.7160404	0.9825846	0.0336408	0.0243578	8.814313333	8.735331579	8.734867647	8.801768182					
B130002B06RIK	ILMN_1231100	0.002085	0.0009077	0.6275775	0.9155026	0.0127637	0.0106019	5.813043333	5.732515789	5.7347	5.7991					
EPN3	ILMN_1239908	0.0081262	0.0018557	0.6086819	0.7130121	0.0408573	0.0200886	5.787266667	5.702681579	5.710923529	5.770054545					
ZC3H12A	ILMN_2856095	0.023892	0.0152977	0.1041123	0.9579116	0.0012163	0.0003304	8.291086667	8.213755263	8.215352941	8.354177273					
2900079J23RIK	ILMN_2458967	0.000691	0.005917	0.8841941	0.2598759	0.0034558	0.0218172	5.841546667	5.736136842	5.765825	5.837059091					
CLEC4B1	ILMN_2959372	0.004988	0.0056659	0.1168793	0.7320233	9.394E-05	5.148E-05	5.798906667	5.716681579	5.724957353	5.853954545					
1300010F03RIK	ILMN_2688415	0.0111316	0.0044511	0.6250101	0.903061	0.041781	0.0317985	6.837643333	6.766381579	6.763723529	6.82175					
KRT39	ILMN_2699061	0.0002457	0.0032069	0.6653659	0.1639792	0.0044085	0.0360122	5.62461	5.520444737	5.551114706	5.6101					
4930519F16RIK	ILMN_2812960	0.0150436	0.0119516	0.7117498	0.8622955	0.0128692	0.0100083	5.689996667	5.611807895	5.616554412	5.702931818					
RHBDD1	ILMN_2680086	0.0019562	0.0265314	0.7428231	0.153613	0.0004957	0.0125101	7.70038	7.587589474	7.627182353	7.714345455					
GDPD1	ILMN_2683528	0.0252047	0.014226	0.9742184	0.976903	0.029161	0.0218301	6.465226667	6.393168421	6.392429412	6.466463636					
ACP2	ILMN_2513205	0.0258929	0.0480152	0.8783636	0.5194416	0.0090843	0.0298416	6.88359	6.791065789	6.810866176	6.8907					
SETD1A	ILMN_1247099	0.029141	0.0180293	0.2892856	0.8016873	0.0023861	0.0009068	9.028223333	8.94825	8.955577941	9.065645455					
OLFR516	ILMN_2975511	0.0058453	0.0065686	0.6318905	0.8480392	0.0046306	0.0050237	5.832843333	5.755394737	5.760235294	5.846913636					
REN1	ILMN_2620114	0.0085901	0.0035255	0.5194519	0.9189352	0.0459334	0.039878	5.670936667	5.596518421	5.598602941	5.650036364					
5330417C22RIK	ILMN_3160472	0.0039298	0.0166523	0.8183059	0.3293711	0.0123984	0.0286015	5.710553333	5.612434211	5.640252941	5.719795455					
AGAP1	ILMN_3086136	0.0013012	0.0142837	0.2177918	0.1437627	0.0002803	0.000833	6.059153333	5.948686842	5.989294118	6.109681818					
DUSP6	ILMN_2701321	0.0419611	0.0207727	0.6564169	0.7667014	0.0151341	0.0053288	5.8376	5.760176316	5.768070588	5.856759091					
CTSL	ILMN_2904703	0.0082365	0.0336987	0.688531	0.394718	0.0033249	0.0172177	5.81264	5.718744737	5.743730882	5.827822727					
SLCO6C1	ILMN_2672845	0.0036617	0.0034252	0.8298071	0.5075665	0.0093559	0.007347	5.686233333	5.602471053	5.617510294	5.693277273					
TBC1D2	ILMN_3140913	0.0091587	0.0270787	0.0776191	0.1161568	5.306E-05	8.344E-06	6.907116667	6.793013158	6.838802941	6.992163636					
OPHN1	ILMN_1218910	0.0070562	0.0167606	0.9612701	0.5267743	0.0127471	0.0292428	5.778693333	5.693957895	5.710407353	5.780409091					

B930048N13RIK	ILMN_1228496	0.0258409	0.0231931	0.9459577	0.6860792	0.0324665	0.0364817	5.961773333	5.883607895	5.894567647	5.95935					
MBL1	ILMN_1238266	0.0194917	0.0187551	0.6216545	0.9538341	0.0038567	0.0070378	5.619243333	5.55345	5.552047059	5.635831818					
A730017D01RIK	ILMN_2448591	0.0351444	0.0250831	0.2771874	0.9789299	0.002874	0.0021749	6.58584	6.519621053	6.518897059	6.621677273					
SNX33	ILMN_1212693	0.0012835	0.035465	0.7593182	0.0768198	0.0017864	0.0303962	7.0541	6.931763158	6.987372059	7.065340909					
E330023P07RIK	ILMN_1240762	0.0403921	0.0100513	0.9004208	0.6995832	0.0425864	0.0176601	5.836216667	5.778460526	5.770314706	5.832118182					
9330152L17	ILMN_2726288	0.0068496	0.0099609	0.8218654	0.6514086	0.003115	0.0070201	5.81598	5.740573684	5.750429412	5.823040909					
PCDH19	ILMN_2696829	0.0097893	0.0131131	0.972637	0.9702742	0.0157433	0.0250866	5.694973333	5.629565789	5.630432353	5.695972727					
CAGE1	ILMN_2861743	0.0002703	0.0145488	0.7809247	0.0671697	0.0006313	0.0159241	5.926	5.816742105	5.861688235	5.935345455					
TMEM56	ILMN_2966162	0.0462108	0.0184806	0.4105332	0.6403937	0.0091299	0.0038463	5.612496667	5.559673684	5.548541176	5.640304545					
ACTR3B	ILMN_2933346	0.0245032	0.0282414	0.8984635	0.8932447	0.0154677	0.0280153	6.533076667	6.4662	6.469547059	6.5374					
ADAM11	ILMN_2629383	0.0026248	0.0095577	0.9602058	0.3205222	0.0136472	0.0338978	5.691363333	5.605971053	5.628829412	5.689736364					
CD151	ILMN_2836710	0.0193575	0.0252142	0.9583206	0.5738569	0.027328	0.0420257	8.090336667	8.015257895	8.028898529	8.088440909					
A930014P08RIK	ILMN_1258114	0.0165319	0.0226352	0.7989225	0.9591495	0.0202646	0.0296379	5.6155	5.555547368	5.554239706	5.621622727					
SLC35E4	ILMN_1225229	0.0120001	0.0364782	0.2142271	0.5822929	0.0006084	0.0021022	5.867336667	5.791586842	5.806241176	5.91005					
PARD3B	ILMN_2860656	0.0221362	0.0242476	0.7871277	0.357316	0.0180473	0.0146645	5.96533	5.880542105	5.904663235	5.975636364					
OLFR586	ILMN_2600115	0.0262931	0.0300541	0.4454466	0.5125899	0.0112671	0.0074731	5.601076667	5.522747368	5.541236765	5.627940909					
D030072B18RIK	ILMN_2611773	0.0121881	0.0138297	0.7042	0.5520373	0.0048147	0.0055113	5.61957	5.547055263	5.559957353	5.631459091					
STARD3	ILMN_2769734	0.0325095	0.0437294	0.7159143	0.6153939	0.0263459	0.0293263	8.494313333	8.422068421	8.435257353	8.509754545					
SFXN3	ILMN_1216734	0.0084465	0.0369416	0.3332499	0.4315651	0.0004631	0.0027125	6.08399	6.006510526	6.025077941	6.121263636					
6430511F03	ILMN_2420209	0.0105929	0.0296337	0.595619	0.682983	0.0051195	0.014191	6.304863333	6.2371	6.246623529	6.322872727					
PROM	ILMN_1238150	0.0039862	0.0081546	0.4662896	0.4395203	0.0006049	0.0010628	5.928333333	5.855444737	5.870188235	5.950177273					
NTE	ILMN_2777668	0.0022045	0.0233767	0.8255128	0.1273946	0.0009399	0.014351	8.366106667	8.27475	8.3088	8.373113636					
OLFR150	ILMN_2541702	0.0205861	0.0086368	0.8151015	0.7718418	0.0358411	0.0222281	5.682813333	5.620110526	5.625554412	5.675772727					
2010001M09RIK	ILMN_2613601	0.0120013	0.0226729	0.063086	0.3826246	1.211E-05	6.331E-06	13.25162667	13.17815526	13.19607647	13.31843636					
KCNC4	ILMN_2771095	0.0157139	0.0423165	0.5244547	0.4022856	0.000772	0.0045142	5.88774	5.814560526	5.832483824	5.911813636					
GM498	ILMN_1247100	0.0436667	0.0088286	0.4972216	0.7028412	0.0293635	0.0052678	5.727336667	5.681271053	5.673501471	5.744981818					
LOC327767	ILMN_2533815	0.0075878	0.0370241	0.3781781	0.3051498	0.002516	0.0069701	6.031383333	5.952915789	5.978073529	6.061031818					
B930097H17RIK	ILMN_2573844	0.0026075	0.0250972	0.4526146	0.1224496	0.0007489	0.0049604	5.844733333	5.755813158	5.792060294	5.865090909					
BMP3	ILMN_1235433	0.0246822	0.0485117	0.5437379	0.9290625	0.0197826	0.0285056	5.675396667	5.620692105	5.622867647	5.694827273					
PLEKHB2	ILMN_2731769	0.001378	0.0287559	0.9350244	0.0991679	0.0013793	0.0451558	9.05776	8.97335	9.006016176	9.055263636					
A630038P11RIK	ILMN_1233452	0.0113427	0.0430203	0.6978785	0.5092482	0.004887	0.0239387	5.602433333	5.537278947	5.551530882	5.614654545					
6720480F11RIK	ILMN_1255586	0.0342299	0.0372474	0.8391307	0.5635111	0.0405714	0.0386927	5.689046667	5.629671053	5.641458824	5.696195455					
CNTNAP3	ILMN_1219653	0.0337739	0.0448523	0.2011159	0.6359243	0.0030471	0.002119	5.841703333	5.786078947	5.796410294	5.878831818					
