# Supplementary material for: Comparing the Biological Impact of Glatiramer Acetate with the Biological Impact of a Generic
Source: PLoS One. 2014 Jan 8;9(1):e83757. doi: 10.1371/journal.pone.0083757 (PMC3885444; doi:10.1371/journal.pone.0083757)
Supplement: Table S8 — Outputs of cell-type enrichment analyses for various lists of genes (higher expression in GA than generic, higher expression in generic than GA, upregulated only by generic, downregulated only by generic, upregulated only by GA and reference standard, downregulated only by GA and reference standard). (PDF) [file pone.0083757.s016.pdf]

TABLE S8

	4-method_up GA				4-method_up generic		
Sample ID	Score	p-value	adjusted p-value	Sample ID	Score	p-value	adjusted p-value
T.8Nve.Sp.OT1.rnk	78.016717	1.30E-93	3.14E-92	MF.Lu.rnk	52.365983	4.74E-19	5.33E-18
T.4FP3.25..LN.rnk	77.258574	4.58E-92	9.88E-91	MF.BM.rnk	52.167673	8.50E-27	4.12E-25
CD4Control.rnk	74.792428	5.10E-82	4.95E-81	Mo.6C.II..BM.rnk	51.566882	3.63E-25	1.41E-23
CD4TESTCJ.rnk	74.304957	7.18E-100	3.48E-98	SC.GMP.BM.rnk	51.558744	7.77E-33	7.53E-31
T.4Nve.Sp.rnk	74.117612	6.71E-104	6.51E-102	MF.11cloSer.SI.rnk	46.967776	2.67E-16	1.56E-15
T.8SP24..Th.rnk	73.133104	6.90E-91	1.34E-89	MLP.FL.rnk	46.519979	7.74E-32	5.01E-30
T.8Nve.LN.rnk	72.608165	2.84E-97	7.86E-96	MF.Thio5.II.480int.PC.rnk	46.093861	1.84E-18	1.55E-17
T.4SP24..Th.rnk	70.766828	3.66E-98	1.18E-96	MF.Thio5.II.480hi.PC.rnk	45.202935	2.74E-16	1.56E-15
T.8Nve.MLN.rnk	69.029666	2.66E-98	1.03E-96	MF.103.11b..SI.rnk	45.121929	6.17E-10	1.93E-09
T.8SP24int.Th.rnk	64.965353	5.23E-76	4.06E-75	MF.II.480hi.PC.rnk	42.259112	5.29E-17	3.66E-16
T.4Nve.LN.rnk	63.682185	1.43E-100	9.23E-99	MF.11cloSer.Salm3.SI.rnk	40.791601	2.09E-15	1.04E-14
CD4TESTJS.rnk	60.571137	1.45E-90	2.56E-89	MF.II.480lo.PC.rnk	39.348471	1.11E-20	1.66E-19
Tgd.vg2..Sp.rnk	59.521782	1.14E-81	1.05E-80	MF.103.11b..Salm3.SI.rnk	37.916306	4.52E-12	1.79E-11
T.4Mem.Sp.rnk	58.4054	1.28E-74	9.20E-74	MF.Thio5.II.480lo.PC.rnk	37.295189	6.98E-16	3.87E-15
T.4Mem44h62l.Sp.rnk	57.606055	1.08E-73	6.98E-73	DC.8..Th.rnk	36.10766	1.28E-16	8.46E-16

4-method_up GA

Cell type	Score	p-value	adjusted p-value
CD8+ T-cell	78.016717	1.30E-93	3.14E-92
*****CD4+ T-cell (FOXP3+)*****	**77.258574**	**4.58E-92**	**9.88E-91**
CD4+ T-cell	74.792428	5.10E-82	4.95E-81
CD4+ T-cell	74.304957	7.18E-100	3.48E-98
CD4+ T-cell	74.117612	6.71E-104	6.51E-102
CD8+ T-cell	73.133104	6.90E-91	1.34E-89
CD8+ T-cell	72.608165	2.84E-97	7.86E-96
CD4+ T-cell	70.766828	3.66E-98	1.18E-96
CD8+ T-cell	69.029666	2.66E-98	1.03E-96
CD8+ T-cell	64.965353	5.23E-76	4.06E-75
CD4+ T-cell	63.682185	1.43E-100	9.23E-99
CD4+ T-cell	60.571137	1.45E-90	2.56E-89

4-method_up generic

Cell type	Score	p-value	adjusted p-value
Macrophage Cell	52.365983	4.74E-19	5.33E-18
Macrophage Cell	52.167673	8.50E-27	4.12E-25
*****MONOCYTE CELL*****	**51.566882**	**3.63E-25**	**1.41E-23**
**Stromal Cell**	**51.558744**	**7.77E-33**	**7.53E-31**
Macrophage Cell	46.967776	2.67E-16	1.56E-15
**Multi-lineage Progenitor**	**46.519979**	**7.74E-32**	**5.01E-30**
Macrophage Cell	46.093861	1.84E-18	1.55E-17
Macrophage Cell	45.202935	2.74E-16	1.56E-15
Macrophage Cell	45.121929	6.17E-10	1.93E-09
Macrophage Cell	42.259112	5.29E-17	3.66E-16
Macrophage Cell	40.791601	2.09E-15	1.04E-14
Macrophage Cell	39.348471	1.11E-20	1.66E-19

Gamma Delta T-Cell	59.521782	1.14E-81	1.05E-80
CD4+ T-cell	58.4054	1.28E-74	9.20E-74
CD4+ T-cell	57.606055	1.08E-73	6.98E-73

Macrophage Cell	37.916306	4.52E-12	1.79E-11
Macrophage Cell	37.295189	6.98E-16	3.87E-15
**Dendritic Cell**	**36.10766**	**1.28E-16**	**8.46E-16**

	UP_in_GA					DOWN_in_GA			
Sample ID	Score	p-value	adjusted p-value		Sample ID	Score	p-value	adjusted p-value	
Tgd.vg2.24ahi.e17.Th.rnk	55.300114	9.65E-11	2.27E-09		MF.Thio5.II.480int.PC.rnk	78.362994	7.87E-13	6.06E-11	
Tgd.vg1.vd6.24ahi.Th.rnk	51.659013	1.41E-11	5.31E-10		MF.103.11b..SI.rnk	70.679218	1.05E-05	5.38E-05	
T.8Eff.Sp.OT1.d5.VSVOva.rnk	49.385908	3.58E-15	3.37E-13		MF.11cloSer.SI.rnk	70.056104	8.81E-09	6.46E-08	
Tgd.vg3.24alo.e17.Th.rnk	48.443386	1.36E-10	2.57E-09		MF.Thio5.II.480hi.PC.rnk	69.920643	4.45E-10	7.61E-09	
T.8Eff.Sp.OT1.d6.LisOva.rnk	44.787648	5.19E-11	1.39E-09		MF.11cloSer.Salm3.SI.rnk	66.685274	3.26E-12	1.25E-10	
NKT.44.NK1.1..Th.rnk	44.353764	5.06E-07	3.07E-06		MF.103.11b..Salm3.SI.rnk	62.942816	1.69E-09	1.63E-08	
T.8SP24int.Th.rnk	42.865925	4.45E-08	4.92E-07		GN.BM.rnk	60.965868	3.15E-10	6.06E-09	
T.DPbl.Th.rnk	42.195941	7.68E-10	1.31E-08		MF.Lu.rnk	58.386331	8.60E-11	2.21E-09	
Tgd.vg5.24ahi.Th.rnk	40.991976	3.97E-12	1.86E-10		MF.II.480hi.PC.rnk	55.697691	9.43E-10	1.25E-08	
T.8Eff.Sp.OT1.48hr.LisOva.rnk	40.833999	8.39E-07	4.78E-06		GN.Thio.PC.rnk	53.404852	8.61E-11	2.21E-09	
T.4FP3.25..LN.rnk	40.093191	1.51E-05	5.06E-05		GN.Arth.BM.rnk	53.148698	1.07E-09	1.26E-08	
T.4Mem.Sp.rnk	40.034017	6.10E-06	2.39E-05		MF.RP.Sp.rnk	50.282383	1.63E-09	1.63E-08	
T.8SP24..Th.rnk	39.397607	1.32E-07	1.25E-06		MF.Thio5.II.480lo.PC.rnk	50.16729	6.62E-09	5.10E-08	
T.4SP24..Th.rnk	37.645363	1.91E-09	2.56E-08		Mo.6C.II..LN.rnk	49.39474	9.73E-10	1.25E-08	
T.4Mem44h62l.Sp.rnk	36.123266	2.52E-06	1.21E-05		Mo.6C.II..BM.rnk	49.223068	1.60E-08	1.07E-07	

UP_in_GA

	**Score**	**p-value**	**adjusted p-value**
Gamma Delta T-Cell	55.300114	9.648E-11	2.267E-09
Gamma Delta T-Cell	51.659013	1.412E-11	5.308E-10
CD8+ T-cell	49.385908	3.581E-15	3.366E-13
Gamma Delta T-Cell	48.443386	1.365E-10	2.565E-09
CD8+ T-cell	44.787648	5.188E-11	1.393E-09
Natural Killer T-Cell	44.353764	5.056E-07	3.066E-06
CD8+ T-cell	42.865925	4.449E-08	4.921E-07
CD4+ CD8+ T-cell blast	42.195941	7.676E-10	1.312E-08

DOWN_in_GA

	**Score**	**p-value**	**adjusted p-value**
Macrophage	78.362994	7.873E-13	6.063E-11
Macrophage	70.679218	1.048E-05	5.38E-05
Macrophage	70.056104	8.813E-09	6.463E-08
Macrophage	69.920643	4.449E-10	7.613E-09
Macrophage	66.685274	3.257E-12	1.254E-10
Macrophage	62.942816	1.689E-09	1.625E-08
**Granulocytes**	**60.965868**	**3.145E-10**	**6.055E-09**
Macrophage	58.386331	8.602E-11	2.209E-09

Gamma Delta T-Cell	40.991976	3.967E-12	1.864E-10
CD8+ T-cell	40.833999	8.385E-07	4.777E-06
CD4+ T-cell (FOXP3+)	40.093191	1.507E-05	5.06E-05
CD4+ T-cell	40.034017	6.104E-06	2.391E-05
CD8+ T-cell	39.397607	1.325E-07	1.245E-06
CD4+ T-cell	37.645363	1.91E-09	2.565E-08
CD4+ T-cell	36.123266	2.516E-06	1.213E-05

Macrophage	55.697691	9.426E-10	1.249E-08
**Granulocytes**	**53.404852**	**8.606E-11**	**2.209E-09**
**Granulocytes**	**53.148698**	**1.067E-09**	**1.264E-08**
Macrophage	50.282383	1.628E-09	1.625E-08
Macrophage	50.16729	6.621E-09	5.098E-08
*****MONOCYTE CELL*****	**49.39474**	**9.734E-10**	**1.249E-08**
*****MONOCYTE CELL*****	**49.223068**	**1.601E-08**	**1.072E-07**

	UP_in_generic					DOWN_in_generic			
Sample ID	Score	p-value	adjusted p-value		Sample ID	Score	p-value	adjusted p-value	
FRC.MLN.rnk	52.352931	3.50E-12	1.89E-10		CD4Control.rnk	27.128445	2.16E-11	8.40E-10	
FRC.SLN.rnk	50.863086	4.71E-10	9.54E-09		T.4SP24..Th.rnk	25.703994	2.33E-10	3.70E-09	
Fi.Sk.rnk	50.416456	5.96E-11	1.61E-09		CD4TESTCJ.rnk	25.048321	4.40E-11	1.22E-09	
LEC.SLN.rnk	50.342848	8.82E-07	6.81E-06		T.8Nve.Sp.OT1.rnk	23.837814	6.69E-10	7.63E-09	
MF.Thio5.II.480hi.PC.rnk	46.614424	2.72E-12	1.89E-10		T.4FP3.25..LN.rnk	23.830619	3.47E-09	2.59E-08	
LEC.MLN.rnk	43.323945	4.18E-07	3.57E-06		T.4Nve.Sp.rnk	23.597706	3.09E-10	4.28E-09	
St.31.38.44..SLN.rnk	41.849758	1.77E-08	2.60E-07		T.4SP69..Th.rnk	22.613314	4.58E-13	2.22E-11	
BEC.SLN.rnk	39.075496	5.58E-06	3.62E-05		T.8SP24int.Th.rnk	22.204889	2.66E-08	1.47E-07	
BEC.MLN.rnk	38.258788	1.37E-05	8.55E-05		T.8SP24..Th.rnk	21.831712	1.02E-08	6.17E-08	
MF.Thio5.II.480int.PC.rnk	38.151955	1.13E-11	4.56E-10		T.4SP24int.Th.rnk	21.588615	7.01E-14	6.80E-12	
Fi.MTS15..Th.rnk	34.64529	4.61E-05	2.34E-04		T.8Eff.Sp.OT1.d6.LisOva.rn	20.621541	9.85E-07	3.61E-06	
MF.BM.rnk	32.936497	6.48E-08	7.50E-07		Tgd.vg2.24ahi.e17.Th.rnk	20.390136	6.79E-04	0.0016877	
MF.103.11b..SI.rnk	32.877997	1.54E-05	9.22E-05		T.4Mem44h62l.LN.rnk	20.158235	4.92E-09	3.41E-08	
MF.II.480lo.PC.rnk	31.911268	9.99E-09	1.62E-07		T.8Nve.LN.rnk	19.500055	8.43E-10	9.09E-09	
MF.II.480hi.PC.rnk	31.785718	6.25E-07	5.06E-06		T.4Mem.Sp.rnk	19.281602	1.35E-09	1.25E-08	

UP_in_generic

	**Score**	**p-value**	**adjusted p-value**
**fibroblastic reticular cell**	**52.352931**	**3.497E-12**	**1.889E-10**
**fibroblastic reticular cell**	**50.863086**	**4.711E-10**	**9.539E-09**
**fibroblast**	**50.416456**	**5.964E-11**	**1.61E-09**

DOWN_in_generic

	**Score**	**p-value**	**adjusted p-value**
CD4+ T-cell	27.128445	2.16E-11	8.40E-10
CD4+ T-cell	25.703994	2.33E-10	3.70E-09
CD4+ T-cell	25.048321	4.40E-11	1.22E-09

**lymphatic endothelial cell**	**50.342848**	**8.823E-07**	**6.806E-06**
Macrophages	46.614424	2.72E-12	1.89E-10
**lymphatic endothelial cell**	**43.323945**	**4.183E-07**	**3.567E-06**
**Stromal Cell**	**41.849758**	**1.772E-08**	**2.603E-07**
**blood endothelial cell**	**39.075496**	**5.581E-06**	**3.616E-05**
**blood endothelial cell**	**38.258788**	**1.372E-05**	**8.55E-05**
Macrophages	38.151955	1.13E-11	4.56E-10
**fibroblast**	**34.64529**	**4.613E-05**	**0.0002335**
Macrophages	32.936497	6.48E-08	7.50E-07
Macrophages	32.877997	1.54E-05	9.22E-05
Macrophages	31.911268	9.99E-09	1.62E-07
Macrophages	31.785718	6.25E-07	5.06E-06

CD8+ T-cell	23.837814	6.69E-10	7.63E-09
*****CD4+ T-cell (FOXP3+)****	**23.830619**	**3.468E-09**	**2.587E-08**
CD4+ T-cell	23.597706	3.09E-10	4.28E-09
CD4+ T-cell	22.613314	4.58E-13	2.22E-11
CD8+ T-cell	22.204889	2.66E-08	1.47E-07
CD8+ T-cell	21.831712	1.02E-08	6.17E-08
CD4+ T-cell	21.588615	7.01E-14	6.80E-12
CD8+ T-cell	20.621541	9.85E-07	3.61E-06
Gamma Delta T-Cell	20.390136	6.79E-04	0.0016877
CD4+ T-cell	20.158235	4.92E-09	3.41E-08
CD8+ T-cell	19.500055	8.43E-10	9.09E-09
CD4+ T-cell	19.281602	1.35E-09	1.25E-08
