# Supplementary material for: Comparing the Biological Impact of Glatiramer Acetate with the Biological Impact of a Generic
Source: PLoS One. 2014 Jan 8;9(1):e83757. doi: 10.1371/journal.pone.0083757 (PMC3885444; doi:10.1371/journal.pone.0083757)
Supplement: Table S9 — List of genes depicted in the heat map in Figure 3 . (PDF) [file pone.0083757.s017.pdf]

| TABLE S9 | | | |
|---|---|---|---|
| | | | |
| Row | Gene | Probe | FoxP3+T Cells |
| 1 | RASGRP1 | ILMN_1214318 | FoxP3+T Cells |
| 2 | IL6RA | ILMN_1216057 | FoxP3+T Cells |
| 3 | CAMK2D | ILMN_1216561 | FoxP3+T Cells |
| 4 | PRKCH | ILMN_1216897 | FoxP3+T Cells |
| 5 | ITK | ILMN_1217899 | FoxP3+T Cells |
| 6 | IQGAP2 | ILMN_1218051 | FoxP3+T Cells |
| 7 | PTPN22 | ILMN_1220975 | FoxP3+T Cells |
| 8 | ZAP70 | ILMN_1223542 | FoxP3+T Cells |
| 9 | ST8SIA4 | ILMN_1223948 | FoxP3+T Cells |
| 10 | SKAP1 | ILMN_1224032 | FoxP3+T Cells |
| 11 | SMPDL3A | ILMN_1224116 | FoxP3+T Cells |
| 12 | FAM102A | ILMN_1225348 | FoxP3+T Cells |
| 13 | ITGB7 | ILMN_1227434 | FoxP3+T Cells |
| 14 | RCAN3 | ILMN_1227610 | FoxP3+T Cells |
| 15 | TRAT1 | ILMN_1229005 | FoxP3+T Cells |
| 16 | FAAH | ILMN_1229163 | FoxP3+T Cells |
| 17 | PDK1 | ILMN_1229318 | FoxP3+T Cells |
| 18 | DDB2 | ILMN_1229502 | FoxP3+T Cells |
| 19 | ECM1 | ILMN_1229746 | FoxP3+T Cells |
| 20 | CEP97 | ILMN_1230717 | FoxP3+T Cells |
| 21 | LEF1 | ILMN_1231087 | FoxP3+T Cells |
| 22 | ZNRF1 | ILMN_1232073 | FoxP3+T Cells |
| 23 | SAMHD1 | ILMN_1232707 | FoxP3+T Cells |
| 24 | IRF9 | ILMN_1233461 | FoxP3+T Cells |
| 25 | DTNB | ILMN_1233894 | FoxP3+T Cells |
| 26 | PPP3CC | ILMN_1233999 | FoxP3+T Cells |
| 27 | P4HA1 | ILMN_1234565 | FoxP3+T Cells |
| 28 | FXYD5 | ILMN_1235493 | FoxP3+T Cells |
| 29 | PROS1 | ILMN_1235499 | FoxP3+T Cells |
| 30 | FYB | ILMN_1236105 | FoxP3+T Cells |
| 31 | CD3E | ILMN_1238847 | FoxP3+T Cells |
| 32 | NOTCH1 | ILMN_1241915 | FoxP3+T Cells |
| 33 | CST7 | ILMN_1244891 | FoxP3+T Cells |
| 34 | IL18R1 | ILMN_1244895 | FoxP3+T Cells |
| 35 | CD84 | ILMN_1245754 | FoxP3+T Cells |
| 36 | SSBP2 | ILMN_1246193 | FoxP3+T Cells |
| 37 | TBXA2R | ILMN_1248837 | FoxP3+T Cells |
| 38 | SLAMF1 | ILMN_1249863 | FoxP3+T Cells |
| 39 | RAPGEF6 | ILMN_1254088 | FoxP3+T Cells |
| 40 | MS4A6B | ILMN_1254692 | FoxP3+T Cells |

| | | | |
|---|---|---|---|
| 41 | STK17B | ILMN_1255834 | FoxP3+T Cells |
| 42 | MCOLN3 | ILMN_1256430 | FoxP3+T Cells |
| 43 | SIT1 | ILMN_1258509 | FoxP3+T Cells |
| 44 | SP6 | ILMN_1258571 | FoxP3+T Cells |
| 45 | TMEM66 | ILMN_1258965 | FoxP3+T Cells |
| 46 | IGFBP4 | ILMN_1258988 | FoxP3+T Cells |
| 47 | TNFRSF18 | ILMN_2419490 | FoxP3+T Cells |
| 48 | TRIB2 | ILMN_2432550 | FoxP3+T Cells |
| 49 | TNIK | ILMN_2470251 | FoxP3+T Cells |
| 50 | SLA2 | ILMN_2474239 | FoxP3+T Cells |
| 51 | ZFP281 | ILMN_2477243 | FoxP3+T Cells |
| 52 | INADL | ILMN_2490495 | FoxP3+T Cells |
| 53 | EMB | ILMN_2547873 | FoxP3+T Cells |
| 54 | SATB1 | ILMN_2561103 | FoxP3+T Cells |
| 55 | SPN | ILMN_2574982 | FoxP3+T Cells |
| 56 | CD28 | ILMN_2589871 | FoxP3+T Cells |
| 57 | IL27RA | ILMN_2591156 | FoxP3+T Cells |
| 58 | RNF125 | ILMN_2594109 | FoxP3+T Cells |
| 59 | GIMAP7 | ILMN_2595918 | FoxP3+T Cells |
| 60 | DNAHC8 | ILMN_2602341 | FoxP3+T Cells |
| 61 | BCL11B | ILMN_2611022 | FoxP3+T Cells |
| 62 | ARHGEF18 | ILMN_2612125 | FoxP3+T Cells |
| 63 | MGST2 | ILMN_2613832 | FoxP3+T Cells |
| 64 | RAB27A | ILMN_2614966 | FoxP3+T Cells |
| 65 | DPP4 | ILMN_2615096 | FoxP3+T Cells |
| 66 | SOCS3 | ILMN_2618176 | FoxP3+T Cells |
| 67 | A630033H20 | ILMN_2622363 | FoxP3+T Cells |
| 68 | CMAH | ILMN_2626252 | FoxP3+T Cells |
| 69 | PELI1 | ILMN_2627441 | FoxP3+T Cells |
| 70 | TMEM71 | ILMN_2631610 | FoxP3+T Cells |
| 71 | THY1 | ILMN_2644350 | FoxP3+T Cells |
| 72 | BZW2 | ILMN_2644587 | FoxP3+T Cells |
| 73 | 4632428N05 | ILMN_2651297 | FoxP3+T Cells |
| 74 | SESN1 | ILMN_2654074 | FoxP3+T Cells |
| 75 | ITGAE | ILMN_2656090 | FoxP3+T Cells |
| 76 | DGKA | ILMN_2658961 | FoxP3+T Cells |
| 77 | LRBA | ILMN_2659960 | FoxP3+T Cells |
| 78 | LAT | ILMN_2660551 | FoxP3+T Cells |
| 79 | SCML4 | ILMN_2661185 | FoxP3+T Cells |
| 80 | SLFN1 | ILMN_2663930 | FoxP3+T Cells |
| 81 | PRKCQ | ILMN_2667829 | FoxP3+T Cells |
| 82 | KIF1B | ILMN_2670778 | FoxP3+T Cells |
| 83 | KLK8 | ILMN_2675232 | FoxP3+T Cells |

| | | | |
|---|---|---|---|
| 84 | ZC3H12D | ILMN_2680398 | FoxP3+T Cells |
| 85 | IL7R | ILMN_2680827 | FoxP3+T Cells |
| 86 | BCL2 | ILMN_2682162 | FoxP3+T Cells |
| 87 | ARID5B | ILMN_2684638 | FoxP3+T Cells |
| 88 | PHF11 | ILMN_2696491 | FoxP3+T Cells |
| 89 | CD3D | ILMN_2697415 | FoxP3+T Cells |
| 90 | RGS10 | ILMN_2699531 | FoxP3+T Cells |
| 91 | ADK | ILMN_2702471 | FoxP3+T Cells |
| 92 | CRLF3 | ILMN_2706803 | FoxP3+T Cells |
| 93 | GPR83 | ILMN_2707941 | FoxP3+T Cells |
| 94 | TIMP2 | ILMN_2712867 | FoxP3+T Cells |
| 95 | LASS4 | ILMN_2718499 | FoxP3+T Cells |
| 96 | CTSW | ILMN_2721399 | FoxP3+T Cells |
| 97 | CD3G | ILMN_2722784 | FoxP3+T Cells |
| 98 | FAM134B | ILMN_2745614 | FoxP3+T Cells |
| 99 | CD2 | ILMN_2753697 | FoxP3+T Cells |
| 100 | ARHGAP15 | ILMN_2759756 | FoxP3+T Cells |
| 101 | PIK3IP1 | ILMN_2769772 | FoxP3+T Cells |
| 102 | CYB5 | ILMN_2775098 | FoxP3+T Cells |
| 103 | LTA | ILMN_2780247 | FoxP3+T Cells |
| 104 | NT5E | ILMN_2813830 | FoxP3+T Cells |
| 105 | SELPLG | ILMN_2820893 | FoxP3+T Cells |
| 106 | 1600014C10 | ILMN_2822842 | FoxP3+T Cells |
| 107 | CD247 | ILMN_2828172 | FoxP3+T Cells |
| 108 | KLHL6 | ILMN_2840514 | FoxP3+T Cells |
| 109 | ACTN1 | ILMN_2844996 | FoxP3+T Cells |
| 110 | INPP4B | ILMN_2854354 | FoxP3+T Cells |
| 111 | ANXA6 | ILMN_2878060 | FoxP3+T Cells |
| 112 | ZBP1 | ILMN_2879614 | FoxP3+T Cells |
| 113 | IL2RG | ILMN_2886646 | FoxP3+T Cells |
| 114 | ADD3 | ILMN_2901283 | FoxP3+T Cells |
| 115 | FOXP3 | ILMN_2917180 | FoxP3+T Cells |
| 116 | GBP3 | ILMN_2918002 | FoxP3+T Cells |
| 117 | ARHGEF1 | ILMN_2920753 | FoxP3+T Cells |
| 118 | ARL5C | ILMN_2943057 | FoxP3+T Cells |
| 119 | DDIT4 | ILMN_2993109 | FoxP3+T Cells |
| 120 | OBFC2A | ILMN_2996904 | FoxP3+T Cells |
| 121 | NDRG2 | ILMN_3001650 | FoxP3+T Cells |
| 122 | ABHD8 | ILMN_3002505 | FoxP3+T Cells |
| 123 | SELL | ILMN_3009860 | FoxP3+T Cells |
| 124 | CD27 | ILMN_3052501 | FoxP3+T Cells |
| 125 | GLIPR2 | ILMN_3053593 | FoxP3+T Cells |
| 126 | TMSB10 | ILMN_3067068 | FoxP3+T Cells |

| | | | |
|---|---|---|---|
| 127 | GIMAP8 | ILMN_3088269 | FoxP3+T Cells |
| 128 | GRAP2 | ILMN_3113787 | FoxP3+T Cells |
| 129 | CD6 | ILMN_3117602 | FoxP3+T Cells |
| 130 | A430078G23 | ILMN_3163163 | FoxP3+T Cells |
| 131 | Blank | | |
| 132 | AMPD3 | ILMN_1212698 | Macrophages |
| 133 | YDJC | ILMN_1213274 | Macrophages |
| 134 | GRWD1 | ILMN_1215755 | Macrophages |
| 135 | CAMK1 | ILMN_1216632 | Macrophages |
| 136 | IFITM6 | ILMN_1218181 | Macrophages |
| 137 | PRMT7 | ILMN_1220228 | Macrophages |
| 138 | CD177 | ILMN_1225233 | Macrophages |
| 139 | TIMM10 | ILMN_1227235 | Macrophages |
| 140 | PYCRL | ILMN_1228613 | Macrophages |
| 141 | SLC43A1 | ILMN_1229667 | Macrophages |
| 142 | GSTZ1 | ILMN_1229964 | Macrophages |
| 143 | CLEC4A1 | ILMN_1230708 | Macrophages |
| 144 | TACSTD2 | ILMN_1231513 | Macrophages |
| 145 | RCL1 | ILMN_1237527 | Macrophages |
| 146 | RAI12 | ILMN_1238130 | Macrophages |
| 147 | CREG1 | ILMN_1239638 | Macrophages |
| 148 | CSF3R | ILMN_1241302 | Macrophages |
| 149 | CNPY2 | ILMN_1242881 | Macrophages |
| 150 | PLRG1 | ILMN_1244226 | Macrophages |
| 151 | KDELC1 | ILMN_1249999 | Macrophages |
| 152 | MS4A3 | ILMN_1250529 | Macrophages |
| 153 | FBXW8 | ILMN_1251236 | Macrophages |
| 154 | 1110008F13I | ILMN_1252407 | Macrophages |
| 155 | PRKAR2B | ILMN_1253819 | Macrophages |
| 156 | GCLM | ILMN_1256354 | Macrophages |
| 157 | HSD3B7 | ILMN_1260456 | Macrophages |
| 158 | TNFRSF21 | ILMN_2464573 | Macrophages |
| 159 | DAD1 | ILMN_2471534 | Macrophages |
| 160 | TNFAIP2 | ILMN_2474858 | Macrophages |
| 161 | PILRA | ILMN_2479717 | Macrophages |
| 162 | MRPS27 | ILMN_2588199 | Macrophages |
| 163 | MT1 | ILMN_2596522 | Macrophages |
| 164 | REPIN1 | ILMN_2605767 | Macrophages |
| 165 | PYGL | ILMN_2615015 | Macrophages |
| 166 | GART | ILMN_2622163 | Macrophages |
| 167 | ACSL1 | ILMN_2622671 | Macrophages |
| 168 | POMP | ILMN_2625352 | Macrophages |
| 169 | FZR1 | ILMN_2629971 | Macrophages |

| | | | |
|---|---|---|---|
| 170 | WDR55 | ILMN_2640453 | Macrophages |
| 171 | NUP133 | ILMN_2654013 | Macrophages |
| 172 | LIG1 | ILMN_2657356 | Macrophages |
| 173 | NDUFA9 | ILMN_2657694 | Macrophages |
| 174 | GSTT3 | ILMN_2665715 | Macrophages |
| 175 | SLC6A9 | ILMN_2667384 | Macrophages |
| 176 | JOSD2 | ILMN_2667614 | Macrophages |
| 177 | SLC39A11 | ILMN_2668706 | Macrophages |
| 178 | TBRG4 | ILMN_2670230 | Macrophages |
| 179 | THOP1 | ILMN_2674324 | Macrophages |
| 180 | SLC11A1 | ILMN_2674884 | Macrophages |
| 181 | MGAT4B | ILMN_2684268 | Macrophages |
| 182 | PCYOX1L | ILMN_2684575 | Macrophages |
| 183 | SORT1 | ILMN_2685772 | Macrophages |
| 184 | BCS1L | ILMN_2691345 | Macrophages |
| 185 | DYRK3 | ILMN_2699522 | Macrophages |
| 186 | PLSCR1 | ILMN_2701271 | Macrophages |
| 187 | SYNGR2 | ILMN_2706231 | Macrophages |
| 188 | SCAMP1 | ILMN_2706853 | Macrophages |
| 189 | S100A8 | ILMN_2710905 | Macrophages |
| 190 | LCN2 | ILMN_2712075 | Macrophages |
| 191 | CHI3L3 | ILMN_2712986 | Macrophages |
| 192 | ABHD4 | ILMN_2713464 | Macrophages |
| 193 | MUC13 | ILMN_2717678 | Macrophages |
| 194 | MPO | ILMN_2719256 | Macrophages |
| 195 | POLR1B | ILMN_2723483 | Macrophages |
| 196 | INTS7 | ILMN_2726174 | Macrophages |
| 197 | RRP12 | ILMN_2728118 | Macrophages |
| 198 | SIRPA | ILMN_2744683 | Macrophages |
| 199 | FPGS | ILMN_2747070 | Macrophages |
| 200 | 2610027L16R | ILMN_2750842 | Macrophages |
| 201 | PRTN3 | ILMN_2758029 | Macrophages |
| 202 | GFI1B | ILMN_2772930 | Macrophages |
| 203 | TMEM176A | ILMN_2795412 | Macrophages |
| 204 | S100A9 | ILMN_2803674 | Macrophages |
| 205 | 0610007P14R | ILMN_2808939 | Macrophages |
| 206 | MED24 | ILMN_2818189 | Macrophages |
| 207 | TGFBI | ILMN_2834379 | Macrophages |
| 208 | CD151 | ILMN_2836710 | Macrophages |
| 209 | SLC25A39 | ILMN_2855261 | Macrophages |
| 210 | PIGQ | ILMN_2877507 | Macrophages |
| 211 | TMBIM1 | ILMN_2908056 | Macrophages |
| 212 | TREM3 | ILMN_2915303 | Macrophages |

| | | | |
|---|---|---|---|
| 213 | CTPS | ILMN_2932964 | Macrophages |
| 214 | ANXA4 | ILMN_2935012 | Macrophages |
| 215 | FBXL6 | ILMN_2935032 | Macrophages |
| 216 | TRAPPC1 | ILMN_2943661 | Macrophages |
| 217 | RASSF4 | ILMN_2956095 | Macrophages |
| 218 | TSPAN31 | ILMN_2964076 | Macrophages |
| 219 | PARP1 | ILMN_2971744 | Macrophages |
| 220 | CMTM7 | ILMN_2985447 | Macrophages |
| 221 | ASNS | ILMN_3006123 | Macrophages |
| 222 | CD63 | ILMN_3052430 | Macrophages |
| 223 | UNG | ILMN_3056503 | Macrophages |
| 224 | NAIP5 | ILMN_3059557 | Macrophages |
| 225 | ATIC | ILMN_3070951 | Macrophages |
| 226 | RBPMS | ILMN_3099398 | Macrophages |
| 227 | FTSJ1 | ILMN_3135668 | Macrophages |
| 228 | ANXA3 | ILMN_3135781 | Macrophages |
| 229 | WDR6 | ILMN_3162081 | Macrophages |
| 230 | Blank | | |
| 231 | AMPD3 | ILMN_1212698 | Monocytes |
| 232 | PLXND1 | ILMN_1214608 | Monocytes |
| 233 | CLN3 | ILMN_1214952 | Monocytes |
| 234 | CAMK1 | ILMN_1216632 | Monocytes |
| 235 | AIFM2 | ILMN_1216842 | Monocytes |
| 236 | IFITM6 | ILMN_1218181 | Monocytes |
| 237 | TM9SF4 | ILMN_1220640 | Monocytes |
| 238 | LMAN2L | ILMN_1220769 | Monocytes |
| 239 | PLEKHG3 | ILMN_1221920 | Monocytes |
| 240 | ROGDI | ILMN_1222821 | Monocytes |
| 241 | CD177 | ILMN_1225233 | Monocytes |
| 242 | SCAMP2 | ILMN_1226985 | Monocytes |
| 243 | ZEB2 | ILMN_1227793 | Monocytes |
| 244 | PIAS3 | ILMN_1227889 | Monocytes |
| 245 | PYCRL | ILMN_1228613 | Monocytes |
| 246 | CLEC4A1 | ILMN_1230708 | Monocytes |
| 247 | CREG1 | ILMN_1239638 | Monocytes |
| 248 | SLC7A7 | ILMN_1240318 | Monocytes |
| 249 | CSF3R | ILMN_1241302 | Monocytes |
| 250 | OLFM1 | ILMN_1241371 | Monocytes |
| 251 | SGPL1 | ILMN_1245609 | Monocytes |
| 252 | CASP1 | ILMN_1247592 | Monocytes |
| 253 | NQO2 | ILMN_1247930 | Monocytes |
| 254 | DUSP6 | ILMN_1248537 | Monocytes |
| 255 | KDELC1 | ILMN_1249999 | Monocytes |

| | | | |
|---|---|---|---|
| 256 | MS4A3 | ILMN_1250529 | Monocytes |
| 257 | FBXW8 | ILMN_1251236 | Monocytes |
| 258 | 1110008F13R | ILMN_1252407 | Monocytes |
| 259 | HSD3B7 | ILMN_1260456 | Monocytes |
| 260 | TYK2 | ILMN_2455701 | Monocytes |
| 261 | TNFRSF21 | ILMN_2464573 | Monocytes |
| 262 | DAD1 | ILMN_2471534 | Monocytes |
| 263 | TNFAIP2 | ILMN_2474858 | Monocytes |
| 264 | PILRA | ILMN_2479717 | Monocytes |
| 265 | VNN3 | ILMN_2491202 | Monocytes |
| 266 | GM962 | ILMN_2531520 | Monocytes |
| 267 | TMEM51 | ILMN_2589741 | Monocytes |
| 268 | UBE2F | ILMN_2590350 | Monocytes |
| 269 | PYGL | ILMN_2615015 | Monocytes |
| 270 | GART | ILMN_2622163 | Monocytes |
| 271 | ACSL1 | ILMN_2622671 | Monocytes |
| 272 | POMP | ILMN_2625352 | Monocytes |
| 273 | FZR1 | ILMN_2629971 | Monocytes |
| 274 | NUP133 | ILMN_2654013 | Monocytes |
| 275 | LIG1 | ILMN_2657356 | Monocytes |
| 276 | STXBP2 | ILMN_2657728 | Monocytes |
| 277 | SLC39A11 | ILMN_2668706 | Monocytes |
| 278 | SLC11A1 | ILMN_2674884 | Monocytes |
| 279 | ATP6V1B2 | ILMN_2680440 | Monocytes |
| 280 | MGAT4B | ILMN_2684268 | Monocytes |
| 281 | PCYOX1L | ILMN_2684575 | Monocytes |
| 282 | SORT1 | ILMN_2685772 | Monocytes |
| 283 | FAM129B | ILMN_2686975 | Monocytes |
| 284 | MTMR14 | ILMN_2689062 | Monocytes |
| 285 | CD44 | ILMN_2697830 | Monocytes |
| 286 | SYNGR2 | ILMN_2706231 | Monocytes |
| 287 | NLRX1 | ILMN_2708222 | Monocytes |
| 288 | S100A8 | ILMN_2710905 | Monocytes |
| 289 | LCN2 | ILMN_2712075 | Monocytes |
| 290 | 1810033B17I | ILMN_2712151 | Monocytes |
| 291 | CHI3L3 | ILMN_2712986 | Monocytes |
| 292 | ABHD4 | ILMN_2713464 | Monocytes |
| 293 | MPO | ILMN_2719256 | Monocytes |
| 294 | MAPK14 | ILMN_2721809 | Monocytes |
| 295 | TLR2 | ILMN_2733733 | Monocytes |
| 296 | WWP2 | ILMN_2744245 | Monocytes |
| 297 | SIRPA | ILMN_2744683 | Monocytes |
| 298 | PLEKHO2 | ILMN_2749448 | Monocytes |

| | | | |
|---|---|---|---|
| 299 | 2610027L16R | ILMN_2750842 | Monocytes |
| 300 | BID | ILMN_2757287 | Monocytes |
| 301 | PRTN3 | ILMN_2758029 | Monocytes |
| 302 | LRRC8D | ILMN_2766455 | Monocytes |
| 303 | TMEM38B | ILMN_2795791 | Monocytes |
| 304 | S100A9 | ILMN_2803674 | Monocytes |
| 305 | HMGCL | ILMN_2822131 | Monocytes |
| 306 | TGFBI | ILMN_2834379 | Monocytes |
| 307 | CD151 | ILMN_2836710 | Monocytes |
| 308 | SLC25A39 | ILMN_2855261 | Monocytes |
| 309 | COPZ1 | ILMN_2862093 | Monocytes |
| 310 | CSF2RA | ILMN_2866276 | Monocytes |
| 311 | CDC42BPB | ILMN_2867696 | Monocytes |
| 312 | MOCOS | ILMN_2906489 | Monocytes |
| 313 | TMBIM1 | ILMN_2908056 | Monocytes |
| 314 | TREM3 | ILMN_2915303 | Monocytes |
| 315 | ANXA4 | ILMN_2935012 | Monocytes |
| 316 | FBXL6 | ILMN_2935032 | Monocytes |
| 317 | IRAK2 | ILMN_2937735 | Monocytes |
| 318 | TRAPPC1 | ILMN_2943661 | Monocytes |
| 319 | RASSF4 | ILMN_2956095 | Monocytes |
| 320 | TSPAN31 | ILMN_2964076 | Monocytes |
| 321 | CMTM7 | ILMN_2985447 | Monocytes |
| 322 | SLC15A3 | ILMN_2987709 | Monocytes |
| 323 | NAIP5 | ILMN_3059557 | Monocytes |
| 324 | RBPMS | ILMN_3099398 | Monocytes |
| 325 | PTPN6 | ILMN_3113420 | Monocytes |
| 326 | ANXA3 | ILMN_3135781 | Monocytes |
| 327 | TBC1D2 | ILMN_3140913 | Monocytes |
